# Design, Synthesis, In Vitro and In Vivo Evaluation of Heterobivalent SiFA*lin*-Modified Peptidic Radioligands Targeting Both Integrin α_v_β_3_ and the MC1 Receptor—Suitable for the Specific Visualization of Melanomas?

**DOI:** 10.3390/ph14060547

**Published:** 2021-06-07

**Authors:** Xia Cheng, Ralph Hübner, Valeska von Kiedrowski, Gert Fricker, Ralf Schirrmacher, Carmen Wängler, Björn Wängler

**Affiliations:** 1 Molecular Imaging and Radiochemistry, Department of Clinical Radiology and Nuclear Medicine, Medical Faculty Mannheim of Heidelberg University, Theodor-Kutzer-Ufer 1–3, 68167 Mannheim, Germany; Xia.Cheng@medma.uni-heidelberg.de (X.C.); valeska.vonkiedrowski@medma.uni-heidelberg.de (V.v.K.); 2 Biomedical Chemistry, Department of Clinical Radiology and Nuclear Medicine, Medical Faculty Mannheim of Heidelberg University, Theodor-Kutzer-Ufer 1–3, 68167 Mannheim, Germany; Ralph.Huebner@medma.uni-heidelberg.de; 3 Institute of Pharmacy and Molecular Biotechnology, University of Heidelberg, Im Neuenheimer Feld 329, 69120 Heidelberg, Germany; gert.fricker@uni-hd.de; 4 Department of Oncology, Division of Oncological Imaging, University of Alberta, 11560 University Avenue, Edmonton, AB T6G 1Z2, Canada; schirrma@ualberta.ca

**Keywords:** malignant melanoma, ^18^F, SiFAlin, MC1R, α_v_β_3_, heterobivalent peptidic ligands, PET/CT imaging

## Abstract

Combining two peptides addressing two different receptors to a heterobivalent peptidic ligand (HBPL) is thought to enable an improved tumor-targeting sensitivity and thus tumor visualization, compared to monovalent peptide ligands. In the case of melanoma, the Melanocortin-1 receptor (MC1R), which is stably overexpressed in the majority of primary malignant melanomas, and integrin α_v_β_3_, which is involved in lymph node metastasis and therefore has an important role in the transition from local to metastatic disease, are important target receptors. Thus, if a radiolabeled HBPL could be developed that was able to bind to both receptor types, the early diagnosis and correct staging of the disease would be significantly increased. Here, we report on the design, synthesis, radiolabeling and in vitro and in vivo testing of different SiFA*lin*-modified HBPLs (SiFA = silicon fluoride acceptor), consisting of an MC1R-targeting (GG-Nle-c(DHfRWK)) and an integrin α_v_β_3_-affine peptide (c(RGDfK)), being connected by a symmetrically branching framework including linkers of differing length and composition. Kit-like ^18^F-radiolabeling of the HBPLs **1**–**6** provided the labeled products [^18^F]**1**–[^18^F]**6** in radiochemical yields of 27–50%, radiochemical purities of ≥95% and non-optimized molar activities of 17–51 GBq/μmol within short preparation times of 25 min. Besides the evaluation of radiotracers regarding log*_D_*_(7.4)_ and stability in human serum, the receptor affinities of the HBPLs were investigated in vitro on cell lines overexpressing integrin α_v_β_3_ (U87MG cells) or the MC1R (B16F10). Based on these results, the most promising compounds [^18^F]**2**, showing the highest affinity to both target receptors (IC_50 (B16F10)_ = 0.99 ± 0.11 nM, IC_50 (U87MG)_ = 1300 ± 288 nM), and [^18^F]**4**, exhibiting the highest hydrophilicity (log*_D_*_(7.4)_ = −1.39 ± 0.03), were further investigated in vivo and ex vivo in a xenograft mouse model bearing both tumors. For both HBPLs, clear visualization of B16F10, as well as U87MG tumors, was feasible. Blocking studies using the respective monospecific peptides demonstrated both peptide binders of the HBPLs contributing to tumor uptake. Despite the somewhat lower target receptor affinities (IC_50 (B16F10)_ = 6.00 ± 0.47 nM and IC_50 (U87MG)_ = 2034 ± 323 nM) of [^18^F]**4**, the tracer showed higher absolute tumor uptakes ([^18^F]**4**: 2.58 ± 0.86% ID/g in B16F10 tumors and 3.92 ± 1.31% ID/g in U87MG tumors; [^18^F]**2**: 2.32 ± 0.49% ID/g in B16F10 tumors and 2.33 ± 0.46% ID/g in U87MG tumors) as well as higher tumor-to-background ratios than [^18^F]**2**. Thus, [^18^F]**4** demonstrates to be a highly potent radiotracer for the sensitive and bispecific imaging of malignant melanoma by PET/CT imaging and impressively illustrates the suitability of the underlying concept to develop heterobivalent integrin α_v_β_3_- and MC1R-bispecific radioligands for the sensitive and specific imaging of malignant melanoma by PET/CT.

## 1. Introduction

With a global incidence increasing over the last decades and being among the tumor types with the most increasing prevalence in Europe, malignant melanoma (MM) is the most aggressive type of skin cancer. The probability of developing the disease is increasing for people with a large number of melanocytic nevi, a fair skin type and genetic predisposition [1,2,3]. Repeated exposure to strong UV (ultraviolet) radiation through recurrent intense sun exposure is the most important environmental risk factor [4]. In most cases, an early diagnosis enables a complete surgical removal and thus the patient to be cured. However, early detection is often not possible since the disease has no particular symptoms, and the tumors can rapidly progress from the fully encapsulated stage to infiltrative growth. In the case of basal membrane penetration, the tumor has access to the blood and lymph vessels, and metastases can be formed in organs or lymph nodes [5]. Since a cure is rarely possible when metastasis has already occurred, an early, very sensitive and specific diagnosis of the disease is of the highest importance. Moreover, the correct staging of the disease is critical, as only, in this case, can an appropriate therapy, having the potential to cure the patient, be chosen.

However, primary diagnosis using positron emission tomography (PET), which has the highest sensitivity compared to other whole-body imaging techniques such as computed tomography (CT) or magnetic resonance imaging (MRI), is often not suitable to correctly identify MM lesions. One drawback of the commonly used radiotracer [^18^F]FDG (2-[^18^F]fluoro-2-deoxyglucose) is its accumulation in inflamed tissues, giving false-positive results. Furthermore, the detection of slowly growing lesions is often difficult as well, resulting in possible false-negative imaging results [6]. Since the tumor visualization sensitivity and specificity using [^18^F]FDG can be low, an early and correct diagnosis and staging are often not possible. An alternative to unspecific, metabolically driven imaging is addressing the tumor by a tumor-specific radiotracer. For this purpose, receptors that are overexpressed in the tumor cell surface are especially useful. In the case of MM, the MC1R is best suited, as this receptor type is overexpressed in about 80% of MM primaries [7,8] and thus is a highly important target structure for MM-specific imaging. However, not all lesions express the MC1R, resulting in an incomplete visualization of the tumor load and thus false staging of the disease. In order to improve the diagnostic imaging of MM and enable an adequate, early and sensitive diagnosis and correct staging, a reliable and sensitive imaging method for MM is needed. Therefore, the development of target-specific accumulating agents that are able to address more than just the MC1R is mandatory.

Such agents should be based on radiolabeled peptides being able to bind with high affinity and specificity to surface receptors overexpressed by malignant cells and thus, enable the distinction between benign and malignant tissue. Ideally, radiolabeled peptides exhibit favorable tumor-to-background ratios, due to their tumor-specific accumulation, and thus produce images of high quality. Furthermore, peptides exhibit low toxicity and immunogenicity, are easily synthetically accessible and can be chemically modified at defined sites. Their pharmacokinetics prove to be very advantageous due to rapid tissue penetration, target accumulation and elimination from non-target tissues [9,10]. Therefore, numerous radiolabeled peptides have been developed for both the diagnosis and therapy of malignancies over the last decades [11,12].

Heterobivalent peptidic ligands (HBPLs), consisting of a radionuclide and two different peptides, each addressing its respective target receptor, have the advantage of a higher target avidity compared to monovalent peptide ligands by being able to bind simultaneously or independently to different target receptors on the tumor surface, resulting in stronger binding to the target cell [13]. Furthermore, HBPLs usually exhibit higher metabolic stability than their respective monomers against peptidases, due to their higher molecular weight and introduction of artificial structural elements [14]. The prerequisite for an HBPL with high tumor visualization potential is at least a moderate binding affinity of each of the included peptides to their target receptors. Ideally, both receptor types should be present in high density to achieve a concomitant binding of both peptide binders of the HBPL; however, the presence of only one target receptor is sufficient to achieve a high tumor uptake [9,15,16,17], resulting in an overall improved imaging sensitivity.

**Figure 1:**
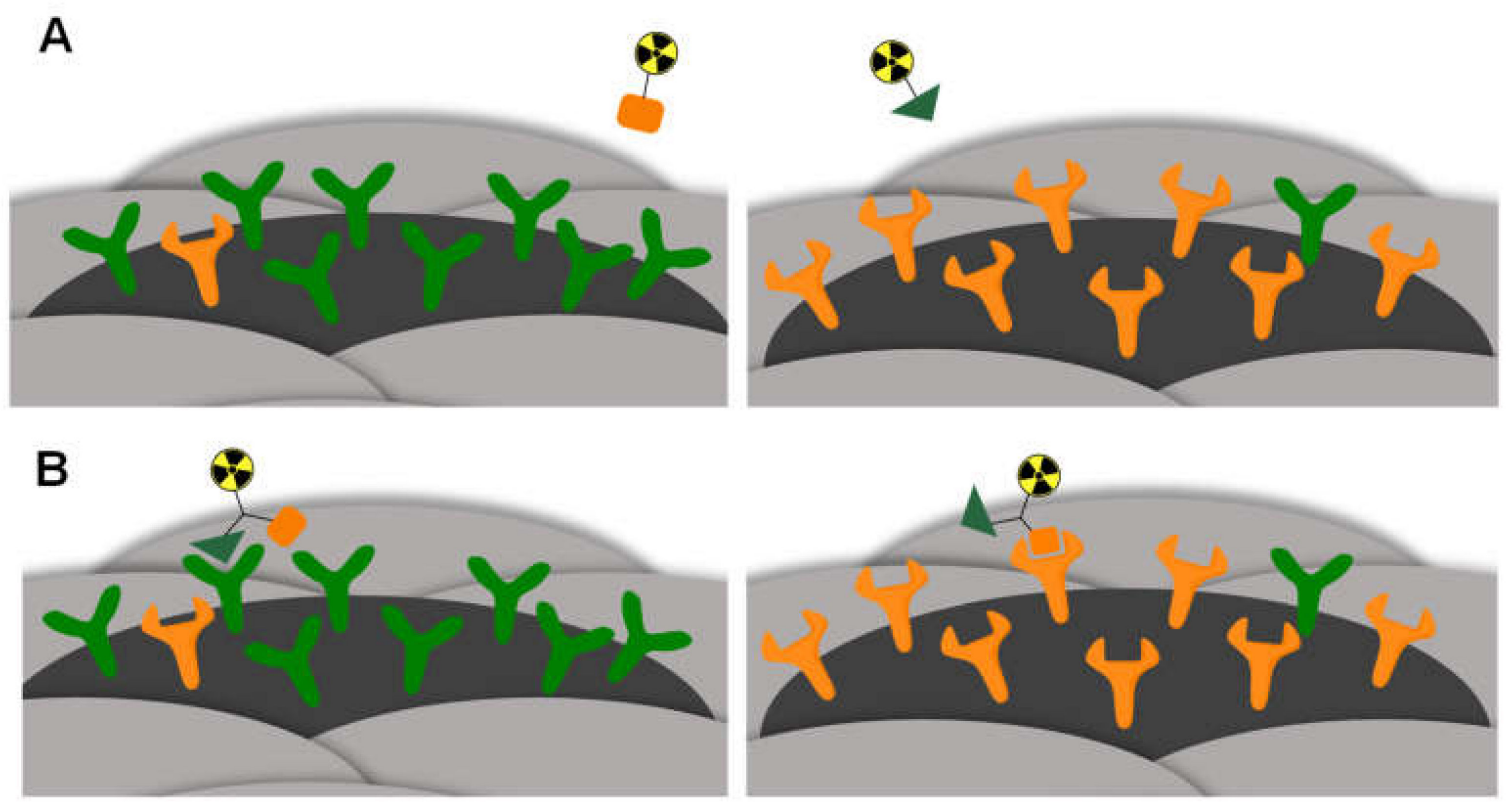
Schematic depiction of the concept of HBPL application exemplified by a comparison of a radiolabeled monospecific (**A**) or heterobivalent peptidic ligand (**B**) binding to tumor entities overexpressing different receptor types. In the case of (**A**), no binding is possible since the respective target receptor is only expressed to a low extent. In the case of (**B**), the HBPL can bind since at least one of the target receptors is expressed on the tumor surface.

In contrast to HBPLs, monovalent peptides, being able to address only one receptor type and thus only visualize tumors that overexpress this particular receptor, can result in limited tumor visualization sensitivity, as tumor cells can overexpress different receptor types. In such cases of inhomogeneous receptor expression, which can further be caused by tumor dedifferentiation, metastasis or triggered by therapy, the target receptor for the monospecific binder can be absent or present in insufficient density [18,19,20]. This results in an insufficient sensitivity of the peptides’ tumor delineation (Figure 1A). In contrast, HBPLs have the advantage of binding to more than one receptor type and thus exhibit a high tumor visualization efficiency (Figure 1B) [9,20].

For the development of HBPLs, some requirements have to be fulfilled. The peptides have to be modified as little as possible in their chemical structure to preserve their binding affinities to their corresponding receptors. In particular, the pharmacophoric site has to remain unchanged. Furthermore, it is important to determine which receptor types are overexpressed in a tumor entity and thus can be addressed by the radioligand to be developed [9,20]. For this purpose, many studies have been performed within recent years regarding the available receptor types on different human malignancies [21]. The results obtained serve as a guideline for the choice of peptidic receptor ligands, yielding potent tumor-targeting HBPLs with highly sensitive visualization properties.

For MM, the MC1R represents one especially useful target structure for the specific imaging of the disease (vide supra). Another receptor type that is of high potential for MM imaging is integrin α_v_β_3_, as it was shown that this receptor is overexpressed in the blood vessels of many human tumors [22,23,24]. Further studies revealed the involvement of integrin α_v_β_3_ in the progression of the disease and in the change of tumor growth from radial to vertical (thus infiltrative) growth [25,26,27,28,29,30]. Therefore, integrin α_v_β_3_, although overexpressed in all neo-angiogenetic processes, is also an important marker protein for MM targeting.

Thus, HBPLs based on MC1R- and integrin α_v_β_3_-affine peptides would be most promising for visualizing MM during all stages of the disease, enabling a highly sensitive and especially correct assessment of the extent of the disease. This is of crucial importance for choosing the optimal therapy approach, adapted to the extent of the disease: an encapsulated tumor can be treated differently than an infiltratively growing or already metastatic tumor. A high sensitivity to tumor imaging, surely identifying all tumor mass, is thus the prerequisite for the choice of the best-suited therapy option.

So far, the concept to develop an HBPL based on an MC1R-specific peptide ([Cys^3,4,10^, DPhe^7^, Arg^11^]αMSH_3-13_) and an integrin α_v_β_3_-affine peptide (c(RGDyD) (cyclic Arg-Gly-Asp-DTyr-Asp)) has only been described once for the radiotracer ^99m^Tc-RGD-Lys-(Arg^11^)CCMSH intended for tumor therapy driven by caspase-3-induced apoptosis induction [31]. The evaluation of this compound was performed in vitro on MC1R-exhibiting B16F1 cells and in vivo in B16F1 melanoma-bearing mice. High binding affinity and tumor uptake, but also a high renal uptake, were detected for this tracer. Therefore, structural modifications are mandatory to obtain an HBPL with more favorable in vivo pharmacokinetics.

In the present study, we developed different radiolabeled MC1R- and α_v_β_3_-bispecific HBPLs. These were based on the α_v_β_3_-affine peptide c(RGDfK) (cyclic Arg-Gly-Asp-DPhe-Lys), showing high stability and integrin target affinity [32], and the macrocyclic lactam GG-Nle-c(DHfRWK) (Gly-Gly-Nle-cyclic Asp-His-DPhe-Arg-Trp-Lys), giving excellent results in terms of MC1R target affinity and stability against proteolytic degradation as well [33,34].

As no HBPLs based on these peptidic ligands have been described so far, we intended to assess the general feasibility of this concept and to develop different HBPLs, consisting of the mentioned peptidic binders, a SiFA*lin*-moiety (for efficient radiolabeling of the HBPL with the positron-emitting nuclide ^18^F) and a varying molecular design. The molecular scaffold for the HBPLs was based on a symmetrical branching unit exhibiting linkers of different lengths and compositions so as to be able to systematically determine the influence of the used linker type and length on the biological parameters of the resulting HBPLs. The developed agents were labeled with ^18^F and evaluated in vitro regarding their lipophilicity, stability in human serum and especially their binding affinity to the respective target receptors. Finally, the most promising ^18^F-labeled derivatives were evaluated in vivo, in terms of their tumor visualization potential, in an appropriate preclinical tumor model using PET/CT imaging and ex vivo biodistribution experiments.

## 2. Results and Discussion

### 2.1. General Considerations for the Design of the Heterobivalent SiFAlin-Modified Peptidic Ligands

**Figure 2:**
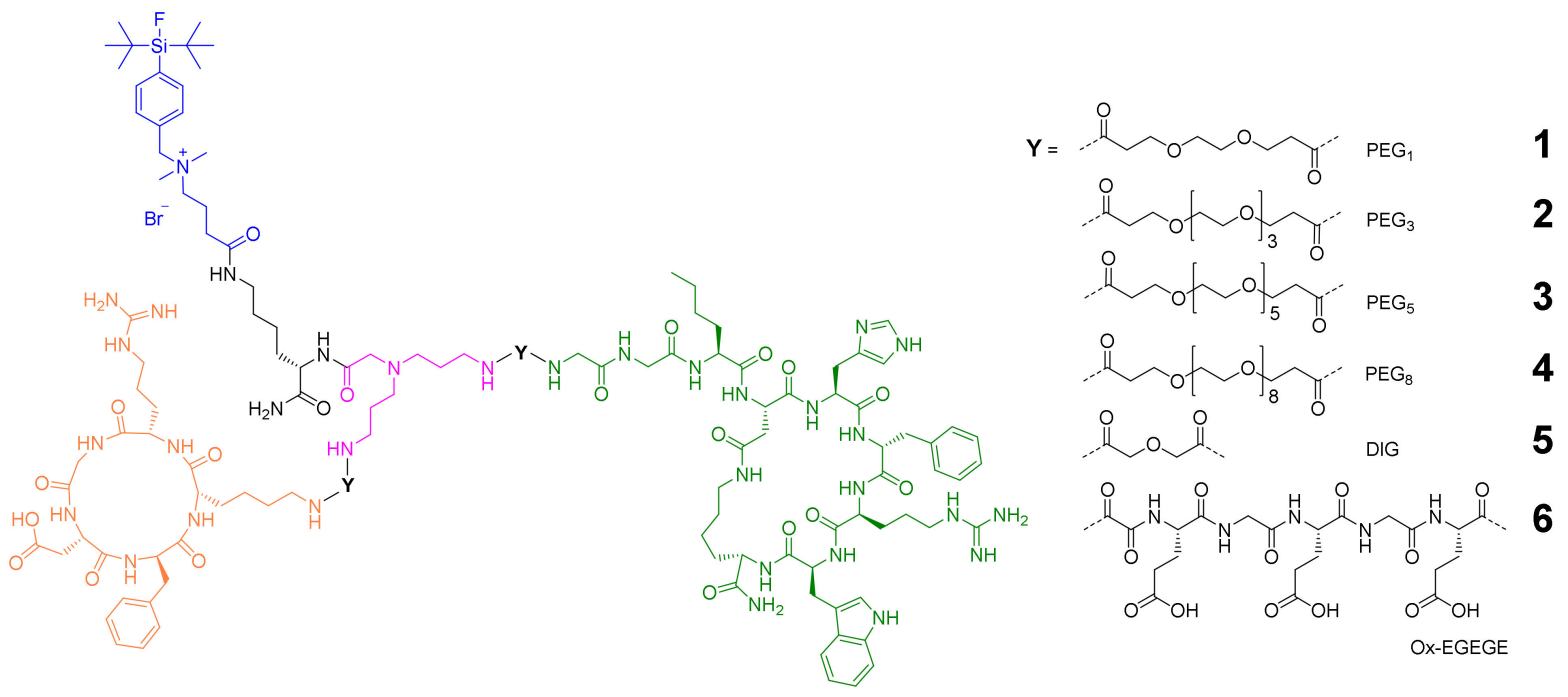
Depiction of the structures of the target HBPLs **1**–**6** consisting of: a SiFA*lin*-moiety (**blue**); a short lysine linker (**black**); the symmetrically branching framework (**pink**); linkers **Y** of different lengths and compositions (PEG_1_, PEG_3_, PEG_5_, PEG_8_, DIG, Ox-EGEGE; PEG = polyethylene glycol; DIG = diglycolic acid; Ox = oxalic acid); the MC1R- and integrin α_v_β_3_-affine peptides GG-Nle-c(DHfRWK) (**green**) and c(RGDfK) (**orange**).

The molecular design of the target compounds (Figure 2) included two different peptides, each addressing specifically one of the two target receptors—c(RGDfK) for integrin α_v_β_3_ and GG-Nle-c(DHfRWK) for MC1R binding—and was based on the following conditions: (i) The HBPLs should contain a SiFA*lin*-moiety exhibiting a permanent positive charge. With this SiFA*lin* building block, the radionuclide ^18^F can be efficiently introduced in one step [35]; (ii) the required molecular building blocks should be connected by a small symmetrically branched framework resulting in homogeneous compounds [9,36]; (iii) a lysine spacer should be introduced between the SiFA*lin*-moiety and the branched framework to achieve a spatial distance between the SiFA*lin* and the peptides, preventing interference with the peptide–receptor interaction, and to obtain the products in higher radiochemical yields [9,20,37,38]; (iv) as much as possible, the syntheses should be carried out on a solid support to facilitate the assembly of the rather complex target molecules; (v) different linker structures should be introduced between the peptides and the branching unit to systematically determine the optimal distance between both peptides. An optimal distance between the peptide binders enables the binding of each peptide to the respective receptor while remaining not interfered with by the second peptide and, at the same time, does not result in a high entropy, limiting the benefits of peptide heterodimerization [15,39,40,41,42,43]. Since the synthetic effort for the SiFA*lin*-linked framework is higher than that of the peptides, the linkers should be introduced as *bis*-NHS (*N*-hydroxysuccinimide) active esters on the peptidic side by derivatizing the *N_ɛ_*-amine of lysine of c(RGDfK) and the *N_α_*-amine of glycine of GG-Nle-c(DHfRWK), or the *N_α_*-amine of glutamic acid for the EGEGE peptides. Regarding the order of peptide-to-framework conjugations, the smaller c(RGDfK)-based peptides should be reacted first, followed by the bulkier GG-Nle-c(DHfRWK) peptides, to achieve higher product yields.

### 2.2. Synthesis of the Heterobivalent SiFAlin-Modified Peptidic Ligands

**Scheme 1:**
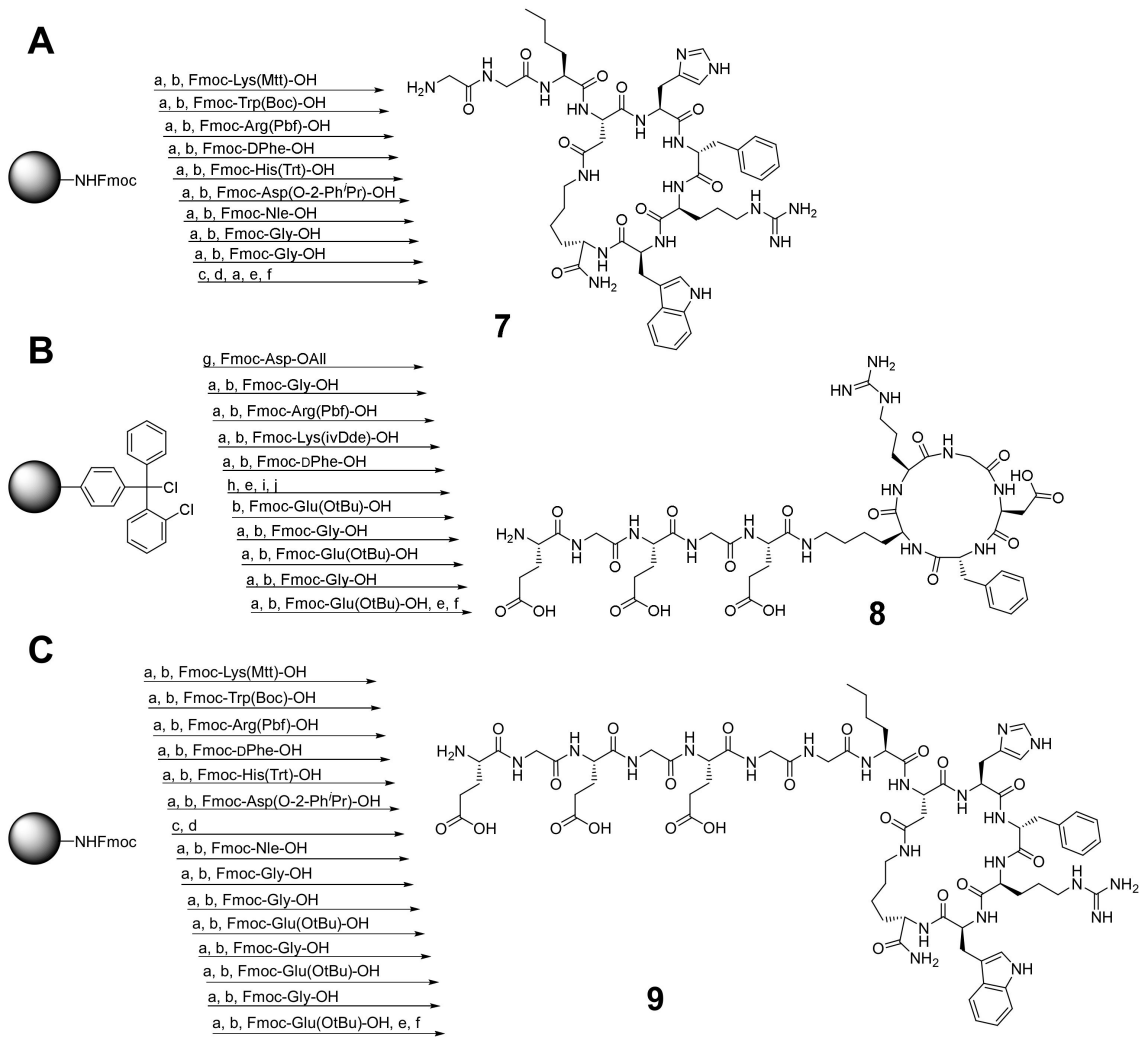
(**A**) Synthesis of GG-Nle-c(DHfRWK)-peptide (**7**); isolated yield: 42%. (**B**) Synthesis of c(RGDfK)-EGEGE-peptide (**8**); isolated yield: 41%. (**C**) Synthesis of EGEGE-GG-Nle-c(DHfRWK)-peptide (**9**); isolated yield: 43%. Conditions: (**a**) deprotection of Fmoc-PG: piperidine/DMF (1/1, *v*/*v*), 2 + 5 min; (**b**) activation of amino acids: 4.0 equiv. DIPEA, 3.9 equiv. HBTU in DMF, 2 min, coupling, 60 min; (**c**) deprotection of Mtt- and *O*-2-Ph*^i^*Pr-PG: TFA/CH_2_Cl_2_ (1/99, *v*/*v*), 90 min; (**d**) cyclization: 4.0 equiv. DIPEA, 3.9 equiv. PyBOP in DMF, 20 h; (**e**) deprotection of Fmoc-PG: piperidine/DMF (1/1, *v*/*v*), 2 × 10 min; (**f**) cleavage from resin and deprotection: TFA/TIS/H_2_O (95/2.5/2.5, *v*/*v*/*v*), 3 h; (**g**) conjugation: 4.0 equiv. DIPEA in CH_2_Cl_2_, 4 h; (**h**) deprotection of All-PG: 24.0 equiv. PhSiH_3_, 0.25 equiv. Pd(PPh_3_)_4_ in CH_2_Cl_2_, 3 × 30 min; (**i**) cyclization: 4.0 equiv. DIPEA, 3.9 equiv. PyBOP in DMF, 12 h; (**j**) deprotection of ivDde-PG: hydrazine/DMF (2/98, *v*/*v*), 2 × 10 min. DIPEA = *N,N*-diisopropylethylamine, DMF = dimethylformamide, HBTU = 2-(1*H*-benzotriazol-1-yl)-1,1,3,3-tetramethyluronium hexafluorophosphate, PyBOP = benzotriazol-1-yloxytripyrrolidino-phosphonium hexafluorophosphate, TFA = trifluoroacetic acid, TIS = triisopropylsilane.

For the assembly of the SiFA*lin*-modified HBPLs **1**–**6**, the monomeric peptides were synthesized according to standard Fmoc-based solid-phase peptide synthesis (SPPS) protocols. The c(RGDfK)-peptide was synthesized according to a known procedure [44] and was obtained in an overall yield of 83%. For the synthesis of the peptide GG-Nle-c(DHfRWK) (**7**) (Scheme 1A), all amino acids were coupled on a rink amide resin. After deprotection of the acid-labile protecting groups (PG)–Mtt and *O*-2-Ph*^i^*Pr–under mildly acidic conditions, the cyclization, deprotection and cleavage from the resin were performed. By optimizing the reaction conditions, peptide **7** was isolated in an overall yield of 42%.

For the synthesis of the charged HBPL **6**, further amino acids had to be added to the monovalent peptides. For c(RGDfK)-EGEGE (**8**) (Scheme 1B), c(RGDfK) was first synthesized according to a known procedure [45] and was then modified with the glutamic acids and glycines at the *N*_ɛ_-amine of lysine (still on a solid support) before the deprotection and cleavage from the resin were carried out. Peptide **8** was obtained in an overall yield of 41%. EGEGE-GG-Nle-c(DHfRWK) (**9**) (Scheme 1C) was prepared in a different way than GG-Nle-c(DHfRWK)-peptide. First, only the first six amino acids were conjugated to the resin. Afterward, the cyclization was performed, and only then the following conjugation of the remaining amino acids followed by the cleavage from the resin was performed. Peptide **9** was obtained in an overall yield of 43% following this route (for analytical data, see Supplementary Materials Figures S1–S3).

After successfully establishing the synthesis of the monovalent peptides c(RGDfK) and **7**–**9**, modification of the peptides with the different linkers (PEG_1_, PEG_3_, PEG_5_, PEG_8_ and DIG) was performed as follows.

**Scheme 2:**
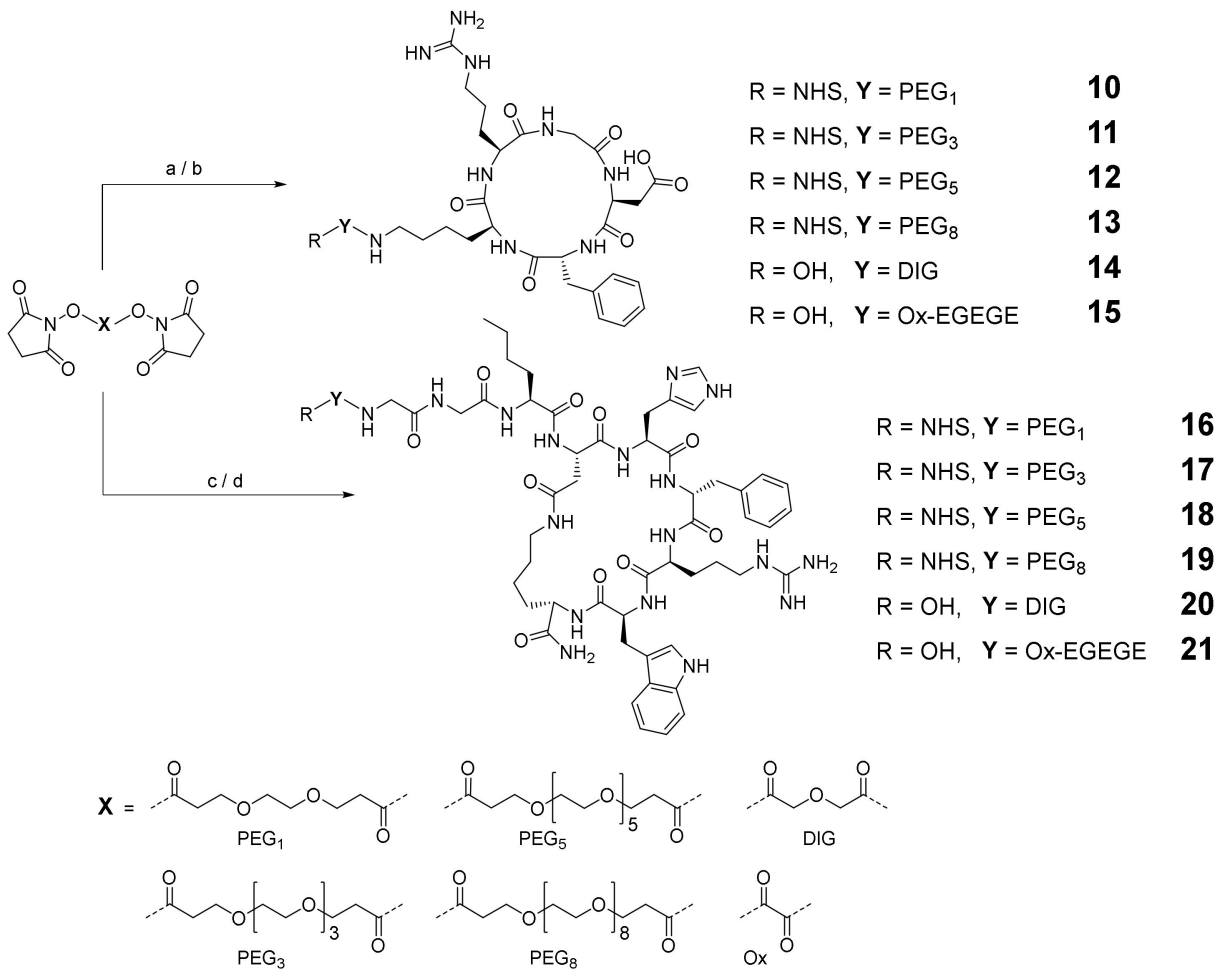
Depiction of the synthesis strategy to obtain the c(RGDfK)–linker conjugates **10**–**15** and GG-Nle-c(DHfRWK)–linker conjugates **16**–**21**. Conditions: (**a**) c(RGDfK), DIPEA, DMF, yields: 41% for **10**, 29% for **11**, 35% for **12**, 32% for **13**, 37% for **14**; (**b**) **8**, DIPEA, DMF, 40% yield for **15**; (**c**) **7**, DIPEA, DMF, yields: 43% for **16**, 49% for **17**, 42% for **18**, 42% for **19**, 61% for **20**; (**d**) **9**, DIPEA, DMF, 44% yield for **21**.

For the evaluation of the optimal conditions to obtain the peptide–linker conjugates **10**–**21** (Scheme 2), which can be used for heterodimer synthesis, different solvent/base systems were tested during the reactions of the peptide c(RGDfK) and **7**–**9** with the respective *bis*-NHS esters of the linkers introduced to obtain the conjugates **10**–**21**. The best results in terms of isolated yields were found for DIPEA in DMF. Besides the target NHS-PEG_n_ peptides **10**–**13** and **16**–**19**, small amounts of hydrolyzed compound and homodimer were also isolated. However, in the case of the DIG- and Ox-linker, only the hydrolyzed carboxylic acids **14**, **15**, **20** and **21** could be isolated, although the reason for this is not obvious. For further reactions of these agents with the framework structure, in order to obtain the target HBPLs, these free carboxylic acid-comprising peptides had to be pre-activated with a suitable activation reagent (for analytical data, see Supplementary Materials Figures S4–S15).

**Scheme 3:**
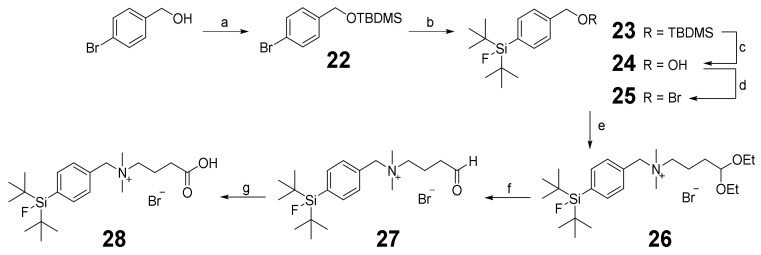
Schematic depiction of the synthesis of SiFA*lin* acid **28**. Conditions: (**a**) TBDMS-Cl, imidazole, DMF, 98% yield; (**b**) SiF_2_(*^t^*Bu)_2_, *^t^*BuLi, Et_2_O, 92% yield; (**c**) HCl, MeOH, 99% yield; (**d**) CBr_4_, PPh_3_, CH_2_Cl_2_, 95% yield; (**e**) 4,4-diethoxy-*N,N*-dimethylbutan-1-amine, CH_2_Cl_2_, 93% yield; (**f**) 95% TFA, 91% yield; (**g**) KMnO_4_, NaH_2_PO_4_, *^t^*BuOH/CH_2_Cl_2_, 69% yield.

To obtain the SiFA*lin* building block **28** (Scheme 3), the acetal **26** was synthesized following a published procedure [46,47,48] with some modifications. First, the hydroxyl function of the 4-bromobenzyl alcohol was protected with TBDMS-Cl (*tert*-butyldimethylsilyl chloride) to produce **22**, then the SiFA unit was introduced by an in-situ-preceding halogen–metal exchange with subsequent transmetalation to produce **23**. After the acidic deprotection of the TBDMS-PG, the resulting alcohol **24** was transferred to the bromide **25** by an Appel reaction. Amination of **25** with 4,4-diethoxy-*N,N*-dimethylbutan-1-amine led to the desired acetal **26**. After the acidic deprotection of **26**, the resulting aldehyde **27** was oxidized using KMnO_4_ (potassium permanganate) to obtain the desired SiFA*lin* building block **28**.

Next, the SiFA*lin*-comprising symmetrically branching building block **29**, being the basis for the following peptide conjugation and thus peptide heterodimer synthesis, was prepared. The branching unit was synthesized on a solid support using the same standard protocols followed for peptide synthesis (Scheme 4). For this purpose, a rink amide resin MBHA LL was first reacted with Fmoc-Lys(Mtt)-OH and *N,N*-bis[3[3 -(Fmoc-amino)propyl]glycine. After the cleavage of the Mtt-PG under mildly acidic conditions, **28** was conjugated to the framework. After the deprotection and cleavage from the resin, **29** was isolated in overall yields of 43% (for analytical data, see Supplementary Materials Figures S16–S46).

**Scheme 5:**
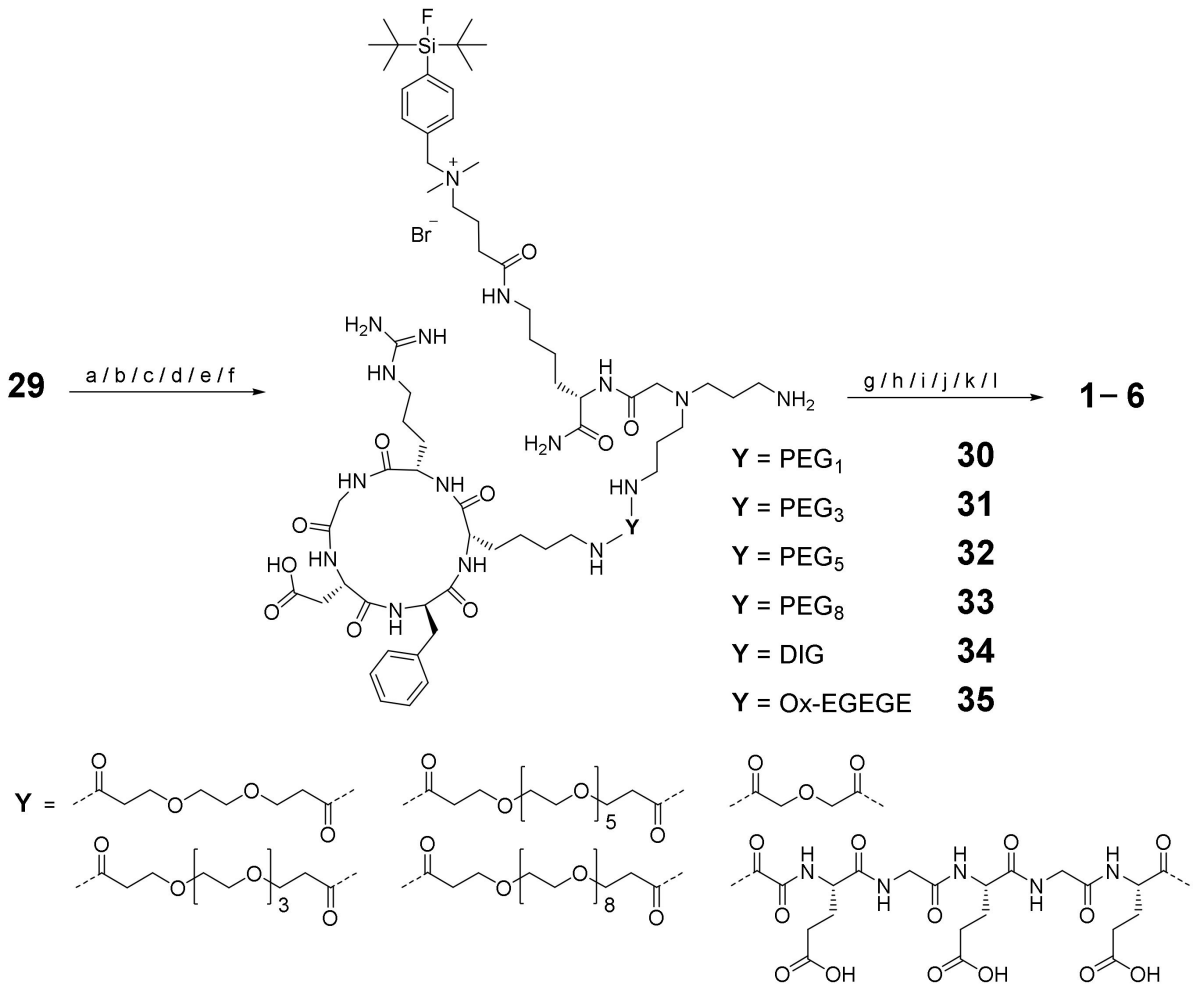
Schematic depiction of the synthesis of the MC1R- and integrin α_v_β_3_-affine HBPLs **1**–**6**. Conditions: (**a**) **10**, DIPEA, DMF, 60% yield for **30**; (**b**) **11**, DIPEA, DMF, 67% yield for **31**; (**c**) **12**, DIPEA, DMF, 41% yield for **32**; (**d**) **13**, DIPEA, DMF, 49% yield for **33**; (**e**) **14**, DIPEA, PyBOP, DMF, 46% yield for **34**; (**f**) **15**, DIPEA, PyBOP, DMF, 58% yield for **35**; (**g**) **16**, DIPEA, DMF, 77% yield for **1**; (**h**) **17**, DIPEA, DMF, 66% yield for **2**; (**i**) **18**, DIPEA, DMF, 49% yield for **3**; (**j**) **19**, DIPEA, DMF, 58% yield for **4**; (**k**) **20**, DIPEA, PyBOP, DMF, 24% yield for **5**; (**l**) **21**, DIPEA, PyBOP, DMF, 21% yield for **6**.

Finally, the synthesized building blocks **10**–**21** and **29** were assembled into the heterobivalent target agents **1**–**6**. For this purpose, **29** was first reacted with the c(RGDfK) derivatives **10**–**15**, which produced the monovalent intermediates **30**–**35**. These were further reacted with the GG-Nle-(DHfRWK) derivatives **16**–**21** into the final products **1**–**6** (Scheme 5), as this order gave better results (in terms of achievable isolated yields) than did first conjugating the structurally more demanding peptides **16**–**21** followed by the smaller ones (**10**–**15**).

The conjugation of the peptide–linker conjugates **10**–**13** and **16**–**19** was conducted analogously to the synthesis of the peptide–linker conjugates by directly reacting the starting materials in DMF using DIPEA as a base. For the conjugation of **14**, **15**, **20** and **21**, which were obtained as free acids instead of the respective NHS esters, the linker-modified peptides had to be activated before conjugation using PyBOP as the coupling agent.

The intermediates **30**–**35** were obtained in yields of 41–67% (for analytical data, see Supplementary Materials Figures S47–S52). The isolated yields of the final products **1**–**6** varied depending on the reaction pathway. Whereas during the conjugation reactions of the NHS-modified peptides **16**–**19** to **30**–**33** relatively high yields of 49–77% could be achieved, the yields during the reactions of **20** and **21** to **34** and **35** were considerably lower at 24% and 21%, respectively. This might be attributable to the additional activation step being required for the free acids **20** and **21** or the shorter linker structure, resulting in a steric hindrance of the conjugation reaction.

For the HBPLs **1**–**6**, ^19^F NMR spectra were recorded (for analytical data, see Supplementary Materials Figures S53–S64) along with standard HR mass spectrometry to verify that all agents contained the required fluorine atom in the SiFA*lin* building block, instead of having formed the hydrolyzed hydroxy-comprising species. All spectra showed a signal with a chemical shift between δ = −175–−177 ppm, which indicates the presence of an intact SiFA*lin*-moiety [49,50].

### 2.3. ^18^F-Radiolabeling of ***1***–***6*** and Determination of Lipophilicity and Stability of [^18^F]***1***–[^18^F]***6*** in Human Serum

In the following procedures, the HBPLs **1**–**6** were radiolabeled with [^18^F]fluoride as instructed by previously published protocols on other SiFA*lin*-modified peptides [35,51]. Briefly, [^18^F]fluoride was dried using the “Munich method” [52] over a QMA carbonate Sep-Pak SPE light cartridge, instead of applying an azeotropic drying, and the activity was eluted from the cartridge using a freshly prepared solution of K_222_ (Kryptofix222) and KOH (potassium hydroxide) in acetonitrile (MeCN). After optimizing the reaction and elution conditions, the pH of the obtained solution was adapted with oxalic acid, preventing potential basic hydrolysis of the SiFA*lin*-moiety. To this mixture, small amounts of the respective precursor molecules **1**–**6** at 25 nmol were added and incubated at ambient temperature for 10 min. Afterward, the radiolabeled products [^18^F]**1**–[^18^F]**6** were purified using a C18 Sep-Pak SPE light cartridge and eluted with EtOH/H_2_O (ethanol/water, 9/1, *v*/*v*). The ^18^F-labeled agents [^18^F]**1**–[^18^F]**6** were obtained in radiochemical yields (RCY) of 27–50%, radiochemical purities (RCP) of ≥95% and non-optimized molar activities (A_m_) of 17–51 GBq/μmol, starting from 0.8–2.2 GBq of [^18^F]fluoride (Table 1) within 25 min overall preparation time.

Since high lipophilicity of peptidic radiotracers can lead to a high plasma protein binding, resulting in unspecific organ and high liver uptakes, thus negatively impact tumor visualization [46,53,54], the lipophilicity of the HBPLs was determined to get an approximate estimation of the in vivo biodistribution behavior of the radioligands. Therefore, the log*_D_*_(7.4)_ values of the SiFA*lin*-modified HBPLs [^18^F]**1**–[^18^F]**6** were determined via their distribution coefficient between *n*-octanol and phosphate buffer at pH 7.4. The results are also summarized in Table 1. In these experiments, [^18^F]**4** (log*_D_*_(7.4)_ = −1.39 ± 0.03) exhibited the highest hydrophilicity of the PEG_n_-linker based HBPLs. Furthermore, it is clear that the introduction of negative charges led to the expected substantially increased hydrophilicity of [^18^F]**6** (log*_D_*_(7.4)_ = −1.52 ± 0.01). Overall, all ^18^F-labeled HBPLs demonstrated hydrophilicity suitable for further in vivo application.

**Figure 3:**
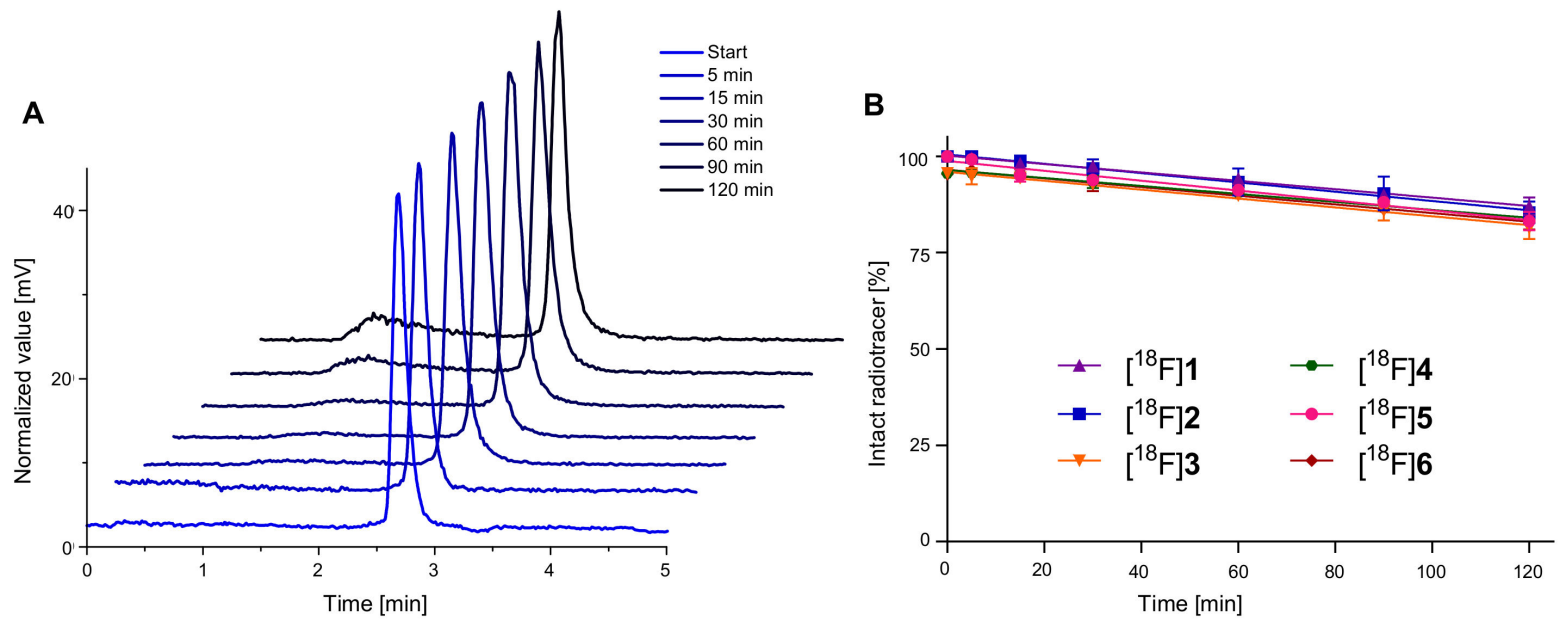
Depiction of the results of the in vitro serum stability evaluation experiments performed in human serum. (**A**) Radio-HPLC chromatograms for [^18^F]**2** at certain time points and (**B**) portions of intact radiotracer over time. Values are depicted as mean (*n* = 3); error bars represent SD.

In addition to the investigation of the radiotracers’ lipophilicity, their stability in human serum was evaluated in order to determine possible stability issues of the newly developed agents. For this purpose, the respective ^18^F-labeled HBPLs were incubated in human serum at 37 °C for 120 min. The results of these experiments are summarized in Table 1, and the corresponding radio-HPLC chromatograms for [^18^F]**2** as representative examples for all compounds studied are depicted in Figure 3A (see Supplementary Materials, Figure S91 for the results obtained for the other radioligands). In Figure 3B, the portions of intact radiotracer over the course of the stability experiments are depicted.

As the results indicated the radiolabeled HBPLs [^18^F]**1**–[^18^F]**6** to be sufficiently stable in vitro (81–87% intact radioligand after 120 min), all radiotracers were found to be suitable for in vivo imaging via PET/CT.

### 2.4. In Vitro Evaluation of ***1***–***6*** Regarding Their Binding Affinities to the Respective Target Receptors

**Figure 4:**
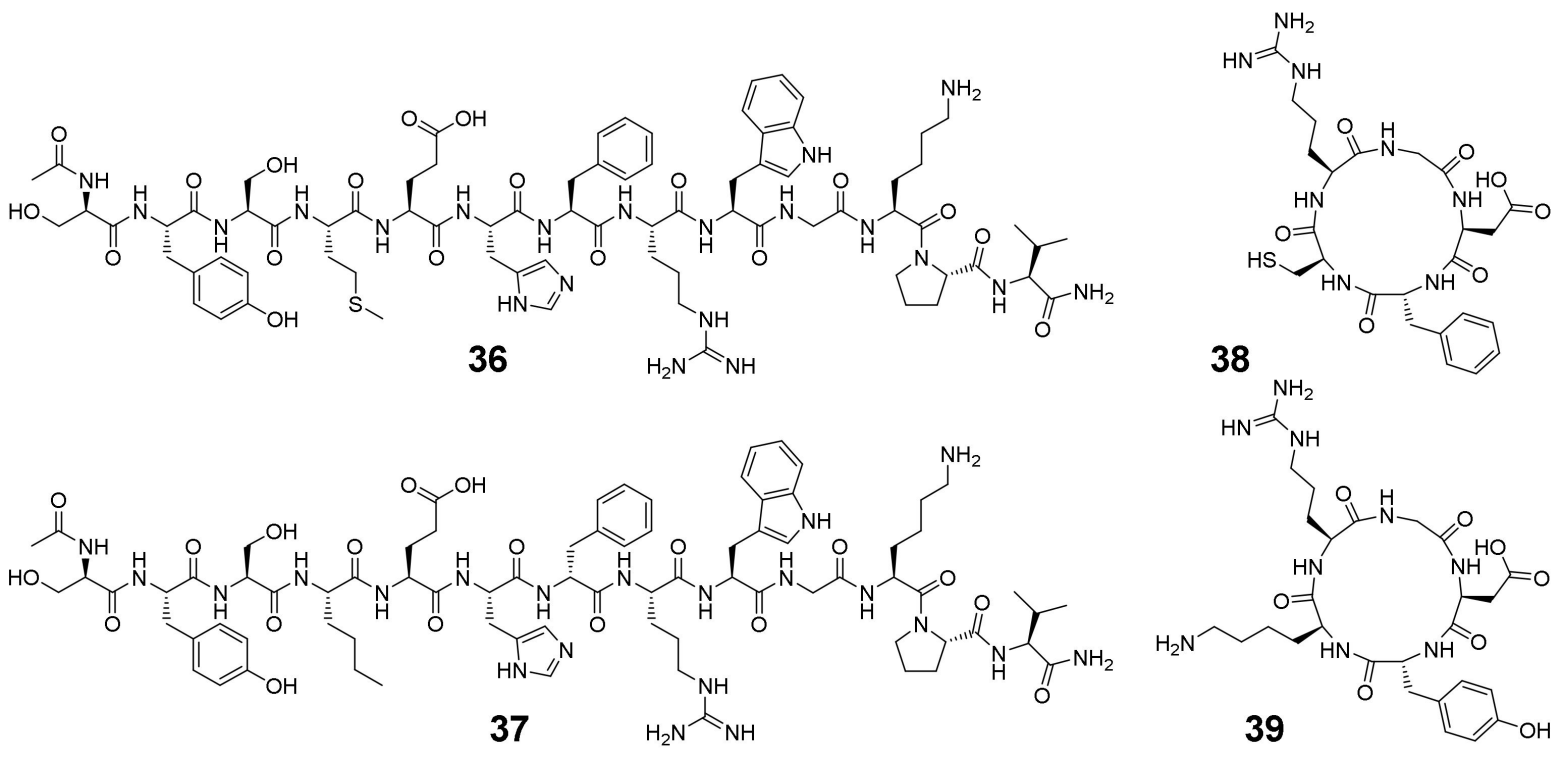
Structures of α-MSH (**36**), NDP (**37**), c(RGDfC) (**38**) and c(RGDyK) (**39**), which were used as monospecific reference peptides during the competitive displacement studies.

As in vitro receptor affinities represent an important parameter for the in vivo tumor uptake of radiotracers, the binding affinities of **1**–**6** were determined to both target receptors—integrin α_v_β_3_ and the MC1R—in competitive displacement assays. During these evaluations on MC1R-positive B16F10 cells [55] and integrin α_v_β_3_-positive U87MG cells [56], α-MSH (**36**), NDP (**37**), c(RGDfC) (**38**) and c(RGDyK) (**39**) (Figure 4) served as reference compounds, and [^125^I]I-echistatin and [^125^I]I-NDP were used as integrin α_v_β_3_-affine and MC1R-affine competitors, respectively. Peptides **36**–**39** were synthesized using the same Fmoc-based SPPS protocols used for the preparation of the other peptidic agents before.

**Figure 5:**
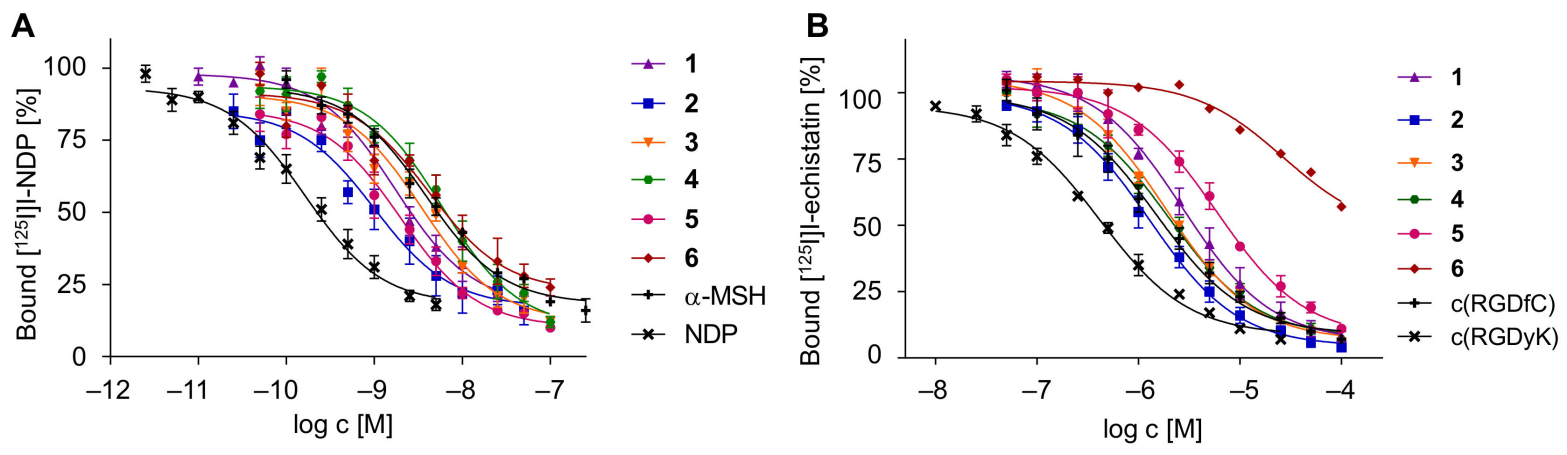
Depiction of the determined binding curves of the HBPLs **1**–**6**, obtained by competitive displacement assays on MC1R-positive B16F10 cells (**A**) and integrin α_v_β_3_-positive U87MG cells (**B**). Values are depicted as mean (*n* = 9); error bars represent SD.

The resulting binding curves and determined IC_50_ values are depicted in Figure 5 and summarized in Table 2.

To ensure that the binding of each peptide binder to its target receptor was unaffected by the other respective peptide of the HBPL, the monomeric peptides c(RGDfK) and GG-Nle-c(DHfRWK), which cannot bind to the receptors MC1R and integrin α_v_β_3_, respectively, were also examined under the same conditions, showing—as expected—no receptor-specific binding (see Supplementary Materials Figure S92 for details).

Considering the receptor affinity data with respect to the MC1R, none of the developed agents was as potent as NDP (IC_50_ of 0.17 ± 0.04 nM), which is however not surprising as NDP is a superpotent synthetic analog of the endogenous ligand α-MSH, thus exhibiting a high potency. However, **3** (IC_50_ of 3.44 ± 0.09 nM), **5** (IC_50_ of 2.05 ± 0.35 nM), **1** (IC_50_ of 1.74 ± 0.25 nM) and **2** (IC_50_ of 0.99 ± 0.11 nM) showed considerably higher affinities than the physiological reference α-MSH (IC_50_ of 3.75 ± 0.61 nM). In comparison, **4** (IC_50_ of 6.00 ± 0.47 nM) showed a decreased affinity and **6**, comprising the charged linker (IC_50_ of 4.18 ± 0.32 nM), exhibited a fourfold higher IC_50_ value compared to its uncharged counterpart, **2** (same distance between both peptide binders but differing linker composition), and thus considerably decreased affinity.

The corresponding experiments on the integrin α_v_β_3_-positive U87MG cells revealed that neither **5** (IC_50_ of 5895 ± 722 nM), **1** (IC_50_ of 2881 of 757 nM), **4** (IC_50_ of 2034 of 323 nM) nor **3** (IC_50_ of 1911 ± 70 nM) were as potent as the highly affine reference peptide c(RGDyK) (IC_50_ of 427 ± 37 nM; in accordance with former values obtained on these cells [57]), whereas at least compound **2**, showing an IC_50_ value of 1300 ± 288 nM, demonstrated a higher integrin affinity than the other reference c(RGDfC) (IC_50_ of 1493 ± 210 nM; also in accordance with literature data [44]). For HBPL **6**, an IC_50_ value towards α_v_β_3_ could not be determined in the same concentration range of the other agents studied but showed a substantially reduced affinity to the target receptor, compared to **1**–**5**. This observed negative influence of anionic charges on the resulting receptor affinities was also described in other studies [58] and could be confirmed here.

From the obtained results, it can be concluded that the introduction of a negatively charged linker impairs binding to the MC1R, as well as to integrin α_v_β_3_, and thus limits the usefulness of the approach. Within the line of the other linkers used, a similar trend can be observed on both cell lines with regard to the linker length used. In both cell lines and thus for both receptor types, the affinities increased with increasing linker length up to the PEG_3_-unit but then decreased with further increasing linker length, thus giving the best results for the PEG_3_-modified analog **2** on both receptor types.

### 2.5. Evaluation of the In Vivo Pharmacokinetics and Ex Vivo Biodistribution of [^18^F]***2*** and [^18^F]***4***

**Figure 6:**
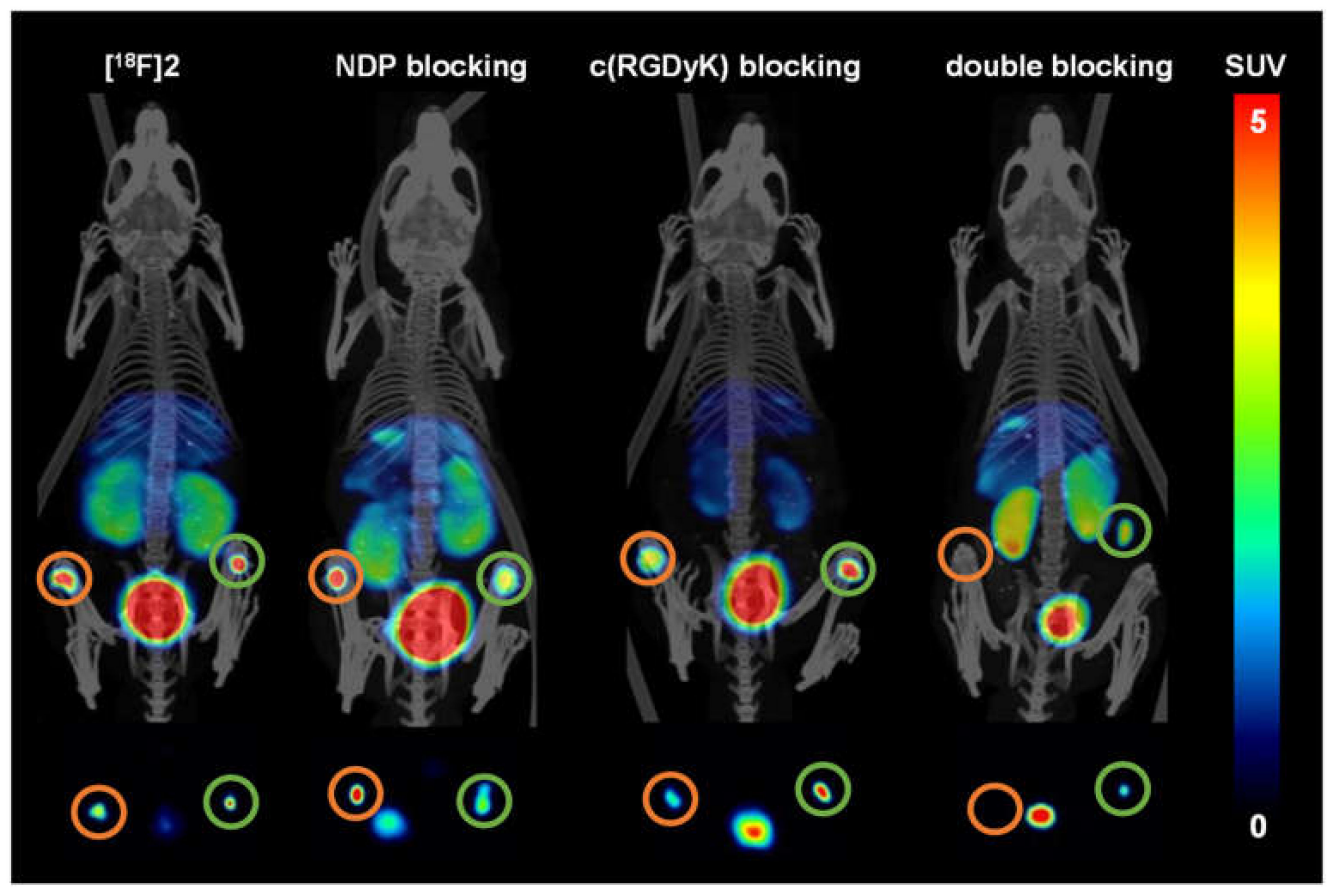
Depiction of the PET/CT images given as maximum intensity projections (MIPs) applying [^18^F]**2** as the radioligand (*n* = 3 for each group). From left to right: [^18^F]**2** without blocking; NDP blocking; c(RGDfK) blocking; blocking using both agents. Shown are MIPs obtained for 50–90 min PI (post-injection). Upper row: coronal slices; bottom row: transaxial slices at the tumor level. Circled in orange: U87MG tumors; circled in green: B16F10 tumors.

**Figure 7:**
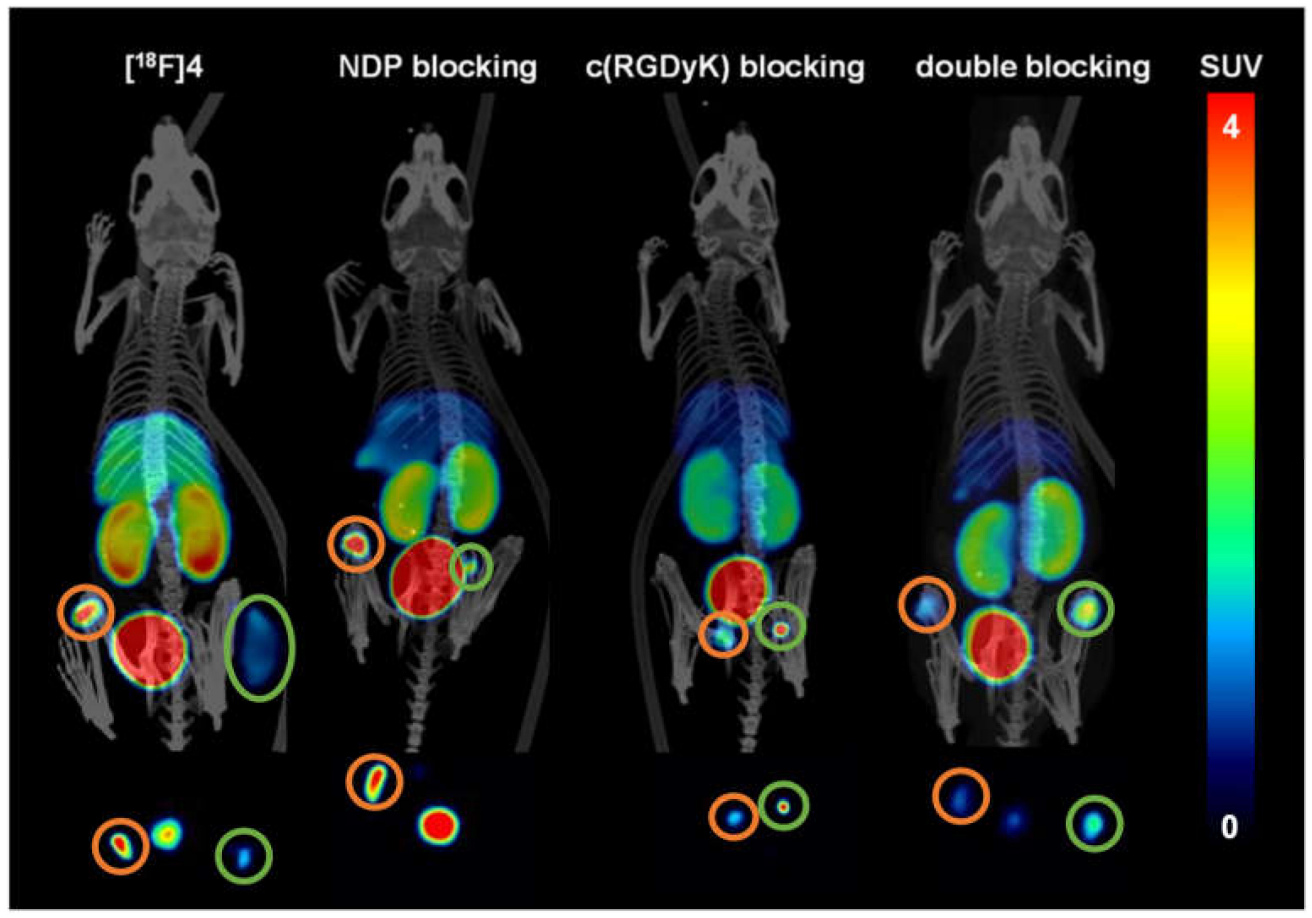
Depiction of the PET/CT images given as MIPs applying [^18^F]**4** as the radioligand (*n* = 3 for each group). From left to right: [^18^F]**4** without blocking; NDP blocking; c(RGDfK) blocking; blocking using both agents. Shown are MIPs obtained for 50–90 min PI (post-injection). Upper row: coronal slices; bottom row: transaxial slices at tumor level. Circled in orange: U87MG tumors; circled in green: B16F10 tumors. In the transaxial sections, it was not always possible to depict both tumors as some were out of plane.

**Figure 8:**
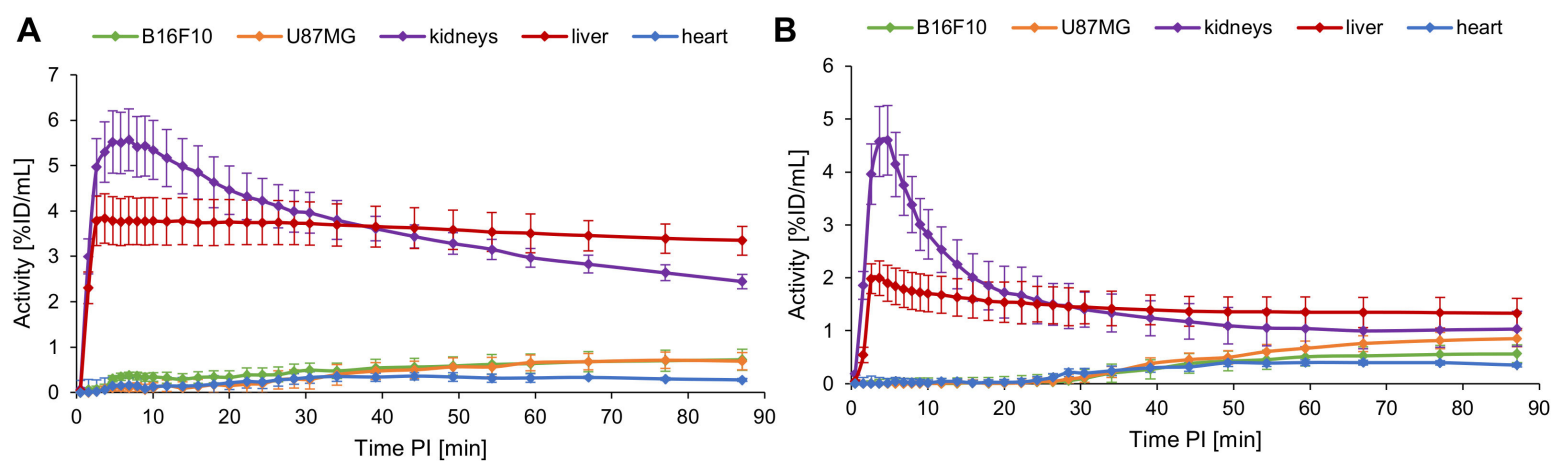
Depiction of the TACs for B16F10 and U87MG tumors, kidneys, liver and heart over 90 min PI for [^18^F]**2** (**A**) and [^18^F]**4** (**B**) (*n* = 3). Values are depicted as mean; error bars represent SD.

For the evaluation of the in vivo pharmacokinetics, the two most promising HBPLs—[^18^F]**2** with the highest affinity to both target receptors and [^18^F]**4** with the highest hydrophilicity and still reasonable binding affinities—were selected. For PET/CT imaging, 6-week-old male nude mice (Balb/cAnNRj-Foxn1^nu/nu^) were subcutaneously injected with 5 × 10^5^ B16F10 cells into the right flank and 2.0–2.5 × 10^6^ U87MG cells into the left flank to generate the respective receptor-positive tumors. When the tumors reached a sufficient size for imaging, each mouse was administered 4.15 ± 2.28 MBq of [^18^F]**2** or 3.95 ± 2.06 MBq of [^18^F]**4** via the lateral tail vein under isoflurane anesthesia. To determine the receptor specificity of both peptide parts of the labeled HBPLs and their relative contribution to overall tumor uptake, blocking experiments were also performed. For these, the respective radiotracer was coinjected with the corresponding blocking substance—20 μg NDP, 200 μg c(RGDyK) or both for double blocking—via the lateral tail vein. After i.v. injection of the tracers, a dynamic PET scan, followed by a CT scan, was performed. The resulting PET/CT images and time–activity curves (TACs) are depicted in Figure 6, Figure 7 and Figure 8. After completion of the diagnostic scans, the mice were sacrificed, their organs (blood, spleen, liver, kidney, pancreas, lung, heart, brain, bone, muscle, tail, tumors, stomach, colon and small intestine) were collected and measured in a γ-counter for ex vivo biodistribution (see Supplementary Materials Table S1 for detailed results).

From the PET/CT scans (Figure 6 and Figure 7) it was apparent that [^18^F]**2** and [^18^F]**4** both could clearly visualize the B16F10 as well as the U87MG tumors. [^18^F]**2** accumulated in the B16F10 tumor to a similar extent (2.32 ± 0.49% ID/g) as in the U87MG tumor (2.33 ± 0.46% ID/g). In contrast, [^18^F]**4** showed a higher accumulation in the U87MG tumor (3.92 ± 1.31% ID/g) than [^18^F]**2** (difference not significant (ns), *p* = 0.17), which is at first glance astonishing, as lower receptor affinities were found for [^18^F]**4** to both receptor types. Tumor uptakes of [^18^F]**4** in the B16F10 tumors were, however, comparable to those of [^18^F]**2** (2.58 ± 0.86% ID/g for [^18^F]**4** and 2.32 ± 0.49% ID/g for [^18^F]**2**).

From the PET/CT data depicted in Figure 7, the visual impression obtained is that [^18^F]**4** accumulates only to a low extent in B16F10 tumors. However, ex vivo biodistribution data confirm the data of the TACs and the uptake to be comparatively high as in the case of [^18^F]**2**. Moreover, during the blocking experiments with c(RGDyK), not affecting the uptake of [^18^F]**4** in B16F10 tumors, the tumor is clearly visible. Thus, the visually lower tumor uptake of [^18^F]**4** in the B16F10 tumor, depicted in Figure 7, should be due to the fact that the large tumor already had partially necrotic areas, showing no tracer uptake anymore. This assumption is supported by the literature [59,60].

In the blocking experiments, the receptor specificity of the tracers could be demonstrated, as blocking with NDP and c(RGDfK) resulted in a considerable decrease of the respective tumor uptakes of both tracers in B16F10 and U87MG tumors. Coinjection with NDP substantially reduced the accumulation in the MC1R-positive B16F10 tumors ([^18^F]**2**: reduction from 2.32 ± 0.49% to 1.83 ± 0.24% ID/g (change ns, *p* = 0.19) and [^18^F]**4**: reduction from 2.58 ± 0.86% to 1.33 ± 0.27% ID/g (change ns, *p* = 0.07)). Corresponding results were also found for c(RGDyK) blocking, where U87MG tumor uptakes were reduced from 2.33 ± 0.46% to 1.48 ± 0.12% ID/g for [^18^F]**2** (change significant, *p* = 0.04) and from 3.92 ± 1.31% to 2.67 ± 0.66% ID/g for [^18^F]**4** (change ns, *p* = 0.21). Despite these mostly insignificant changes observed for tumor uptakes of both tracers by blocking, the trends are nonetheless clearly visible, confirming that both radiolabeled HBPLs bind specifically to both target receptors, and each peptide part equally contributed to tumor visualization.

The TACs of [^18^F]**2** and [^18^F]**4** show a comparable uptake pattern of both tracers over time. The uptakes in kidneys and liver reached their maximum after 5–10 min, whereas the curves of both tumors approached a plateau only after about 70 min. In the direct comparison of both tracers, [^18^F]**4** showed a delayed accumulation in the tumors compared to [^18^F]**2,** for which the reason is not obvious. [^18^F]**4** furthermore showed a considerably lower uptake into kidneys and liver, resulting in higher tumor-to-organ ratios for [^18^F]**4**. Additionally, for the blood and muscle, a lower unspecific uptake of [^18^F]**4** was found compared to [^18^F]**2**, thus resulting in overall much more favorable and mostly significantly higher tumor-to-organ ratios of [^18^F]**4** (see Supplementary Materials Table S2 for details).

In summary, both radiotracers developed were able to clearly visualize both integrin α_v_β_3_-positive and MC1R-positive tumors, and both parts of the heterobivalent agents contributed to receptor-specific tumor uptakes (see Supplementary Materials Figure S93). However, [^18^F]**4** demonstrated lower non-target organ uptakes and faster clearance than [^18^F]**2**, thus resulting in considerably higher tumor-to-background ratios, despite its lower in vitro receptor binding affinities to both target receptor types.

Therefore, [^18^F]**4** proved to be the more promising radiotracer for the bispecific imaging of malignant melanoma by PET/CT, having a high potential for clinical translation.

## 3. Materials and Methods

### 3.1. General

#### 3.1.1. Chemistry

All reagents and solvents for synthesis were at least of analytical grade and were used without further purification. Dried solvents were obtained from Sigma-Aldrich (Taufkirchen, Germany) and were stored under inert gas. Fmoc-protected amino acids and resins were purchased from Novabiochem (Darmstadt, Germany). Water in HPLC grade and spectroscopic trifluoroacetic acid (TFA) were obtained from Carl Roth (Karlsruhe, Germany), and acetonitrile (MeCN) in HPLC grade from Häberle LABORTECHNIK (Lonsee-Ettlenschieß, Germany). Moreover, 2-(bis(3-(((9*H*-fluoren-9-yl)methoxy)carbonylamino)-propyl)amino)-acetic acid potassium hemisulfate ((Fmoc-NH-propyl)_2_Gly-OHxKHSO_4_) and the bis-*N*-hydroxy-succinimide (NHS)-esters NHS-PEG_1_-NHS, NHS-PEG_3_-NHS and NHS-PEG_8_-NHS were purchased from Iris Biotech (Marktredwitz, Germany) and NHS-PEG_5_-NHS from BroadPharm (San Diego, CA, USA). Other chemicals and solvents were obtained from commercial suppliers (Sigma-Aldrich (Taufkirchen, Germany), Merck (Darmstadt, Germany), TCI (Eschborn, Germany), abcr (Karlsruhe, Germany), Carl Roth (Karlsruhe, Germany) and Alfa Aesar (Schwerte, Germany)). The synthesis of c(RGDfK) was carried out according to published procedures [44]. Thin-layer chromatography (TLC) was performed on silica gel 60 F_254_ plates (MACHEREY-NAGEL; Düren, Germany) and visualized by exposure to ultraviolet (UV) light at 254 nm. Column chromatography was performed on silica gel 60 (0.04–0.063 mm) (Carl Roth; Karlsruhe, Germany). NMR (nuclear magnetic resonance) spectra were recorded on a Varian NMR System Spectrometer (500 MHz for ^1^H and 126 MHz for ^13^C) and a Varian Mercury Plus Spectrometer (282 MHz for ^19^F) at room temperature. The chemical shifts (δ) of ^1^H- and ^13^C-spectra were internally referenced to residual solvent signals and are expressed in parts per million (ppm). For ^19^F-spectra, trifluoroacetic acid was used as external reference (δ = −76.55 ppm). All coupling constants (*J*) are reported in Hertz (Hz), and the following notations indicate the multiplicity of the signals: s (singlet), d (doublet), t (triplet) and m (multiplet). MS (mass spectrometry) and HR (high-resolution) MS measurements were performed on a Bruker Daltonics Microflex MALDI-TOF (MALDI: matrix-assisted laser desorption/ionization), Jeol AccuTOF GCx FI/FD (FI: field ionization; FD: field desorption), Bruker ApexQe DART/ESI Instrument (DART: direct analysis in real-time; ESI: electrospray ionization) and Finnigan MAT95Q HR-ESI spectrometers. For HPLC (high-performance liquid chromatography), a Dionex UltiMate 3000 system from Thermo Fisher Scientific was used together with Chromeleon software (v7.11). For analytical chromatography and semipreparative analyses, Chromolith Performance (RP-18e, 100–4.6 mm, Merck; Darmstadt, Germany) and Chromolith (RP-18e, 100–10 mm, Merck; Darmstadt, Germany) columns were used, respectively. All operations were performed with a flow rate of 4 mL/min using H_2_O + 0.1% TFA and MeCN + 0.1% TFA as solvents.

#### 3.1.2. Radiolabeling

Tracepur H_2_O and Kryptofix222 (K_222_) were purchased from Merck. Anhydrous acetonitrile (MeCN), dimethyl sulfoxide (DMSO), oxalic acid and *n*-octanol were obtained from Sigma-Aldrich; ethanol (EtOH), sodium dihydrogen phosphate (NaH_2_PO_4_) and disodium hydrogen phosphate (Na_2_HPO_4_) were obtained from Carl Roth; 0.9% sodium chloride (NaCl)-solution was obtained from VWR (Bruchsal, Germany). Aqueous [^18^F]fluoride solution was purchased from EuroPET in Freiburg or the University Hospital Tübingen. The Sep-Pak Accell Plus QMA Carbonate (46 mg) and Sep-Pak C18 Plus SPE Light cartridges (130 mg) were obtained from Waters (Eschborn, Germany). For radioanalytical use, a Dionex UltiMate 3000 system equipped with a Raytest Gabi Star radioactivity detector was used together with a Chromolith Performance (RP-18e, 100–4.6 mm, Merck, Germany) column. All operations were performed with a flow rate of 4 mL/min using H_2_O + 0.1% TFA and MeCN + 0.1% TFA as solvents. Radioactivity was measured by an ISOMED 2010 activimeter.

#### 3.1.3. Competitive Binding Studies

Murine melanoma cells (B16F10) and human glioblastoma cells (U87MG) as well as Dulbecco’s Modified Eagle’s Medium (DMEM) and Eagle’s Minimum Essential Medium (EMEM) were purchased from ATCC (Wesel, Germany). Fetal calf serum (FCS) was obtained from Bio&SELL (Feucht, Germany); phosphate-buffered saline (PBS), 1,10-phenanthroline, *tris*(hydroxymethyl)aminomethane hydrochloride (Tris·HCl) and manganese chloride (MnCl_2_) were obtained from Sigma-Aldrich (Taufkirchen, Germany); penicillin/streptomycin (pen/strep) and 0.25% Trypsin with 0.02% EDTA-solution in PBS were obtained from Gibco (Schwerte, Germany); 2-(4-(2-hydroxyethyl)-1-piperazinyl)-ethanesulfonic acid (HEPES) was obtained from Gerbu (Heidelberg, Germany); bovine serum albumin (BSA), sodium chloride (NaCl), calcium chloride (CaCl_2_) and magnesium chloride (MgCl_2_) were obtained from Carl Roth (Karlsruhe, Germany). [^125^I]I-NDP (NEX352, 81.4 GBq/μmol) and [^125^I]I-echistatin (NEX083, 81.4 GBq/μmol) were purchased from PerkinElmer (Rodgau, Germany). γ-counting was performed using a 2480 Wizard^2^ gamma counter system from PerkinElmer.

#### 3.1.4. In Vivo PET Imaging

Briefly, 4–5-week-old male nude mice (Balb/cAnNRj-Foxn1^nu/nu^) were obtained from Janvier. A dynamic PET scan over 90 min and a subsequent CT image over 20 min were acquired using a triple-modality Bruker Albira II small-animal PET/CT/SPECT scanner. Three animals were studied per group.

#### 3.1.5. Statistical Analyses

For statistical analyses, unpaired, parametric two-tailed *t*-tests were performed. Statistical significance is indicated as * (*p* < 0.05), ** (*p* < 0.01) or *** (*p* < 0.001).

### 3.2. Chemical Syntheses

#### 3.2.1. Synthesis of Peptides **7**–**9**

General procedure for peptide synthesis (GP1): Peptides were synthesized on a solid support according to standard Fmoc-based solid-phase peptide synthesis (SPPS) protocols. The resin was first swollen in CH_2_Cl_2_ for 60 min and then rinsed with DMF. After deprotection of the Fmoc-protecting group (PG) with piperidine/DMF (1/1, *v*/*v*) for 2 + 5 min, the respective amino acid (4.0 equiv.) was pre-activated with DIPEA (4.0 equiv.) and HBTU (3.9 equiv.) in DMF for 2 min and then coupled for 60 min. These steps were repeated until the respective peptide sequence was complete.

GG-Nle-c(DHfRWK) (**7**): According to GP1, the peptide sequence GG-Nle-DHfRWK was synthesized on rink amide resin AM LL (73.5 mg, 25 μmol, 1.0 equiv., 0.34 mmol/g) and then the Mtt- and *O*-2-Ph*^i^*Pr-PG were removed with TFA/CH_2_Cl_2_ (1/99, *v*/*v*) for 90 min. Subsequently, the cyclization was conducted using DIPEA (17 μL, 100 μmol, 4.0 equiv.) and PyBOP (50.7 mg, 97.5 μmol, 3.9 equiv.) in DMF for 20 h. After Fmoc deprotection, the cyclized peptide was cleaved from the resin and deprotected with TFA/TIS/H_2_O (95/2.5/2.5, *v*/*v*/*v*) for 3 h. Briefly, **7** was purified by HPLC (semipreparative, 0–30% MeCN + 0.1% TFA in 6 min, t_R_ = 5.71 min) and isolated as a colorless solid (11.5 mg, 10.5 μmol, yield: 42%, purity: >99%). MS (MALDI) m/z calculated for C_52_H_74_N_17_O_10_ [M + H]^+^: 1096.58, found: 1096.01; HR-ESI-MS m/z calculated for C_52_H_75_N_17_O_10_ [M + 2H]^2+^: 548.7936, found: 548.7928; HR-ESI-MS m/z calculated for C_52_H_74_N_17_O_10_ [M + H]^+^: 1096.5799, found: 1096.5779.

c(RGDfK)-EGEGE (**8**): According to GP1, the peptide sequence DGRKf was synthesized on 2-Chlorotrityl chloride resin (164 mg, 200 μmol, 1.0 equiv., 1.22 mmol/g), then the Alloc-PG was removed with PhSiH_3_ (590 μL, 519 mg, 4.8 mmol, 24.0 equiv.) and Pd(PPh_3_)_4_ (28.9 mg, 25 μmol, 0.25 equiv.) in CH_2_Cl_2_ for 3 × 30 min. After deprotection of the Fmoc-PG with piperidine/DMF (1/1, *v*/*v*) for 2 × 10 min, the peptide was cyclized using DIPEA (136 μL, 800 μmol, 4.0 equiv.) and PyBOP (406 mg, 780 μmol, 3.9 equiv.) in DMF for 12 h. Subsequently, the ivDde-PG of lysine was deprotected using hydrazine/DMF (2/98, *v*/*v*) for 2 × 10 min and the remaining amino acids of the sequence EGEGE sequence were coupled. The cyclized peptide was cleaved and deprotected with TFA/TIS/H_2_O (95/2.5/2.5, *v*/*v*/*v*) for 3 h. Peptide **8** was precipitated in cold Et_2_O, purified by HPLC (semipreparative, 0–40% MeCN + 0.1% TFA in 8 min, t_R_ = 3.88 min) and isolated as a colorless solid (90.4 mg, 82 mmol, yield: 41%, purity: >99%). MS (MALDI) m/z calculated for C_46_H_69_N_14_O_18_ [M + H]^+^: 1105.49, found 1105.39; HR-ESI-MS m/z calculated for C_46_H_70_N_14_O_18_ [M + 2H]^2+^: 553.2491, found: 553.2486; HR-ESI-MS m/z calculated for C_46_H_69_N_14_O_18_ [M + H]^+^: 1105.4909, found: 1105.4905.

EGEGE-GG-Nle-c(DHfRWK) (**9**): According to GP1, the peptide sequence KWRfHD was synthesized on rink amide resin AM LL (347 mg, 125 μmol, 1.0 equiv., 0.36 mmol/g), then the Mtt- and O-2-Ph^i^Pr-PG were removed with TFA/CH_2_Cl_2_ (1/99, *v*/*v*) for 90 min. Subsequently, the cyclization was conducted using DIPEA (85 μL, 500 μmol, 4.0 equiv.) and PyBOP (254 mg, 488 μmol, 3.9 equiv.) in DMF for 15 h. After Fmoc-deprotection for 2 × 10 min, the remaining amino acids of the EGEGE–GG–Nle sequence were coupled. The cyclized peptide was cleaved and deprotected with TFA/TIS/H_2_O (95/2.5/2.5, *v*/*v*/*v*) for 3 h. Peptide **9** was precipitated in cold Et_2_O, purified by HPLC (semipreparative, 0–30% MeCN + 0.1% TFA in 8 min, t_R_ = 7.18 min) and isolated as a colorless solid (85.2 mg, 53.3 μmol, yield: 43%, purity: >88%). MS (MALDI) *m*/*z* calculated for C_71_H_101_N_22_O_21_ [M + H]^+^: 1597.75, found: 1597.30; HR-ESI-MS *m*/*z* calculated for C_71_H_102_N_22_O_21_ [M + 2H]^2+^: 799.3789, found: 799.3790; HR-ESI-MS *m*/*z* calculated for C_71_H_102_N_22_O_21_ [M + 2H]^+^: 1598.7590, found: 1598.7538.

#### 3.2.2. Modification of the Peptides with Linker Structures to Obtain **10**–**21**

General procedure for the synthesis of NHS-PEG_n_ peptides (GP2): All steps were carried out under an N_2_ atmosphere. A total of 1.0 equiv. of the respective peptide was added to a solution of 1.0 equiv. *bis*-NHS-ester and 0.5–1.0 equiv. DIPEA in dry DMF. Subsequently, the reaction mixture was stirred for 5–40 min at room temperature, while reaction control was performed by HPLC (analytical, 0–50% MeCN + 0.1% TFA in 5 min). After the removal of the solvent under reduced pressure, the corresponding NHS-PEG_n_-peptide was obtained after purification via semipreparative HPLC. In addition to the respective target product, small amounts of the hydrolyzed compound HO-PEG_n_-peptide and the dimer peptide-PEG_n_-peptide were isolated (for analytical data, see Supplementary Materials Figures S65–S80).

NHS-PEG_1_-c(RGDfK) (**10**): According to GP2, NHS-PEG_1_-NHS (3.3 mg, 8.3 μmol, 1.0 equiv.), DIPEA (1.4 μL, 1.1 mg, 8.3 μmol, 1.0 equiv.) and c(RGDfK) (5.0 mg, 8.3 μmol, 1.0 equiv.) were reacted in 5 mL dry DMF for 20 min. After purification by HPLC (semipreparative, 0–30% MeCN + 0.1% TFA in 10 min, t_R_ = 7.01 min), **10** was isolated as a colorless solid (3.0 mg, 3.4 μmol, yield: 41%, purity: >93%). MS (MALDI) *m*/*z* calculated for C_39_H_56_N_10_O_14_ [M]^+^: 888.40, found: 888.26; HR-ESI-MS *m*/*z* calculated for C_39_H_57_N_10_O_14_ [M + H]^+^: 889.4050, found: 889.4038.

NHS-PEG_3_-c(RGDfK) (**11**): According to GP2, NHS-PEG_3_-NHS (12.1 mg, 24.8 μmol, 1.0 equiv.), DIPEA (4.2 μL, 3.2 mg, 24.8 μmol, 1.0 equiv.) and c(RGDfK) (15.0 mg, 24.8 μmol, 1.0 equiv.) were reacted in 8 mL dry DMF for 15 min. After purification by HPLC (semipreparative, 0–30% MeCN + 0.1% TFA in 10 min, t_R_ = 4.84 min), **11** was isolated as a colorless solid (7.0 mg, 7.2 μmol, yield: 29%, purity: >89%). MS (MALDI) *m*/*z* calculated for C_43_H_64_N_10_O_16_ [M]^+^: 976.45, found: 976.68; HR-ESI-MS *m*/*z* calculated for C_43_H_65_N_10_O_16_ [M + H]^+^: 977.4575, found: 977.4567.

NHS-PEG_5_-c(RGDfK) (**12**): According to GP2, NHS-PEG_5_-NHS (9.6 mg, 16.6 μmol, 1.0 equiv.), DIPEA (2.8 μL, 2.2 mg, 16.6 μmol, 1.0 equiv.) and c(RGDfK) (10.0 mg, 16.6 μmol, 1.0 equiv.) were reacted in 6 mL dry DMF for 30 min. After purification by HPLC (semipreparative, 15–30% MeCN + 0.1% TFA in 10 min, t_R_ = 4.44 min), **12** was isolated as a colorless solid (6.1 mg, 5.7 μmol, yield: 35%, purity: >91%). MS (MALDI) *m*/*z* calculated for C_47_H_73_N_10_O_18_ [M + H]^+^: 1065.51, found: 1065.22; HR-ESI-MS *m*/*z* calculated for C_47_H_73_N_10_O_8_ [M + H]^+^: 1065.5099, found: 1065.5099.

NHS-PEG_8_-c(RGDfK) (**13**): According to GP2, NHS-PEG_8_-NHS (13.5 mg, 19.1 μmol, 1.0 equiv.), DIPEA (3.3 μL, 2.5 mg, 19.1 μmol, 1.0 equiv.) and c(RGDfK) (11.5 mg, 19.1 μmol, 1.0 equiv.) were reacted in 6 mL dry DMF for 40 min. After purification by HPLC (semipreparative, 0–30% MeCN + 0.1% TFA in 10 min, t_R_ = 7.01 min), **13** was isolated as a colorless solid (7.3 mg, 6.1 μmol, yield: 32%, purity: >82%). MS (MALDI) *m*/*z* calculated for C_53_H_85_N_10_O_21_ [M + H]^+^: 1197.59, found: 1197.34; HR-ESI-MS *m*/*z* calculated for C_53_H_84_N_10_O_21_ [M + H]^+^: 1197.5885, found: 1197.5892.

NHS-PEG_1_-GG-Nle-c(DHfRWK) (**16**): According to GP2, NHS-PEG_1_-NHS (5.4 mg, 6.1 μmol, 1.0 equiv.), DIPEA (0.5 μL, 0.4 mg, 3.1 μmol, 0.5 equiv.) and **7** (6.7 mg, 6.1 μmol, 1.0 equiv.) were reacted in 5 mL dry DMF for 5 min. After purification by HPLC (semipreparative, 15–50% MeCN + 0.1% TFA in 10 min, t_R_ = 4.80 min), **16** was isolated as a colorless solid (3.6 mg, 2.6 μmol, yield: 43%, purity: >93%). MS (MALDI) *m*/*z* calculated for C_64_H_89_N_18_O_17_ [M + H]^+^: 1381.66, found: 1381.34; HR-ESI-MS *m*/*z* calculated for C_64_H_89_N_18_O_17_ [M + H]^+^: 1381.6648, found: 1381.6643.

NHS-PEG_3_-GG-Nle-c(DHfRWK) (**17**): According to GP2, NHS-PEG_3_-NHS (4.7 mg, 9.6 μmol, 1.0 equiv.), DIPEA (1.6 μL, 1.2 mg, 9.6 μmol, 1.0 equiv.) and **7** (10.5 mg, 9.6 μmol, 1.0 equiv.) were reacted in 6 mL dry DMF for 40 min. After purification by HPLC (semipreparative, 10–30% MeCN + 0.1% TFA in 10 min, t_R_ = 8.51 min), **17** was isolated as a colorless solid (6.9 mg, 4.7 μmol, yield: 49%, purity: >91%). MS (MALDI) m/z calculated for C_68_H_97_N_18_O_19_ [M + H]^+^: 1469.72, found: 1469.47; HR-ESI-MS *m*/*z* calculated for C_68_H_98_N_18_O_19_ [M + 2H]^2+^: 735.3622, found: 735.3619; HR-ESI-MS *m*/*z* calculated for C_68_H_97_N_18_O_19_ [M + H]^+^: 1469.7172, found: 1469.7154.

NHS-PEG_5_-GG-Nle-c(DHfRWK) (**18**): According to GP2, NHS-PEG_5_-NHS (5.3 mg, 9.1 μmol, 1.0 equiv.), DIPEA (1.6 μL, 1.2 mg, 9.1 μmol, 1.0 equiv.) and **7** (10.0 mg, 9.1 μmol, 1.0 equiv.) were reacted in 5 mL dry DMF for 30 min. After purification by HPLC (semipreparative, 10–50% MeCN + 0.1% TFA in 8 min, t_R_ = 5.86 min), **18** was isolated as a colorless solid (5.9 mg, 3.8 μmol, yield: 42%, purity: >90%). MS (MALDI) *m*/*z* calculated for C_72_H_104_N_18_O_21_ [M]^+^: 1556.73, found: 1556.24; HR-ESI-MS *m*/*z* calculated for C_72_H_106_N_18_O_21_ [M + 2H]^2+^: 779.3884, found: 779.3884.

NHS-PEG_8_-GG-Nle-c(DHfRWK) (**19**): According to GP2, NHS-PEG_8_-NHS (3.3 mg, 4.7 μmol, 1.0 equiv.), DIPEA (0.8 μL, 0.6 mg, 4.7 μmol, 1.0 equiv.) and **7** (5.1 mg, 4.7 μmol, 1.0 equiv.) were reacted in 3 mL dry DMF for 40 min. After purification by HPLC (semipreparative, 0–50% MeCN + 0.1% TFA in 8 min, t_R_ = 6.09 min), **19** was isolated as a colorless solid (3.3 mg, 2.0 μmol, yield: 42%, purity: >90%). MS (MALDI) *m*/*z* calculated for C_78_H_117_N_18_O_24_ [M + H]^+^: 1689.85, found: 1689.09; HR-ESI-MS *m*/*z* calculated for C_78_H_118_N_18_O_24_ [M + 2H]^2+^: 845.4278, found: 845.4275.

General procedure for the synthesis of HO-DIG- and HO-Ox-EGEGE peptides (GP3): All steps were carried out under an N_2_ atmosphere. In total, 1.0–10.0 equiv. of the respective peptide was added to a solution of 1.0–10.0 equiv. *bis*-NHS-ester and 1.0 equiv. DIPEA in dry DMF. Subsequently, the reaction mixture was stirred for 5–50 min at room temperature, while the reaction control was performed by HPLC (analytical, 0–50% MeCN + 0.1% TFA in 5 min). After the removal of the solvent under reduced pressure, the corresponding HO-DIG- or HO-Ox-EGEGE peptides were obtained after semipreparative HPLC purification. In addition to the target products, small amounts of the dimers peptide-DIG/Ox-EGEGE-peptide were isolated (for analytical data, see Supplementary Materials Figures S81–S84).

HO-DIG-c(RGDfK) (**14**): According to GP3, NHS-DIG-NHS (8.2 mg, 24.9 μmol, 1.5 equiv.), c(RGDfK) (10.0 mg, 16.6 μmol, 1.0 equiv.) and DIPEA (2.8 μL, 2.1 mg, 16.6 μmol, 1.0 equiv.) were reacted in 11 mL dry DMF for 50 min. After purification by HPLC (semipreparative, 5–40% MeCN + 0.1% TFA in 10 min, t_R_ = 3.80 min), **14** was isolated as a colorless solid (4.4 mg, 6.1 μmol, yield: 37%, purity: >99%). MS (MALDI) *m*/*z* calculated for C_31_H_45_N_9_O_11_ [M]^+^: 719.32, found: 719.59; HR-ESI-MS *m*/*z* calculated for C_31_H_46_N_9_O_11_ [M + H]^+^: 720.3311, found: 720.3309.

HO-Ox-EGEGE-c(RGDfK) (**15**): According to GP3, NHS-Ox-NHS (17.9 mg, 63 μmol, 5.0 equiv.), **8** (13.9 mg, 12.6 μmol, 1.0 equiv.) and DIPEA (10.7 μL, 8.1 mg, 63 μmol, 5.0 equiv.) were reacted in 8 mL dry DMF for 5 min. After purification by HPLC (semipreparative, 5–20% MeCN + 0.1% TFA in 8 min, t_R_ = 4.29 min), **15** was isolated as a colorless solid (5.9 mg, 5.0 μmol, yield: 40%, purity: >99%). MS (MALDI) *m*/*z* calculated for C_48_H_69_N_14_O_21_ [M + H]^+^: 1177.48, found: 1177.02; HR-ESI-MS *m*/*z* calculated for C_48_H_68_N_14_O_21_ [M]^+^: 1176.4683, found: 1176.4913.

HO-DIG-GG-Nle-c(DHfRWK) (**20**): According to GP3, NHS-DIG-NHS (1.5 mg, 3.6 μmol, 1.0 equiv.), **7** (5.0 mg, 3.6 μmol, 1.0 equiv.) and DIPEA (0.6 μL, 0.5 mg, 3.6 μmol, 1.0 equiv.) were reacted in 2 mL dry DMF for 25 min. After purification by HPLC (semipreparative, 10–40% MeCN + 0.1% TFA in 10 min, t_R_ = 5.36 min), **20** was isolated as a colorless solid (2.7 mg, 2.2 μmol, yield: 61%, purity: >99%). MS (MALDI) *m*/*z* calculated for C_56_H_78_N_17_O_14_ [M + H]^+^: 1212.59, found: 1212.16; HR-ESI-MS *m*/*z* calculated for C_56_H_79_N_17_O_14_ [M + 2H]^2+^: 606.7991, found: 606.7991.

HO-Ox-EGEGE-GG-Nle-c(DHfRWK) (**21**): According to GP3, NHS-Ox-NHS (17.8 mg, 62.6 μmol, 10.0 equiv.), **9** (10.0 mg, 6.3 μmol, 1.0 equiv.) and DIPEA (10.7 μL, 8.1 mg, 62.6 μmol, 10.0 equiv.) were reacted in 5 mL dry DMF for 10 min. After purification by HPLC (semipreparative, 0–40% MeCN + 0.1% TFA in 8 min, t_R_ = 6.41 min), **21** was isolated as a colorless solid (4.5 mg, 2.7 μmol, yield: 44%, purity: >99%). MS (MALDI) *m*/*z* calculated for C_73_H_100_N_22_O_24_ [M]^+^: 1668.73, found: 1668.90; HR-ESI-MS *m*/*z* calculated for C_73_H_102_N_22_O_24_ [M + 2H]^2+^: 835.3713, found: 835.3716.

#### 3.2.3. Synthesis of SiFA*lin*-Carboxylic Acid **28** and SiFA*lin*-Modified Symmetrically Branching Framework **29**

((4-Bromobenzyl)oxy)(*tert*-butyl)dimethylsilane (**22**): All steps were carried out under an N_2_ atmosphere. TBDMS-Cl (5.81 g, 38.6 mmol, 1.2 equiv.) was added under ice-cooling to a solution of 4-bromobenzyl alcohol (6.01 g, 32.1 mmol, 1.0 equiv.) and imidazole (5.47 g, 80.3 mmol, 2.5 equiv.) in 36 mL dry DMF. After stirring for 20 h at room temperature, the reaction mixture was extracted with Et_2_O. The combined organic layers were washed with H_2_O, dried over Na_2_SO_4_, concentrated under reduced pressure and the crude product was purified by column chromatography (*n*-hexane/EtOAc 50/1 → 10/1) to give **22** (9.47 g, 31.4 mmol, 98%) as a colorless liquid. ^1^H NMR (500 MHz, CDCl_3_) δ 0.10 (s, 6H, SiCH_3_), 0.94 (s, 9H, CH_3_), 4.68 (s, 2H, H-5), 7.19 (s, *J* = 8.2 Hz, 2H, H-3), 7.45 (d, *J* = 8.2 Hz, 2H, H-2) ppm; ^13^C NMR (126 MHz, CDCl_3_) δ −5.12 (s, 2C, SiCH_3_), 18.54 (s, 1C, C_q_CH_3_), 26.07 (s, 3C, CH_3_), 64.46 (s, 1C, C-5), 120.71 (s, 1H, C-1), 127.85 (s, 2C, C-3), 131.41 (s, 2C, C-2), 140.60 (s, 1C, C-4) ppm; MS (FI) *m*/*z* calculated for C_13_H_22_BrOSi [M + H]^+^: 301.1, found: 301.9.

Di-*tert*-butyl(4-(((*tert*-butyldimethylsilyl)oxy)methyl)phenyl)fluorosilane (**23**): The reaction was carried out in heat-dried glassware under an N_2_ atmosphere. ^t^BuLi in pentane (1.6 M, 2.2 mL, 3.49 mmol, 2.1 equiv.) was added over a period of 15 min to a −78 °C cooled solution of **22** (500 mg, 1.66 mmol, 1.0 equiv.) in 2 mL dry Et_2_O. After stirring for 15 min at −78 °C, a solution of di-*tert*-butyldifluorosilane (385 mg, 2.14 mmol, 1.3 equiv.) in 1 mL dry Et_2_O was added over a period of 15 min at −78 °C. The reaction mixture was stirred for 2 d at room temperature, quenched with 10 mL saturated aqueous NaCl solution and extracted with 3 × 15 mL Et_2_O. The combined organic layers were dried over Na_2_SO_4_ and concentrated under reduced pressure. Purification by column chromatography (*n*-hexane) gave compound **23** (582 mg, 1.52 mmol, 92%) as a colorless liquid. ^1^H NMR (500 MHz, CDCl_3_) δ 0.11 (s, 6H, SiCH_3_), 0.96 (s, 9H, CH_3_), 1.06 (s, 18H, H-1), 4.77 (s, 2H, H-7), 7.34 (d, *J* = 7.8 Hz, 2H, H-5), 7.57 (d, *J* = 7.8 Hz, 2H, H-4) ppm; ^13^C NMR (126 MHz, CDCl_3_) δ −5.11 (s, 2C, SiCH_3_), 18.60 (s, 1H, C_q_CH_3_), 20.41 (d, *J* = 12.4 Hz, 2C, C-2), 26.13 (s, 3C, CH_3_), 27.50 (d, *J* = 0.9 Hz, 6C, C-1), 65.00 (s, 1C, C-7), 125.33 (d, *J* = 0.9 Hz, 2C, C-5), 131.98 (d, *J* = 13.6 Hz, 1C, C-3), 134.07 (d, J = 4.2 Hz, 2C, C-4), 142.97 (s, 1C, C-6) ppm; ^19^F NMR (282 MHz, CDCl_3_) δ −188.99 (s, 1F, SiF) ppm; MS (FD) *m*/*z* calculated for C_21_H_39_FOSi_2_ [M]^+^: 382.2, found: 382.2; MS (DART) *m*/*z* calculated for C_21_H_38_FOSi_2_ [M − H]^+^: 381.2445, found: 381.2440.

(4-(Di-*tert*-butylfluorosilyl)phenyl)methanol (**24**): 6 μL (1 vol.-%) conc. HCl was added to a colorless solution of **23** (72.7 mg, 190 μmol, 1.0 equiv.) in 600 μL MeOH. After stirring for 2 h at room temperature, the solvent was removed under reduced pressure. The crude product was purified by column chromatography (*n*-hexane/EtOAc 9/1 → 1/1) to give compound **24** (50.8 mg, 189 μmol, 99%) as a colorless solid. ^1^H NMR (500 MHz, CDCl_3_) δ 1.06 (s, 18H, H-1), 4.72 (s, 2H, H-7), 7.38 (d, *J* = 7.8 Hz, 2H, H-5), 7.61 (d, *J* = 7.8 Hz, 2H, H-4) ppm; ^13^C NMR (126 MHz, CDCl_3_) δ 20.40 (d, *J* = 12.5 Hz, 2C, C-2), 27.47 (d, *J* = 1.0 Hz, 6C, C-1), 65.42 (s, 1C, C-7), 126.26 (d, *J* = 1.0 Hz, 2C, C-5), 133.10 (d, *J* = 13.7 Hz, 1C, C-3), 134.39 (d, *J* = 4.2 Hz, 2C, C-4), 142.26 (s, 1C, C-6) ppm; ^19^F NMR (282 MHz, CDCl_3_) δ −188.90 (s, 1F, SiF) ppm; MS (FD) *m*/*z* calculated for C_15_H_25_FOSi [M]^+^: 268.1, found: 268.1; MS (DART) *m*/*z* calculated for C_15_H_29_FNOSi [M + NH_4_]^+^: 286.1997, found: 286.1996.

(4-(Bromomethyl)phenyl)di-*tert*-butylfluorosilane (**25**): All steps were carried out under an N_2_ atmosphere. PPh_3_ (1.25 g, 4.70 mmol, 1.1 equiv.) was added in portions over a period of 15 min to a solution of **24** (1.15 g, 4.27 mmol, 1.0 equiv.) and *tetra*-bromomethane (1.57 g, 4.70 mmol, 1.1 equiv.) in 20 mL dry CH_2_Cl_2_. After stirring for 12 h at room temperature, the solvent was removed under reduced pressure, and the crude product was purified by column filtration (*n*-hexane) to give compound **25** (1.34 g, 4.04 mmol, 95%) as a colorless solid. ^1^H NMR (500 MHz, CDCl_3_) δ 1.05 (s, 18H, H-1), 4.50 (s, 2H, H-7), 7.40 (d, *J* = 7.9 Hz, 2H, H-5), 7.58 (d, *J* = 7.9 Hz, 2H, H-4) ppm; ^13^C NMR (126 MHz, CDCl_3_) δ 20.41 (d, *J* = 12.4 Hz, 2C, C-2), 27.45 (d, *J* = 0.9 Hz, 6C, C-1), 33.48 (s, 1C, C-7), 128.29 (d, *J* = 1.0 Hz, 2C, C-5), 134.31 (d, *J* = 13.7 Hz, 1C, C-3), 134.54 (d, *J* = 4.3 Hz, 2C, C-4), 139.05 (s, 1C, C-6) ppm; ^19^F NMR (282 MHz, CDCl_3_) δ −188.83 (s, 1F, SiF) ppm; MS (FD) *m*/*z* calculated for C_15_H_24_BrFSi [M]^+^: 330.1, found: 330.1.

*N*-(4-(Di-*tert*-butylfluorosilyl)benzyl)-4,4-diethoxy-*N*,*N*-dimethylbutan-1-aminium bromide (**26**): All steps were carried out under an N_2_ atmosphere. Moreover, 4,4-diethoxy-*N,N*-dimethylbutan-1-amine (0.76 g, 0.9 mL, 4.04 mmol, 1.0 equiv.) was added to a solution of **25** (1.34 g, 4.04 mmol, 1.0 equiv.) in 20 mL dry CH_2_Cl_2_. After stirring for 12 h at room temperature, the solvent was removed under reduced pressure and the crude product was purified by column chromatography (CH_2_Cl_2_/MeOH, 20/1 → 5/1) to give compound **26** (1.96 g, 3.77 mmol, 93%) as a colorless foam. ^1^H NMR (500 MHz, CDCl_3_) δ 1.02 (s, 18H, H-1), 1.16 (t, *J* = 7.0 Hz, 6H, CH_3_), 1.66–1.71 (m, 2H, H-9), 1.90–1.97 (m, 2H, H-10), 3.25 (s, 6H, NCH_3_), 3.43–3.51 (m, 4H, OCH_2_), 3.59–3.66 (m, 2H, H-8), 4.50 (t, *J* = 4.6 Hz, 1H, H-11), 4.84 (s, 2H, H-7), 7.60 (d, *J* = 7.9 Hz, 2H, H-5), 7.67 (d, *J* = 7.9 Hz, 2H, H-4) ppm; ^13^C NMR (126 MHz, CDCl_3_) δ 15.44 (s, 2C, CH_3_), 17.58 (s, 1C, C-10), 20.30 (d, *J* = 12.2 Hz, 2C, C-2), 27.34 (s, 6C, C-1), 30.40 (s, 1C, C-9), 50.00 (s, 2C, NCH_3_), 62.62 (s, 2C, OCH_2_), 63.51 (s, 1C, C-8), 67.65 (s, 1C, C-7), 102.03 (s, 1C, C-11), 128.53 (s, 1C, C-6), 132.24 (s, 2C, C-5), 134.84 (d, *J* = 4.3 Hz, 2C, C-4), 137.53 (d, *J* = 13.9 Hz, 2C, C-3) ppm; ^19^F NMR (282 MHz, CDCl_3_) δ −188.58 (s, 1F, SiF) ppm; MS (FD) *m*/*z* calculated for C_25_H_47_FNO_2_Si [M]^+:^ 440.3, found: 440.1; HR-ESI-MS *m*/*z* calculated for C_25_H_47_FNO_2_Si [M]^+^: 440.3355, found: 440.3356.

*N*-(4-(Di-*tert*-butylfluorosilyl)benzyl)-*N,N*-dimethyl-4-oxobutan-1-aminium bromide (**27**): 1 mL of a TFA/H_2_O (*v*/*v*, 95/5) solution was added to **26** (48.4 mg, 93.0 mmol, 1.0 equiv.). After stirring for 30 min at room temperature, saturated aqueous NaCl solution was added, and the reaction mixture was acidified with neat HCl. The solution was extracted with EtOAc, the combined organic layers were dried over Na_2_SO_4_ and concentrated under reduced pressure. Purification by column chromatography (CH_2_Cl_2_/MeOH 100/1 → 5/1) gave compound **27** (37.7 mg, 84.4 mmol, 91%) as a colorless oil. ^1^H NMR (500 MHz, CD_3_OD) δ 1.08 (s, 18H, H-1), 1.63–1.69 (m, 2H, H-10), 1.96–2.04 (m, 2H, H-9), 3.05 (s, 6H, NCH_3_), 3.34–3.38 (m, 2H, H-8), 4.54 (s, 2H, H-7), 7.61 (d, *J* = 8.0 Hz, 2H, H-5), 7.80 (d, *J* = 8.0 Hz, 2H, H-4) ppm; ^13^C NMR (126 MHz, CD_3_OD) δ 18.95 (s, 1C, C-9), 21.02 (d, *J* = 12.1 Hz, 2C, C-2), 27.68 (s, 6C, C-1), 34.23 (s, 1C, C-10), 50.51 (s, 2C, NCH_3_), 65.54 (s, 1C, C-8), 68.61 (s, 1C, C-7), 130.30 (s, 1C, C-6), 133.25 (d, *J* = 3.4 Hz, 2C, C-5), 135.93 (d, *J* = 4.2 Hz, 2C, C-4), 138.06 (s, 1C, C-3) ppm; ^19^F NMR (282 MHz, CD_3_OD) δ −189.05 (s, 1F, SiF) ppm; MS (FD) m/z calculated for C_21_H_37_FNOSi [M]^+^: 366.2, found: 366.1; HR-ESI-MS *m*/*z* calculated for C_21_H_37_FNOSi [M]^+^: 366.2623, found: 366.2624.

3-Carboxy-*N*-(4-(di-*tert*-butylfluorosilyl)benzyl)-*N*,*N*-dimethylpropan-1-aminium bromide (**28**): 1 M KMnO_4_ (503 μL, 503 μmol, 6.0 equiv.) and 1.25 M NaH_2_PO_4_ solution (335 μL, 419 μmol, 5.0 equiv.) were added to a solution of **27** (37.3 mg, 83.5 μmol, 1.0 equiv.) in 1.2 mL *^t^*BuOH and 55 μL CH_2_Cl_2_. After the violet reaction mixture was stirred for 100 min at room temperature, it was diluted with H_2_O and quenched with saturated aqueous Na_2_SO_3_ solution. The precipitated MnO_2_ in clear solution was dissolved by adding 12 M HCl. The solution was extracted with EtOAc, and the combined organic layers were dried over Na_2_SO_4_ and concentrated under reduced pressure. After purification by HPLC (semipreparative, 20–60% MeCN + 0.1% TFA in 8 min, t_R_ = 4.54 min), compound **28** was isolated as a colorless solid (26.6 mg, 57.5 μmol, yield: 69%, purity: >99%). ^1^H NMR (500 MHz, CD_3_OD) δ 1.08 (s, 18H, H-1), 2.10–2.23 (m, 2H, H-9), 2.47 (t, *J* = 6.8 Hz, 2H, H-10), 3.07 (s, 6H, NCH_3_), 3.35–3.47 (m, 2H, H-8), 4.56 (s, 2H, H-7), 7.63 (d, *J* = 7.9 Hz, 2H, H-5), 7.80 (d, *J* = 7.9 Hz, 2H, H-4) ppm; ^13^C NMR (126 MHz, CD_3_OD) δ 19.19 (s, 1C, C-9), 21.02 (d, *J* = 12.3 Hz, 2C, C-2), 27.50 (s, 6C, C-1), 30.87 (s, 1C, C-10), 50.51 (s, 2C, NCH_3_), 65.00 (s, 1C, C-8), 68.84 (s, 1C, C-7), 130.21 (s, 1C, C-6), 133.31 (s, 2C, C-5), 135.94 (d, *J* = 4.4 Hz, 2C, C-4), 138.50 (d, *J* = 13.9 Hz, 1C, C-3), 175.32 (s, 1C, C-11) ppm; ^19^F NMR (282 MHz, CD_3_OD) δ −189.00 (s, 1F, SiF) ppm; MS (FD) *m*/*z* calculated for C_21_H_35_FNO_2_Si [M − 2H]^+^ 380.2, found 380.2; MS (MALDI) *m*/*z* calculated for C_21_H_37_FNO_2_Si [M]^+^: 382.26, found: 382.35; HR-ESI-MS *m*/*z* calculated for C_21_H_37_FNO_2_Si [M]^+^: 382.2572, found: 382.2575.

(*S*)-4-((6-Amino-5-(2-(bis(3-aminopropyl)amino)acetamido)-6-oxohexyl)amino)-*N*-(4-(di-*tert*-butylfluoro-silyl)benzyl)-*N*,*N*-dimethyl-4-oxobutan-1-aminium bromide (SiFA*lin*-APG) (**29**): According to GP1, Fmoc-Lys(Mtt)-OH (87.5 mg, 140 μmol, 4.0 equiv.) and ((Fmoc-NH-propyl)_2_Gly-OHxKHSO_4_ (108 mg, 140 μmol, 4.0 equiv.) were coupled one after the other using DIPEA (23.8 μL, 140 μmol, 4.0 equiv.) and PyBOP (71.4 mg, 140 μmol, 3.9 equiv.) in DMF for 60 min on a rink amide resin MBHA LL (106 mg, 35 μmol, 1.0 equiv., 0.33 mmol/g). After deprotection of the Mtt-PG with TFA/CH_2_Cl_2_ (1/99, *v*/*v*) for 90 min, **28** was coupled with DIPEA and PyBOP on the resin. After Fmoc-deprotection with piperidine/DMF (1/1, *v*/*v*) for 2x10 min, the product was cleaved from resin with TFA/TIS/H_2_O (95/2.5/2.5, *v*/*v*/*v*) for 2 h. Peptide **29** was purified by HPLC (semipreparative, 0–60% MeCN + 0.1% TFA in 8 min, t_R_ = 4.18 min) and isolated as a colorless solid (11.5 mg, 15.1 μmol, yield: 43%, purity: >99%). ^1^H NMR (500 MHz, D_2_O) δ 1.07 (s, 18H, H-1), 1.36–1.42 (m, 2H, H-14), 1.49–1.56 (m, 2H, H-13), 1.72–1.85 (m, 2H, H-15), 2.10–2.20 (m, 6H, H-9, H-20), 2.34 (t, *J* = 7.0 Hz, 2H, H-10), 3.08 (s, 6H, NCH_3_), 3.10 (d, *J* = 7.7 Hz, 4H, H-21), 3.16 (t, *J* = 7.2 Hz, 2H, H-12), 3.22–3.29 (m, 2H, H-8), 3.33–3.40 (m, 4H, H-19), 4.16 (d, *J* = 5.1 Hz, 2H, H-18), 4.26–4.30 (t, *J* = 7.3 Hz, 1H, H-16), 4.54 (s, 2H, H-7), 7.58 (d, *J* = 7.9 Hz, 2H, H-5), 7.83 (d, *J* = 7.9 Hz, 2H, H-4) ppm; ^13^C NMR (126 MHz, D_2_O) δ 18.48 (s, 1C, C-9), 19.21 (d, *J* = 12.2 Hz, 2C, C-2), 21.88 (s, 2C, C-20), 22.36 (s, 1C, C-14), 26.37 (s, 6C, C-1), 27.91 (s, 1C, C-13), 30.50 (s, 1C, C-15), 31.75 (s, 1C, C-10), 36.35 (s, 2C, C-21), 39.04 (s, 1C, C-12), 49.82 (s, 2C, NCH_3_), 52.28 (s, 2C, C-19), 53.99 (s, 1C, C-16), 54.26 (s, 1C, C-18), 62.58 (s, 1C, C-8), 67.82 (s, 1C, C-7), 128.30 (s, 1C, C-6), 131.82 (s, 2C, C-5), 134.64 (d, *J* = 4.2 Hz, 2C, C-4), 136.81 (d, *J* = 14.2 Hz, 2, C-3), 165.43 (s, 1C, C-17), 173.84 (s, 1C, C-11), 176.11 (s, 1C, CO) ppm; ^19^F NMR (282 MHz, D_2_O) δ −188.04 (s, 1F, SiF) ppm; MS (MALDI) *m*/*z* calculated for C_35_H_67_FN_7_O_3_Si [M]^+^:680.51, found: 680.35; HR-ESI-MS *m*/*z* calculated for C_35_H_68_FN_7_O_3_Si [M + H]^2+^:340.7563, found: 340.7558; *m*/*z* calculated for C_35_H_67_FN_7_O_3_Si [M]^+^:680.5053, found: 680.5042.

#### 3.2.4. Conjugation of **10**–**21** to the SiFA*lin*-Modified Framework **29** to Obtain the Target HBPLs **1**–**6** via the Intermediates **30**–**35**

General procedure for the synthesis of SiFA*lin*-APG-PEG_n_ peptides (GP4): All steps were carried out under an N_2_ atmosphere. A total of 0.5–1.0 equiv. of the respective NHS-PEG_n_-peptide and 0.5–2.0 equiv. DIPEA were added to a solution of 1.0 equiv. **29** in dry DMF. Subsequently, the reaction mixture was stirred for 15–150 min at room temperature, while a reaction control was performed by HPLC (analytical, 0–50% MeCN + 0.1% TFA in 5 min). After the removal of the solvent under reduced pressure, the respective SiFA*lin*-APG-PEG_n_-peptide was obtained after semipreparative HPLC purification. In addition to the target products, small amounts of the dimer SiFA*lin*-APG-[PEG_n_-peptide]_2_ were isolated (for analytical data, see Supplementary Materials Figures S85–S88).

SiFA*lin*-APG-PEG_1_-c(RGDfK) (**30**): According to GP4, **29** (9.3 mg, 12.1 μmol, 1.0 equiv.), **10** (5.4 mg, 6.1 μmol, 0.5 equiv.) and DIPEA (1.0 μL, 0.8 mg, 6.1 μmol, 0.5 equiv.) were reacted in 16 mL dry DMF for 80 min. After purification by HPLC (semipreparative, 15–50% MeCN + 0.1% TFA in 10 min, t_R_ = 5.64 min), **30** was isolated as a colorless solid (11.2 mg, 7.3 μmol, yield: 60%, purity: >96%). MS (MALDI) *m*/*z* calculated for C_70_H_118_N_16_O_14_Si [M]^+^: 1453.88, found: 1453.58; HR-ESI-MS *m*/*z* calculated for C_70_H_120_FN_16_O_14_Si [M + 2H]^3+^: 485.2969, found: 485.2967; HR-ESI-MS *m*/*z* calculated for C_70_H_119_FN_16_O_14_Si [M + 2H]^2+^: 727.4417, found: 727.4410.

SiFA*lin*-APG-PEG_3_-c(RGDfK) (**31**): According to GP4, **29** (5.0 mg, 6.5 μmol, 1.0 equiv.), **11** (6.1 mg, 6.5 μmol, 1.0 equiv.) and DIPEA (2.2 μL, 1.7 mg, 13.0 μmol, 2.0 equiv.) were reacted in 7 mL dry DMF for 45 min. After purification by HPLC (semipreparative, 15–50% MeCN + 0.1% TFA in 10 min, t_R_ = 5.95 min), **31** was isolated as a colorless solid (7.1 mg, 4.4 μmol, yield: 67%, purity: >96%). MS (MALDI) *m*/*z* calculated for C_74_H_126_FN_16_O_16_Si [M]^+^ 1541.93, found: 1541.82; HR-ESI-MS *m*/*z* calculated for C_74_H_128_FN_16_O_16_Si [M + 2H]^3+^: 514.6477, found: 514.6474; HR-ESI-MS *m*/*z* calculated for C_74_H_127_FN_16_O_16_Si [M + 2H]^2+^: 771.4679, found: 771.4676; HR-ESI-MS *m*/*z* calculated for C_74_H_126_FN_16_O_16_Si [M]^+^: 1541.9286, found: 1541.9278.

SiFA*lin*-APG-PEG_5_-c(RGDfK) (**32**): According to GP4, **29** (12.0 mg, 15.8 μmol, 1.0 equiv.), **12** (8.7 mg, 8.2 μmol, 0.5 equiv.) and DIPEA (2.7 μL, 2.0 mg, 15.8 μmol, 1.0 equiv.) were reacted in 14 mL dry DMF for 150 min. After purification by HPLC (semipreparative, 0–50% MeCN + 0.1% TFA in 8 min, t_R_ = 7.10 min), **32** was isolated as a colorless solid (10.6 mg, 6.5 μmol, yield: 41%, purity: >99%). MS (MALDI) *m*/*z* calculated for C_78_H_134_FN_16_O_18_Si [M]^+^: 1629.98, found: 1629.16; HR-ESI-MS *m*/*z* for C_78_H_137_FN_16_O_18_Si [M + 3H]^4+^: 408.2507, found: 408.2506; HR-ESI-MS *m*/*z* calculated for C_78_H_136_FN_16_O_18_Si [M + 2H]^3+^: 543.9985, found: 543.9983; HR-ESI-MS *m*/*z* calculated for C_78_H_135_FN_16_O_18_Si [M + H]^2+^: 815.4941, found: 815.4933.

SiFA*lin*-APG-PEG_8_-c(RGDfK) (**33**): According to GP4, **29** (7.3 mg, 9.6 μmol, 1.0 equiv.), **13** (5.9 mg, 4.9 μmol, 0.5 equiv.) and DIPEA (1.6 μL, 1.2 mg, 9.6 μmol, 1.0 equiv.) were reacted in 10 mL dry DMF for 130 min. After purification by HPLC (semipreparative, 0–50% MeCN + 0.1% TFA in 8 min, t_R_ = 7.27 min), **33** was isolated as a colorless solid (8.7 mg, 4.7 μmol, yield: 49%, purity: >99%). MS (MALDI) *m*/*z* calculated for C_84_H_146_FN_16_O_21_Si [M]^+^: 1762.06, found: 1762.21; HR-ESI-MS *m*/*z* calculated for C_84_H_148_FN_16_O_21_Si [M + 2H]^3+^: 588.3592, found: 588.3585; HR-ESI-MS *m*/*z* calculated for C_84_H_147_FN_16_O_21_Si [M + H]^2+^: 881.5335, found: 881.5559.

General procedure for the synthesis of SiFA*lin*-APG-DIG- and SiFA*lin*-APG-Ox-EGEGE peptides (GP5): All steps were carried out under an N_2_ atmosphere. Then, 0.5 equiv. PyBOP and 0.5–2.0 equiv. DIPEA was added to a solution of 0.5 equiv. HO-DIG/Ox-EGEGE-peptide in dry DMF and stirred for 15 min. Subsequently, 1.0 equiv. **29** was added and the reaction mixture was stirred for 1–4 h at room temperature, while the reaction control was performed by HPLC (analytical, 0–50% MeCN + 0.1% TFA in 5 min). After the removal of the solvent under reduced pressure, the respective SiFA*lin*-APG-DIG/Ox-EGEGE-peptide was obtained after semipreparative HPLC purification. In addition to the target product, small amounts of the dimer SiFA*lin*-APG-[DIG/Ox-EGEGE-peptide]_2_ were isolated (for analytical data, see Supplementary Materials Figures S89–S90).

SiFA*lin*-APG-DIG-c(RGDfK) (**34**): According to GP5, **29** (3.9 mg, 5.1 μmol, 1.0 equiv.), **14** (1.9 mg, 2.6 μmol, 0.5 equiv.), PyBOP (1.4 mg, 2.6 μmol, 0.5 equiv.) and DIPEA (0.4 μL, 0.3 mg, 2.6 μmol, 0.5 equiv.) were reacted in 2.5 mL dry DMF for 1 h. After purification by HPLC (semipreparative, 5–40% MeCN + 0.1% TFA in 10 min, t_R_ = 3.09 min), **34** was isolated as a colorless solid (3.4 mg, 2.3 μmol, yield: 46%, purity: >97%). MS (MALDI) *m*/*z* calculated for C_66_H_110_N_16_O_13_Si [M]^+^: 1381.82, found: 1381.45; HR-ESI-MS *m*/*z* calculated for C_66_H_112_FN_16_O_13_Si [M + 2H]^3+^: 461.2777, found: 461.2772; HR-ESI-MS *m*/*z* calculated for C_66_H_111_FN_16_O_13_Si [M + H]^2+^: 691.4129, found: 691.4125.

SiFA*lin*-APG-Ox-EGEGE-c(RGDfK) (**35**): According to GP5, **29** (1.3 mg, 1.7 μmol, 1.0 equiv.), **15** (1.0 mg, 0.9 μmol, 0.5 equiv.), PyBOP (0.4 mg, 0.9 μmol, 0.5 equiv.) and DIPEA (0.5 μL, 0.4 mg, 3.4 μmol, 2.0 equiv.) were reacted in 1.5 mL dry DMF for 4 h. After purification by HPLC (semipreparative, 0–50% MeCN + 0.1% TFA in 8 min, t_R_ = 6.56 min), **35** was isolated as a colorless solid (1.9 mg, 1.0 μmol, yield: 58%, purity: >98%). MS (MALDI) m/z calculated for C_83_H_134_FN_21_O_23_Si [M+H]^+^: 1839.97, found: 1839.06; HR-ESI-MS m/z calculated for C_37_H_69_FN_8_O_5_Si [M-C_46_H_64_N_13_O_18_]^+^: 752.5139, found: 752.5257; HR-ESI-MS m/z calculated for C_44_H_81_FN_10_O_9_Si [M-C_44_H_52_N_11_O_14_]^2+^: 940.5936, found: 940.4168.

General procedure for the synthesis of heterobivalent SiFA*lin*-modified HBPLs **1**–**4** (GP6): All steps were carried out under an N_2_ atmosphere. 3.0–6.0 equiv. DIPEA was added within a period of 0.5–6 h to a solution of 1.0 equiv. SiFA*lin*-APG-PEG_n_-c(RGDfK) and 1.0–2.0 equiv. NHS-PEG_n_-GG-Nle-c(DHfRWK) in dry DMF. Subsequently, the reaction mixture was stirred for 60 min at room temperature, while the reaction control was performed by HPLC (analytical, 0–50% MeCN + 0.1% TFA in 5 min). After the removal of the solvent under reduced pressure, the respective SiFA*lin*-APG-PEG_n_-c(RGDfK)/GG-Nle-c(DHfRWK)-heterodimer was obtained after semipreparative HPLC purification.

SiFA*lin*-APG-PEG_1_-c(RGDfK)/GG-Nle-c(DHfRWK) (**1**): According to GP6, **30** (5.1 mg, 3.3 μmol, 1.0 equiv.), **16** (6.1 mg, 4.4 μmol, 1.3 equiv.) and DIPEA (6 × 0.6 μL, 2.6 mg, 19.8 μmol, 6.0 equiv.) were reacted in 9 mL dry DMF for 7 h. After purification by HPLC (semipreparative, 15–50% MeCN + 0.1% TFA in 10 min, t_R_ = 6.91 min), HBPL **1** was isolated as a colorless solid (7.0 mg, 2.5 μmol, yield: 77%, purity: >99%). ^19^F NMR (282 MHz, D_2_O) δ −176.21 (s, 1F, SiF) ppm; MS (MALDI) *m*/*z* calculated for C_130_H_202_FN_33_O_28_Si [M + H]^+^: 2720.51, found: 2720.11; HR-ESI-MS *m*/*z* calculated for C_130_H_205_FN_33_O_28_Si [M + 4H]^5+^: 544.9078, found: 544.9072; HR-ESI-MS *m*/*z* calculated for C_130_H_204_FN_33_O_28_Si [M + 3H]^4+^: 680.8830, found: 680.8824.

SiFA*lin*-APG-PEG_3_-c(RGDfK)/GG-Nle-c(DHfRWK) (**2**): According to GP6, **31** (4.3 mg, 2.7 μmol, 1.0 equiv.), **17** (6.9 mg, 4.7 μmol, 1.8 equiv.) and DIPEA (5x0.5 μL, 1.9 mg, 14.7 μmol, 5.0 equiv.) were reacted in 11 mL dry DMF for 7 h. After purification by HPLC (semipreparative, 15–50% MeCN + 0.1% TFA in 10 min, t_R_ = 7.10 min), HBPL **2** was isolated as a colorless solid (5.3 mg, 1.8 μmol, yield: 66%, purity: >99%). ^19^F NMR (282 MHz, D_2_O) δ −176.21 (s, 1F, SiF) ppm; MS (MALDI) *m*/*z* calculated for C_138_H_218_FN_33_O_32_Si [M + H]^+^: 2896.62, found: 2896.11; HR-ESI-MS *m*/*z* calculated for C_138_H_220_FN_33_O_32_Si [M + 3H]^4+^: 724.9092, found: 724.9101; HR-ESI-MS *m*/*z* calculated for C_138_H_219_FN_33_O_32_Si [M + 2H]^3+^: 966.2098, found: 966.2106; HR-ESI-MS *m*/*z* calculated for C_138_H_218_FN_33_O_32_Si [M + H]^2+^: 1448.8111, found: 1448.8124.

SiFA*lin*-APG-PEG_5_-c(RGDfK)/GG-Nle-c(DHfRWK) (**3**): According to GP6, **32** (4.2 mg, 2.5 μmol, 1.0 equiv.), **18** (4.6 mg, 3.0 μmol, 1.2 equiv.) and DIPEA (2 × 0.8 μL, 3.3 mg, 19.5 μmol, 4.0 equiv.) were reacted in 5 mL dry DMF for 3 h. After purification by HPLC (semipreparative, 10–50% MeCN + 0.1% TFA in 8 min, t_R_ = 7.26 min), HBPL **3** was isolated as a colorless solid (3.8 mg, 1.2 μmol, yield: 49%, purity: >99%). ^19^F NMR (282 MHz, D_2_O) δ −176.21 (s, 1F, SiF) ppm; MS (MALDI) *m*/*z* calculated for C_146_H_233_FN_33_O_36_Si [M]^+^: 3071.72, found: 3071.49; HR-ESI-MS *m*/*z* calculated for C_146_H_237_FN_33_O_36_Si [M + 4H]^5+^: 615.3498, found: 615.3494; HR-ESI-MS *m*/*z* calculated for C_146_H_236_FN_33_O_36_Si [M + 3H]^4+^: 768.9354, found: 768.9353.

SiFA*lin*-APG-PEG_8_-c(RGDfK)/GG-Nle-c(DHfRWK) (**4**): According to GP6, **33** (6.4 mg, 3.5 μmol, 1.0 equiv.), **19** (11.2 mg, 6.6 μmol, 1.9 equiv.) and DIPEA (6 × 0.6 μL, 2.7 mg, 20.9 μmol, 6.0 equiv.) were reacted in 6 mL dry DMF for 4 h. After purification by HPLC (semipreparative, 10–50% MeCN + 0.1% TFA in 8 min, t_R_ = 7.55 min), HBPL **4** was isolated as a colorless solid (3.0 mg, 2.0 μmol, yield: 58%, purity: >99%). ^19^F NMR (282 MHz, D_2_O) δ −176.20 (s, 1F, SiF) ppm; MS (MALDI) *m*/*z* calculated for C_158_H_258_FN_33_O_42_Si [M]^+^ 3336.88, found: 3336.07; HR-ESI-MS *m*/*z* calculated for C_158_H_261_FN_33_O_42_Si [M + 4H]^5+^: 668.1812, found: 668.1820; HR-ESI-MS *m*/*z* calculated for C_158_H_260_FN_33_O_42_Si [M + 3H]^4+^: 835.2256, found: 835.2269.

General procedure for the synthesis of heterobivalent SiFA*lin*-modified HBPLs **5** and **6** (GP7): All steps were carried out under an N_2_ atmosphere. 1.3–3.0 equiv. PyBOP and 1.3–12.0 equiv. DIPEA were added to a solution of 1.3–3.0 equiv. HO-DIG-GG-Nle-c(DHfRWK) or Ox-EGEGE-GG-Nle-c(DHfRWK) in dry DMF and stirred for 60 min. Subsequently, 1.0 equiv. SiFA*lin*-APG-DIG-c(RGDfK) (**34**) or SiFA*lin*-APG-Ox-EGEGE-c(RGDfK) (**35**) were added, and the reaction mixture was reacted for 2–5 h at room temperature, while the reaction control was performed by HPLC (analytical, 0–50% MeCN + 0.1% TFA in 5 min). After the removal of the solvent under reduced pressure, the SiFA*lin*-modified HBPLs **5** and **6** were obtained after semipreparative HPLC purification.

SiFA*lin*-APG-DIG-c(RGDfK)/GG-Nle-c(DHfRWK) (**5**): According to GP7, **34** (1.8 mg, 1.2 μmol, 1.0 equiv.), **20** (2.0 mg, 1.6 μmol, 1.3 equiv.), PyBOP (0.8 mg, 1.6 μmol, 1.3 equiv.) and DIPEA (0.3 μL, 0.2 mg, 1.6 μmol, 1.3 equiv.) were reacted in 2 mL dry DMF for 5 h. After purification by HPLC (semipreparative, 10–40% MeCN + 0.1% TFA in 11 min, t_R_ = 10.18 min), HBPL **5** was isolated as a colorless solid (0.7 mg, 0.3 μmol, yield: 24%, purity: >99%). ^19^F NMR (282 MHz, D_2_O) δ −176.21 (s, 1F, SiF) ppm; MS (MALDI) *m*/*z* calculated for C_122_H_185_FN_33_O_26_Si [M]^+^: 2575.39, found: 2575.82; HR-ESI-MS *m*/*z* calculated for C_122_H_189_FN_33_O_26_Si [M + 4H]^5+^: 516.0848, found: 516.0843; HR-ESI-MS *m*/*z* calculated for C_122_H_186_FN_33_O_26_Si [M + 3H]^4+^: 644.8542, found: 644.8542.

SiFA*lin*-APG-Ox-EGEGE-c(RGDfK)/GG-Nle-c(DHfRWK) (**6**): According to GP7, **35** (1.3 mg, 0.7 μmol, 1.0 equiv.), **21** (3.4 mg, 2.0 μmol, 3.0 equiv.), PyBOP (1.1 mg, 2.0 μmol, 3.0 equiv.) and DIPEA (4 × 0.3 μL, 1.1 mg, 8.4 μmol, 12.0 equiv.) were reacted in 2.5 mL dry DMF for 3.5 h. After purification by HPLC (semipreparative, 0–50% MeCN + 0.1% TFA in 10 min, t_R_ = 8.54 min; 20–40% MeCN + 0.1% TFA in 10 min, t_R_ = 7.24 min), HBPL **6** was isolated as a colorless solid (0.5 mg, 0.15 μmol, yield: 21%, purity: >99%). ^19^F NMR (282 MHz, D_2_O) δ −176.19 (s, 1F, SiF) ppm; MS (MALDI) *m*/*z* calculated for C_156_H_231_FN_43_O_46_Si [M]^+^: 3489.68, found: 3489.25; HR-ESI-MS *m*/*z* calculated for C_129_H_190_FN_35_O_39_Si [M-C_27_H_41_N_8_O_7_]^5+^: 580.2744, found: 580.1284; HR-ESI-MS m/z calculated for C_129_H_189_FN_35_O_39_Si [M-C_27_H_42_N_8_O_7_]^4+^: 724.8403, found: 724.9088.

### 3.3. ^18^F-Radiolabeling, Evaluation of log_D(7.4)_, Stability and Binding Affinities for HBPLs ***1***–***6***

#### 3.3.1. ^18^F-Radiolabeling of the HBPLs **1**–**6**

Aqueous [^18^F]fluoride solution (0.3–2.0 mL, 0.8–2.2 GBq) was flushed through an anion exchange resin (Sep-Pak Accell Plus QMA Carbonate Plus SPE Light cartridge, 46 mg, Waters; Eschborn, Germany) which was preconditioned with 10 mL Tracepur H_2_O. After drying with 20 mL air, removal of the remaining water with 5 mL dry MeCN and repeated drying with 20 mL air, the radioactivity was eluted from the cartridge with KOH (100 μmol) and Kryptofix222 (K_222_, 41.4 mg, 110 μmol) in 500 μL dry MeCN. To the obtained dry [^18^F]KF-K_222_-hydroxide complex, a solution of oxalic acid in dry MeCN (25 μL, 25 μmol, 1 M) was added first and then a solution of the respective HBPL precursor **1**–**6** in dry DMSO (25 μL, 25 nmol, 1 mM) was added afterward. After reaction for 10 min at room temperature, the mixture was analyzed by analytical radio-HPLC (0–100% MeCN + 0.1% TFA in 5 min). For purification, the reaction mixture was diluted with 9 mL 0.1 M HEPES solution (pH = 2) and passed through a C18 cartridge (Sep-Pak C18 Plus SPE Light cartridge, 130 mg, Waters; Eschborn, Germany), which was preconditioned with 10 mL EtOH and 10 mL Tracepur H_2_O. The cartridge was washed with 10 mL 0.05 M phosphate buffer (pH = 7.4) and dried with 10 mL air. Finally, the radiotracer was eluted with 500 μL EtOH/Tracepur H_2_O (9/1, *v*/*v*), and the RCPs of the radiolabeled products were determined by analytical radio-HPLC (0–100% MeCN + 0.1% TFA in 5 min). The radiotracers were obtained in RCYs of 27–50%, RCPs of 95–98% and A_m_ of 17–51 GBq/μmol after an overall synthesis time of 25 min.

#### 3.3.2. Log_D(7.4)_ Determination of [^18^F]**1**–[^18^F]**6**

The radiotracer solutions were first diluted with 0.9% NaCl solution to give a final EtOH concentration of <10%. A total of 5 MBq of the respective radiotracer in solution was added to 1.6 mL of a mixture of *n*-octanol and 0.05 M phosphate buffer (pH = 7.4) (*v*/*v*, 1/1), and the solution was vigorously shaken for 5 min. Subsequently, the phases were separated by centrifugation at 13.4 rpm for 2 min, and 100 μL of organic and aqueous phases were collected. The activity of each sample was measured using a gamma counter. The lipophilicity of each compound was determined in triplicate in three independent experiments.

#### 3.3.3. Determination of the Stability of [^18^F]**1**–[^18^F]**6** in Human Serum

The radiotracer solutions were first diluted with 0.9% NaCl solution to give a final EtOH concentration of <10%. Then, 6 × 25 μL radiotracer solution was added to 6 × 100 μL human serum and incubated at 37°C for 120 min. At defined time points (5, 15, 30, 60, 90 and 120 min), 125 μL EtOH was added to one of the mixtures and the precipitation of serum proteins was supported by ice-cooling for 2 min. After centrifugation at 13.4 rpm for 2 min, the supernatant was analyzed by analytical radio-HPLC (0–100% MeCN + 0.1% TFA in 5 min). The experiment was performed for each compound trice by three independent experiments.

#### 3.3.4. Cell Culture

All cell lines were cultivated at 37 °C in a humidified incubator at 5% CO_2_. B16F10 cells were cultured in DMEM and the U87MG cells in EMEM, each medium supplemented with 10% fetal bovine serum and 1% penicillin/streptomycin. The medium was exchanged every 2–3 days and cells were split at 70–90% confluence using 0.25/0.02% Trypsin/EDTA (*w*/*v*) in PBS. A medium change was performed 24 h before an experiment. For in vivo experiments, the cell resuspension after centrifugation was performed in PBS. The cells were homogenized in PBS to give a concentration of 5 × 10^6^ B16F10 cells/mL and 25 × 10^6^ U87MG cells/mL and were aliquoted and stored on ice upon use.

#### 3.3.5. Competitive Displacement Studies on B16F10 and U87MG Cells

To determine the binding affinity to the respective receptor, competitive displacement studies were performed on MC1R-expressing B16F10 and on integrin α_v_β_3_-expressing U87MG cells. Each compound was evaluated at least three times, each experiment being performed in triplicate. As radioligands, [^125^I]I-NDP (81.4 GBq/μmol) and [^125^I]I-echistatin (81.4 GBq/μmol) were used as competitors. First, the Millipore MultiScreen 96-well filter plate was incubated with 200 μL/well of a BSA/PBS (1/99, *w*/*v*) solution at 25 °C for 1 h. After preparing the binding buffers (DMEM with 25 mM HEPES, 0.3 mM 1,10-phenanthroline and 0.2% BSA; EMEM with 20 mM Tris·HCl, 150 mM NaCl, 2 mM CaCl_2_, 1 mM MgCl_2_, 1 mM MnCl_2_ and 0.1% BSA), the dilution series of the HBPLs **1**–**6** (0.04–4 μM and 2–400 μM) and the reference agents **36**–**39** (0.01–1 μM and 0.04–400 μM) were prepared in the respective binding buffer. The solution of the respective radioligand was prepared by adding 55–75 kBq of the respective ^125^I-labeled competitor to 3.5 mL of binding buffer. The respective cells were harvested and re-suspended in the binding buffer to give a cell concentration of 2 × 10^6^/mL. After the BSA solution was filtered using the Millipore Multiscreen vacuum manifold, 50 μL of a cell suspension containing 10^5^ cells were seeded in each well. Subsequently, 25 μL of the ^125^I-labeled competitor solution (0.018 kBq/μL) and 25 μL of the respective compound to be tested were added. The compound to be tested was added in eleven increasing concentrations, whereas the 12th well contained no test compound to ensure the 100% binding of the ^125^I-labeled competitor. After incubation of the plate for another hour at 25°C, the solution was filtrated using the Millipore Multiscreen vacuum manifold, and the cells were washed three times with cold PBS (1 × 200 μL, 2 × 100 μL). Using a Millipore MultiScreen disposable punch and a Millipore MultiScreen punch kit, the filters of the well plate were collected in γ-counter tubes separately and measured by γ-counting. The determination of the half-maximal inhibitory concentration (IC_50_) values was performed by fitting the obtained data via nonlinear regression using GraphPad Prism (v6.05).

### 3.4. In Vivo PET/CT Imaging and Ex Vivo Biodistribution of [^18^F]***2*** and [^18^F]***4***

Each male nude mouse (six weeks old) was injected subcutaneously with 5 × 10^5^ B16F10 cells into the right flank and 2.0–2.5 × 10^6^ U87MG cells into the left flank. The health status and tumor growth of the mice were monitored regularly until the animals could be examined after 15–21 days, depending on the tumor size. For the in vivo experiments, the ^18^F-radiolabeled compound was diluted in 0.9% saline to give a final EtOH concentration of <10%. Each mouse was injected with 4.15 ± 2.28 MBq of [^18^F]**2** or 3.95 ± 2.06 MBq of [^18^F]**4** via the lateral tail vein under isoflurane anesthesia. For the blocking studies, the respective radiotracer was coinjected with 20 μg NDP, 200 μg c(RGDyK) or both substances (double blocking). Each ^18^F-labeled compound was studied with or without blocking in at least three mice. Mice were measured under isoflurane anesthesia in a small PET/SPECT/CT animal imaging system. First, a dynamic PET scan was performed over 90 min and the scan was framed in 29 timeframes (10 × 1 min, 10 × 2 min, 6 × 5 min, 3 × 10 min). Images were reconstructed using 12 iterations, a maximum likelihood expectation maximization (MLEM) algorithm including corrections for scattered radiation and decay and a voxel size of 0.5 mm. All PET scans were immediately followed by CT acquisition at a voltage of 45 kV and a current of 400 μA. The images were reconstructed using the filtered back projection (FBP) algorithm with a voxel size of 250 μm. The analysis of the data, including the generation of TACs of the kidneys, liver and tumors and the MIPs, was performed via PMOD (v3.8). For ex vivo biodistribution, the mice were sacrificed directly after the PET/CT scan. Organs (blood, spleen, liver, kidneys, pancreas, lungs, heart, brain, bone, muscle, tail, tumors, stomach, colon and small intestine) were collected, weighed and their radioactivity measured in a γ-counter. The percentage injected dose per gram (% ID/g) of each tissue was calculated from the determined values, organ weights, reference values and injected activity.

## 4. Conclusions

In the present study, different heterobivalent bispecific ^18^F-labeled agents for the sensitive and receptor-specific imaging of malignant melanoma using PET/CT were developed. After establishing the chemical and radio synthesis of the agents, the obtained tracers were studied systematically in vitro, regarding their hydrophilicity, stability in human serum and receptor-binding potential of both target receptor types, integrin α_v_β_3_ and MC1R. It was shown that the distance between the peptide binders strongly influences receptor affinities and that the introduction of negatively charged linkers negatively affects the receptor-binding potential of both receptor types. In vivo, the most potent tracers were studied in direct comparison to PET/CT imaging and ex vivo biodistribution studies. These experiments showed higher absolute tumor uptakes and tumor-to-background ratios and thus more favorable in vivo pharmacokinetics for the agent, demonstrating slightly lower affinities but comprising longer PEG linkers, though not for that agent exhibiting the highest receptor affinities. Heterodimer [^18^F]**4** thus demonstrated an excellent receptor-specific tumor visualization ability and impressively illustrated the suitability of the underlying concept to develop heterobivalent integrin α_v_β_3_- and MC_1_R-bispecific radioligands for the sensitive and specific imaging of malignant melanoma by PET/CT.

## Figures and Tables

**Scheme 4:**
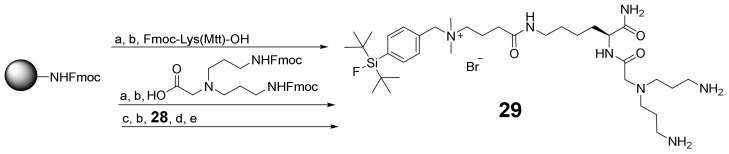
Schematic depiction of the synthesis pathway towards the SiFA*lin*-modified branching framework **29**. Conditions: (**a**) deprotection of Fmoc-PG: piperidine/DMF (1/1, *v*/*v*), 2 + 5 min; (**b**) activation of amino acids: 4.0 equiv. DIPEA, 3.9 equiv. HBTU in DMF, 2 min, coupling, 60 min; (**c**) deprotection of Mtt-PG: TFA/CH_2_Cl_2_ (1/99, *v*/*v*), 90 min; (**d**) deprotection of Fmoc-PGs: piperidine/DMF (1/1, *v*/*v*), 2 × 10 min; (**e**) cleavage from the resin: TFA/TIS/H_2_O (95/2.5/2.5, *v*/*v*/*v*), 2 h; isolated yield: 43%.

**Table 1:** Summary of the results from ^18^F-radiolabeling and in vitro log*_D_*_(7.4)_ and stability evaluations. A_0_: starting activity. Values are given as mean ± SD (standard deviation).

HBPL	Linker	RCP [%]	RCY [%]	A_m_ [GBq/µmol]	A_0_ [GBq]	log*_D_*_(7.4)_	Stability * [%]
[^18^F]**1**	PEG_1_	≥98	27.2 ± 1.4	17.5–28.6	1.0–1.6	−1.08 ± 0.07	87.0 ± 2.3
[^18^F]**2**	PEG_3_	≥98	40.3 ± 2.6	20.0–31.2	0.8–1.2	−1.19 ± 0.05	85.4 ± 2.8
[^18^F]**3**	PEG_5_	≥97	50.4 ± 3.1	25.1–49.8	0.9–2.0	−1.15 ± 0.01	81.4 ± 2.9
[^18^F]**4**	PEG_8_	≥97	38.4 ± 0.6	25.7–42.7	1.1–1.9	−1.39 ± 0.03	83.6 ± 0.8
[^18^F]**5**	DIG	≥95	27.3 ± 9.9	17.0–47.6	1.8–2.2	−1.21 ± 0.01	83.2 ± 2.4
[^18^F]**6**	Ox-EGEGE	≥97	43.2 ± 1.6	39.3–51.4	1.2–1.9	−1.52 ± 0.01	82.7 ± 1.8

* Intact radiotracer after 120 min of incubation in human serum.

**Table 2:** Summary of the IC_50_ values determined for the HBPLs **1**–**6** by competitive displacement studies on MC1R-positive B16F10 cells and integrin α_v_β_3_-positive U87MG cells. Values are given as mean ± SD.

Compound	IC_50 (B16F10)_ [nM]	Compound	IC_50 (U87MG)_ [nM]
**1**	1.74 ± 0.25	**1**	2881 ± 757
**2**	0.99 ± 0.11	**2**	1300 ± 288
**3**	3.44 ± 0.09	**3**	1911 ± 70
**4**	6.00 ± 0.47	**4**	2034 ± 323
**5**	2.05 ± 0.35	**5**	5895 ± 722
**6**	4.18 ± 0.32	**6**	>100,000
α-MSH	3.75 ± 0.61	c(RGDfC)	1493 ± 210
NDP	0.17 ± 0.04	c(RGDfK)	427 ± 37

## Data Availability

The data presented in this study are available on request from the corresponding author.

## Supplementary Materials

The following are available online at https://www.mdpi.com/article/10.3390/ph14060547/s1; analytical data, binding curves of c(RGDfK) and GG-Nle-c(DHfRWK) and ex vivo biodistribution data of [^18^F]**2** and [^18^F]**4**.
