# Supplementary material for: Design, Synthesis, In Vitro and In Vivo Evaluation of Heterobivalent SiFAlin-Modified Peptidic Radioligands Targeting Both Integrin αvβ3 and the MC1 Receptor—Suitable for the Specific Visualization of Melanomas?
Source: Pharmaceuticals (Basel). 2021 Jun 7;14(6):547. doi: 10.3390/ph14060547 (PMC8228600; doi:10.3390/ph14060547)
Supplement: Supplementary file 1 [file pharmaceuticals-14-00547-s001.zip › pharmaceuticals-1230451-supplementary.pdf]

Supplementary Information (SI)

Design, synthesis, *in vitro* and *in vivo* evaluation of heterobivalent SiFA*lin*-coupled peptidic radioligands targeting both $\alpha_v\beta_3$-integrin and MC1-receptor – Suitable for the specific visualization of melanomas?

Xia Cheng [1], Ralph Hübner [2], Valeska von Kiedrowski [1], Gert Fricker [3], Ralf Schirrmacher [4], Carmen Wängler [2,*] and Björn Wängler [1,*]

[1] Molecular Imaging and Radiochemistry, Department of Clinical Radiology and Nuclear Medicine, Medical Faculty Mannheim of Heidelberg University, Theodor-Kutzer-Ufer 1-3, 68167 Mannheim, Germany

[2] Biomedical Chemistry, Department of Clinical Radiology and Nuclear Medicine, Medical Faculty Mannheim of Heidelberg University, Theodor-Kutzer-Ufer 1-3, 68167 Mannheim, Germany

[3] Institute of Pharmacy and Molecular Biotechnology, University of Heidelberg, Im Neuenheimer Feld 329, 69120 Heidelberg, Germany

[4] Department of Oncology, Division of Oncological Imaging, University of Alberta, 11560 University Avenue, Edmonton, T6G 1Z2, Alberta, Canada

* Correspondence: Carmen.Waengler@medma.uni-heidelberg.de; Bjoern.Waengler@medma.uni-heidelberg.de

Table of Contents

1. HPLC chromatograms and mass spectra of the peptides **7**–**9** and the linker-modified peptides **10**–**21** 1

2. NMR and mass spectra of SiFA*lin* **28**, SiFA*lin*-modified framework **29** and their intermediates **22**–**27** 16

3. Mass and ^{19}F NMR spectra of HBPLs **1**–**6** and mass spectra of their intermediates **30**–**35** 47

4. Mass data, HPLC chromatograms and mass spectra of peptidic side products **a**–**z** 65

5. Radio-HPLC chromatograms of [^{18}F]**1**-[^{18}F]**6** from the investigation regarding serum stability 93

6. Binding curves of the monomeric peptides c(RGDfK) and GG-Nle-c(DHfRWK).. 93

7. *Ex vivo* biodistribution data of [^{18}F]**2** and [^{18}F]**4** 94

1. HPLC chromatograms and mass spectra of the peptides 7–9 and the linker-modified peptides 10–21

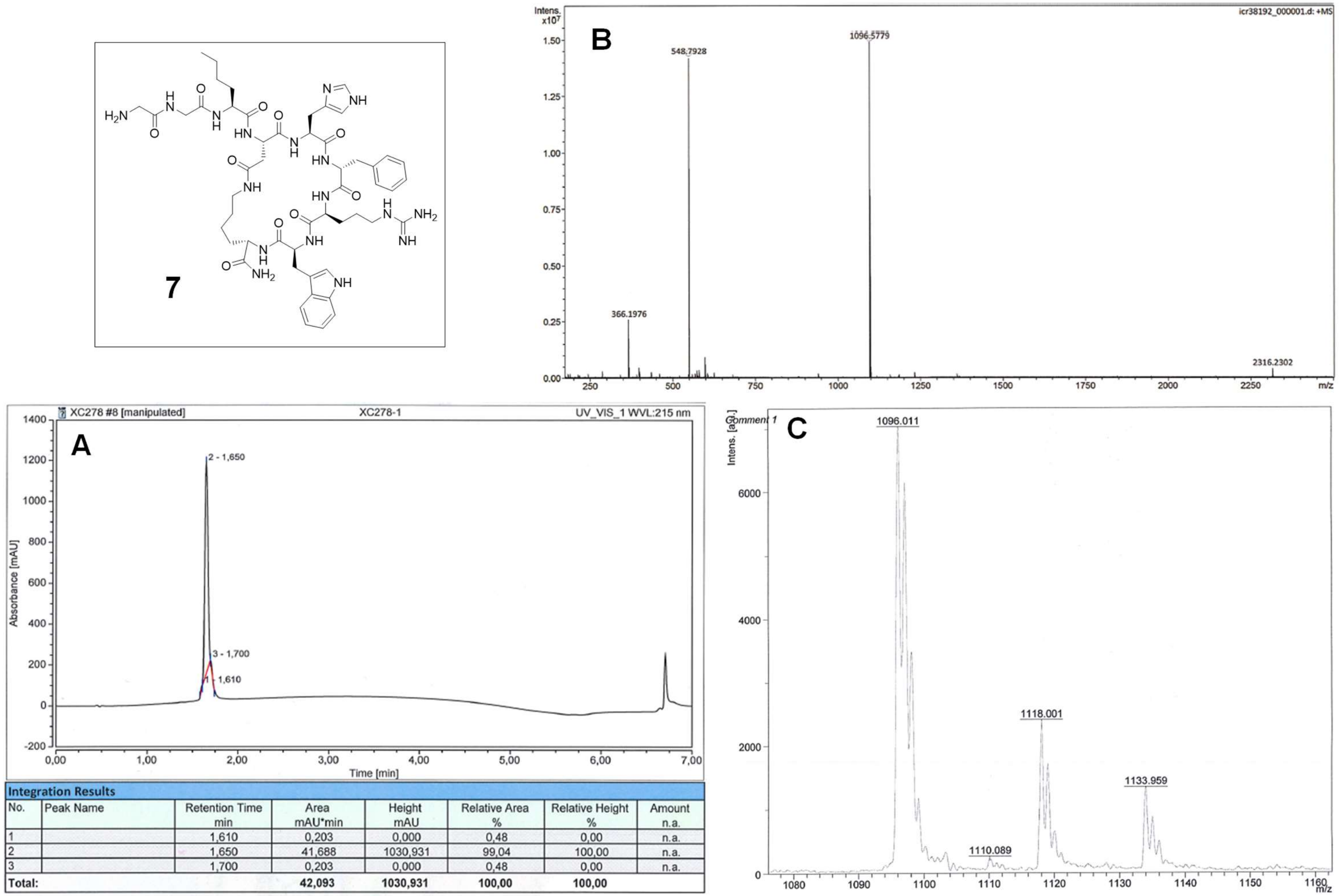

No.	Peak Name	Retention Time min	Area mAU*min	Height mAU	Relative Area %	Relative Height %	Amount n.a.
1		1,610	0,203	0,000	0,48	0,00	n.a.
2		1,650	41,688	1030,931	99,04	100,00	n.a.
3		1,700	0,203	0,000	0,48	0,00	n.a.
Total:			42,093	1030,931	100,00	100,00	

Figure S1. HPLC chromatogram (**A**) and mass spectra (**B**: ESI, **C**: MALDI) of GG-Nle-c(DHfRWK) (**7**).

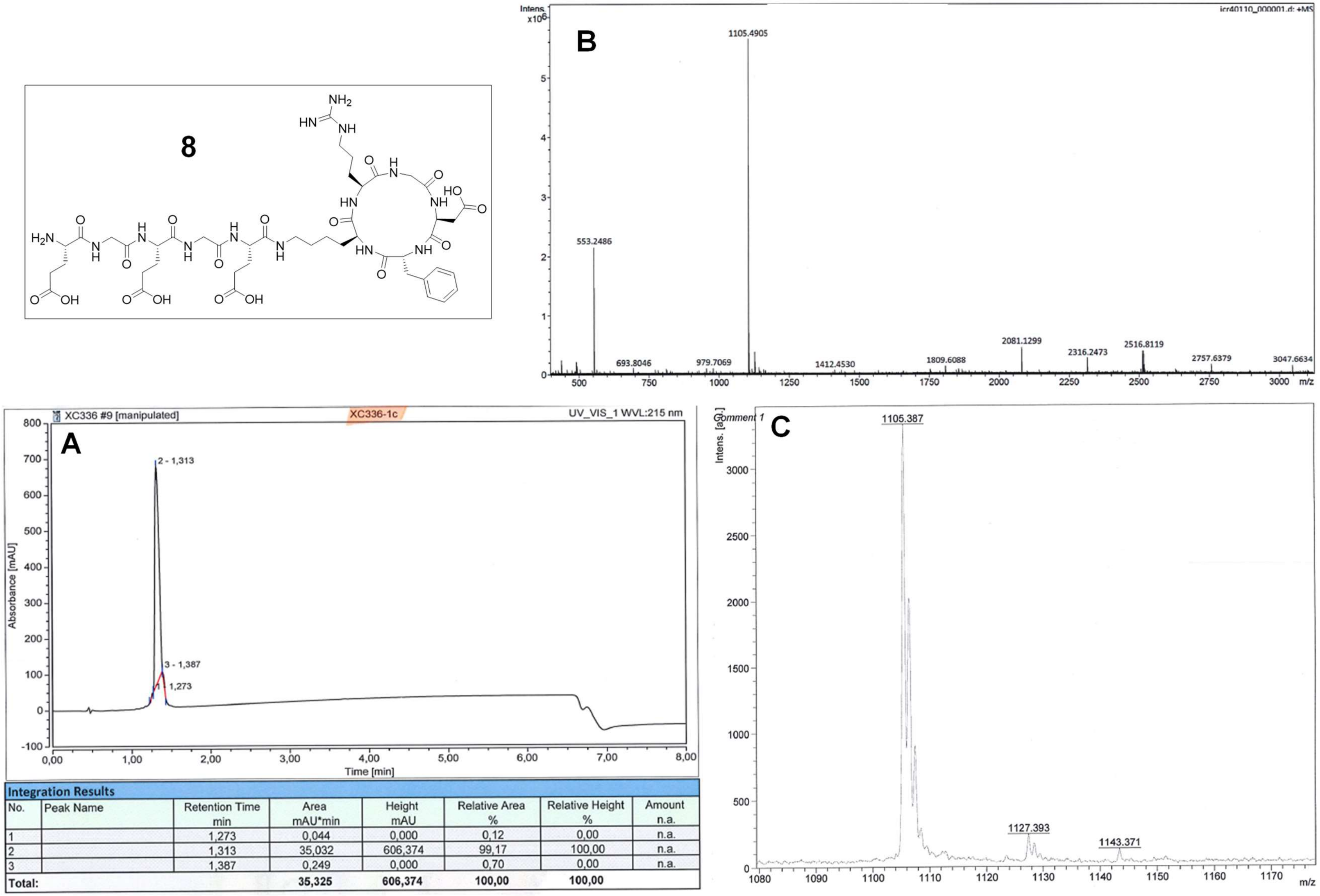

Integration Results

No.	Peak Name	Retention Time min	Area mAU*min	Height mAU	Relative Area %	Relative Height %	Amount n.a.
1		1,273	0,044	0,000	0,12	0,00	n.a.
2		1,313	35,032	606,374	99,17	100,00	n.a.
3		1,387	0,249	0,000	0,70	0,00	n.a.
Total:			35,325	606,374	100,00	100,00	

Figure S2. HPLC chromatogram (**A**) and mass spectra (**B**: ESI, **C**: MALDI) of c(RGDfK)-EGEGE (**8**).

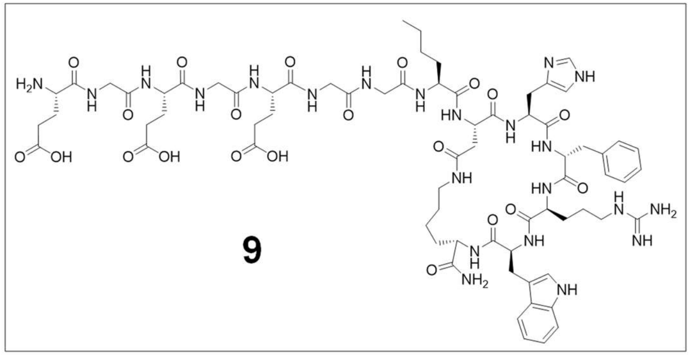

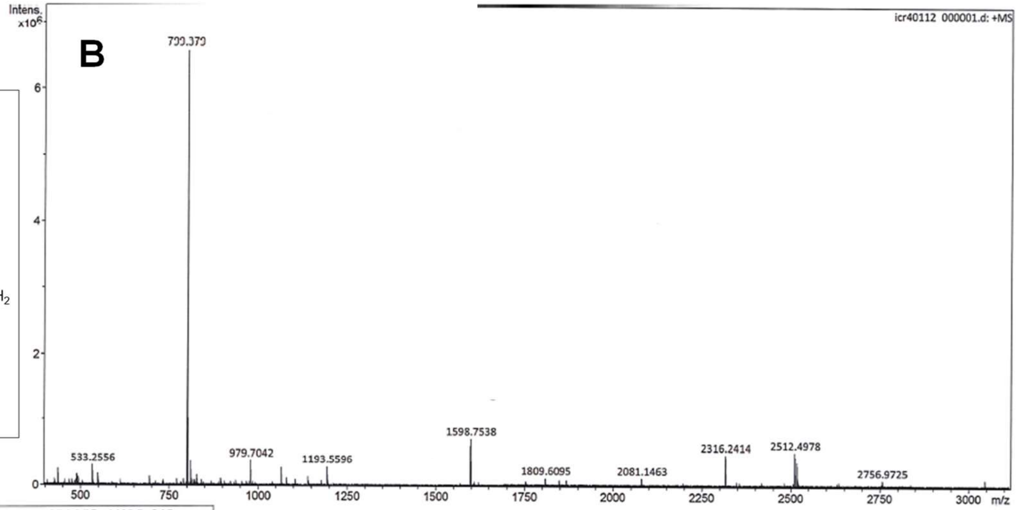

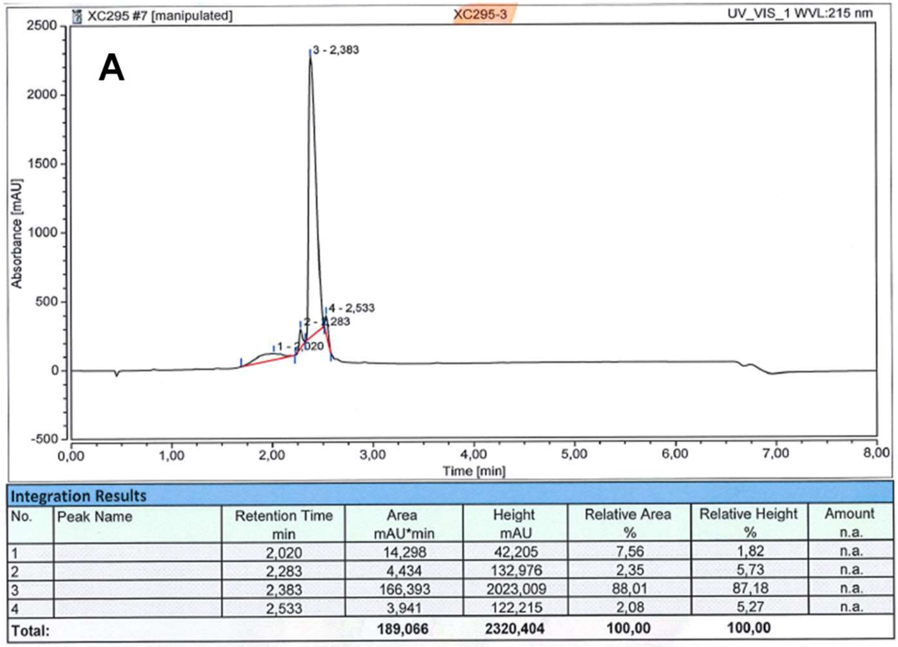

Integration Results

No.	Peak Name	Retention Time min	Area mAU*min	Height mAU	Relative Area %	Relative Height %	Amount n.a.
1		2,020	14,298	42,205	7,56	1,82	n.a.
2		2,283	4,434	132,976	2,35	5,73	n.a.
3		2,383	166,393	2023,009	88,01	87,18	n.a.
4		2,533	3,941	122,215	2,08	5,27	n.a.
Total:			189,066	2320,404	100,00	100,00	

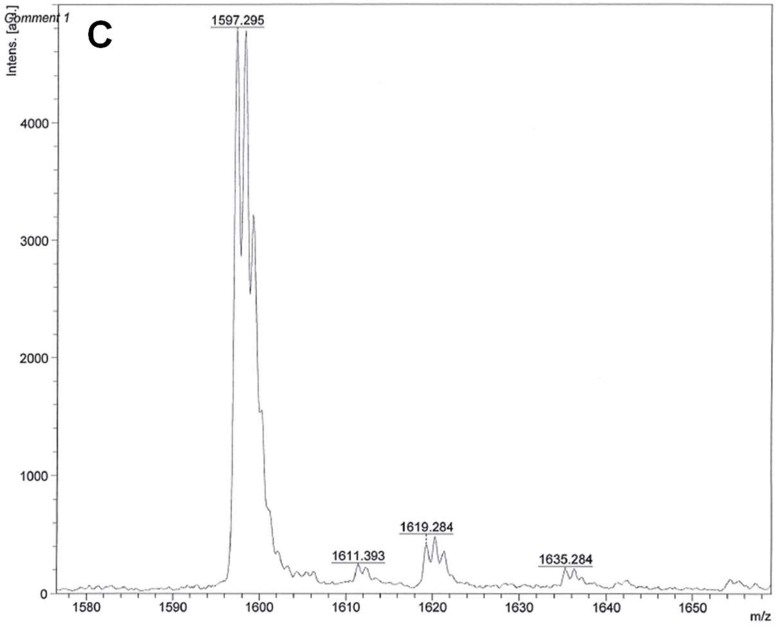

Figure S3. HPLC chromatogram (**A**) and mass spectra (**B**: ESI, **C**: MALDI) of EGEGE-GG-Nle-c(DHfRWK) (**9**).

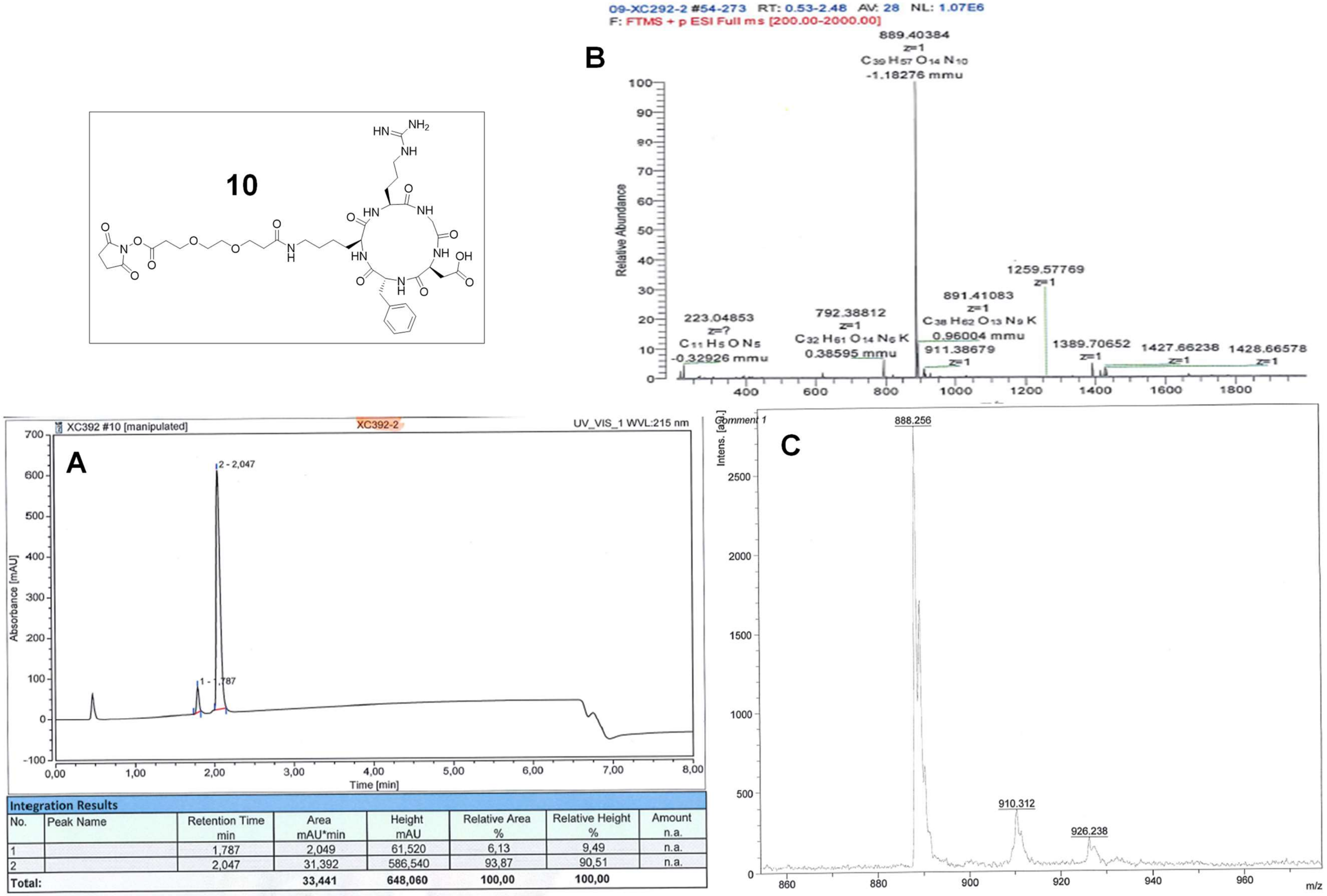

Integration Results

No.	Peak Name	Retention Time min	Area mAU*min	Height mAU	Relative Area %	Relative Height %	Amount n.a.
1		1,787	2,049	61,520	6,13	9,49	n.a.
2		2,047	31,392	586,540	93,87	90,51	n.a.
Total:			33,441	648,060	100,00	100,00	

Figure S4. HPLC chromatogram (**A**) and mass spectra (**B**: ESI, **C**: MALDI) of NHS-PEG$_1$-c(RGDfK) (**10**).

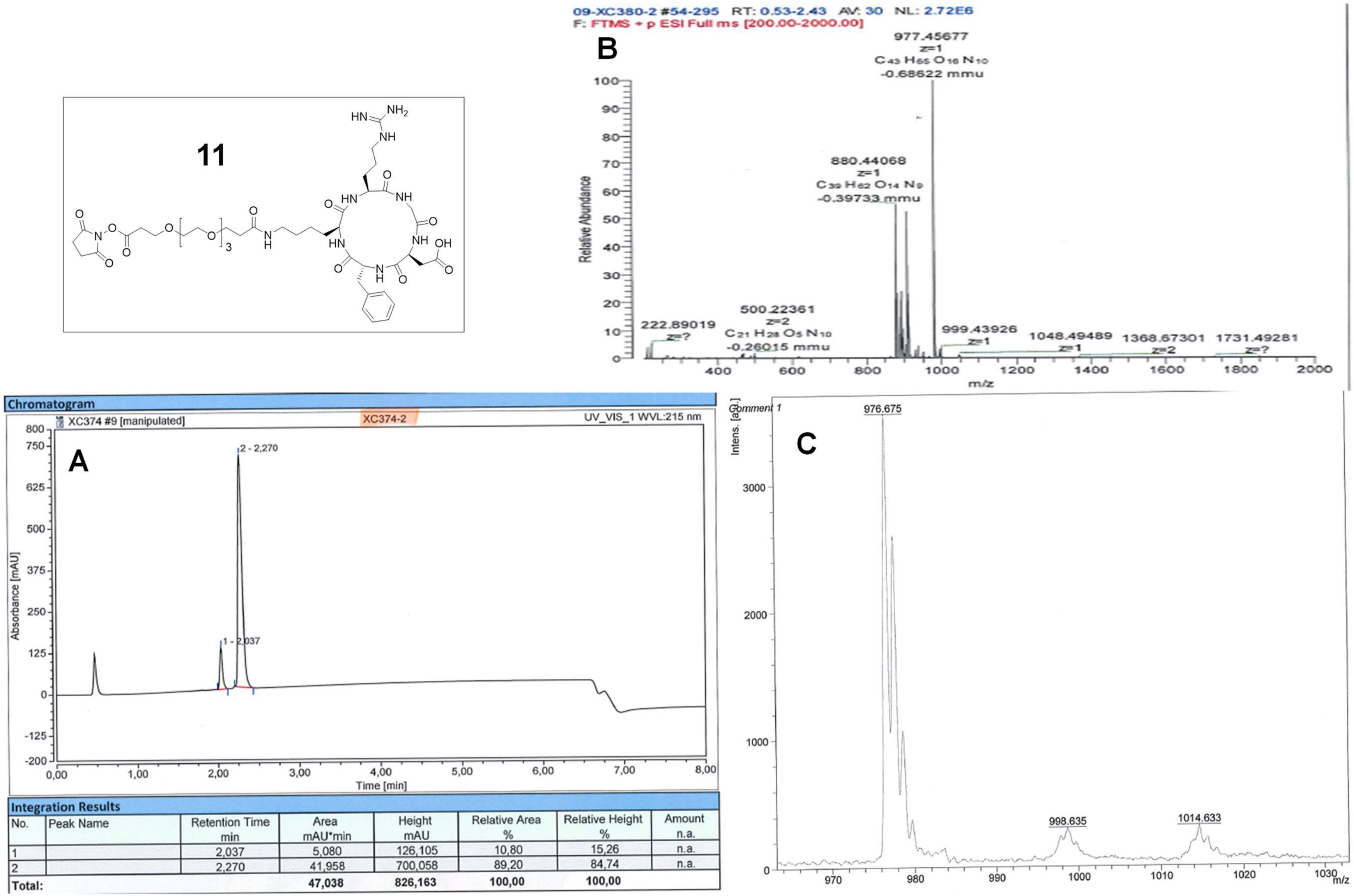

Integration Results

No.	Peak Name	Retention Time min	Area mAU*min	Height mAU	Relative Area %	Relative Height %	Amount n.a.
1		2,037	5,080	126,105	10,80	15,26	n.a.
2		2,270	41,958	700,058	89,20	84,74	n.a.
Total:			47,038	826,163	100,00	100,00	

Figure S5. HPLC chromatogram (**A**) and mass spectra (**B**: ESI, **C**: MALDI) of NHS-PEG$_3$-c(RGDfK) (**11**).

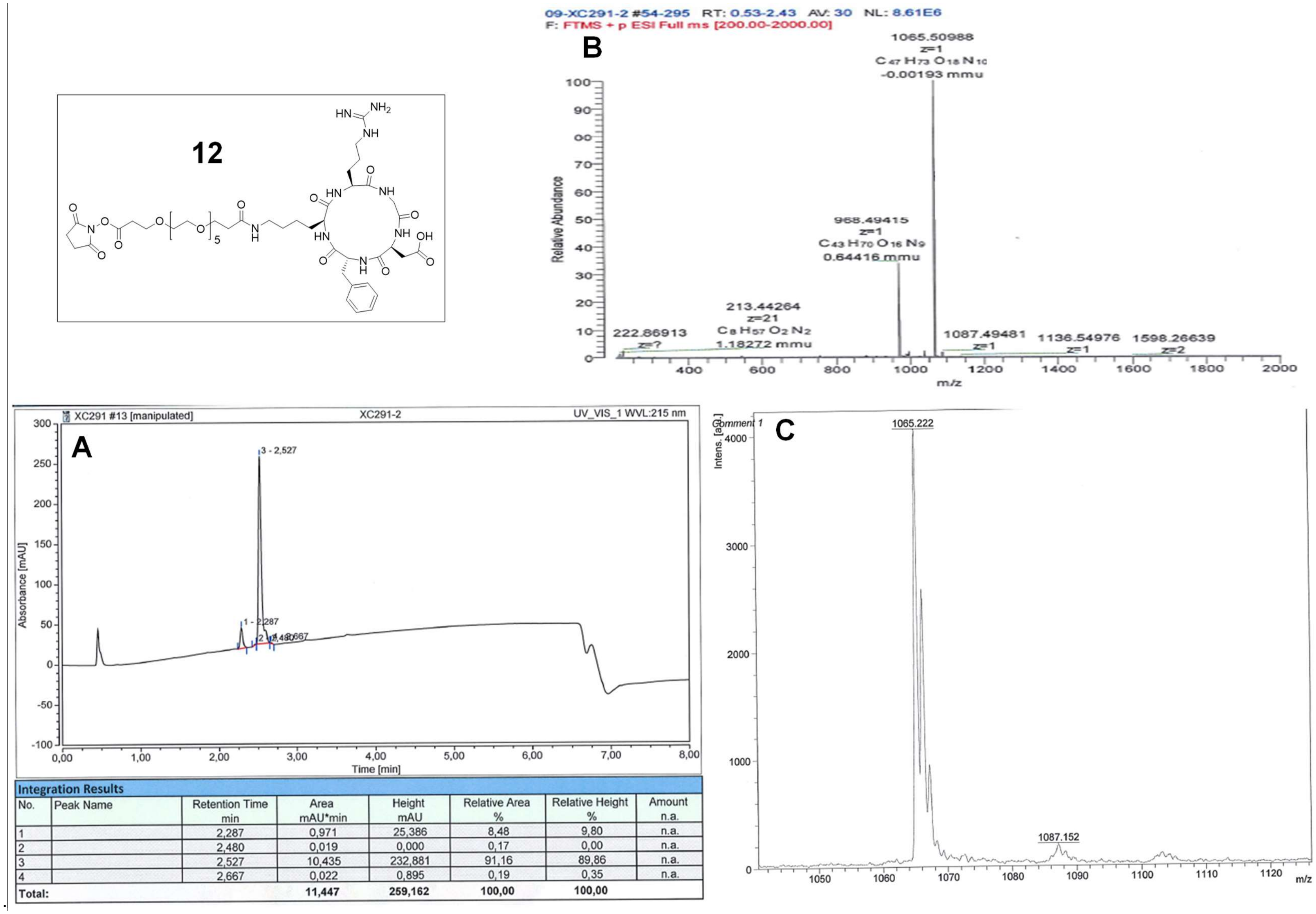

Integration Results

No.	Peak Name	Retention Time min	Area mAU*min	Height mAU	Relative Area %	Relative Height %	Amount n.a.
1		2,287	0,971	25,386	8,48	9,80	n.a.
2		2,480	0,019	0,000	0,17	0,00	n.a.
3		2,527	10,435	232,881	91,16	89,86	n.a.
4		2,667	0,022	0,895	0,19	0,35	n.a.
**Total:**			**11,447**	**259,162**	**100,00**	**100,00**	

Figure S6. HPLC chromatogram (**A**) and mass spectra (**B**: ESI, **C**: MALDI) of NHS-PEG$_5$-c(RGDfK) (**12**).

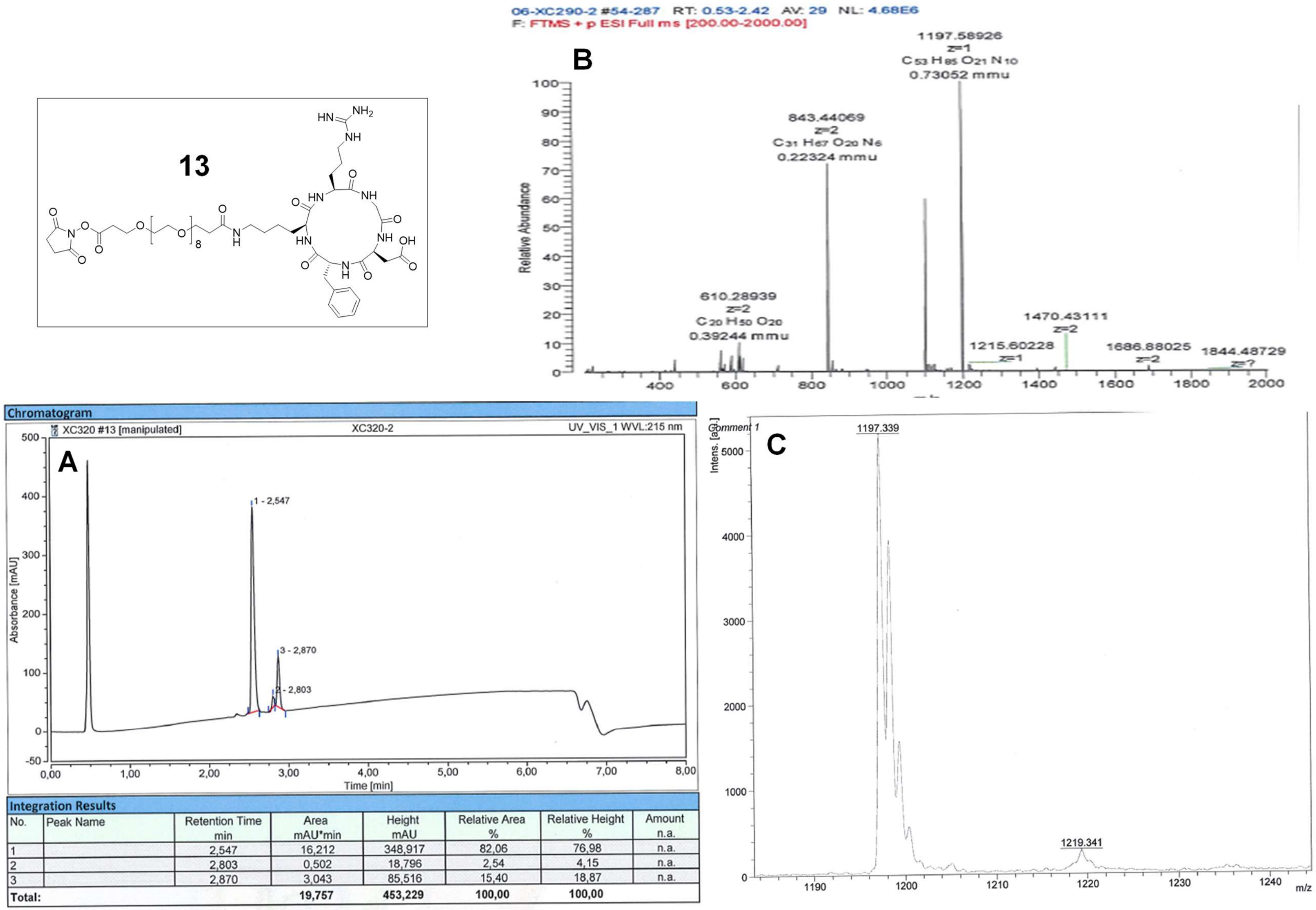

No.	Peak Name	Retention Time min	Area mAU*min	Height mAU	Relative Area %	Relative Height %	Amount n.a.
1		2,547	16,212	348,917	82,06	76,98	n.a.
2		2,803	0,502	18,796	2,54	4,15	n.a.
3		2,870	3,043	85,516	15,40	18,87	n.a.
Total:			19,757	453,229	100,00	100,00	

Figure S7. HPLC chromatogram (**A**) and mass spectra (**B**: ESI, **C**: MALDI) of NHS-PEG$_8$-c(RGDfK) (**13**).

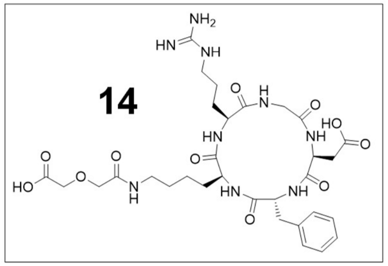

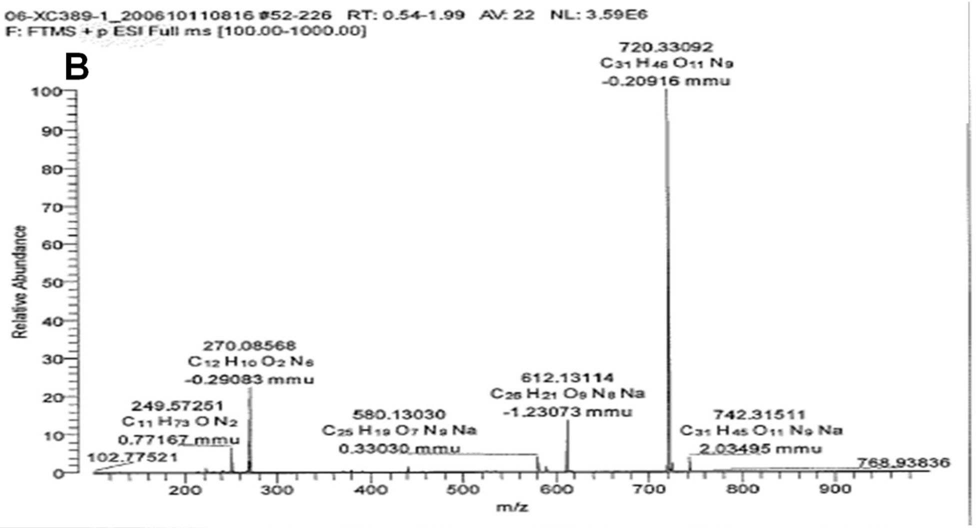

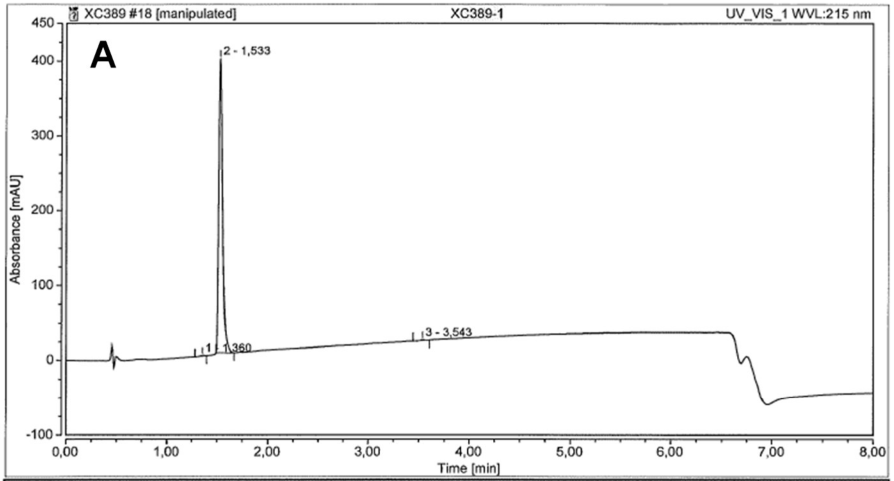

Integration Results

No.	Peak Name	Retention Time min	Area mAU*min	Height mAU	Relative Area %	Relative Height %	Amount n.a.
1		1,360	0,036	0,726	0,21	0,18	n.a.
2		1,533	17,231	392,723	99,67	99,68	n.a.
3		3,543	0,021	0,536	0,12	0,14	n.a.
**Total:**			**17,288**	**393,985**	**100,00**	**100,00**	

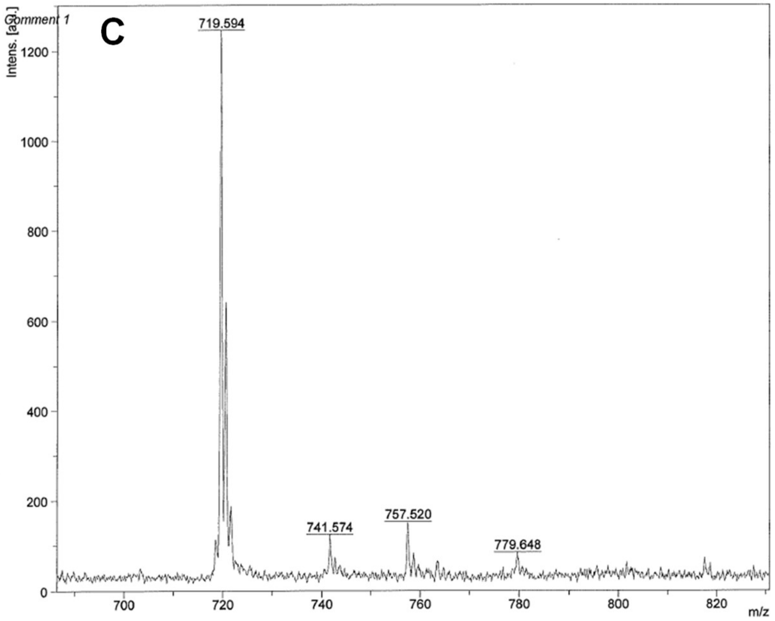

Figure S8. HPLC chromatogram (**A**) and mass spectra (**B**: ESI, **C**: MALDI) of HO-DIG-c(RGDfK) (**14**).

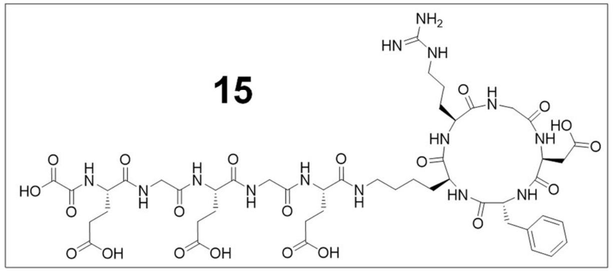

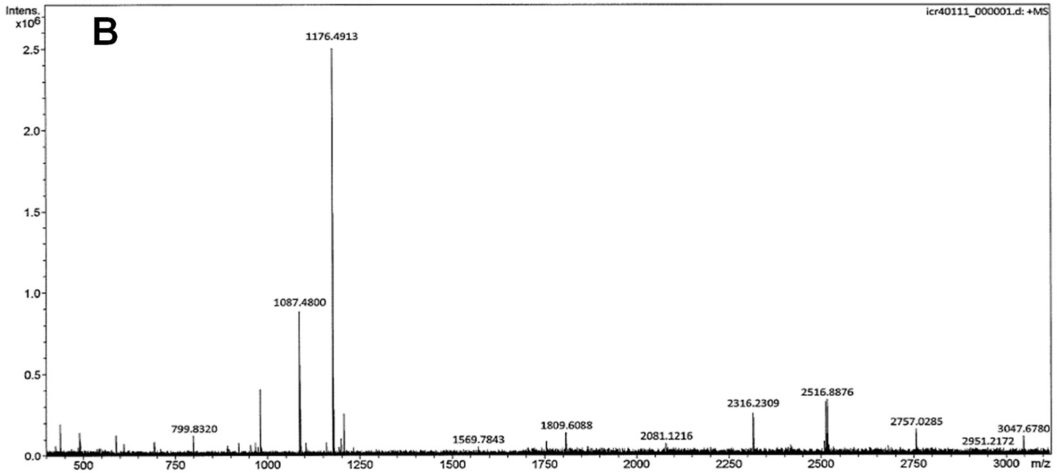

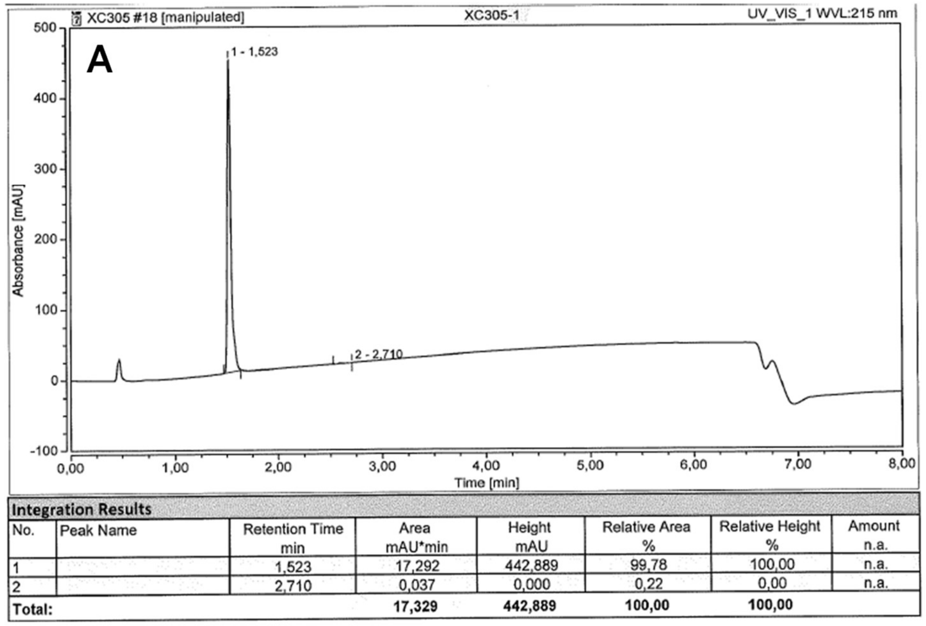

Integration Results							
No.	Peak Name	Retention Time min	Area mAU*min	Height mAU	Relative Area %	Relative Height %	Amount n.a.
1		1,523	17,292	442,889	99,78	100,00	n.a.
2		2,710	0,037	0,000	0,22	0,00	n.a.
**Total:**			**17,329**	**442,889**	**100,00**	**100,00**	

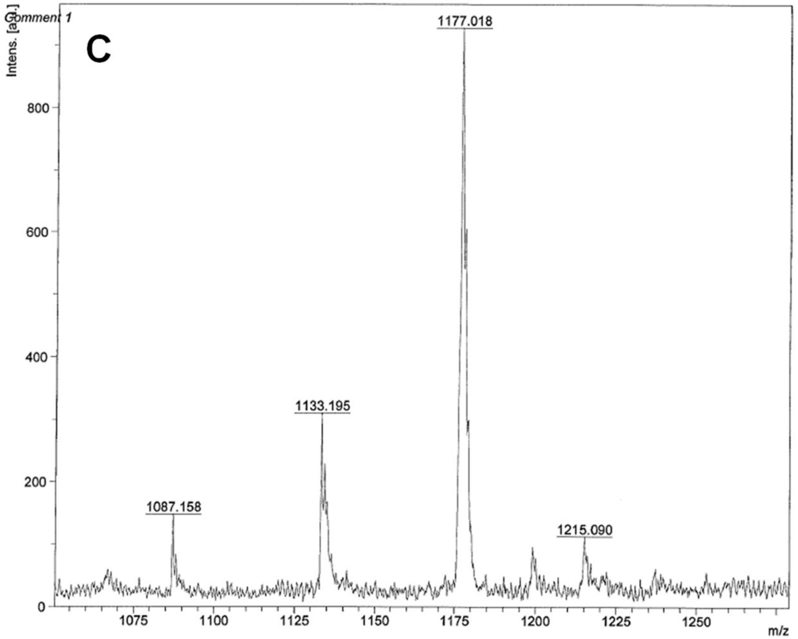

Figure S9. HPLC chromatogram (**A**) and mass spectra (**B**: ESI, **C**: MALDI) of HO-Ox-EGEGE-c(RGDfK) (**15**).

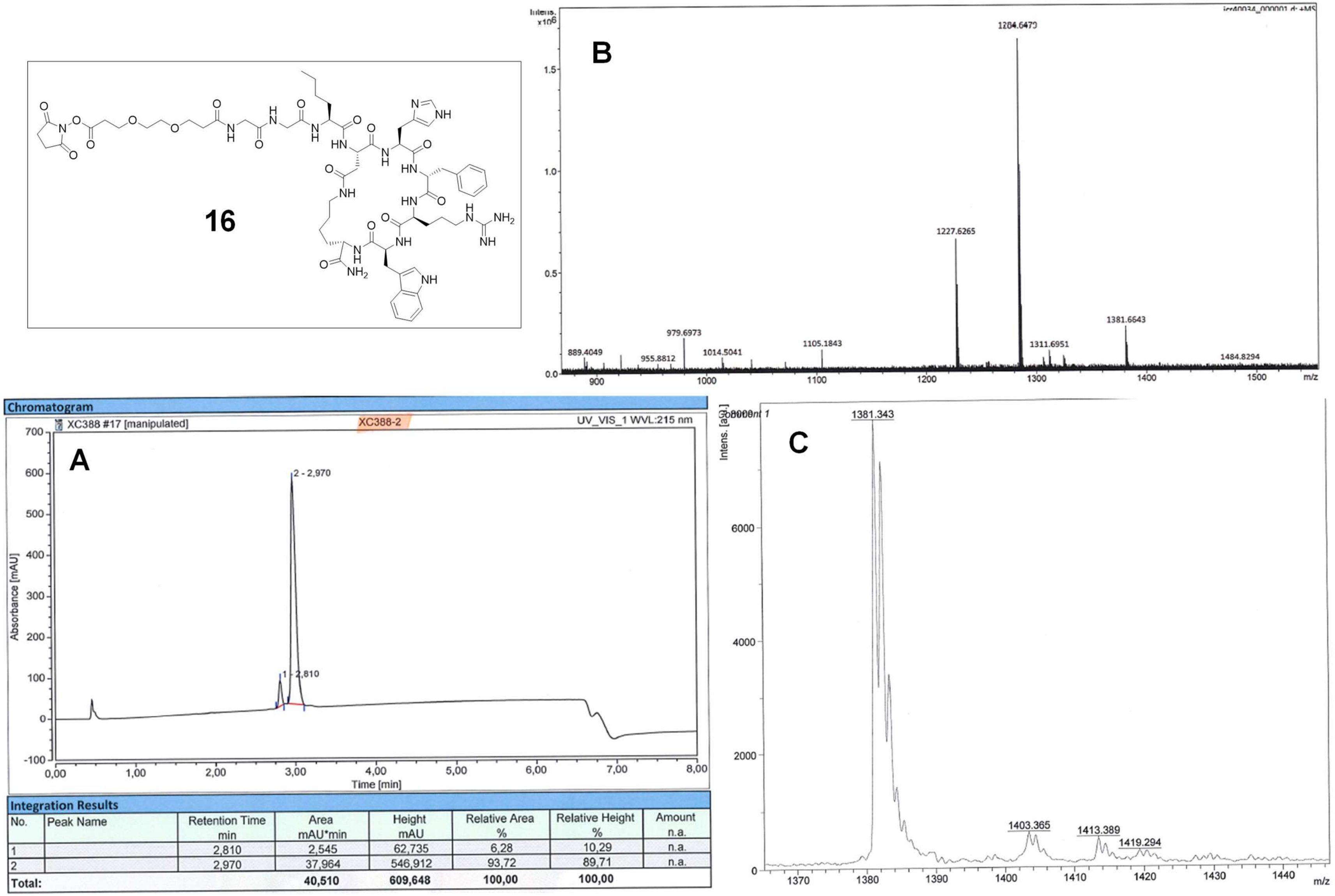

Integration Results

No.	Peak Name	Retention Time min	Area mAU*min	Height mAU	Relative Area %	Relative Height %	Amount n.a.
1		2,810	2,545	62,735	6,28	10,29	n.a.
2		2,970	37,964	546,912	93,72	89,71	n.a.
**Total:**			**40,510**	**609,648**	**100,00**	**100,00**	

Figure S10. HPLC chromatogram (**A**) and mass spectra (**B**: ESI, **C**: MALDI) of NHS-PEG$_1$-GG-Nle-c(DHfRWK) (**16**).

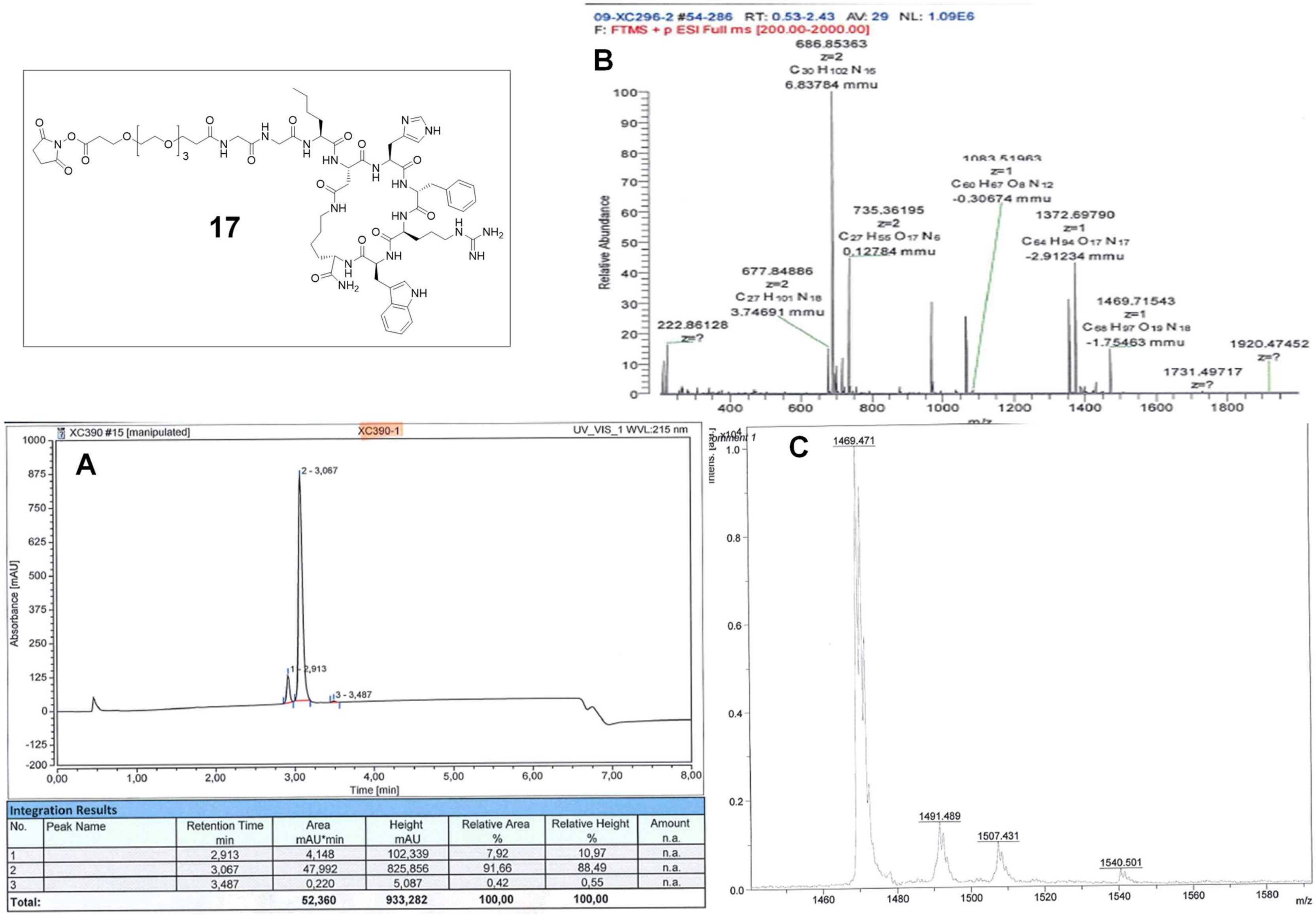

Integration Results							
No.	Peak Name	Retention Time min	Area mAU*min	Height mAU	Relative Area %	Relative Height %	Amount n.a.
1		2,913	4,148	102,339	7,92	10,97	n.a.
2		3,067	47,992	825,856	91,66	88,49	n.a.
3		3,487	0,220	5,087	0,42	0,55	n.a.
Total:			52,360	933,282	100,00	100,00	

Figure S11. HPLC chromatogram (**A**) and mass spectra (**B**: ESI, **C**: MALDI) of NHS-PEG3-GG-Nle-c(DHfRWK) (**17**).

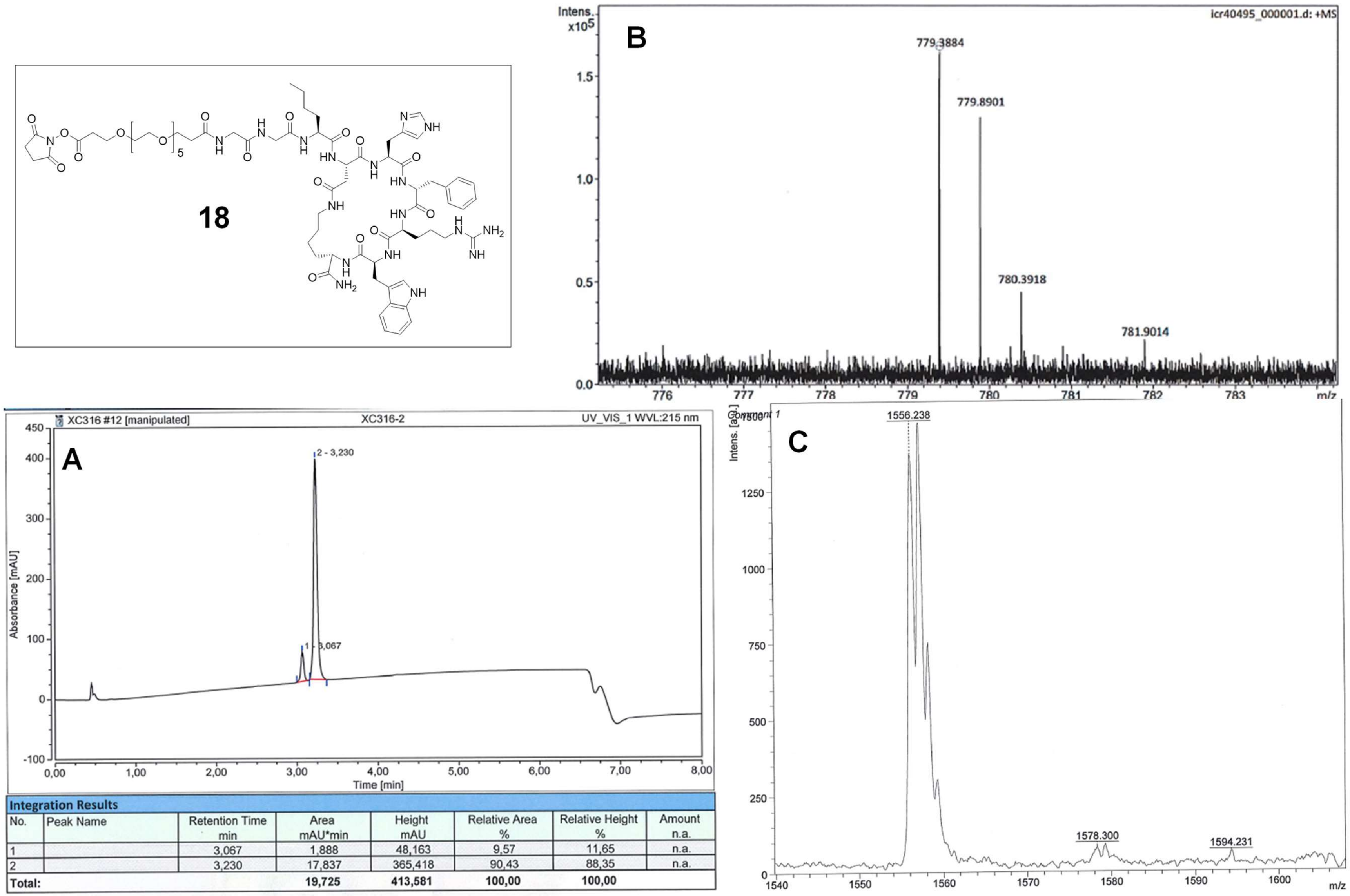

Integration Results

No.	Peak Name	Retention Time min	Area mAU*min	Height mAU	Relative Area %	Relative Height %	Amount n.a.
1		3,067	1,888	48,163	9,57	11,65	n.a.
2		3,230	17,837	365,418	90,43	88,35	n.a.
**Total:**			**19,725**	**413,581**	**100,00**	**100,00**	

Figure S12. HPLC chromatogram (**A**) and mass spectra (**B**: ESI, **C**: MALDI) of NHS-PEG$_5$-GG-Nle-c(DHfRWK) (**18**).

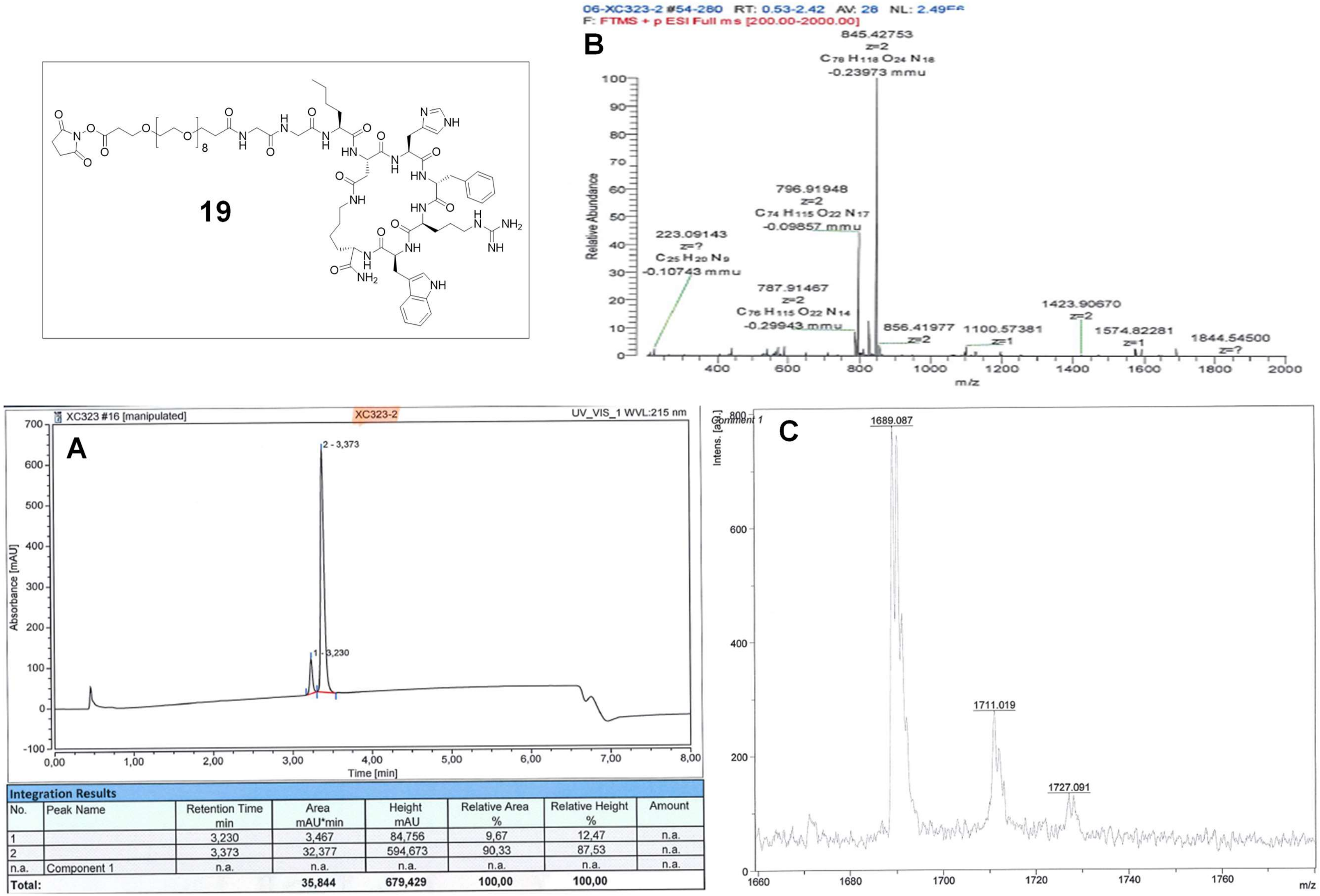

Integration Results

No.	Peak Name	Retention Time min	Area mAU*min	Height mAU	Relative Area %	Relative Height %	Amount
1		3,230	3,467	84,756	9,67	12,47	n.a.
2		3,373	32,377	594,673	90,33	87,53	n.a.
n.a.	Component 1	n.a.	n.a.	n.a.	n.a.	n.a.	n.a.
**Total:**			**35,844**	**679,429**	**100,00**	**100,00**	

Figure S13. HPLC chromatogram (**A**) and mass spectra (**B**: ESI, **C**: MALDI) of NHS-PEG$_8$-GG-Nle-c(DHfRWK) (**19**).

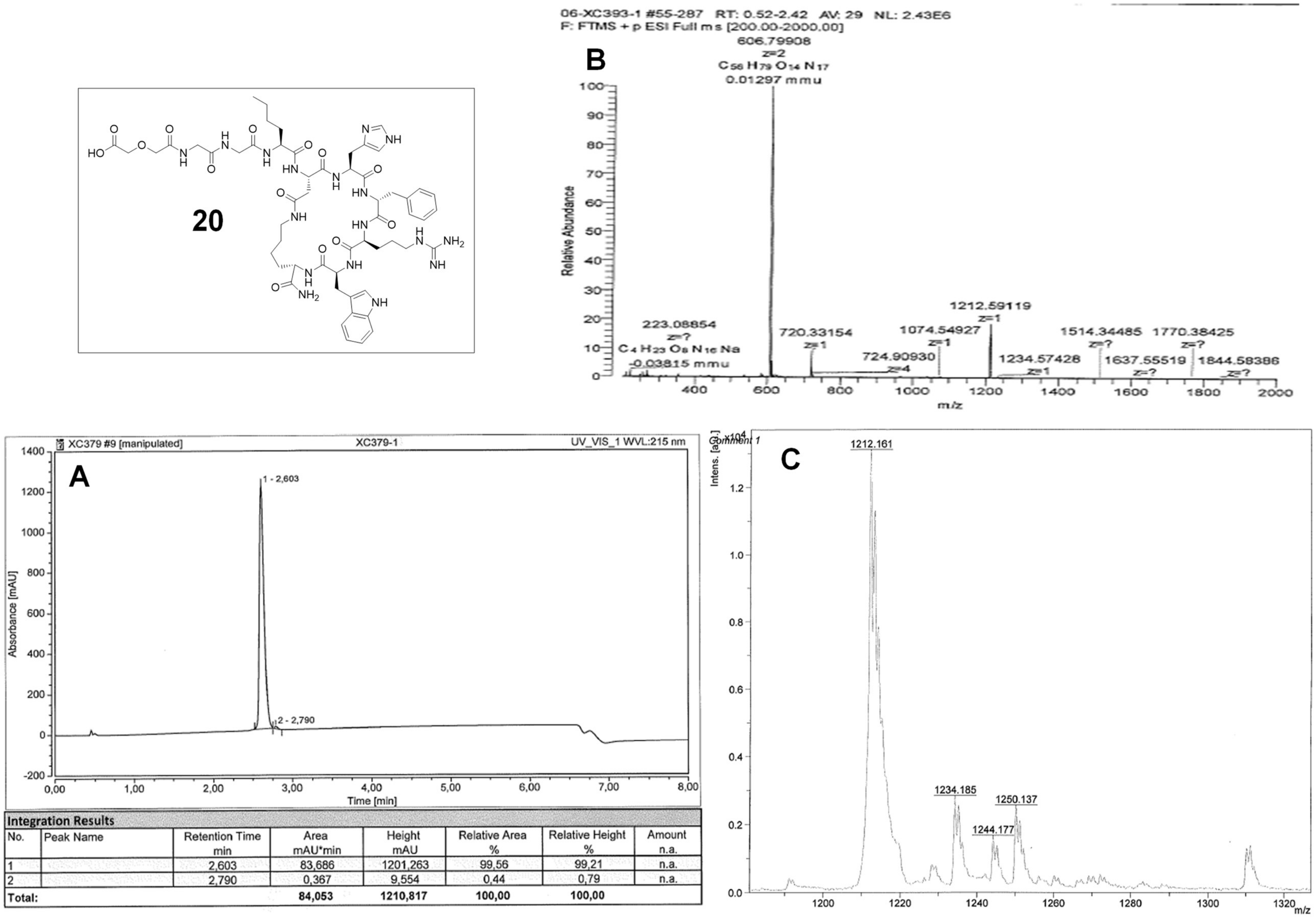

Integration Results							
No.	Peak Name	Retention Time min	Area mAU*min	Height mAU	Relative Area %	Relative Height %	Amount n.a.
1		2,603	83,686	1201,263	99,56	99,21	n.a.
2		2,790	0,367	9,554	0,44	0,79	n.a.
Total:			84,053	1210,817	100,00	100,00	

Figure S14. HPLC chromatogram (**A**) and mass spectra (**B**: ESI, **C**: MALDI) of HO-DIG-GG-Nle-c(DHfRWK) (**20**).

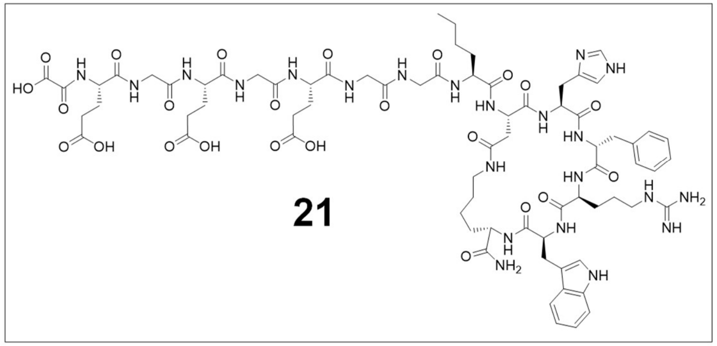

21

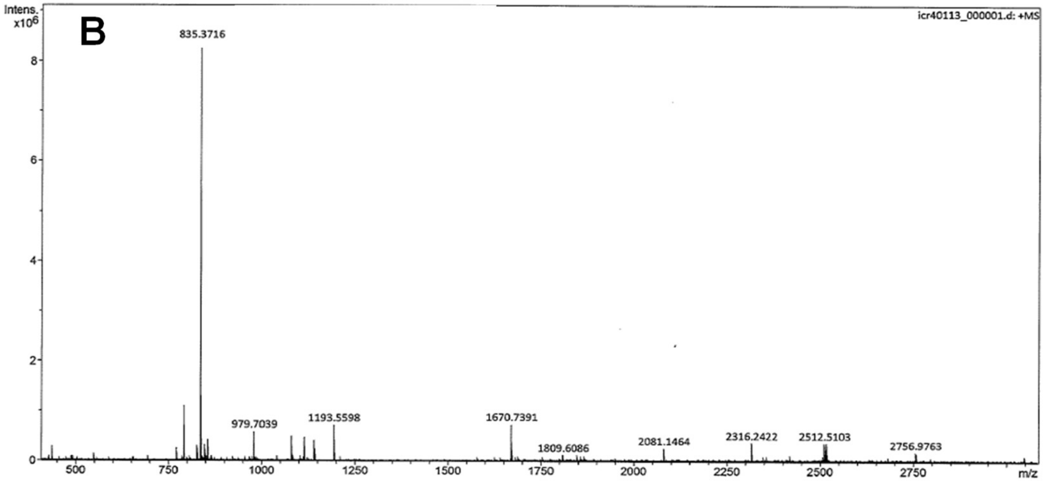

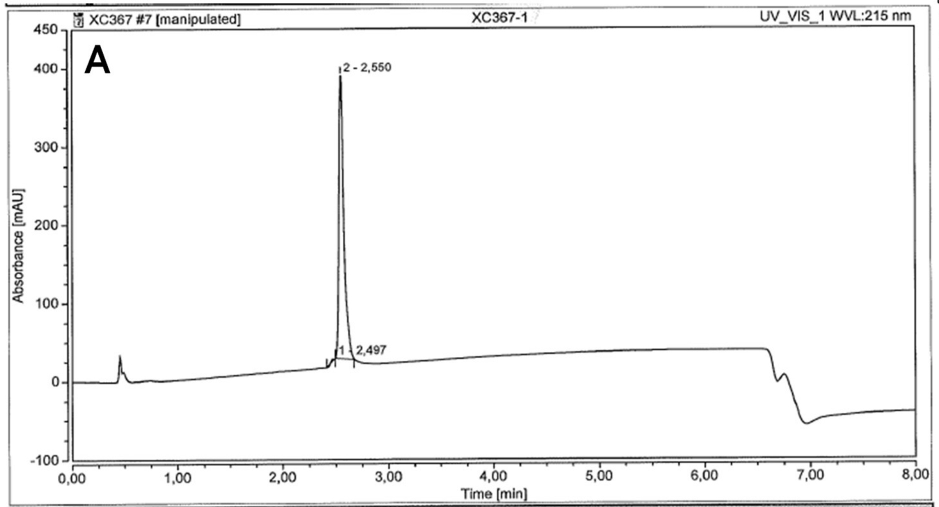

Integration Results

No.	Peak Name	Retention Time min	Area mAU*min	Height mAU	Relative Area %	Relative Height %	Amount n.a.
1		2,497	0,039	0,000	0,21	0,00	n.a.
2		2,550	18,969	361,396	99,79	100,00	n.a.
**Total:**			**19,008**	**361,396**	**100,00**	**100,00**	

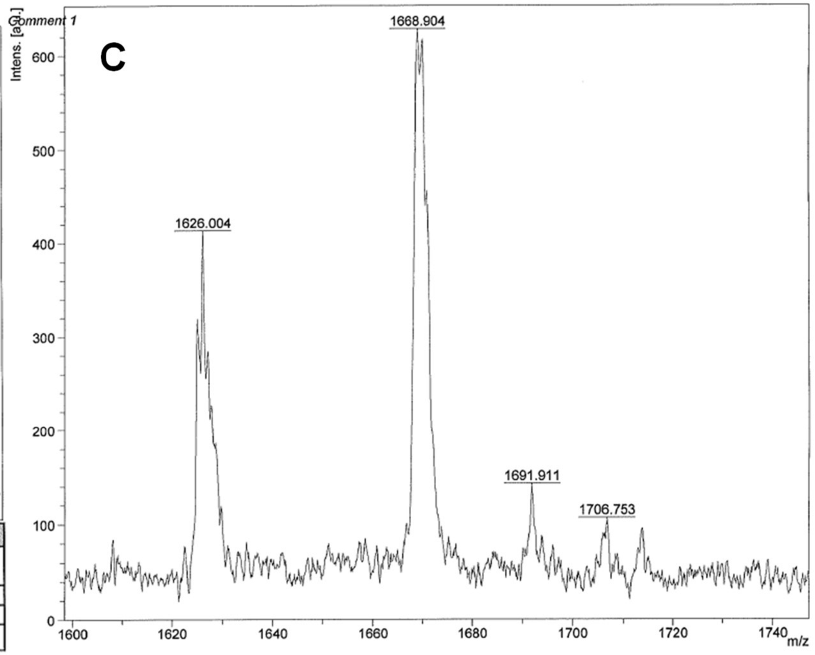

Figure S15. HPLC chromatogram (**A**) and mass spectra (**B**: ESI, **C**: MALDI) of HO-Ox-EGEGE-GG-Nle-c(DHfRWK) (**21**).

2. NMR and mass spectra of SiFA*lin* 28, SiFA*lin*-modified framework 29 and their intermediates 22–27

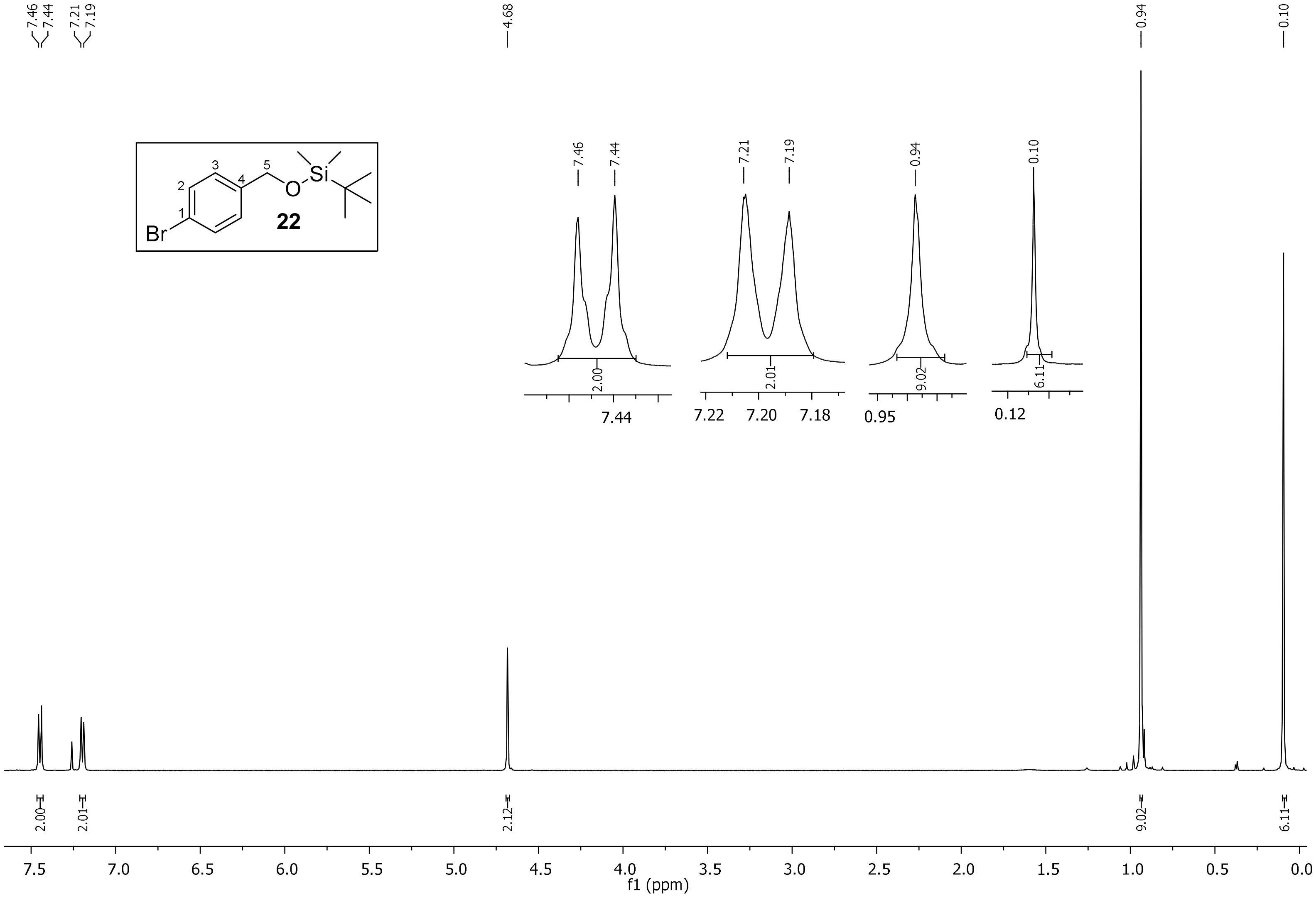

Figure S16: ^{1}H NMR spectrum of ((4-Bromobenzyl)oxy)(*tert*-butyl)dimethylsilane (**22**).

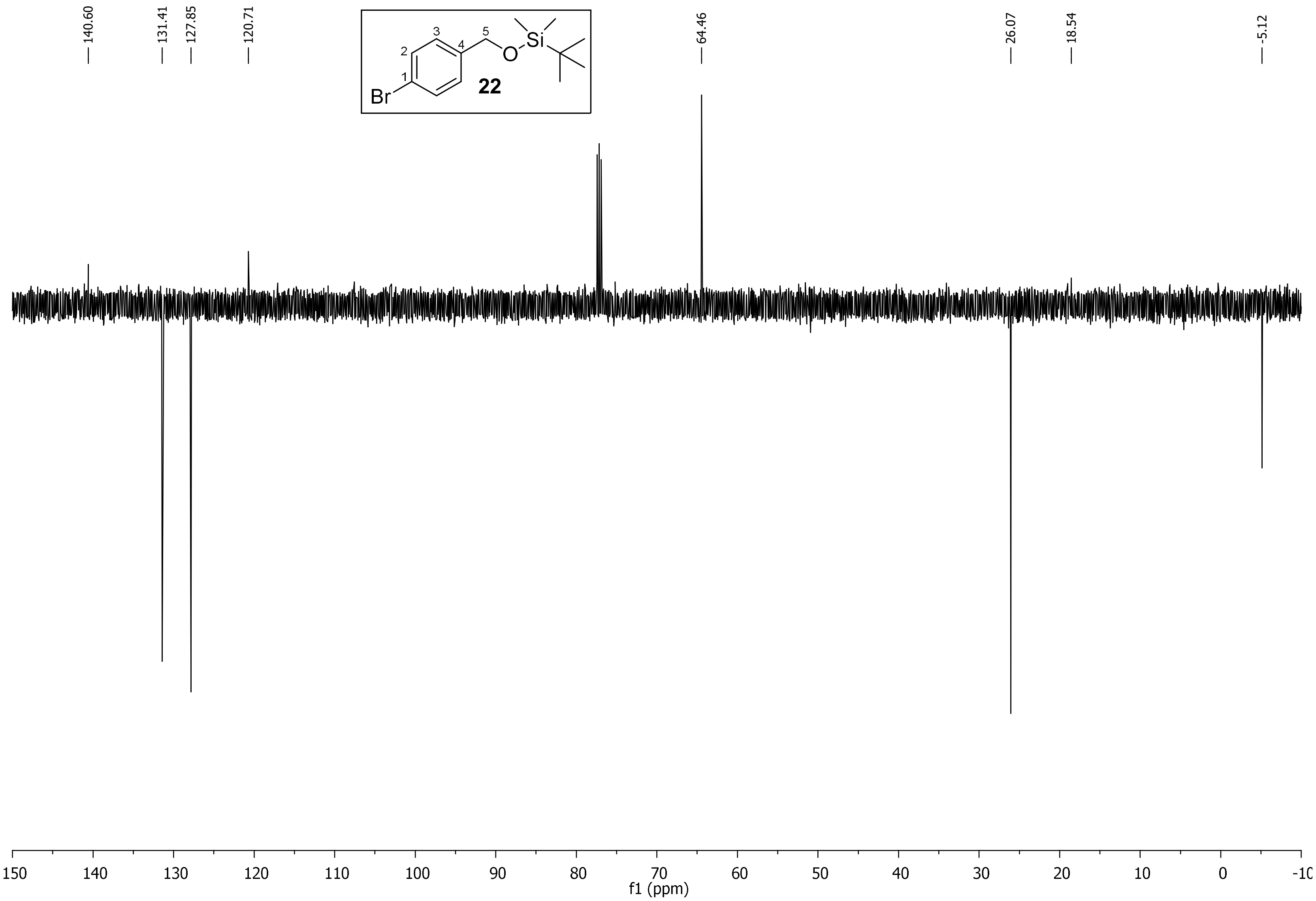

Figure S17. ^{13}C NMR spectrum of ((4-Bromobenzyl)oxy)(*tert*-butyl)dimethylsilane (**22**).

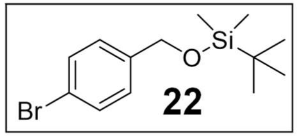

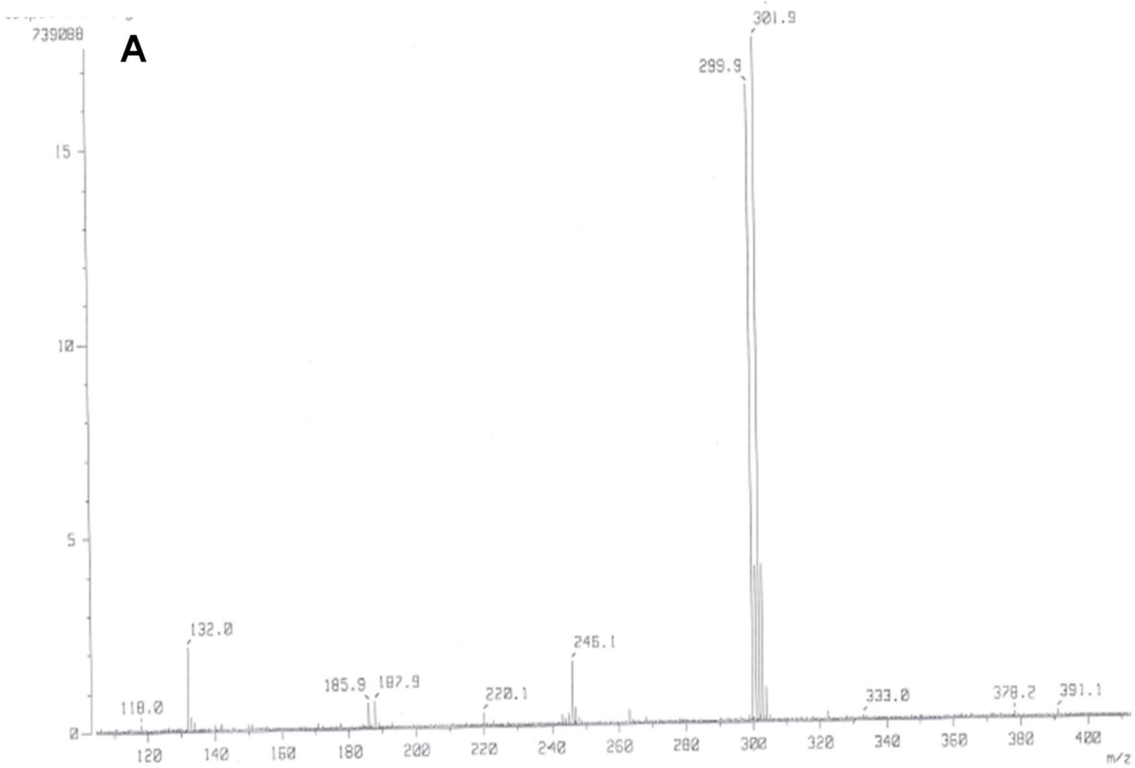

Figure S18. Mass spectrum (**A**: FI) of ((4-Bromobenzyl)oxy)(*tert*-butyl)dimethylsilane (**22**).

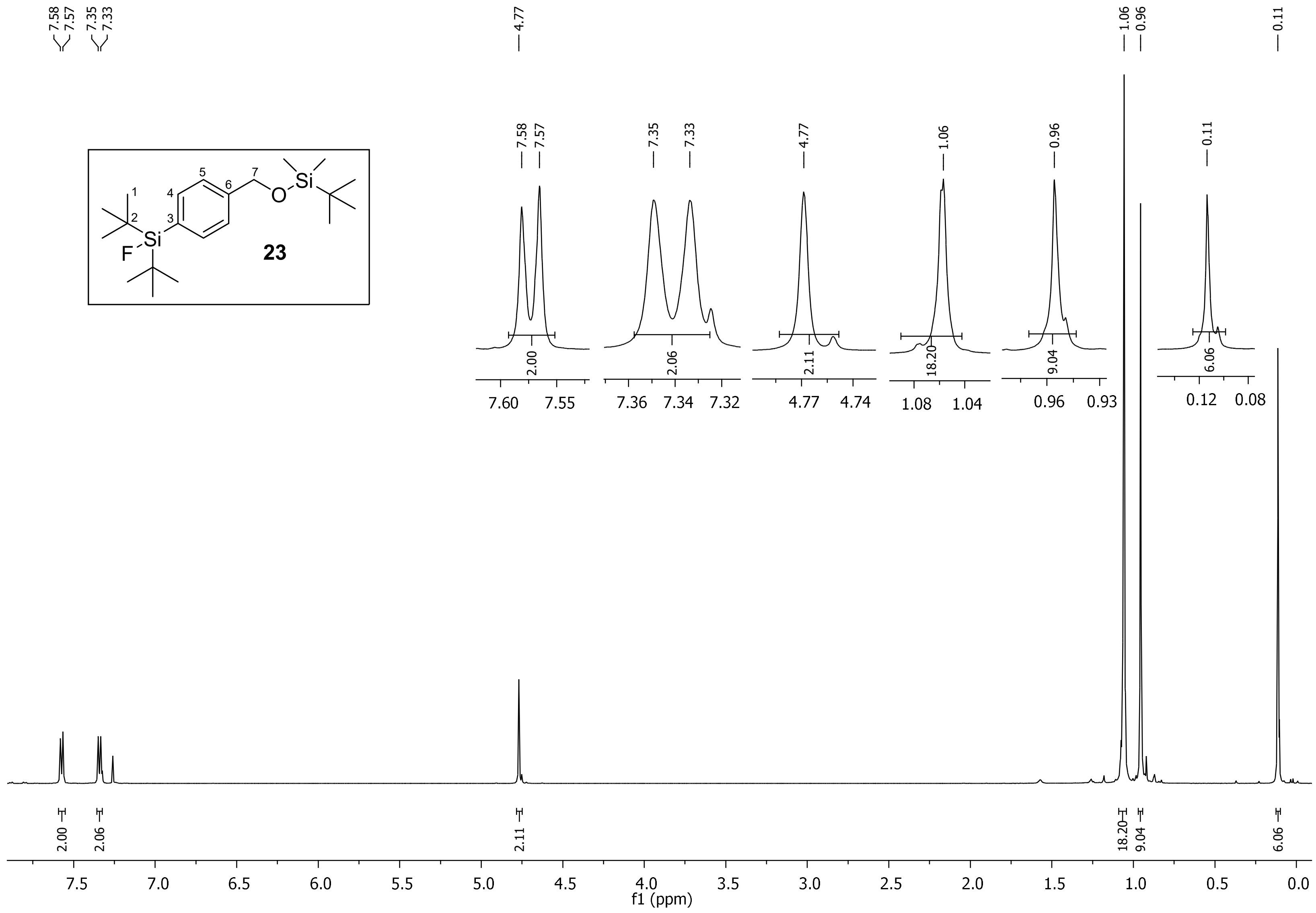

Figure S19. ^{1}H NMR spectrum of Di-*tert*-butyl(4-(((*tert*-butyldimethylsilyl)oxy)methyl)phenyl)fluorosilane (**23**).

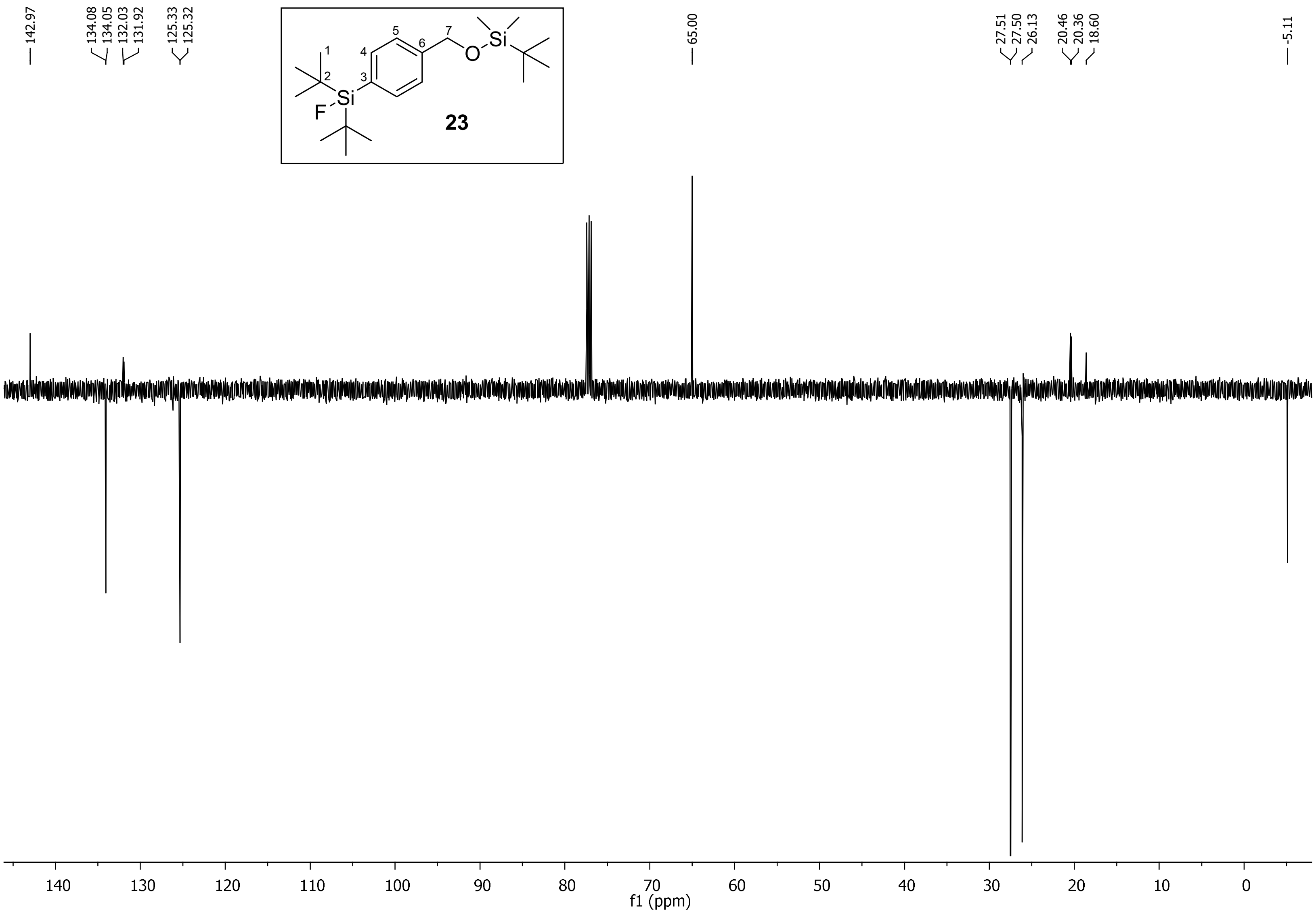

Figure S20. ^{13}C NMR spectrum of Di-*tert*-butyl(4-(((*tert*-butyldimethylsilyl)oxy)methyl)phenyl)fluorosilane (**23**).

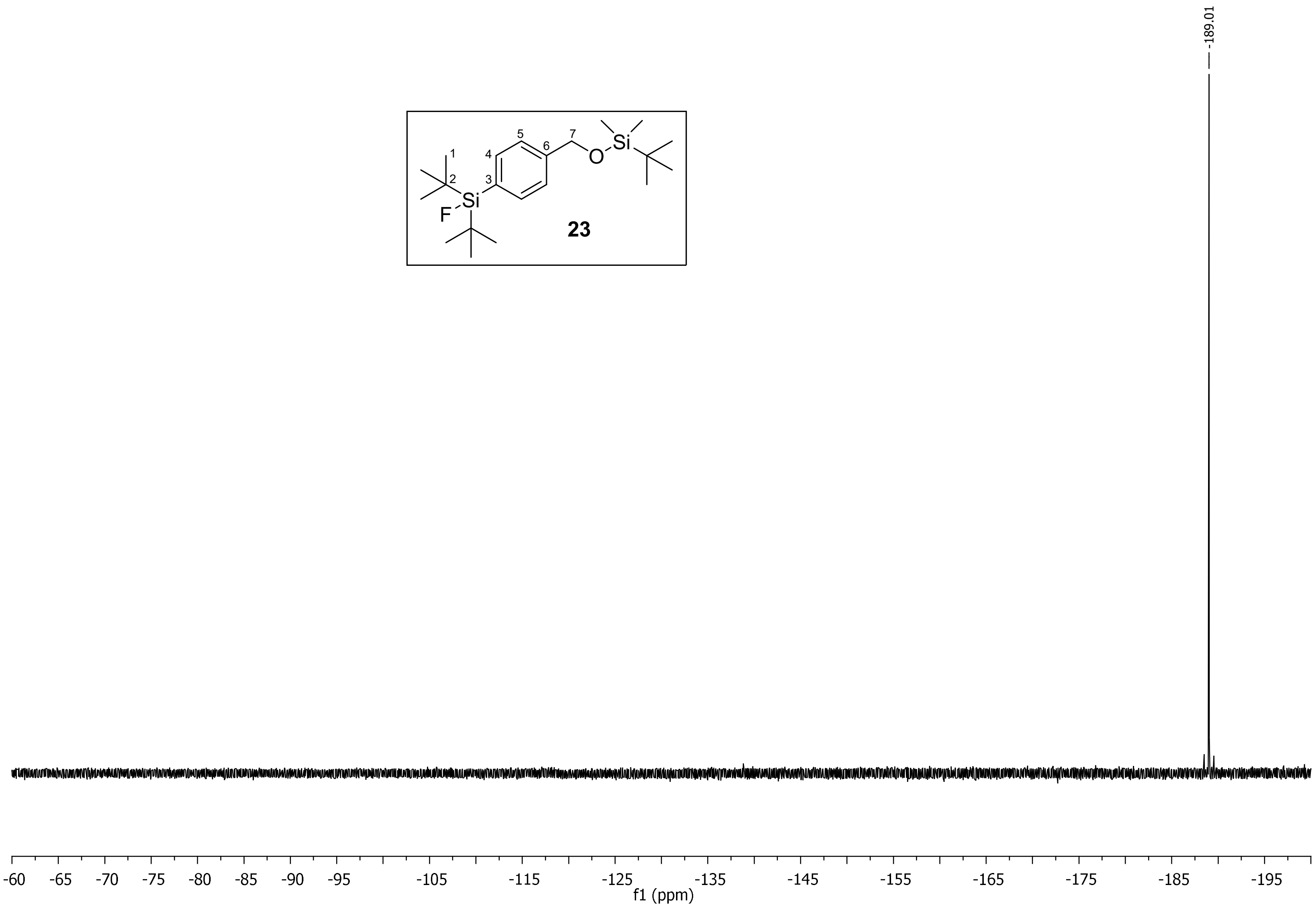

Figure S21. ^{19}F NMR spectrum of Di-*tert*-butyl(4-(((*tert*-butyldimethylsilyl)oxy)methyl)phenyl)fluorosilane (**23**).

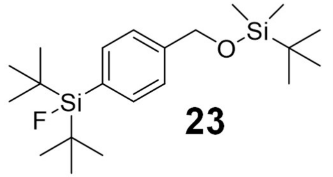

23

A

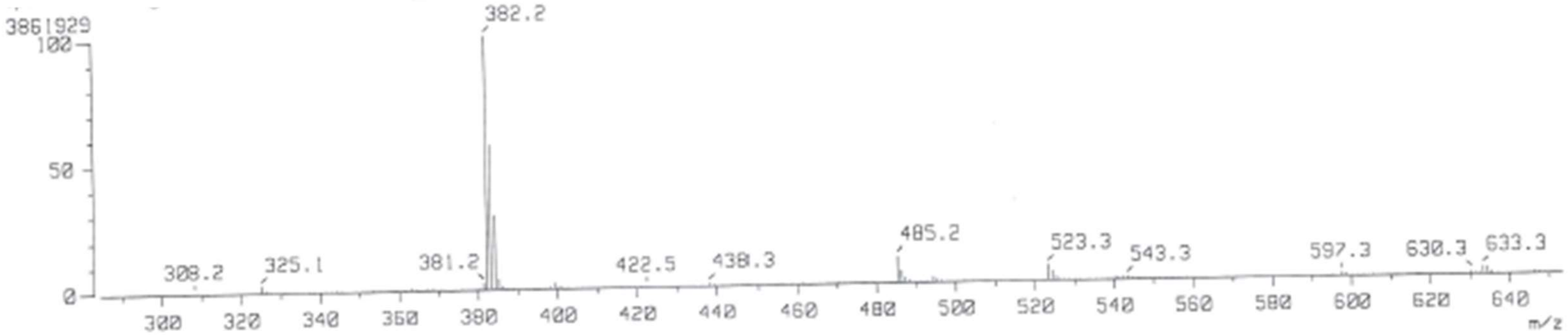

Figure S22. Mass spectrum (**A**: FD) of Di-*tert*-butyl(4-(((*tert*-butyldimethylsilyl)oxy)methyl)phenyl)fluorosilane (**23**).

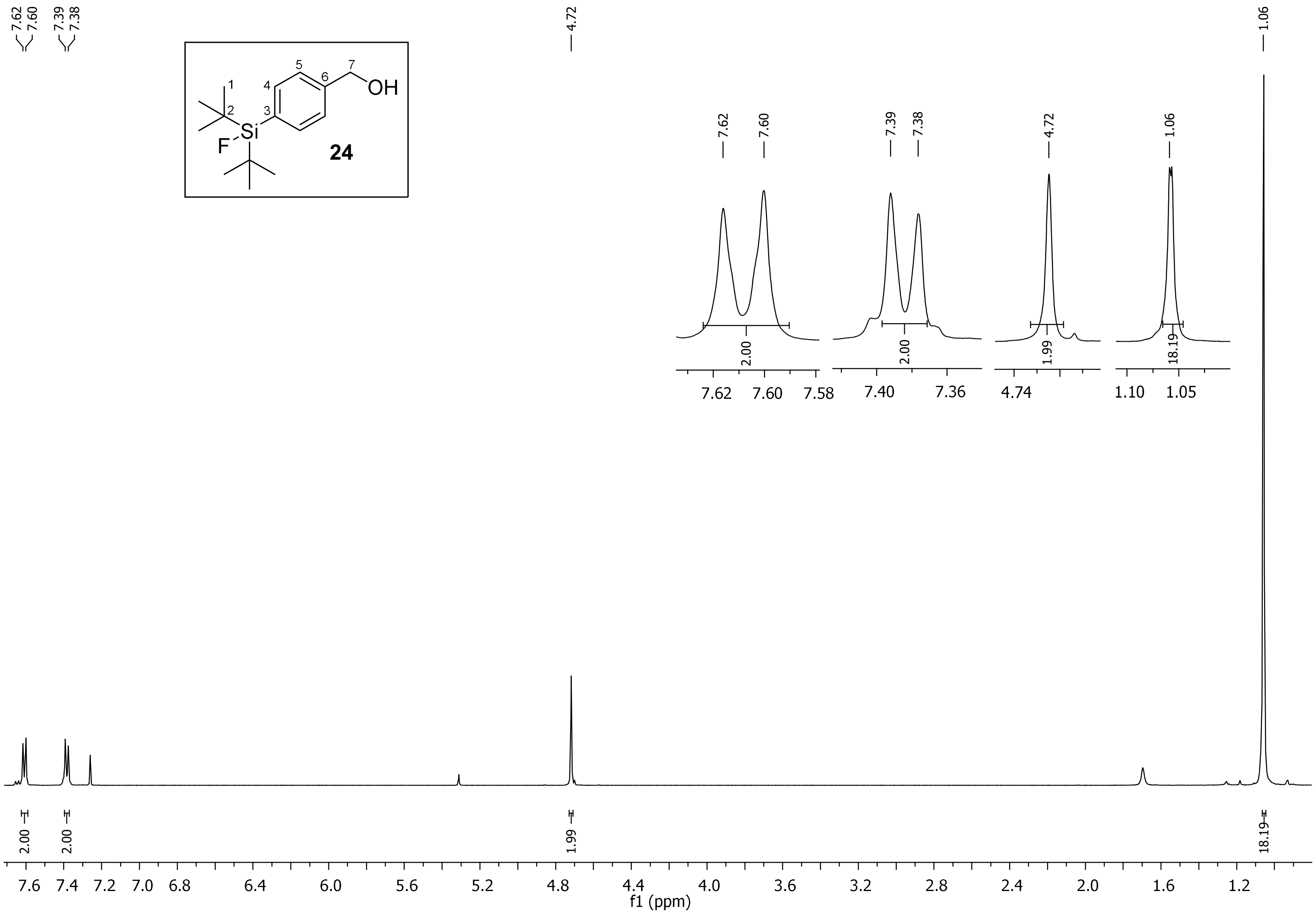

Figure S23. ^{1}H NMR spectrum of (4-(Di-*tert*-butylfluorosilyl)phenyl)methanol (**24**).

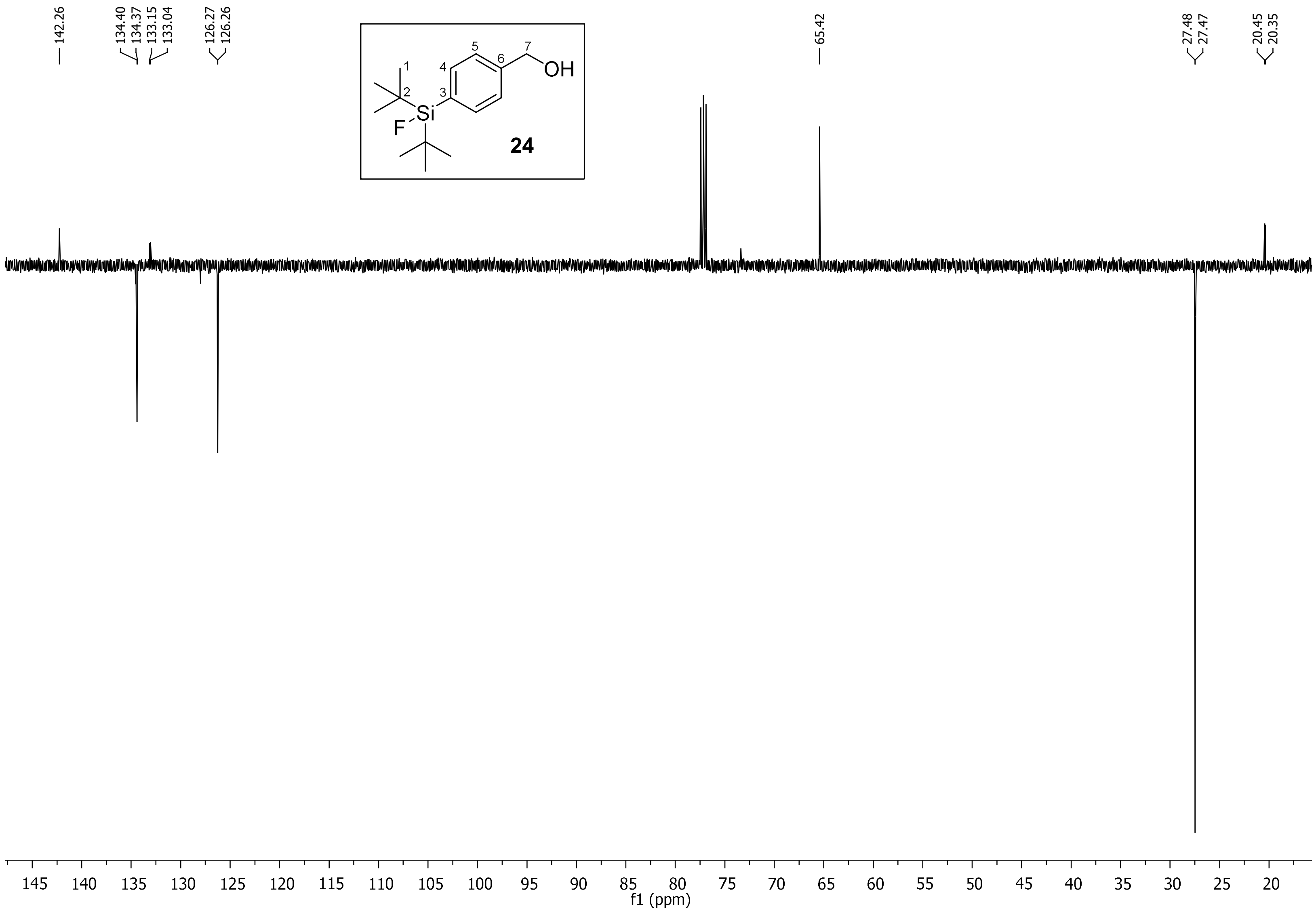

Figure S24. ^{13}C NMR spectrum of (4-(Di-*tert*-butylfluorosilyl)phenyl)methanol (**24**).

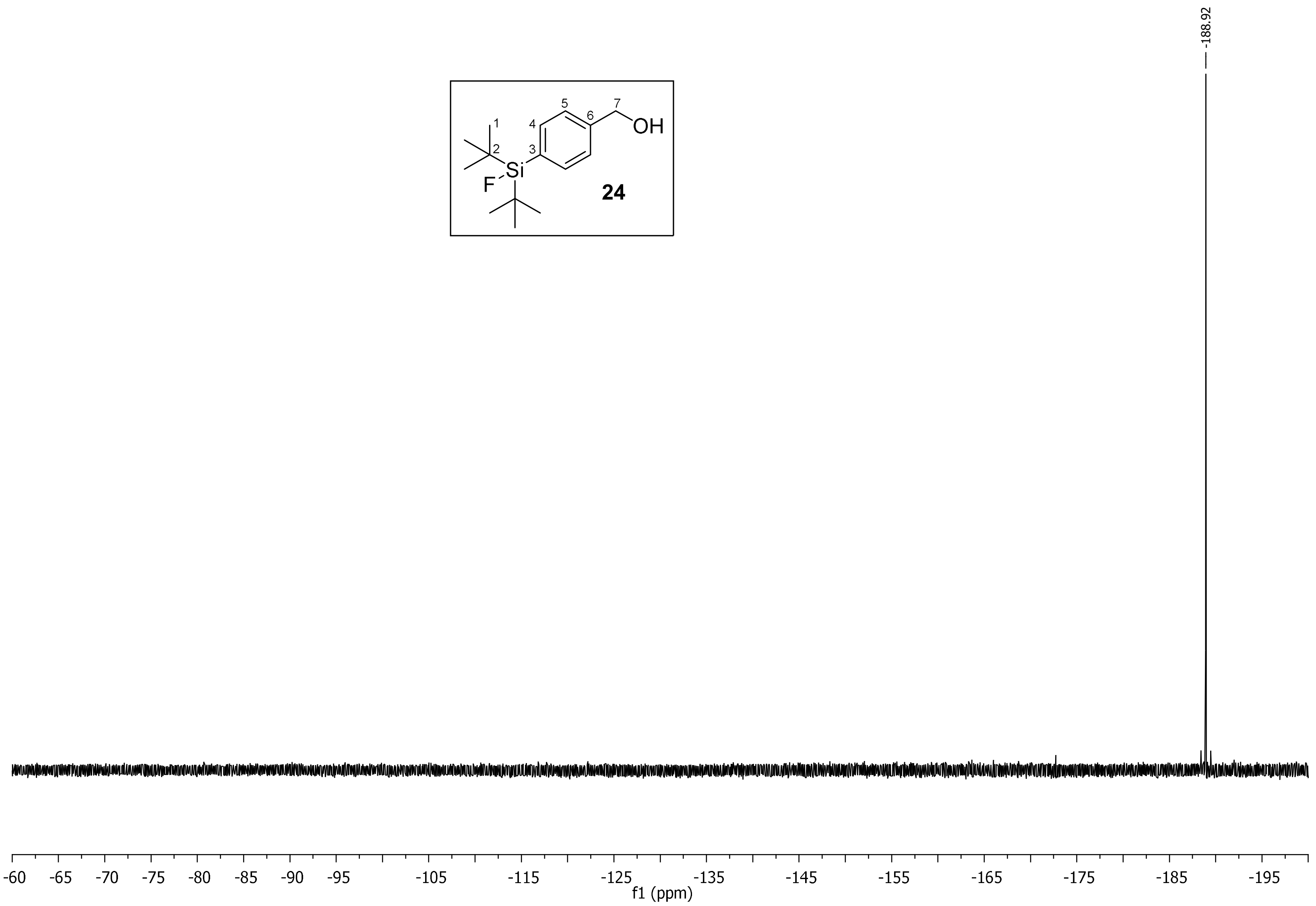

Figure S25. ^{19}F NMR spectrum of (4-(Di-*tert*-butylfluorosilyl)phenyl)methanol (**24**).

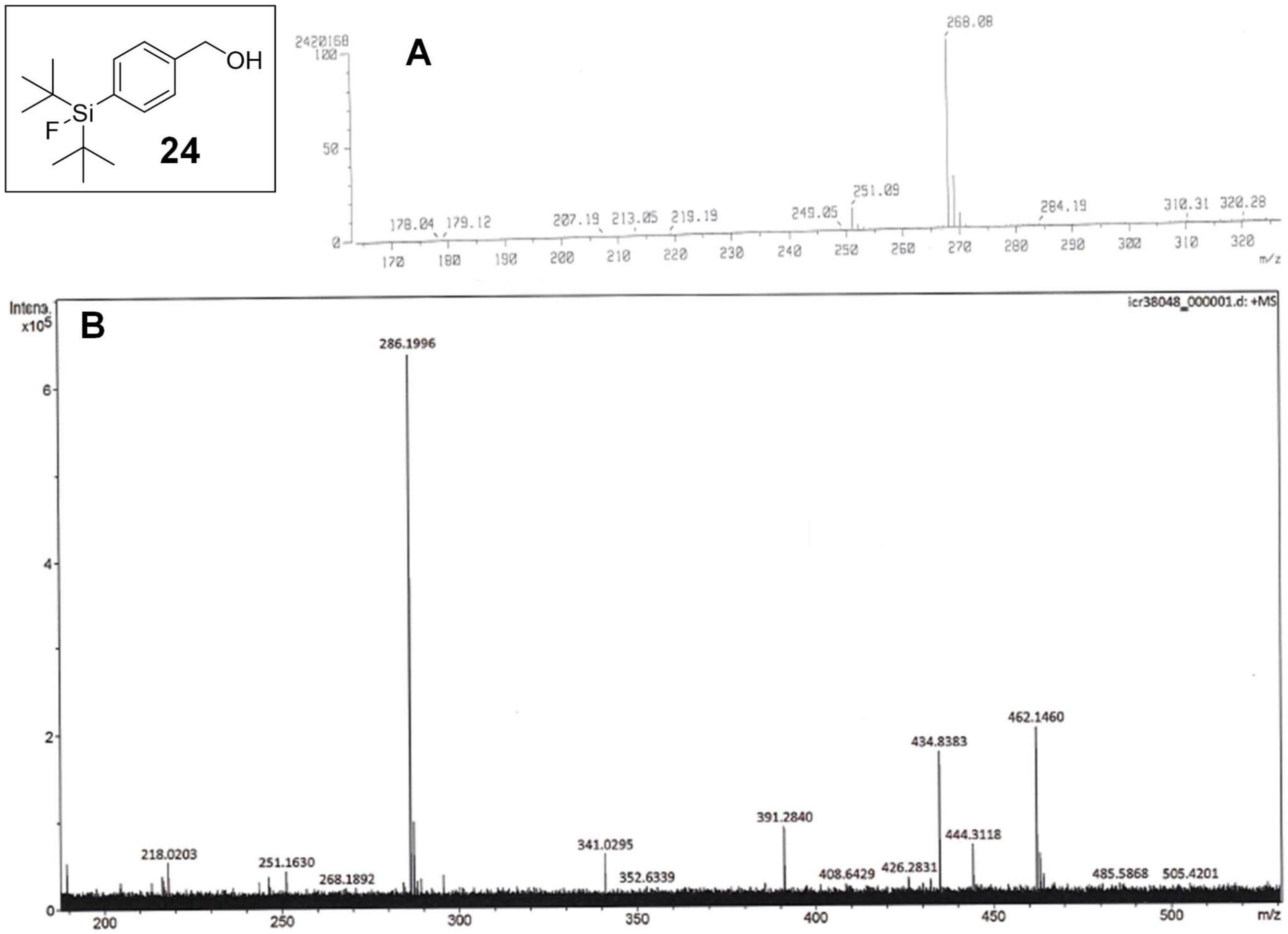

Figure S26. Mass spectra (**A**: FD, **B**: DART) of (4-(Di-*tert*-butylfluorosilyl)phenyl)methanol (**24**).

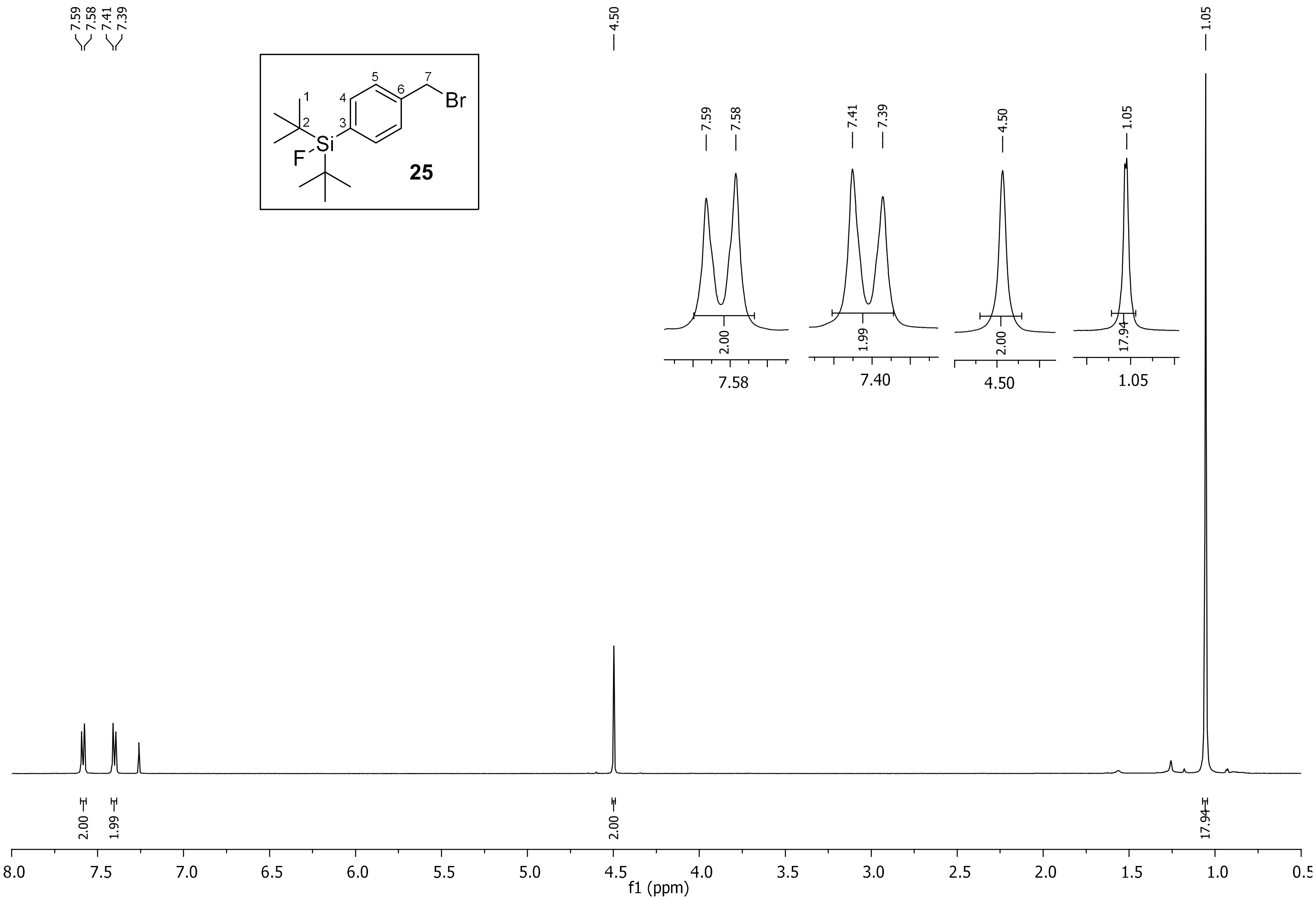

Figure S27. ^{1}H NMR spectrum of (4-(Bromomethyl)phenyl)di-*tert*-butylfluorosilane (**25**).

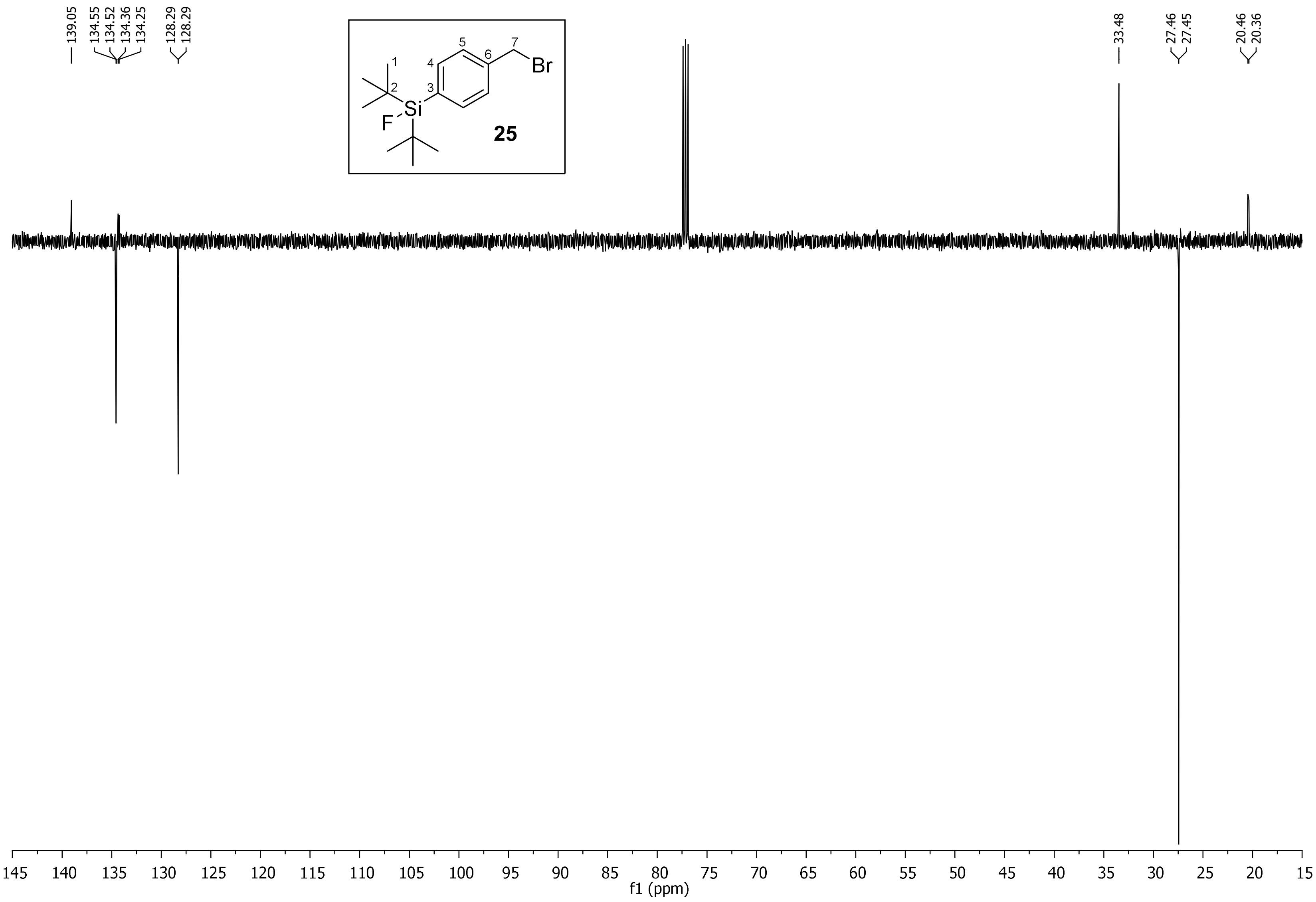

Figure S28. ^{13}C NMR spectrum of (4-(Bromomethyl)phenyl)di-*tert*-butylfluorosilane (**25**).

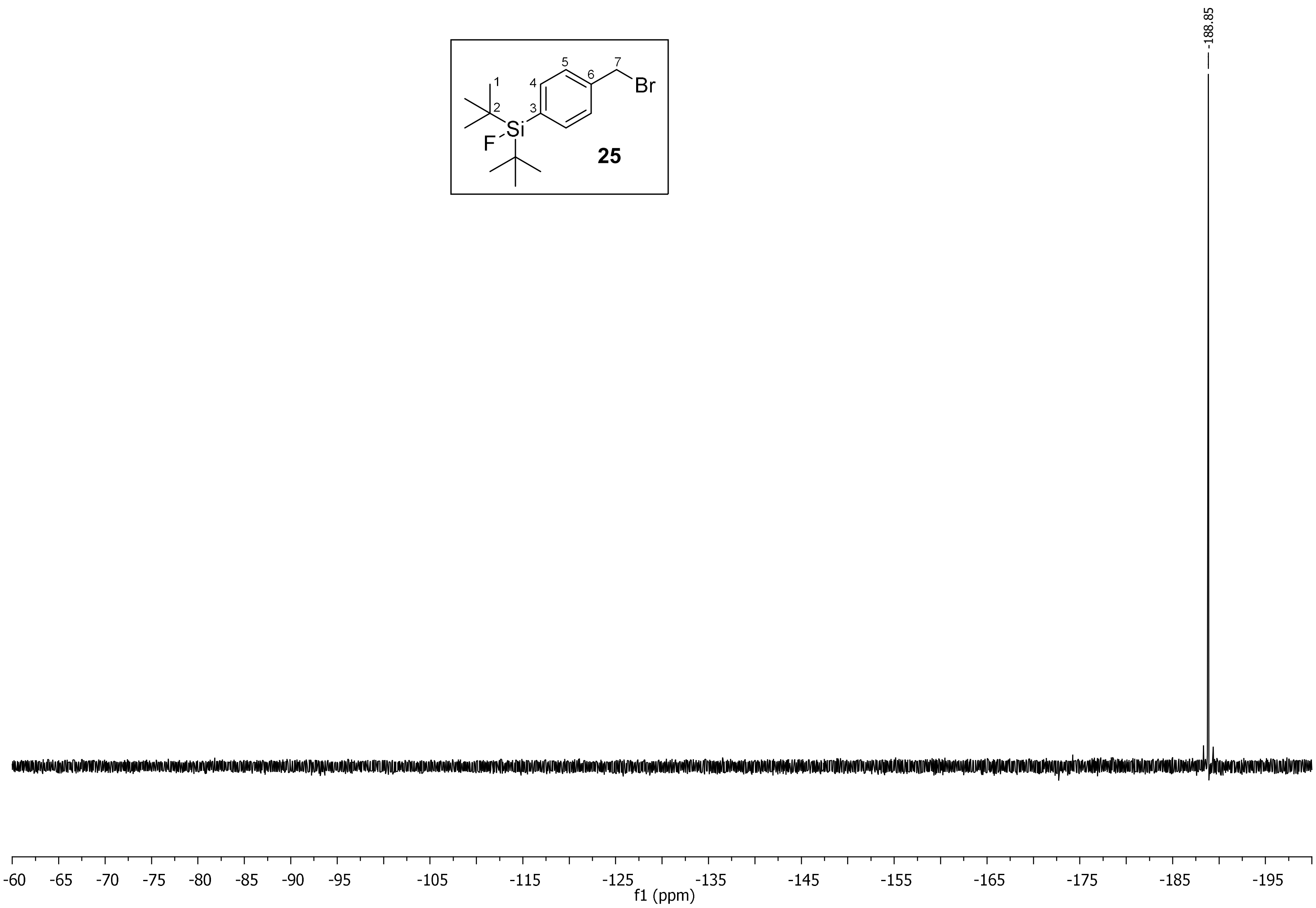

Figure S29. ^{19}F NMR spectrum of (4-(Bromomethyl)phenyl)di-*tert*-butylfluorosilane (**25**).

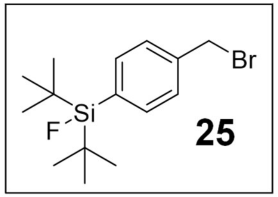

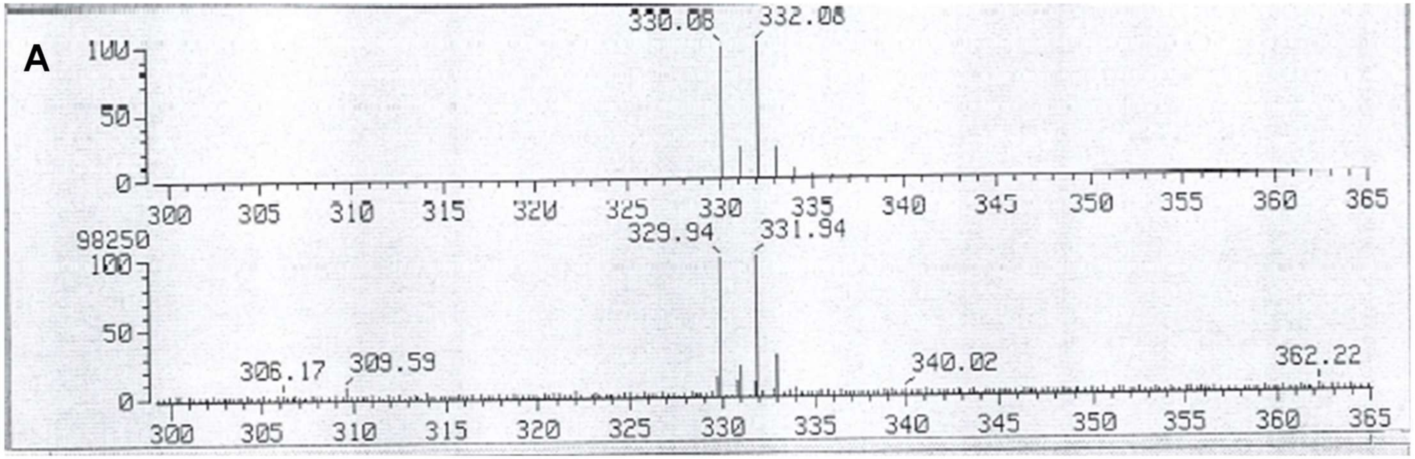

Figure S30. Mass spectrum (**A**: FD) of (4-(Bromomethyl)phenyl)di-*tert*-butylfluorosilane (**25**).

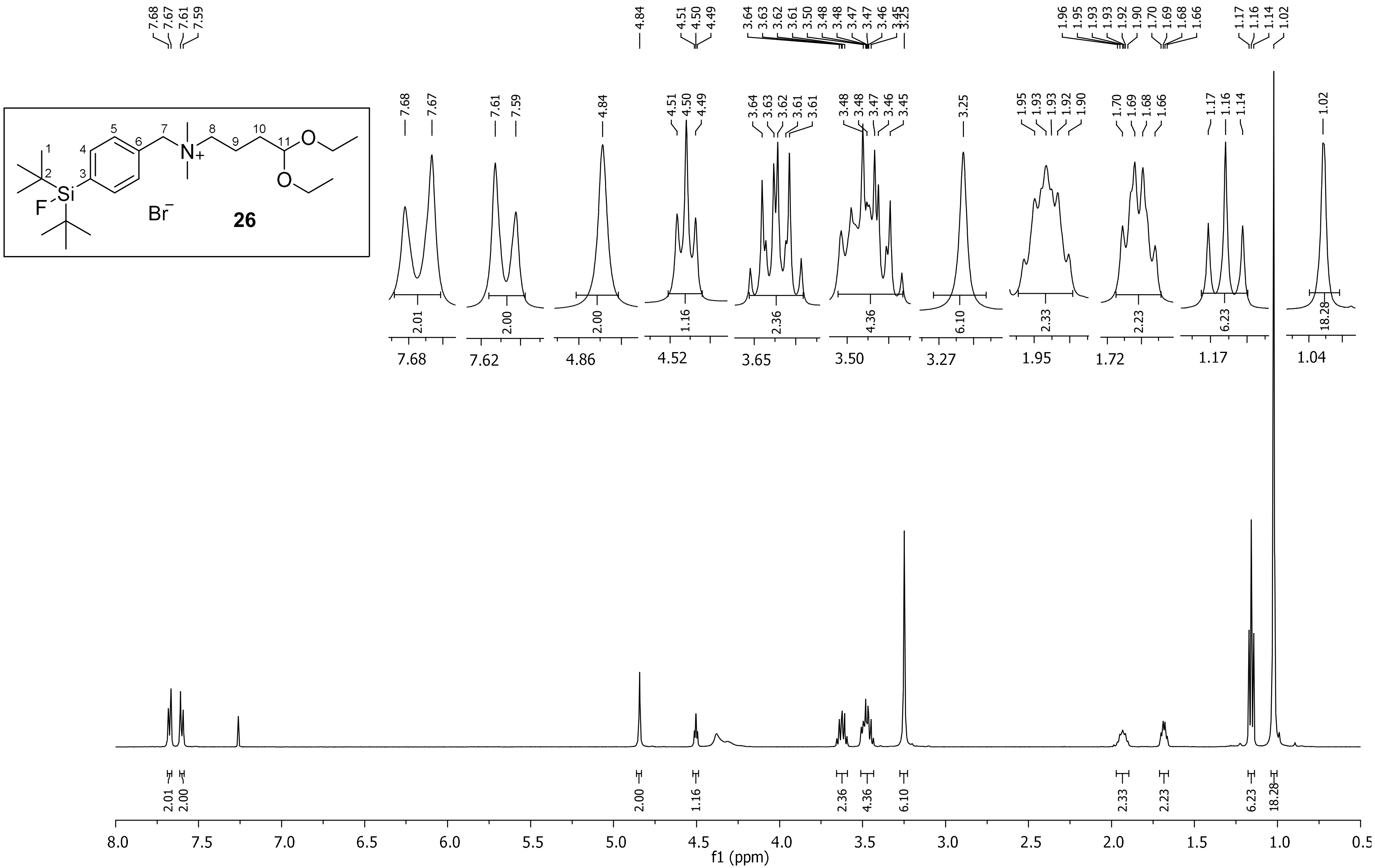

Figure S31. ^{1}H NMR spectrum of *N*-(4-(Di-*tert*-butylfluorosilyl)benzyl)-4,4-diethoxy-*N,N*-dimethylbutan-1-aminium bromide (**26**).

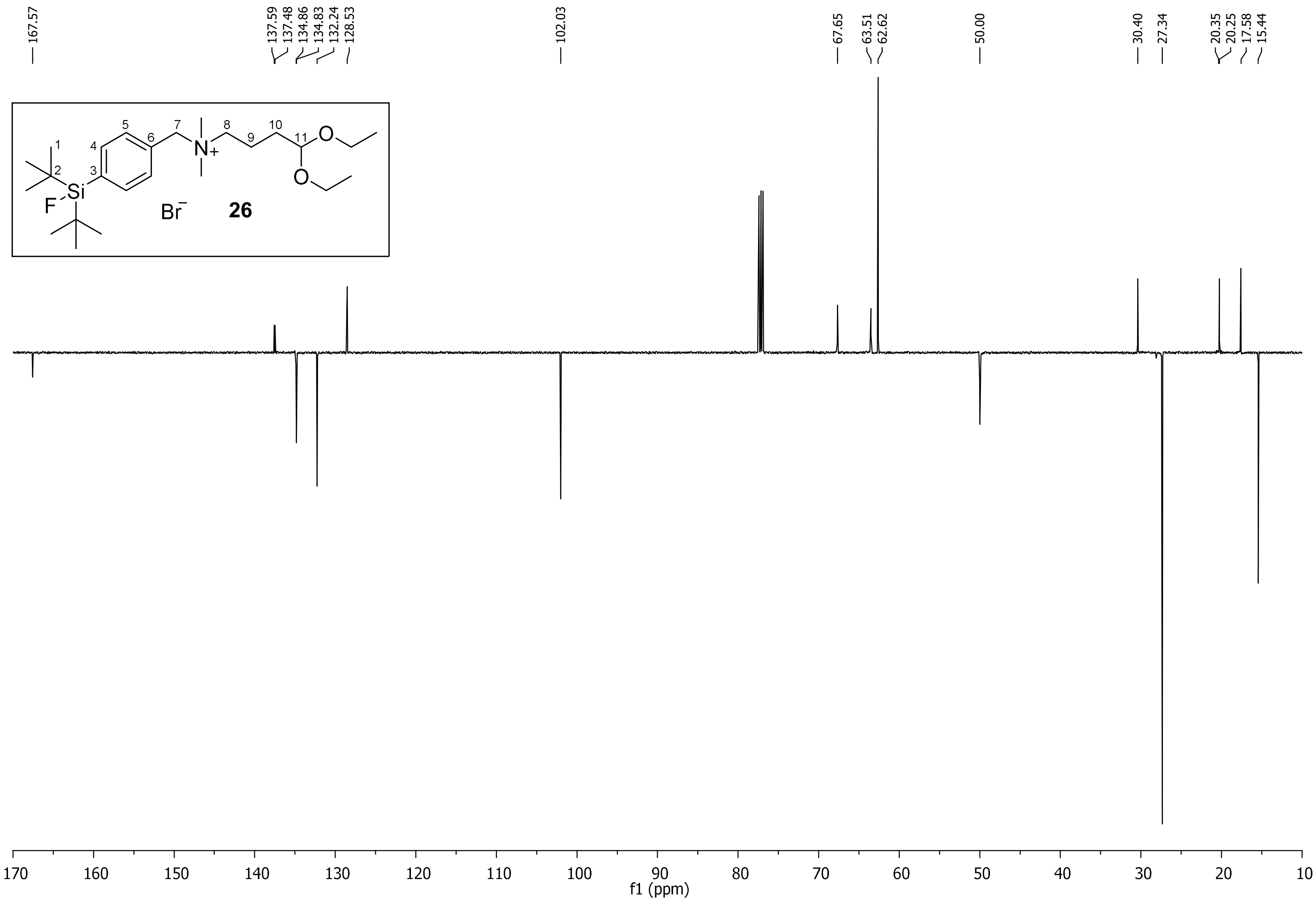

Figure S32. ^{13}C NMR spectrum of *N*-(4-(Di-*tert*-butylfluorosilyl)benzyl)-4,4-diethoxy-*N,N*-dimethylbutan-1-aminium bromide (**26**).

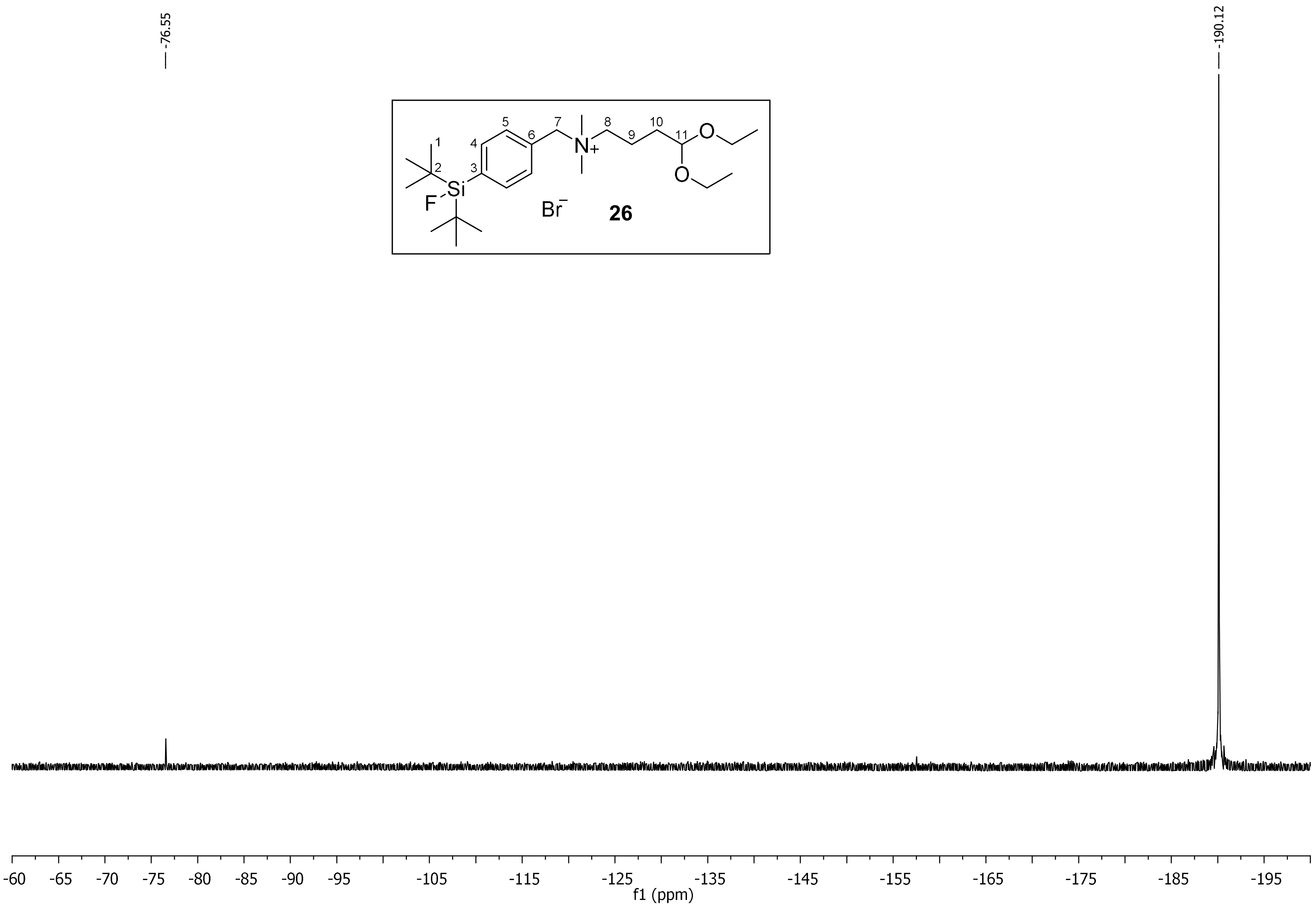

Figure S33. ^{19}F NMR spectrum of *N*-(4-(Di-*tert*-butylfluorosilyl)benzyl)-4,4-diethoxy-*N,N*-dimethylbutan-1-aminium bromide (**26**).

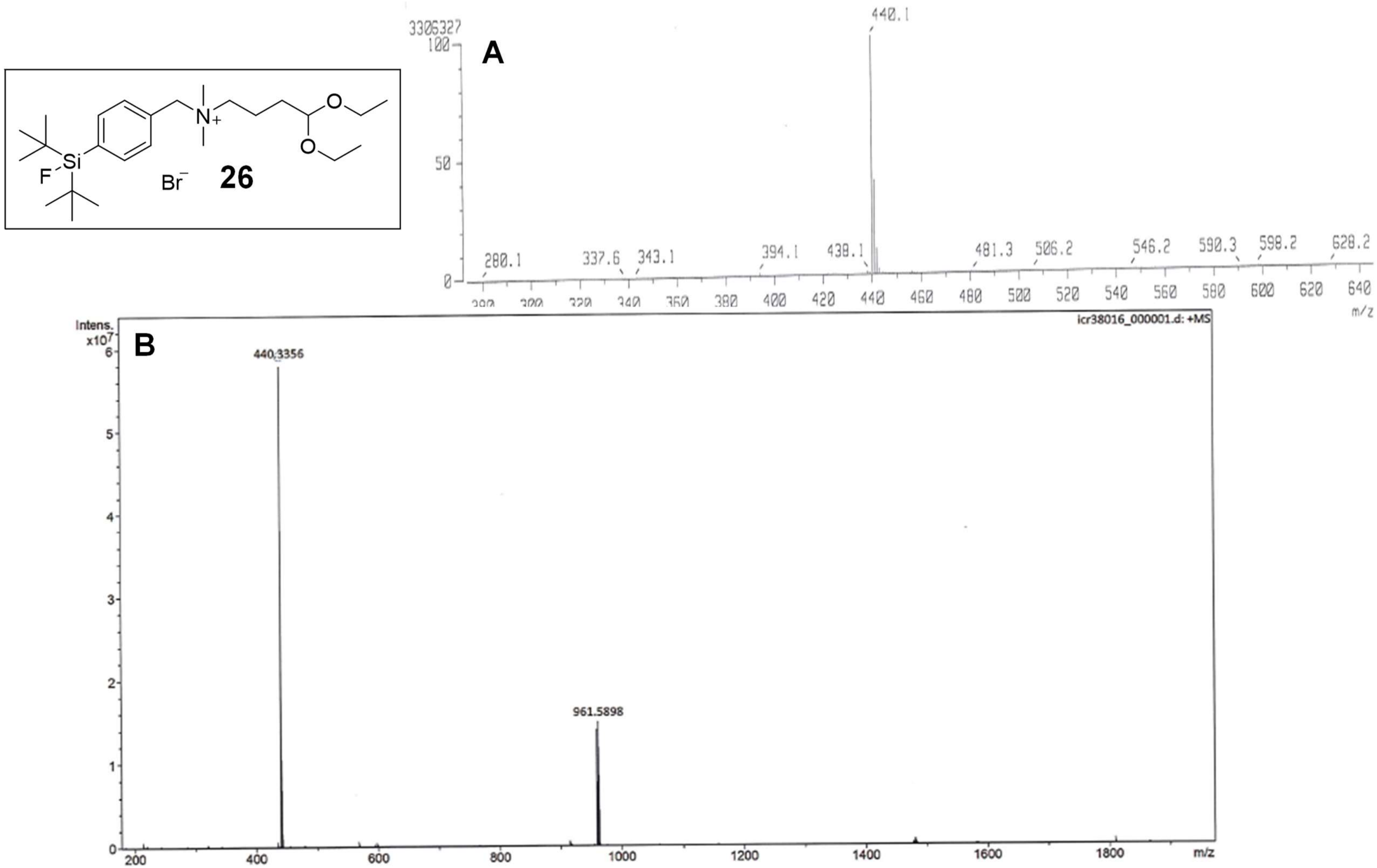

Figure S34. Mass spectra (**A**: FD, **B** ESI) of *N*-(4-(Di-*tert*-butylfluorosilyl)benzyl)-4,4-diethoxy-*N,N*-dimethylbutan-1-aminium bromide (**26**).

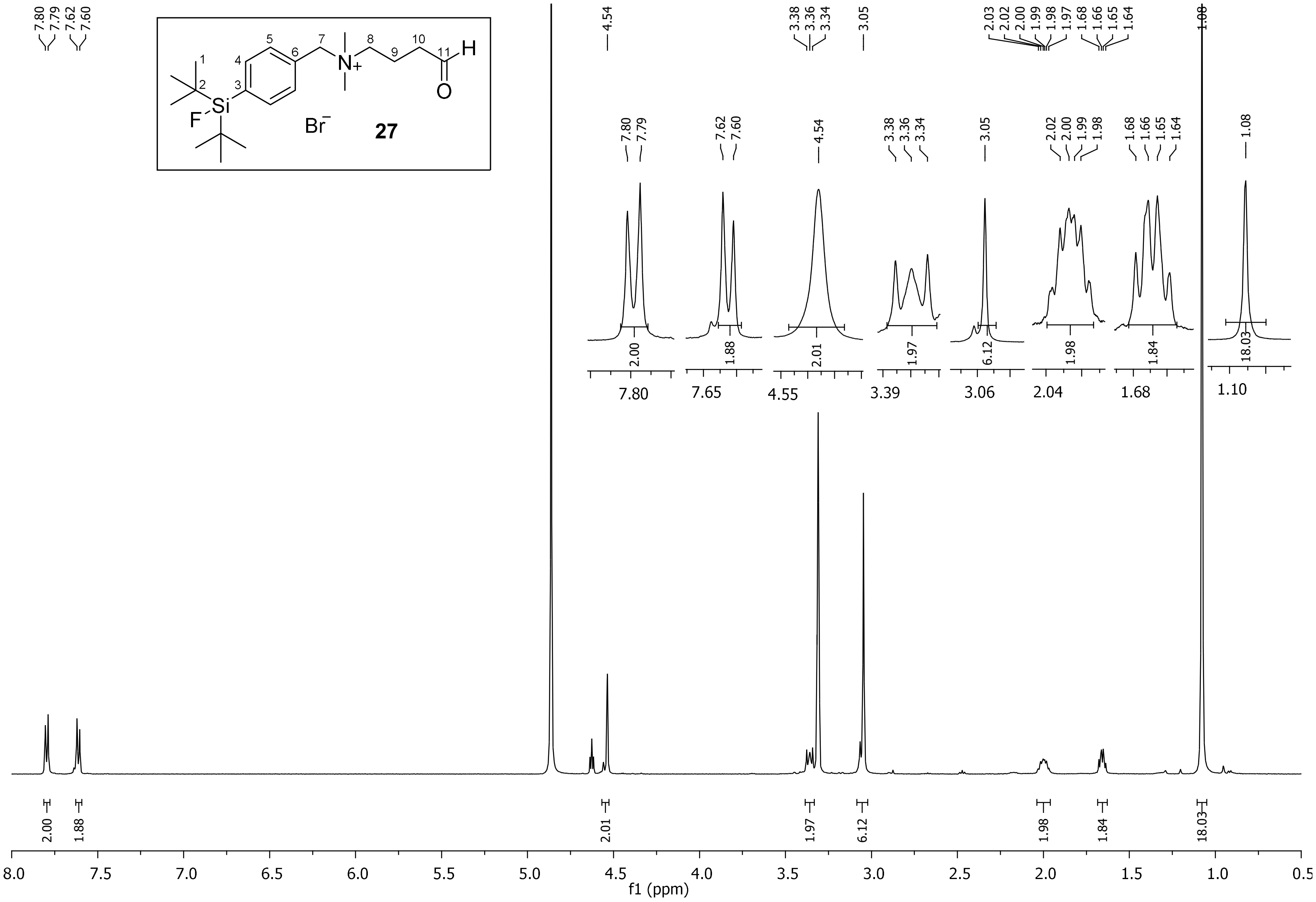

Figure S35. ^{1}H NMR spectrum of *N*-(4-(Di-*tert*-butylfluorosilyl)benzyl)-*N,N*-dimethyl-4-oxobutan-1-aminium bromide (**27**).

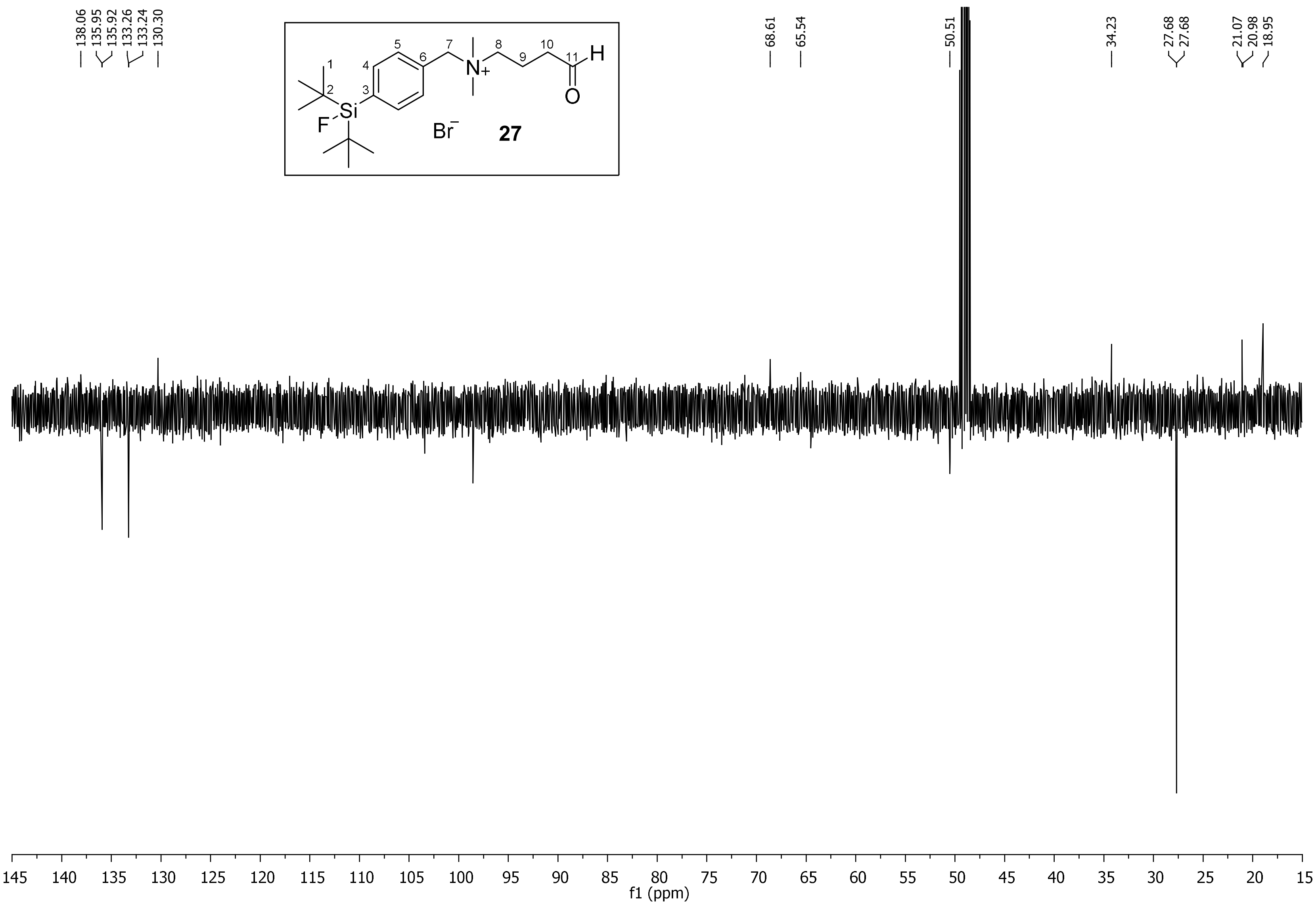

Figure S36. ^{13}C NMR spectrum of *N*-(4-(Di-*tert*-butylfluorosilyl)benzyl)-*N,N*-dimethyl-4-oxobutan-1-aminium bromide (**27**).

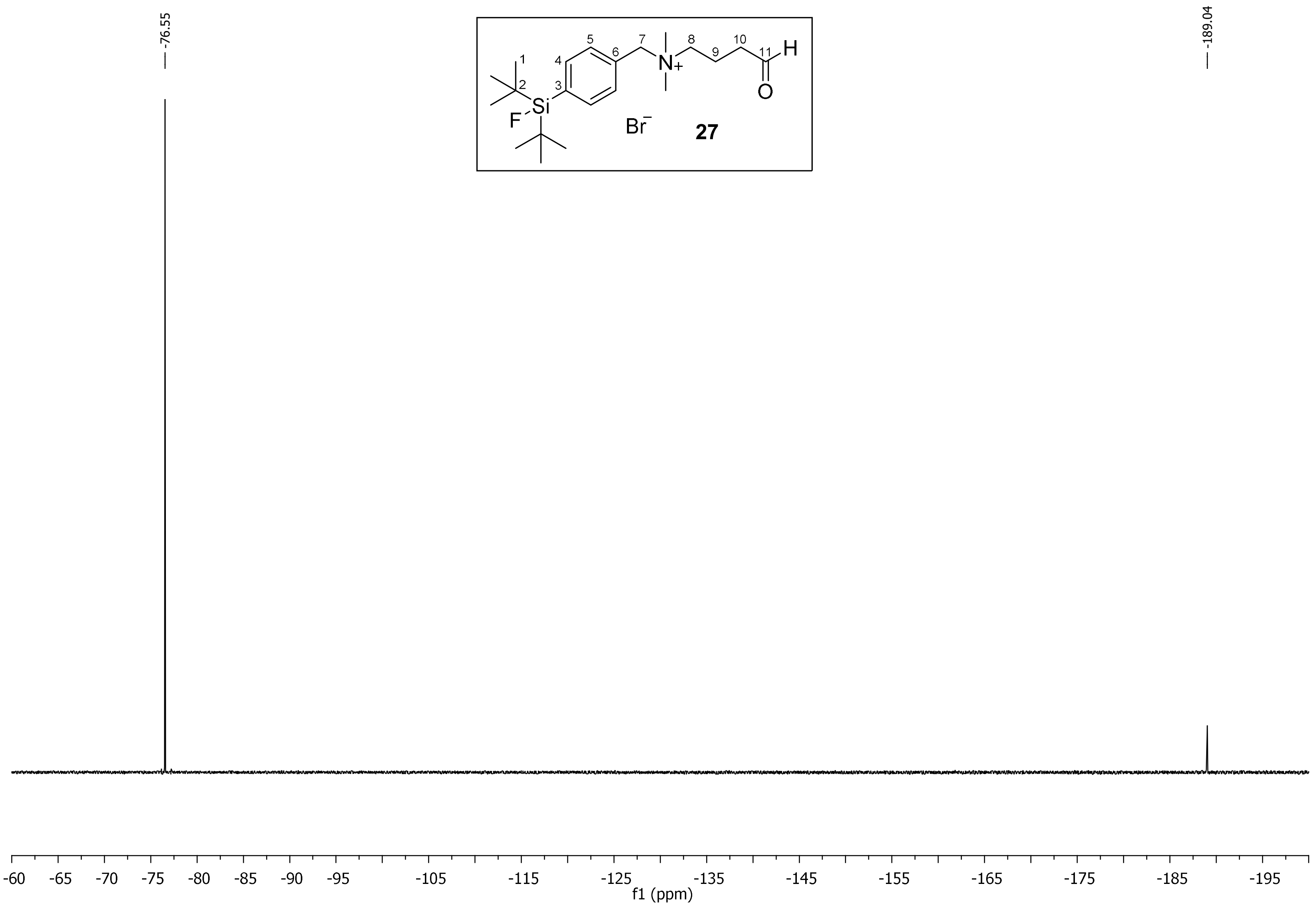

Figure S37. ^{19}F NMR spectrum of *N*-(4-(Di-*tert*-butylfluorosilyl)benzyl)-*N,N*-dimethyl-4-oxobutan-1-aminium bromide (**27**).

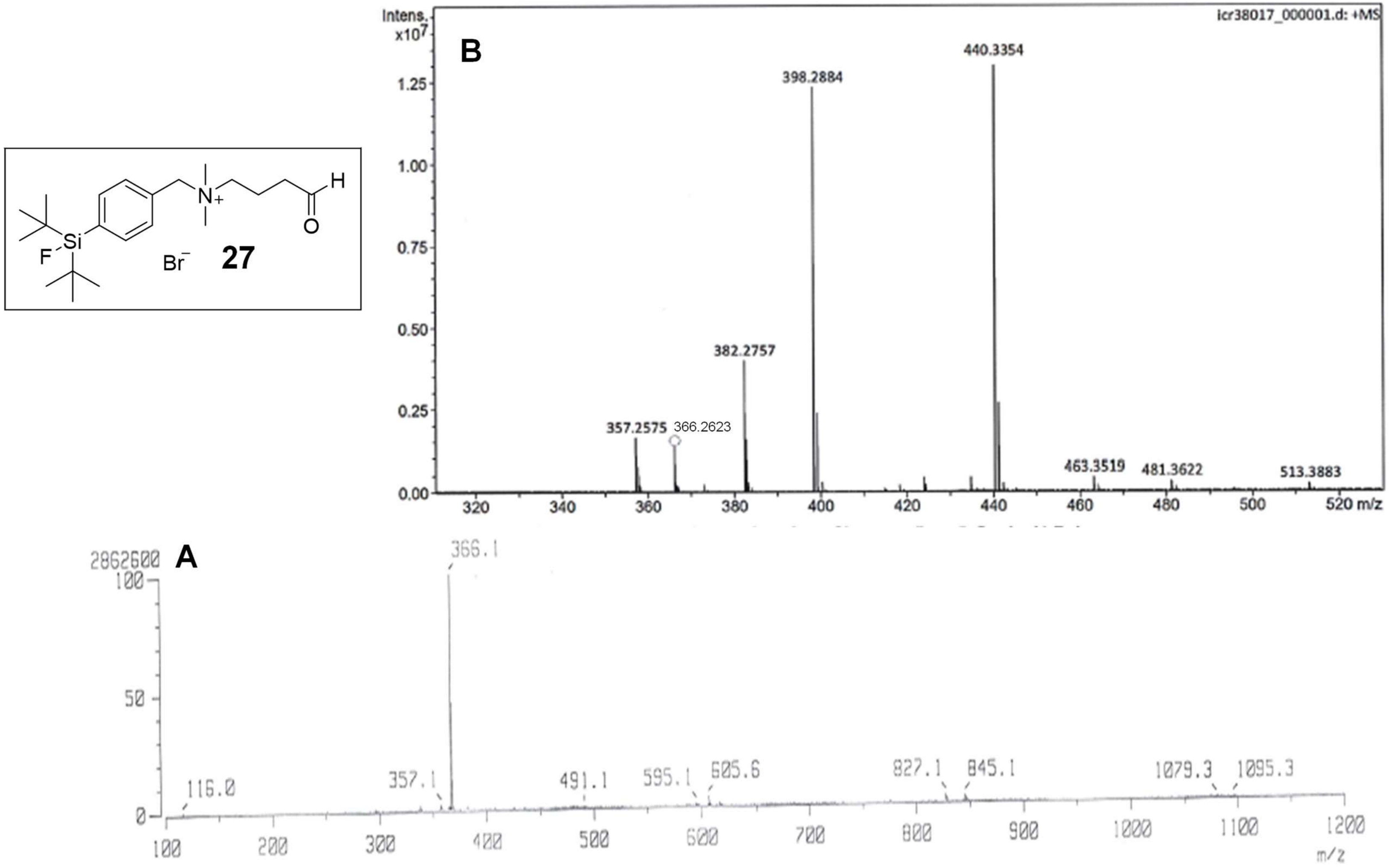

Figure S38. Mass spectra (**A**: FD, **B** ESI) of *N*-(4-(Di-*tert*-butylfluorosilyl)benzyl)-*N,N*-dimethyl-4-oxobutan-1-aminium bromide (**27**).

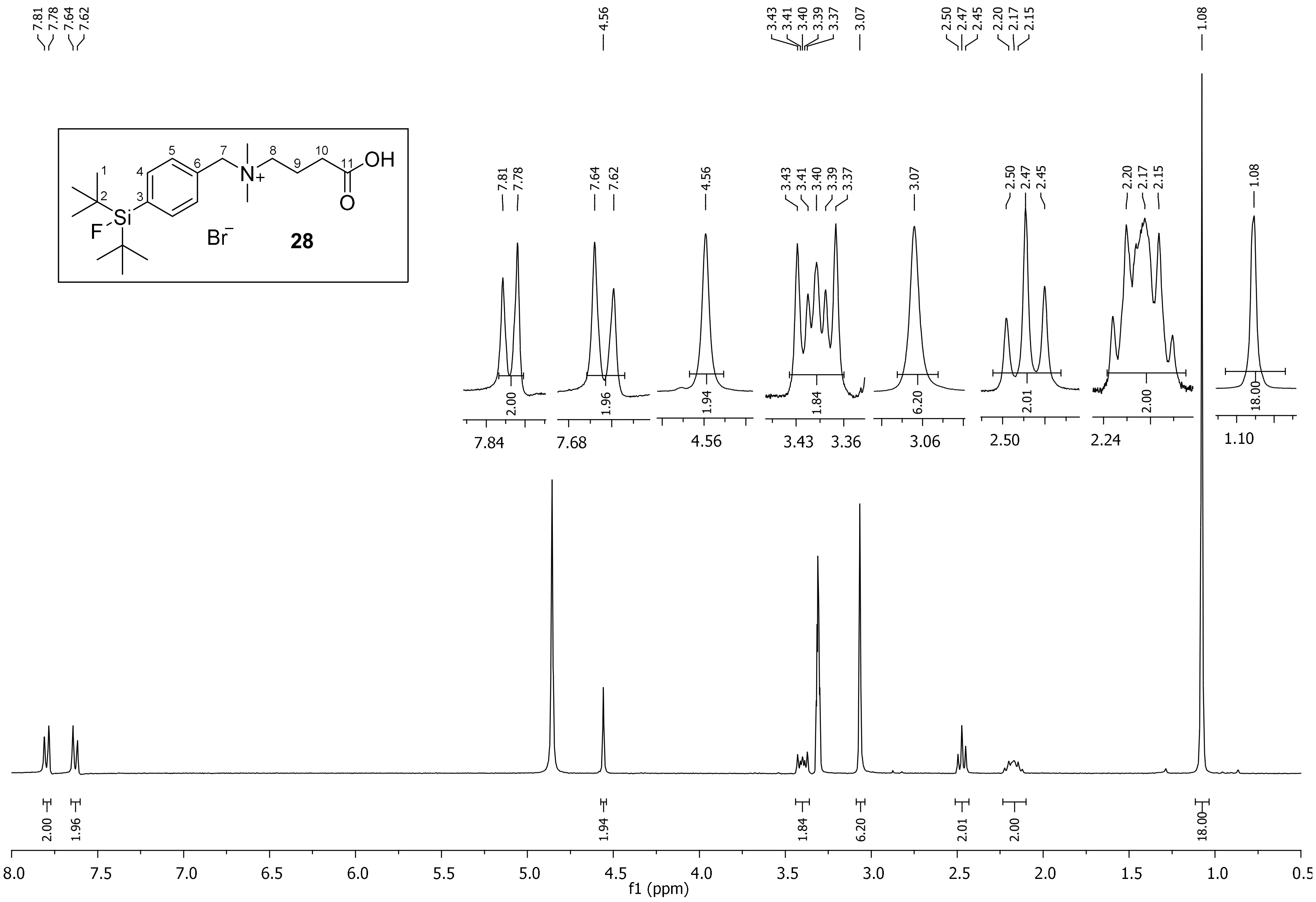

Figure S39. ^{1}H NMR spectrum of 3-Carboxy-*N*-(4-(di-*tert*-butylfluorosilyl)benzyl)-*N,N*-dimethylpropan-1-aminium bromide (**28**).

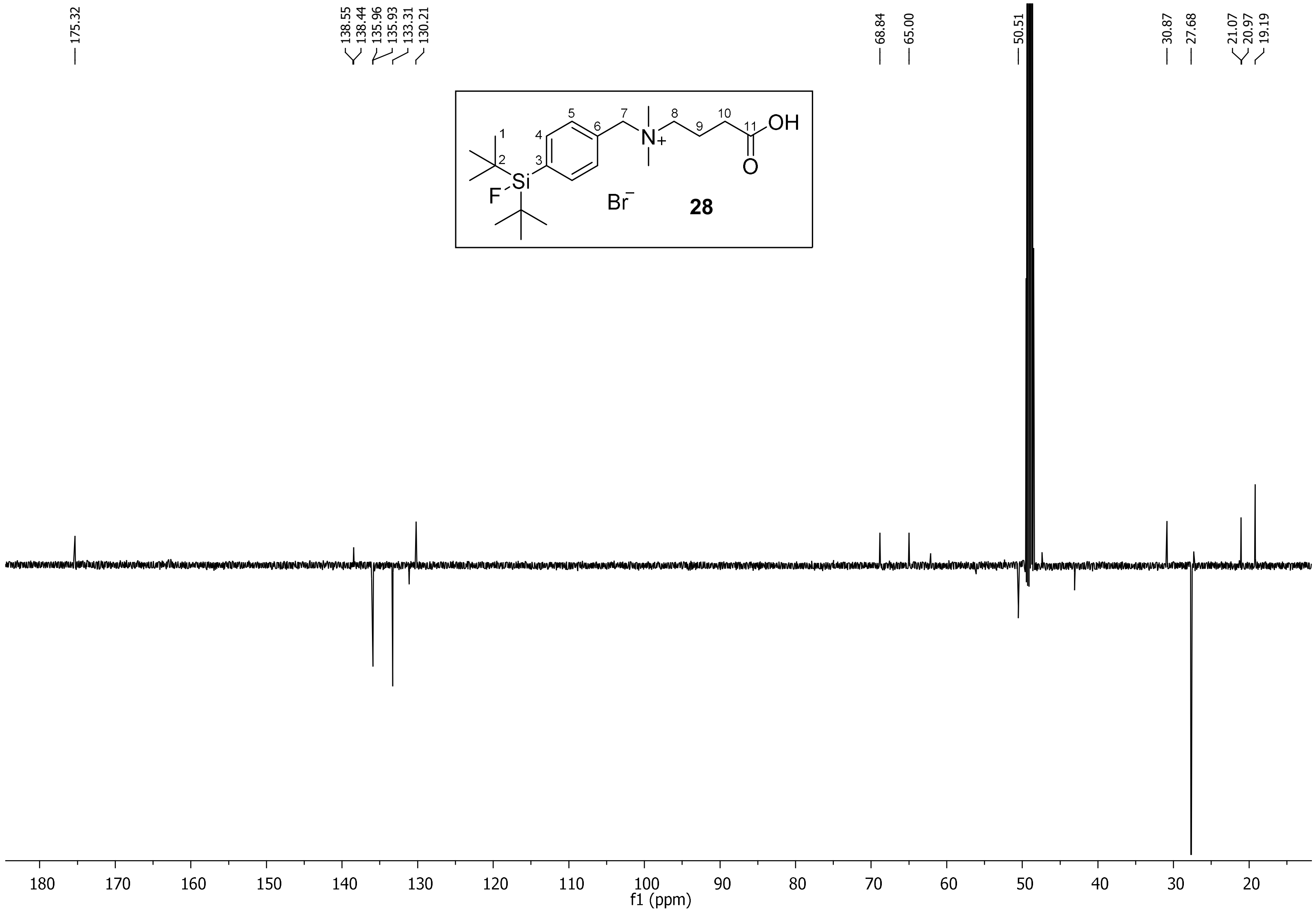

Figure S40. ^{13}C NMR spectrum of 3-Carboxy-*N*-(4-(di-*tert*-butylfluorosilyl)benzyl)-*N,N*-dimethylpropan-1-aminium bromide (**28**).

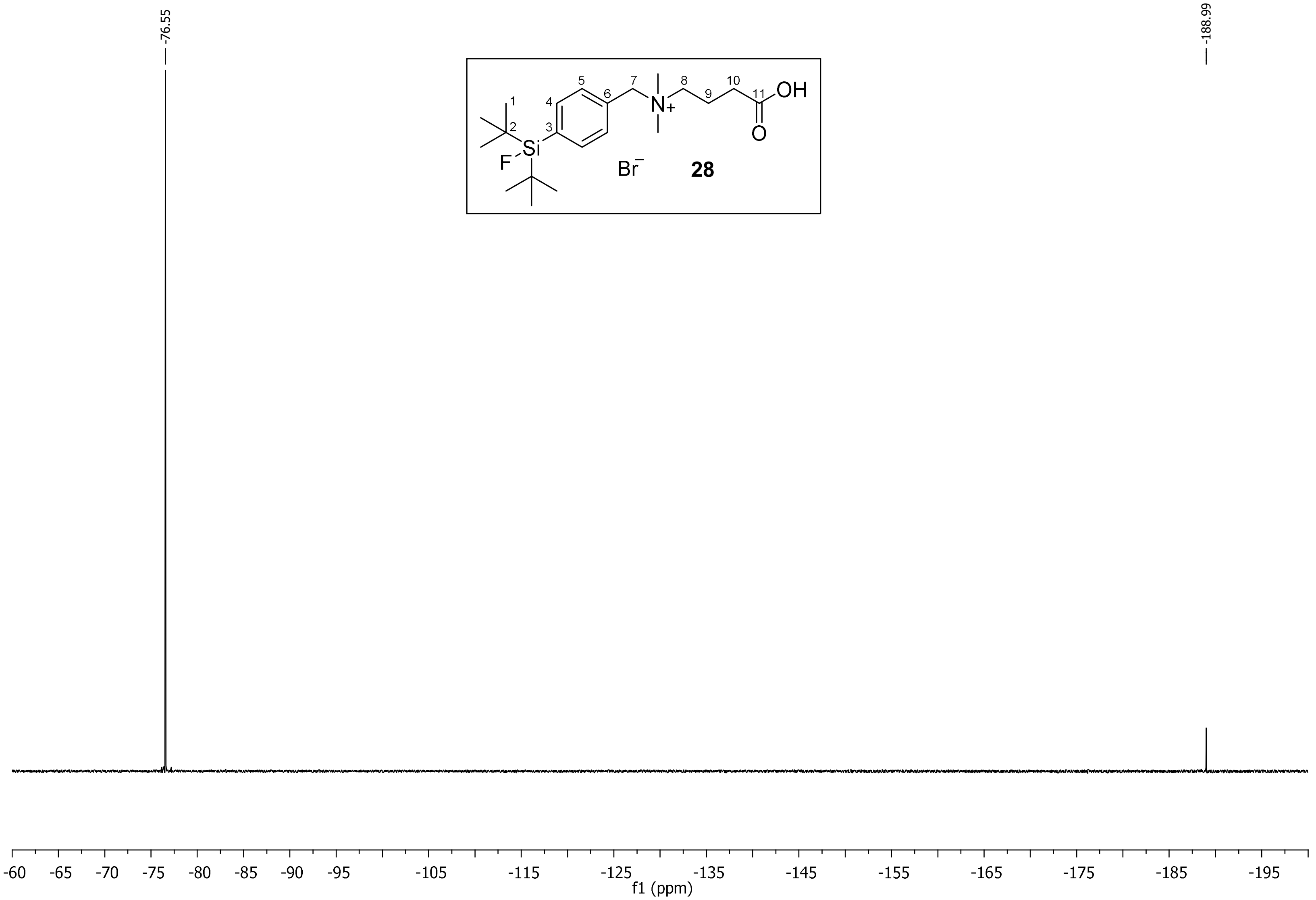

Figure S41. ^{19}F NMR spectrum of 3-Carboxy-*N*-(4-(di-*tert*-butylfluorosilyl)benzyl)-*N,N*-dimethylpropan-1-aminium bromide (**28**).

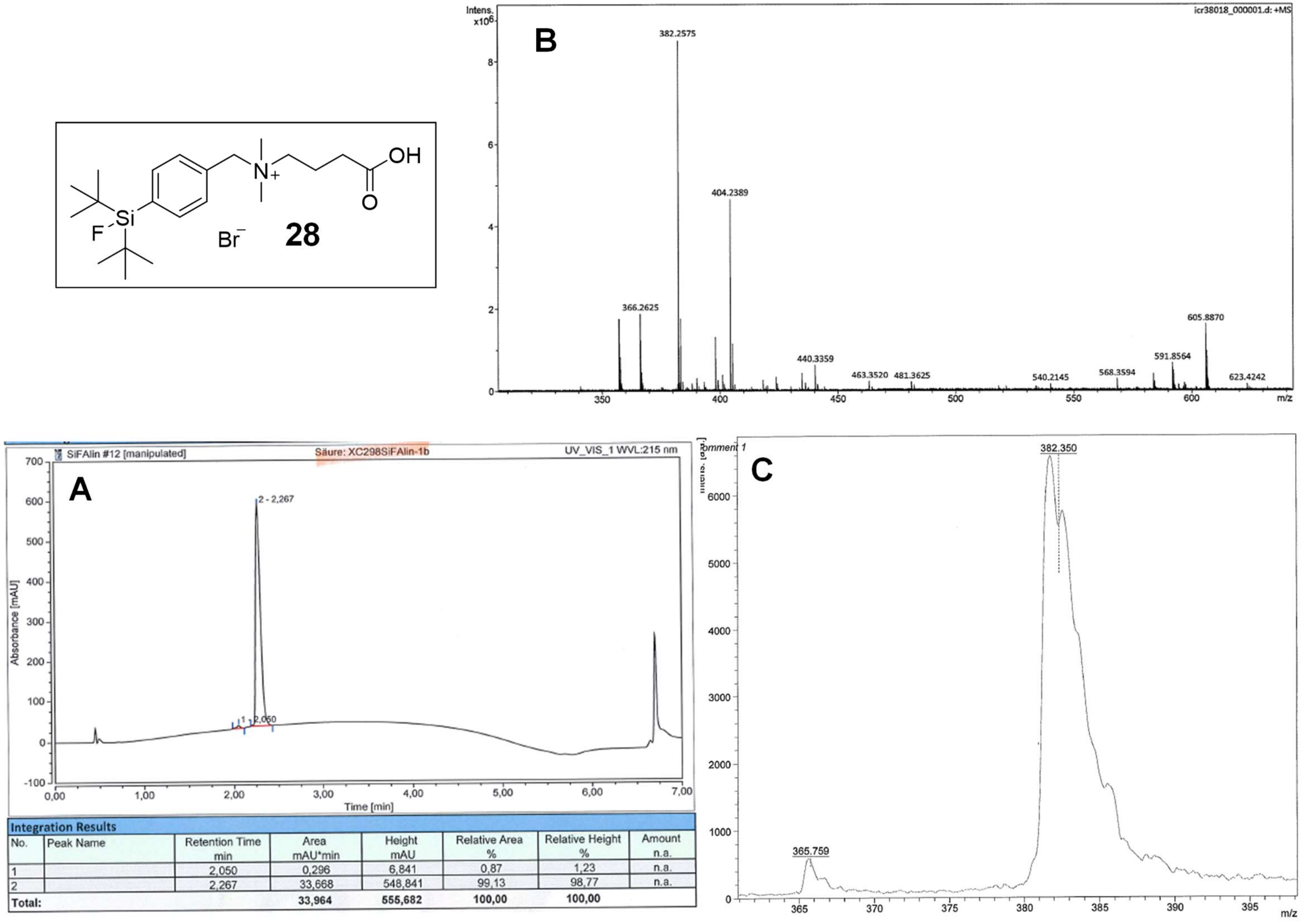

Integration Results

No.	Peak Name	Retention Time min	Area mAU*min	Height mAU	Relative Area %	Relative Height %	Amount n.a.
1		2,050	0,296	6,841	0,87	1,23	n.a.
2		2,267	33,668	548,841	99,13	98,77	n.a.
Total:			33,964	555,682	100,00	100,00	

Figure S42. HPLC chromatogram (**A**) and mass spectra (**B**: ESI, **C** MALDI) of 3-Carboxy-*N*-(4-(di-*tert*-butylfluorosilyl)benzyl)-*N,N*-dimethylpropan-1-aminium bromide (**28**).

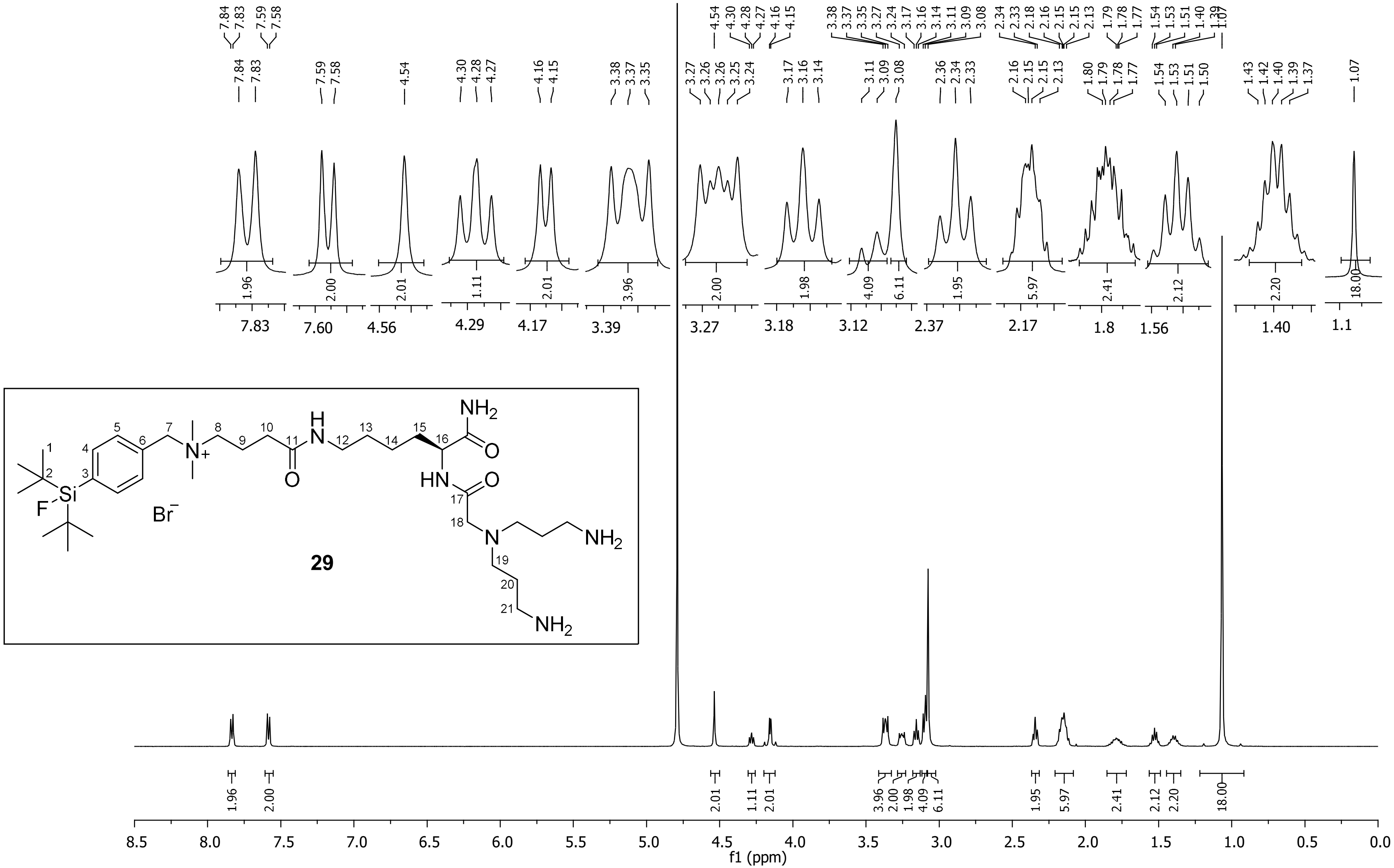

Figure S43. ^{1}H NMR spectrum of (*S*)-4-((6-Amino-5-(2-(bis(3-aminopropyl)amino)acetamido)-6-oxohexyl)amino)-*N*-(4-(di-*tert*-butylfluorosilyl)benzyl)-*N,N*-dimethyl-4-oxobutan-1-aminium bromide (**29**).

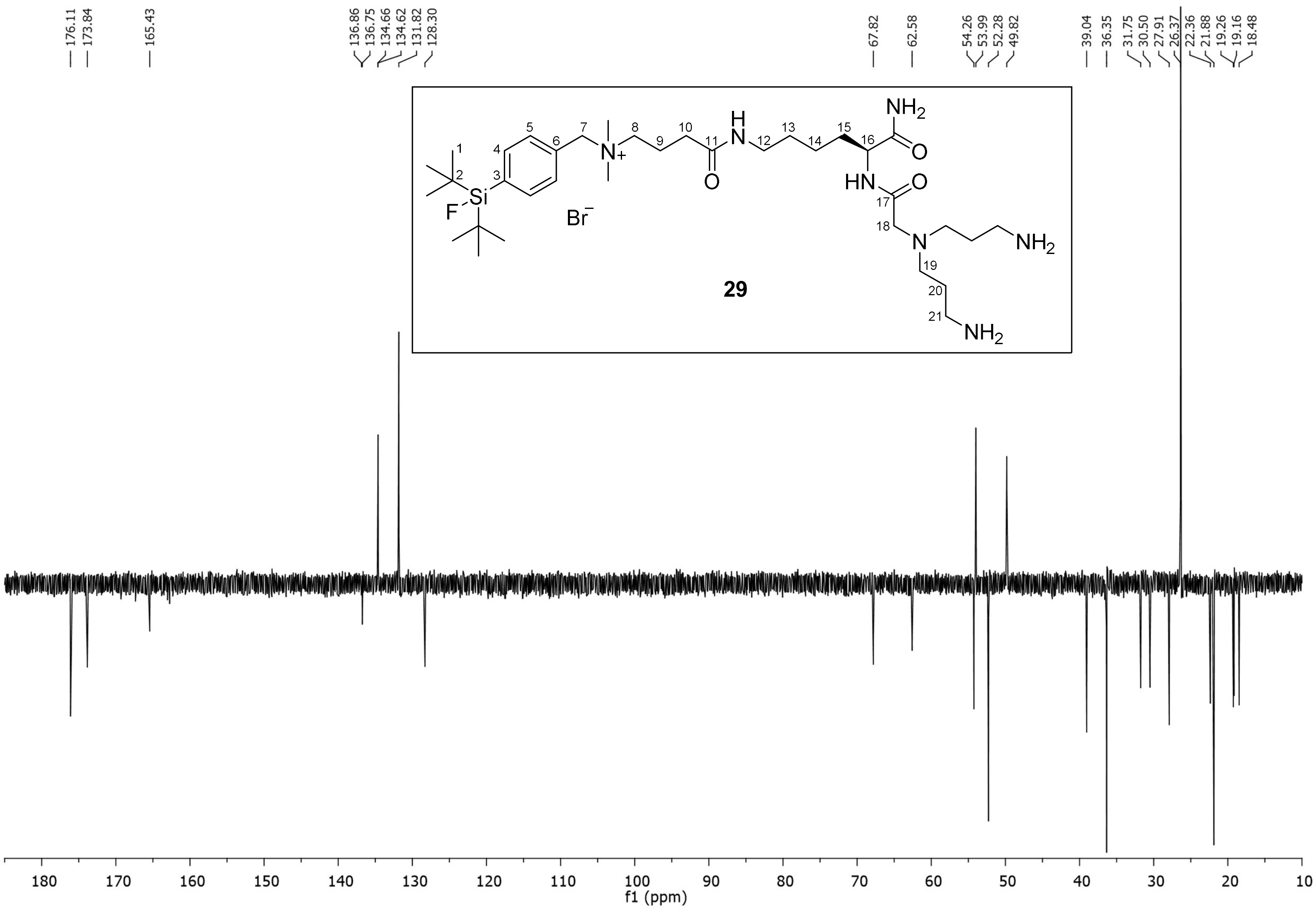

Figure S44. ^{13}C NMR spectrum of (*S*)-4-((6-Amino-5-(2-(bis(3-aminopropyl)amino)acetamido)-6-oxohexyl)amino)-*N*-(4-(di-*tert*-butylfluorosilyl)benzyl)-*N,N*-dimethyl-4-oxobutan-1-aminium bromide (**29**).

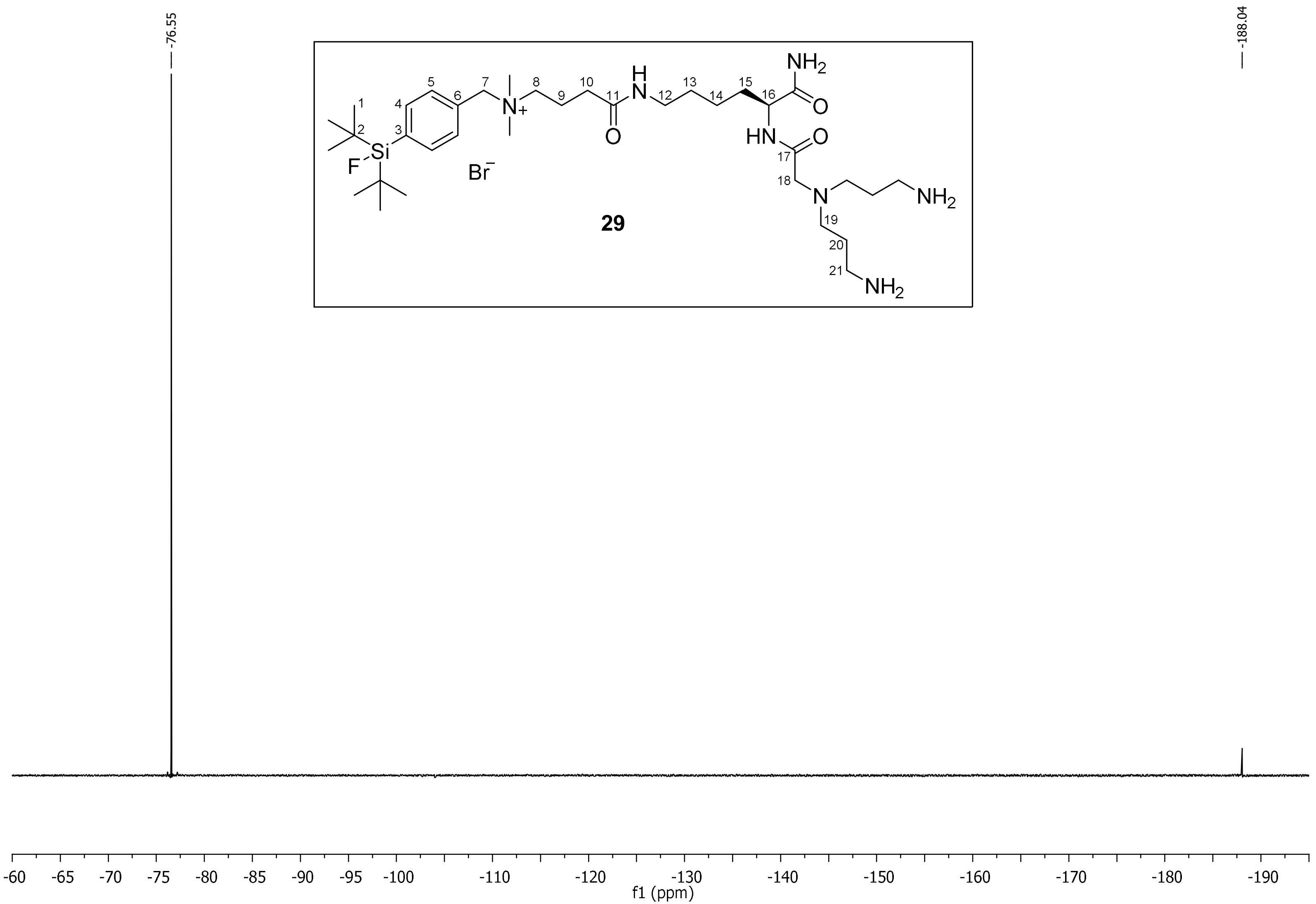

Figure S45. ^{19}F NMR spectrum of (*S*)-4-((6-Amino-5-(2-(bis(3-aminopropyl)amino)acetamido)-6-oxohexyl)amino)-*N*-(4-(di-*tert*-butylfluorosilyl)benzyl)-*N,N*-dimethyl-4-oxobutan-1-aminium bromide (**29**).

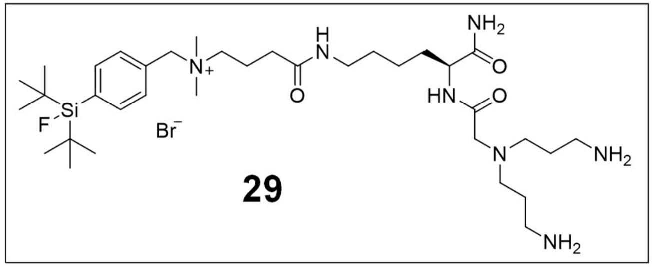

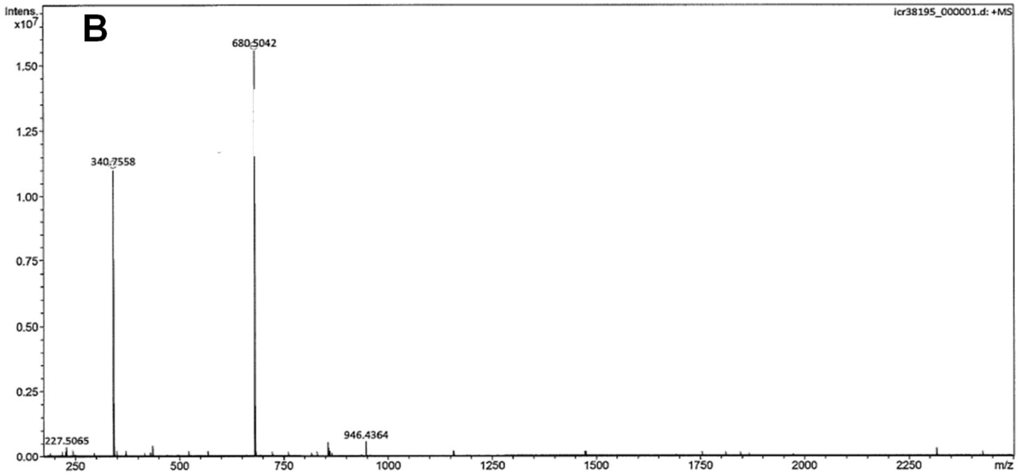

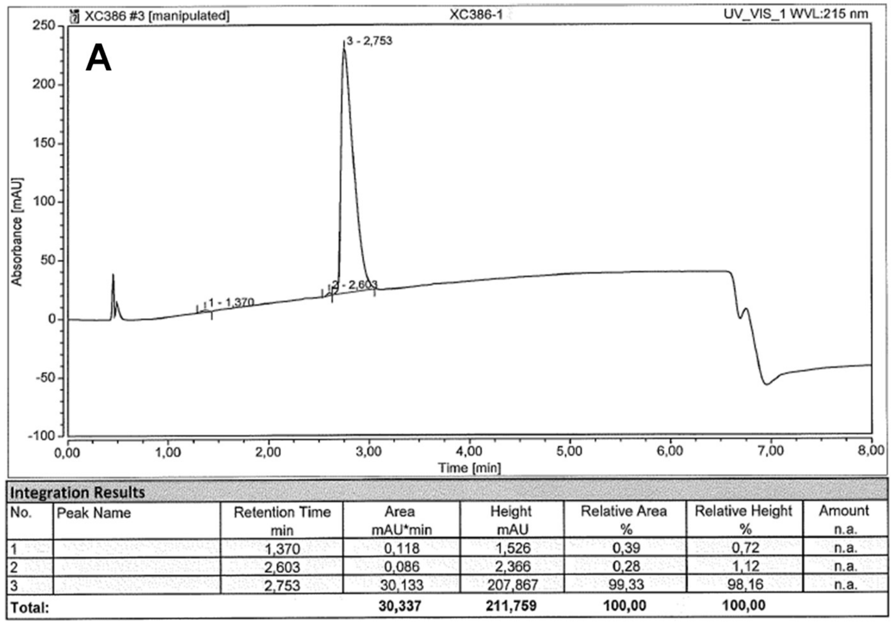

Integration Results							
No.	Peak Name	Retention Time min	Area mAU*min	Height mAU	Relative Area %	Relative Height %	Amount n.a.
1		1,370	0,118	1,526	0,39	0,72	n.a.
2		2,603	0,086	2,366	0,28	1,12	n.a.
3		2,753	30,133	207,867	99,33	98,16	n.a.
**Total:**			**30,337**	**211,759**	**100,00**	**100,00**	

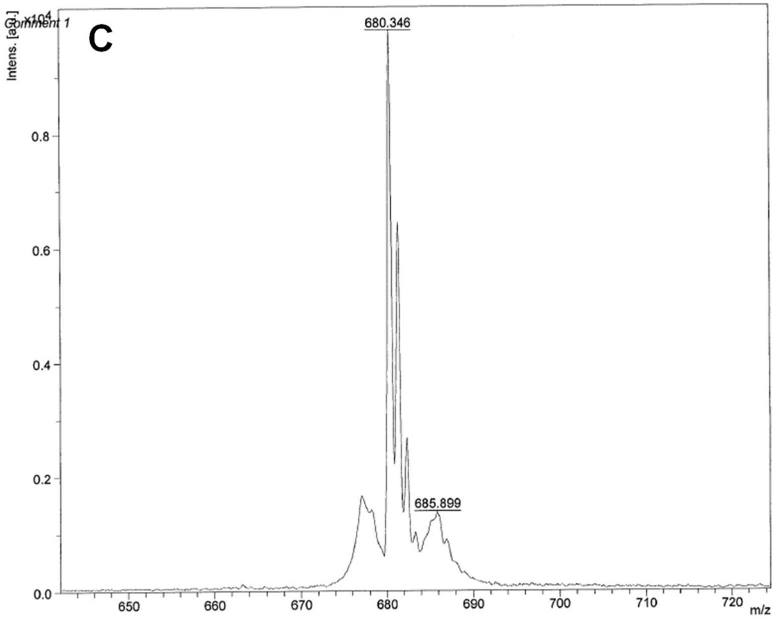

Figure S46. HPLC chromatogram (**A**) and mass spectra (**B**: ESI, **C** MALDI) of (*S*)-4-((6-Amino-5-(2-(bis(3-aminopropyl)amino)acetamido)-6-oxohexyl)amino)-*N*-(4-(di-*tert*-butylfluorosilyl)benzyl)-*N,N*-dimethyl-4-oxobutan-1-aminium bromide (**29**).

3. Mass and ^{19}F-NMR spectra of HBPLs 1–6 and mass spectra of their intermediates 30–35

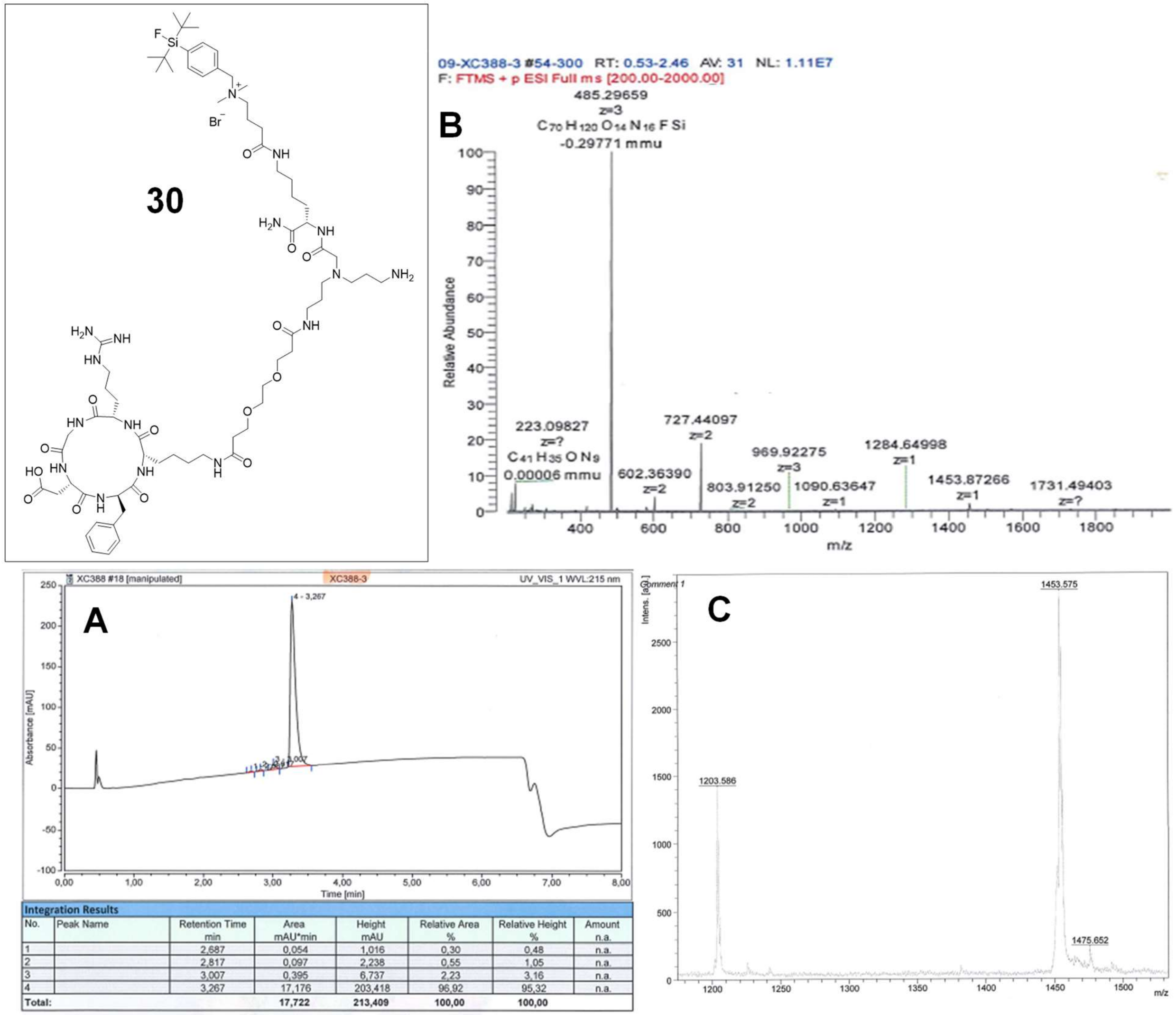

Integration Results							
No.	Peak Name	Retention Time min	Area mAU*min	Height mAU	Relative Area %	Relative Height %	Amount n.a.
1		2,687	0,054	1,016	0,30	0,48	n.a.
2		2,817	0,097	2,238	0,55	1,05	n.a.
3		3,007	0,395	6,737	2,23	3,16	n.a.
4		3,267	17,176	203,418	96,92	95,32	n.a.
Total:			17,722	213,409	100,00	100,00	

Figure S47. HPLC chromatogram (**A**) and mass spectra (**B**: ESI, **C**: MALDI) of SiFA*lin*-APG-PEG$_1$-c(RGDfK) (**30**).

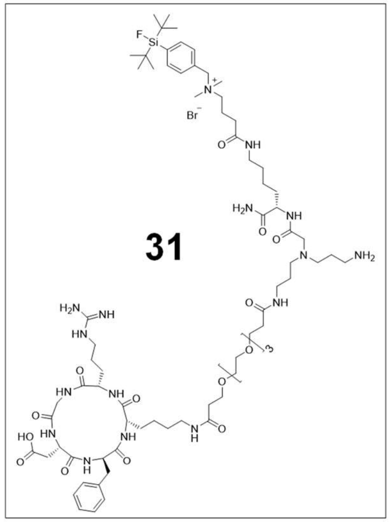

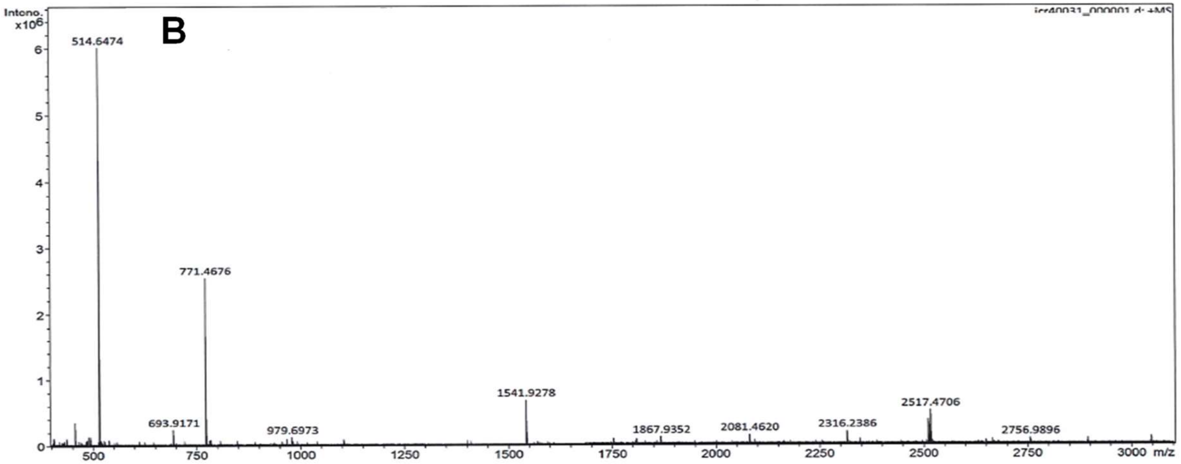

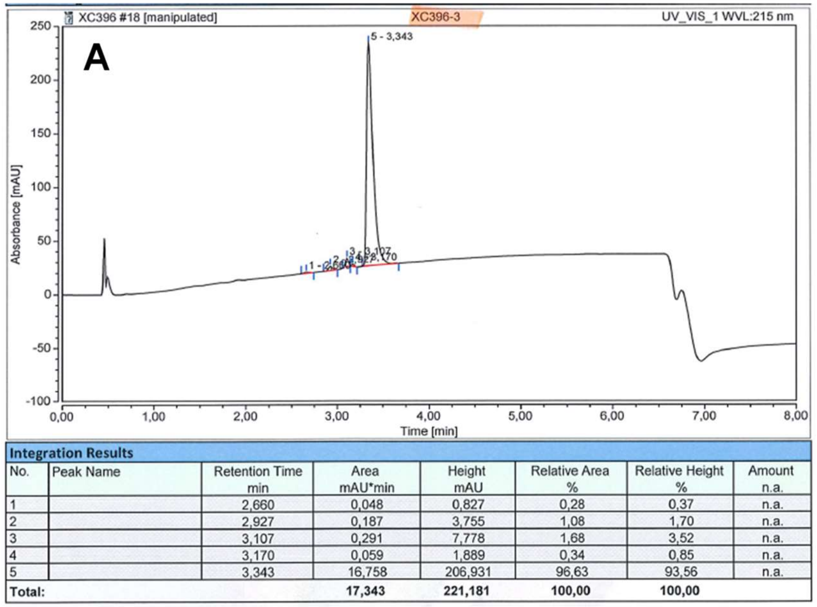

Integration Results							
No.	Peak Name	Retention Time min	Area mAU*min	Height mAU	Relative Area %	Relative Height %	Amount n.a.
1		2,660	0,048	0,827	0,28	0,37	n.a.
2		2,927	0,187	3,755	1,08	1,70	n.a.
3		3,107	0,291	7,778	1,68	3,52	n.a.
4		3,170	0,059	1,889	0,34	0,85	n.a.
5		3,343	16,758	206,931	96,63	93,56	n.a.
Total:			17,343	221,181	100,00	100,00	

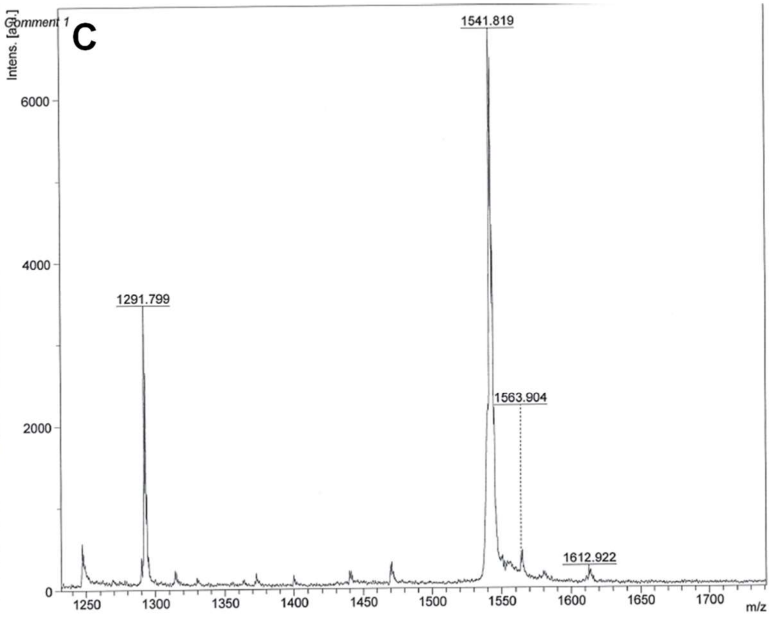

Figure S48. HPLC chromatogram (**A**) and mass spectra (**B**: ESI, **C**: MALDI) of SiFA*lin*-APG-PEG$_3$-c(RGDfK) (**31**).

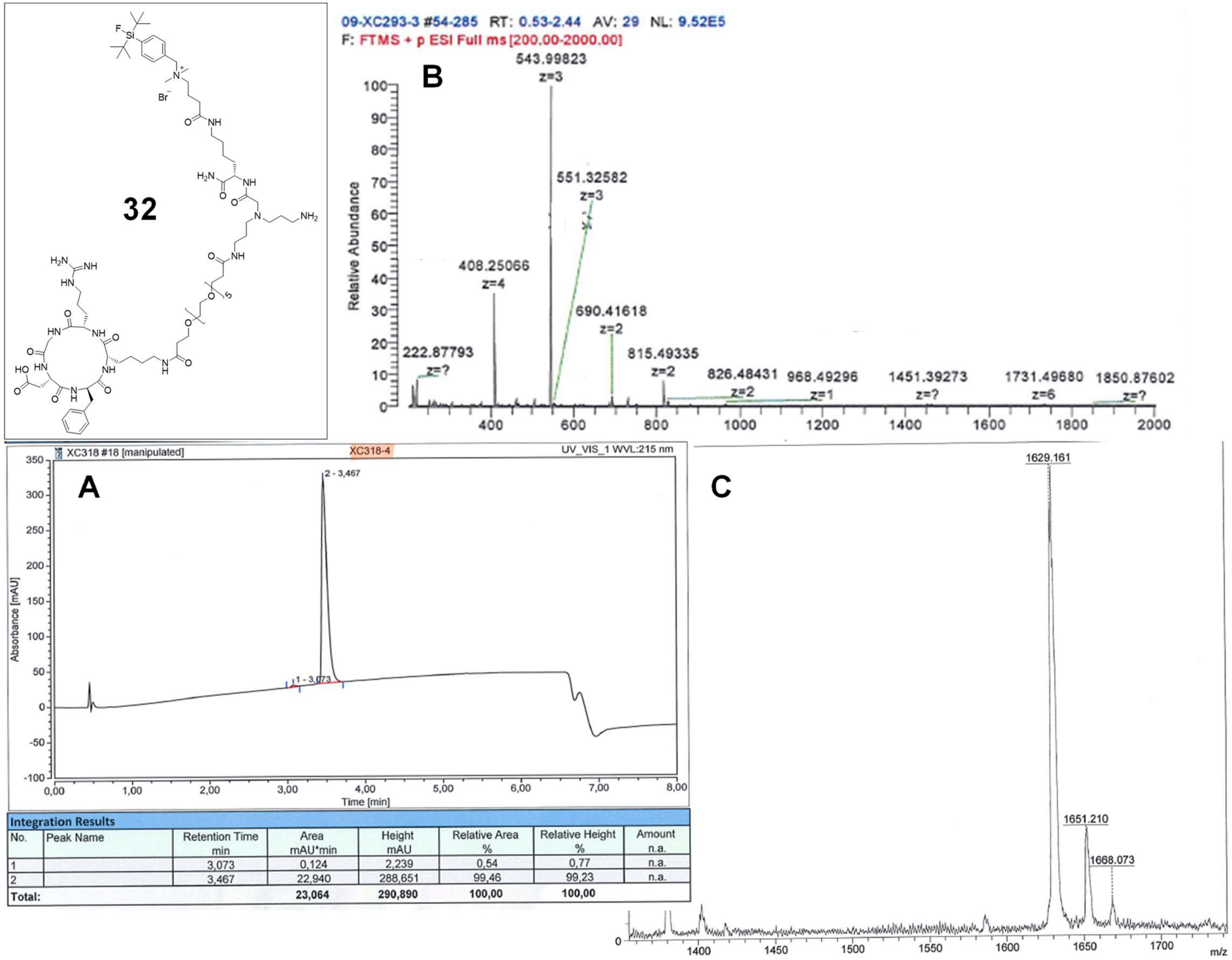

Integration Results

No.	Peak Name	Retention Time min	Area mAU*min	Height mAU	Relative Area %	Relative Height %	Amount n.a.
1		3,073	0,124	2,239	0,54	0,77	n.a.
2		3,467	22,940	288,651	99,46	99,23	n.a.
**Total:**			**23,064**	**290,890**	**100,00**	**100,00**	

Figure S49. HPLC chromatogram (**A**) and mass spectra (**B**: ESI, **C**: MALDI) of SiFA*lin*-APG-PEG$_5$-c(RGDfK) (**32**).

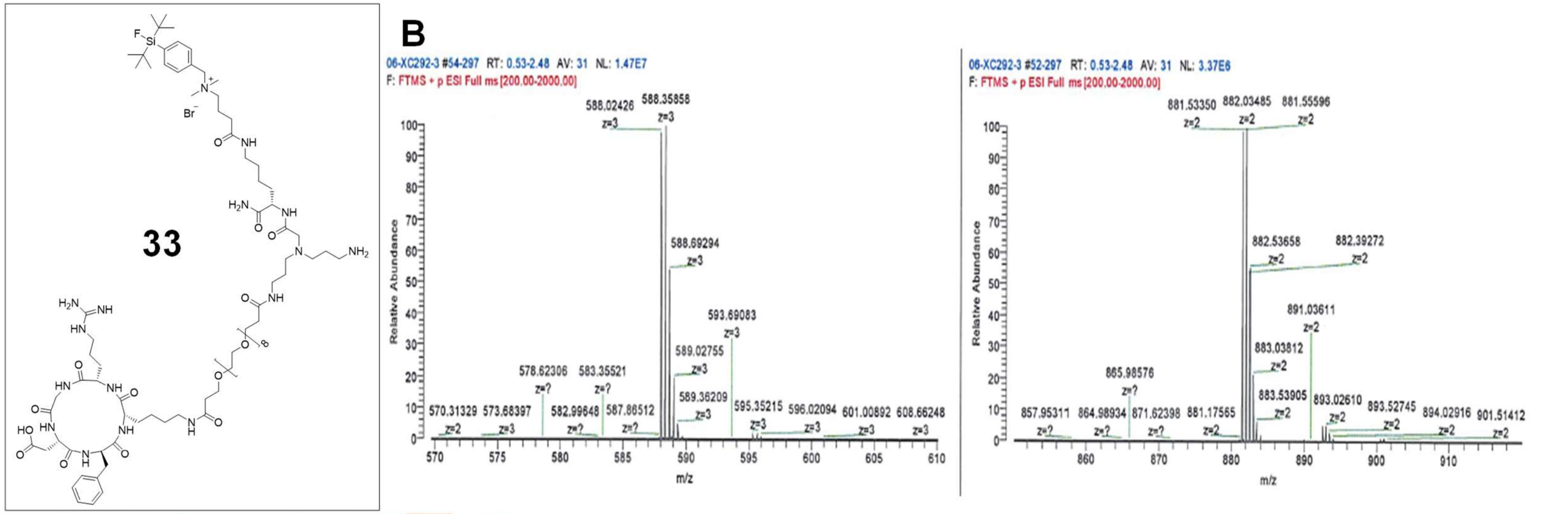

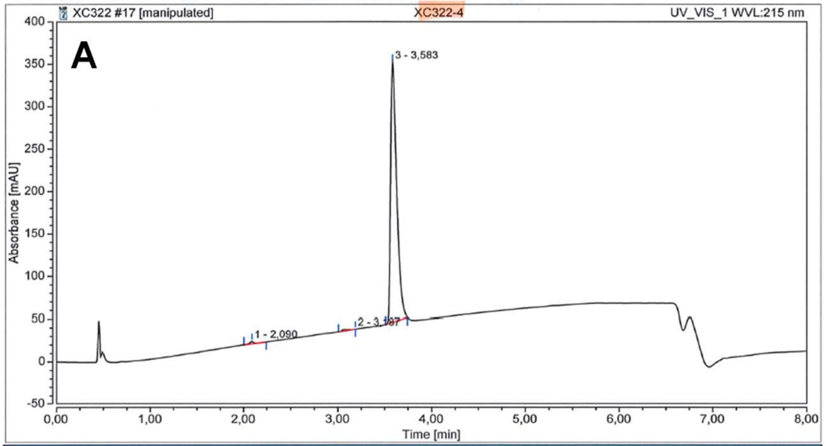

Integration Results							
No.	Peak Name	Retention Time min	Area mAU*min	Height mAU	Relative Area %	Relative Height %	Amount n.a.
1		2,090	0,113	2,808	0,49	0,91	n.a.
2		3,187	0,106	0,000	0,45	0,00	n.a.
3		3,583	23,099	306,116	99,06	99,09	n.a.
**Total:**			**23,318**	**308,924**	**100,00**	**100,00**	

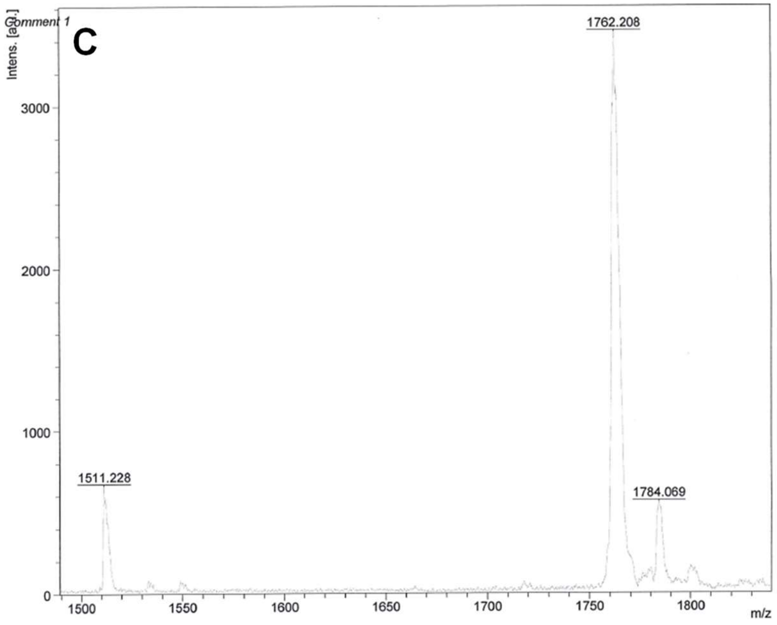

Figure S50. HPLC chromatogram (**A**) and mass spectra (**B**: ESI, **C**: MALDI) of SiFA*lin*-APG-PEG$_8$-c(RGDfK) (**33**).

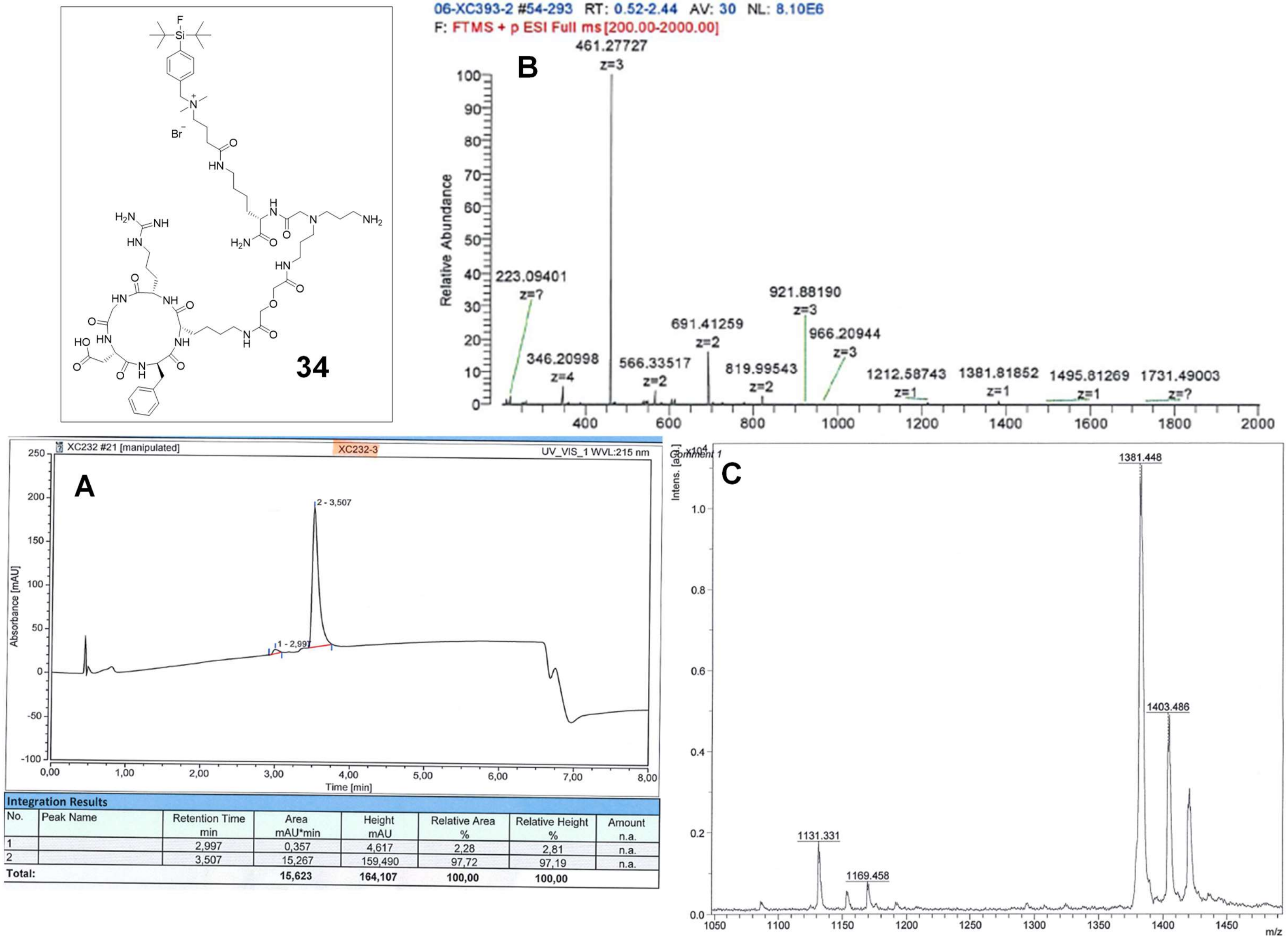

Integration Results

No.	Peak Name	Retention Time min	Area mAU*min	Height mAU	Relative Area %	Relative Height %	Amount n.a.
1		2,997	0,357	4,617	2,28	2,81	n.a.
2		3,507	15,267	159,490	97,72	97,19	n.a.
Total:			15,623	164,107	100,00	100,00	

Figure S51. HPLC chromatogram (**A**) and mass spectra (**B**: ESI, **C**: MALDI) of SiFA*lin*-APG-DIG-c(RGDfK) (**34**).

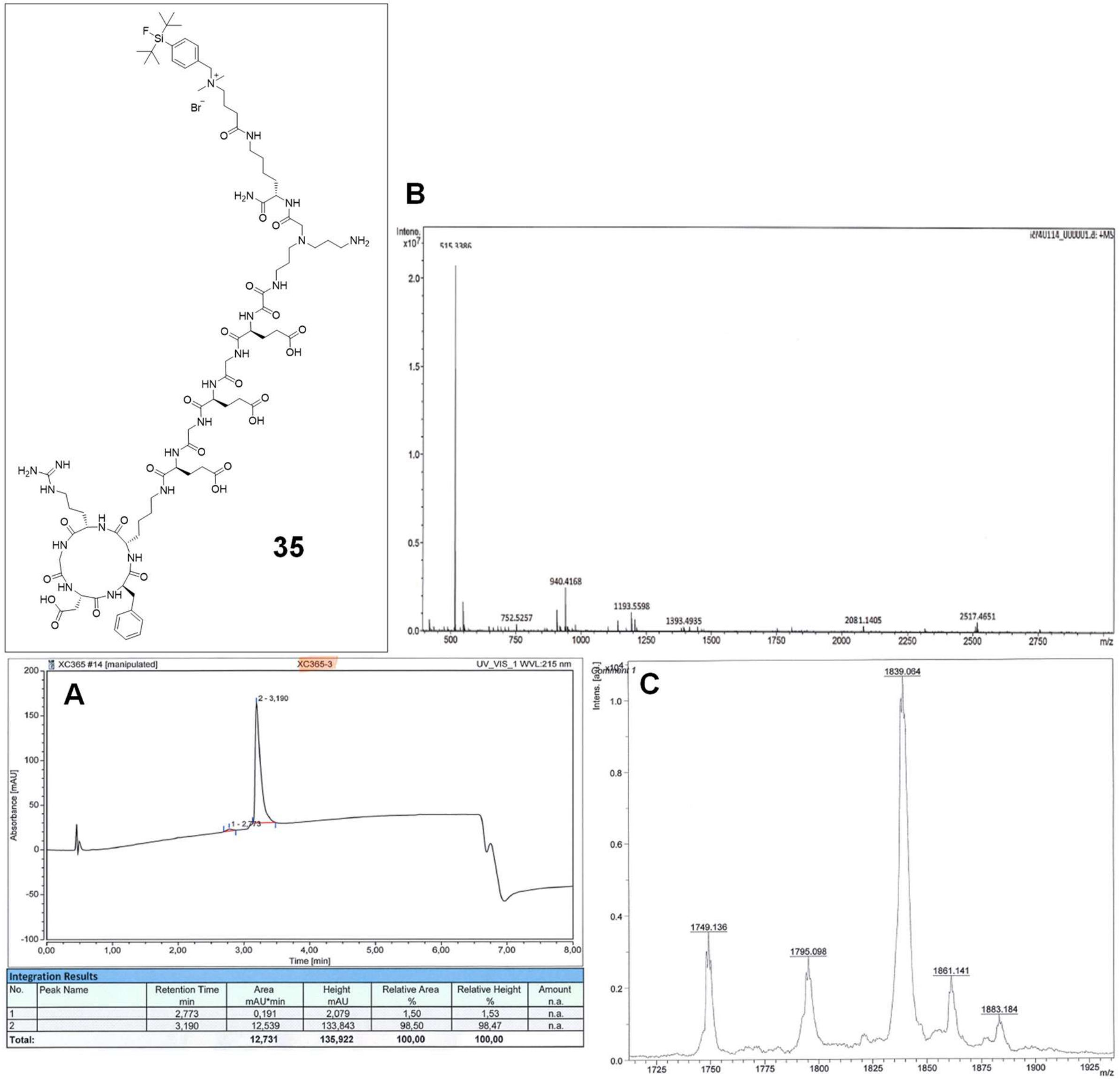

Integration Results							
No.	Peak Name	Retention Time min	Area mAU*min	Height mAU	Relative Area %	Relative Height %	Amount n.a.
1		2,773	0,191	2,079	1,50	1,53	n.a.
2		3,190	12,539	133,843	98,50	98,47	n.a.
Total:			12,731	135,922	100,00	100,00	

Figure S52. HPLC chromatogram (**A**) and mass spectra (**B**: ESI, **C**: MALDI) of SiFA*lin*-APG-Ox-EGEGE-c(RGDfK) (**35**).

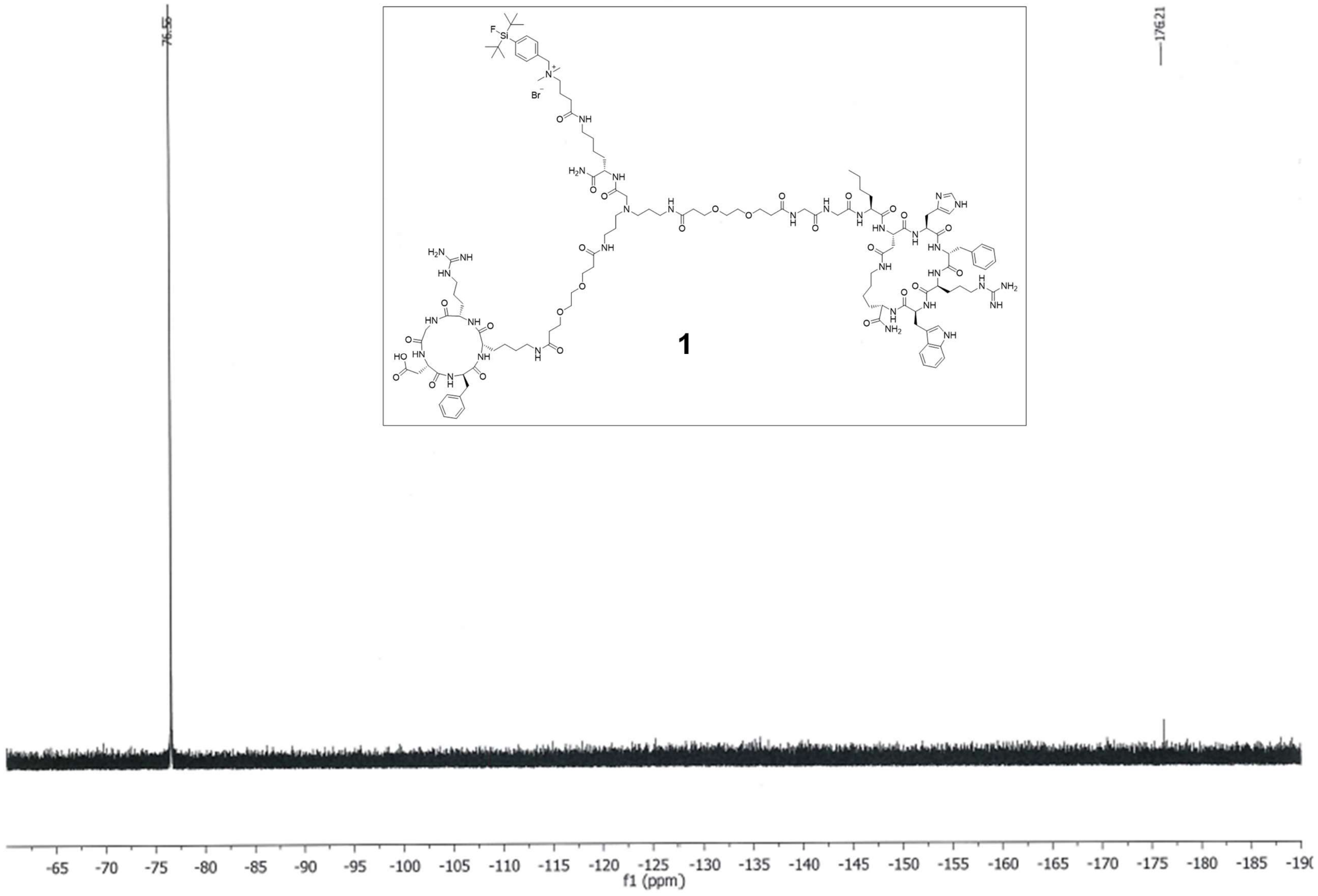

Figure S53. ^{19}F NMR spectrum of SiFA*lin*-APG-PEG$_1$-c(RGDfK)/GG-Nle-c(DHfRWK) (**1**).

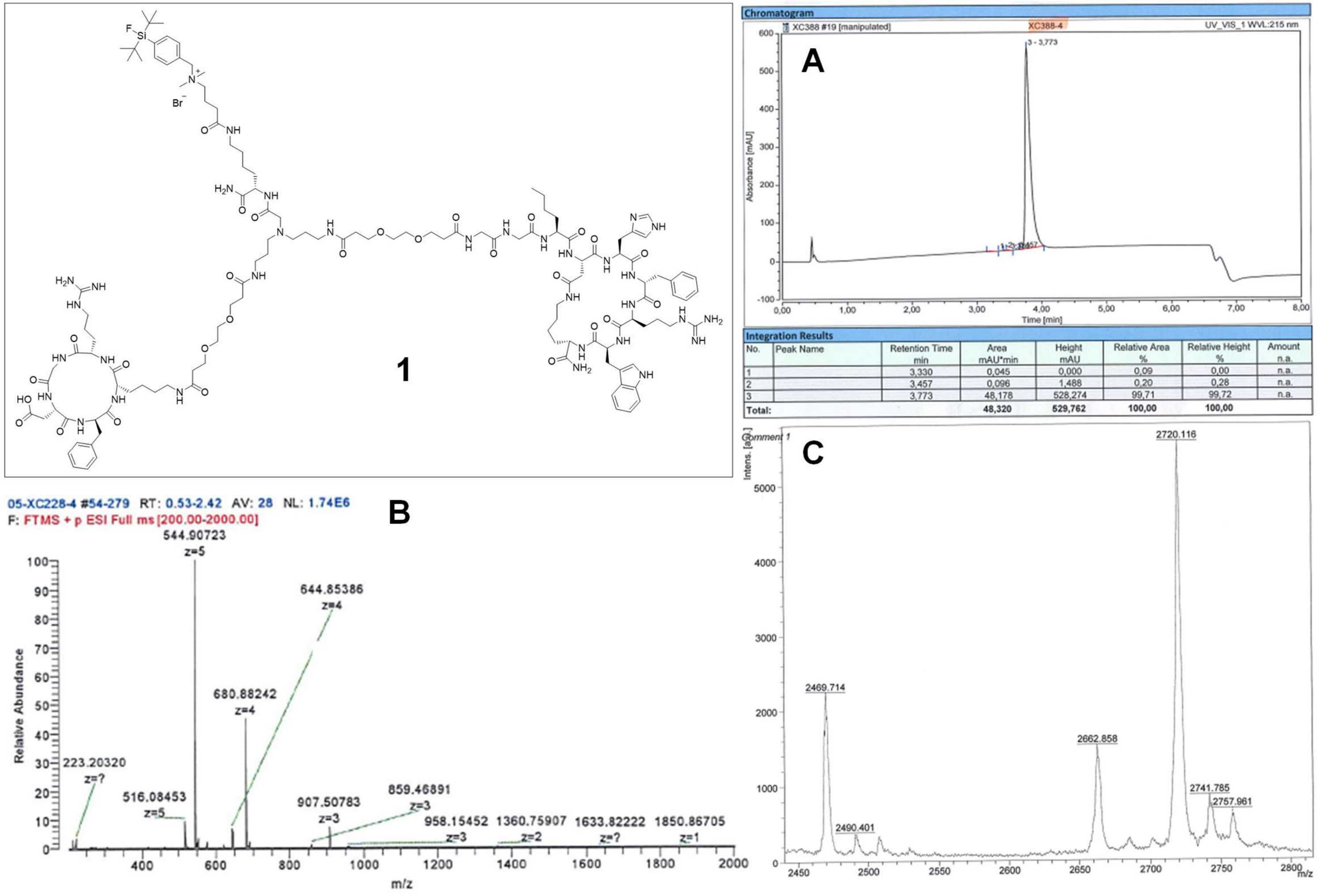

Integration Results

No.	Peak Name	Retention Time min	Area mAU*min	Height mAU	Relative Area %	Relative Height %	Amount n.a.
1		3,330	0,045	0,000	0,09	0,00	n.a.
2		3,457	0,096	1,488	0,20	0,28	n.a.
3		3,773	48,178	528,274	99,71	99,72	n.a.
**Total:**			**48,320**	**529,762**	**100,00**	**100,00**	

Figure S54. HPLC chromatogram (**A**) and mass spectra (**B**: ESI, **C**: MALDI) of SiFA*lin*-APG-PEG$_1$-c(RGDfK)/GG-Nle-c(DHfRWK) (**1**).

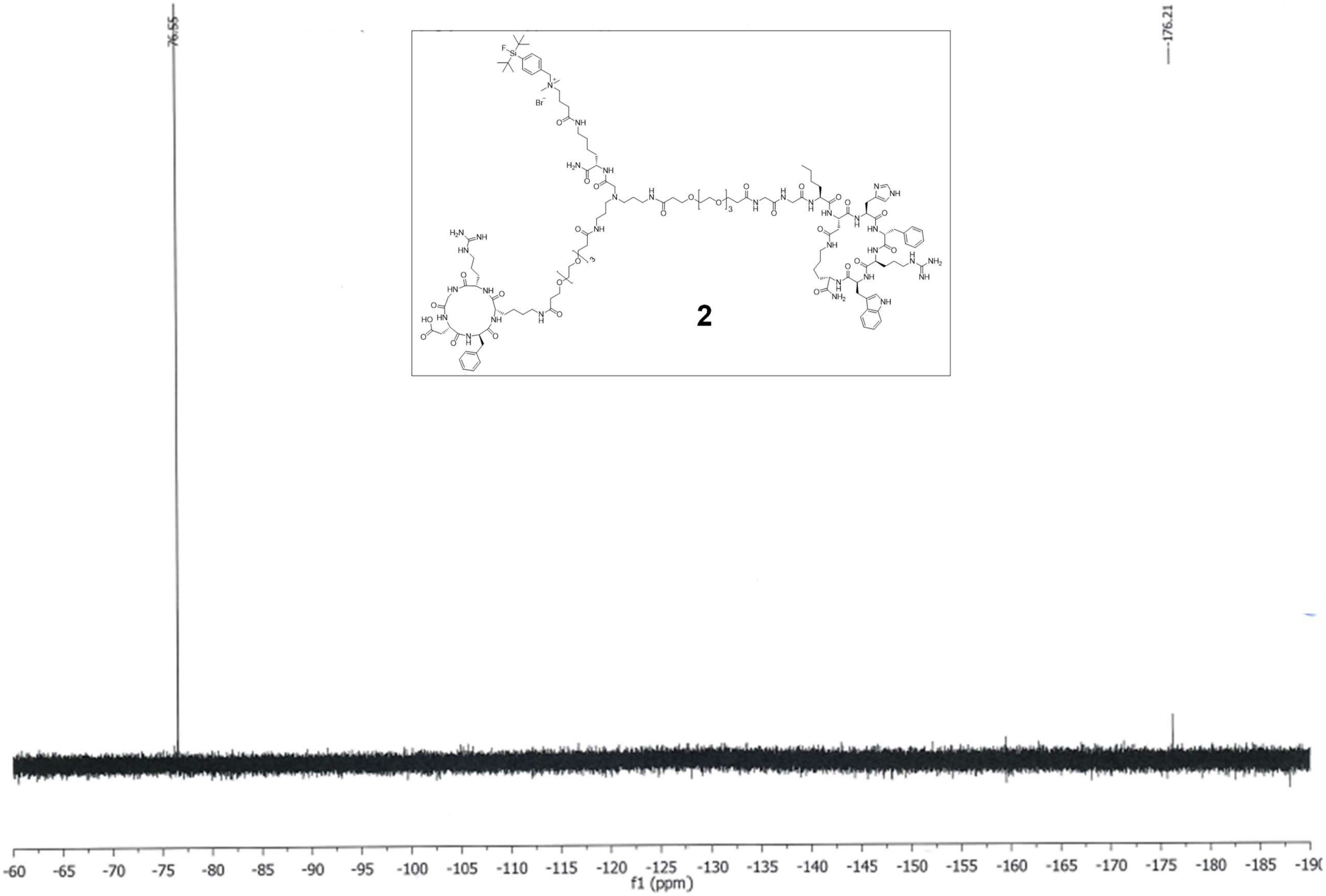

Figure S55. ^{19}F NMR spectrum of SiFA*lin*-APG-PEG$_3$-c(RGDfK)/GG-Nle-c(DHfRWK) (**2**).

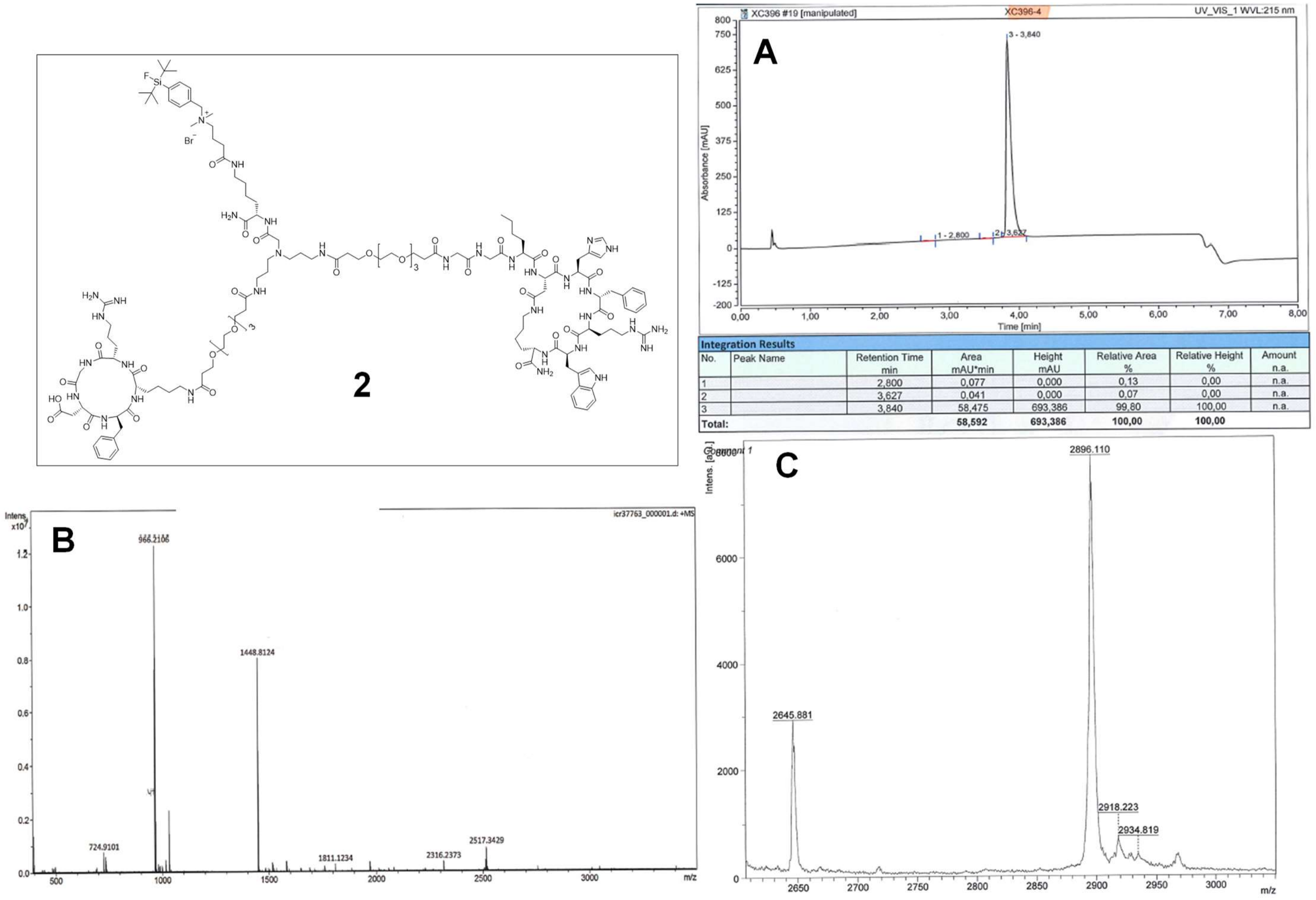

Integration Results							
No.	Peak Name	Retention Time min	Area mAU*min	Height mAU	Relative Area %	Relative Height %	Amount n.a.
1		2,800	0,077	0,000	0,13	0,00	n.a.
2		3,627	0,041	0,000	0,07	0,00	n.a.
3		3,840	58,475	693,386	99,80	100,00	n.a.
**Total:**			**58,592**	**693,386**	**100,00**	**100,00**	

Figure S56. HPLC chromatogram (**A**) and mass spectra (**B**: ESI, **C**: MALDI) of SiFA*lin*-APG-PEG$_3$-c(RGDfK)/GG-Nle-c(DHfRWK) (**2**).

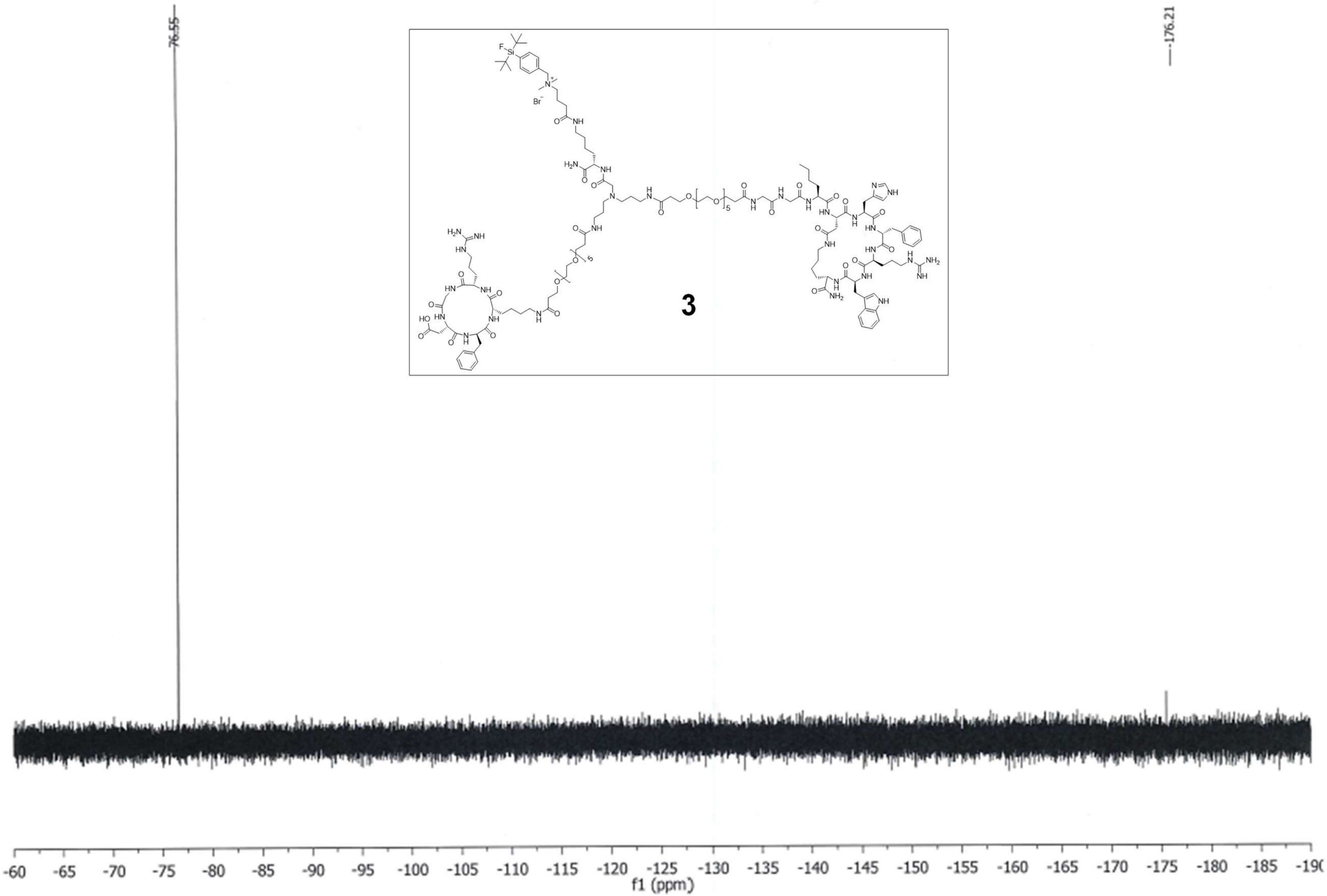

Figure S57. ^{19}F NMR spectrum of SiFA*lin*-APG-PEG$_5$-c(RGDfK)/GG-Nle-c(DHfRWK) (**3**).

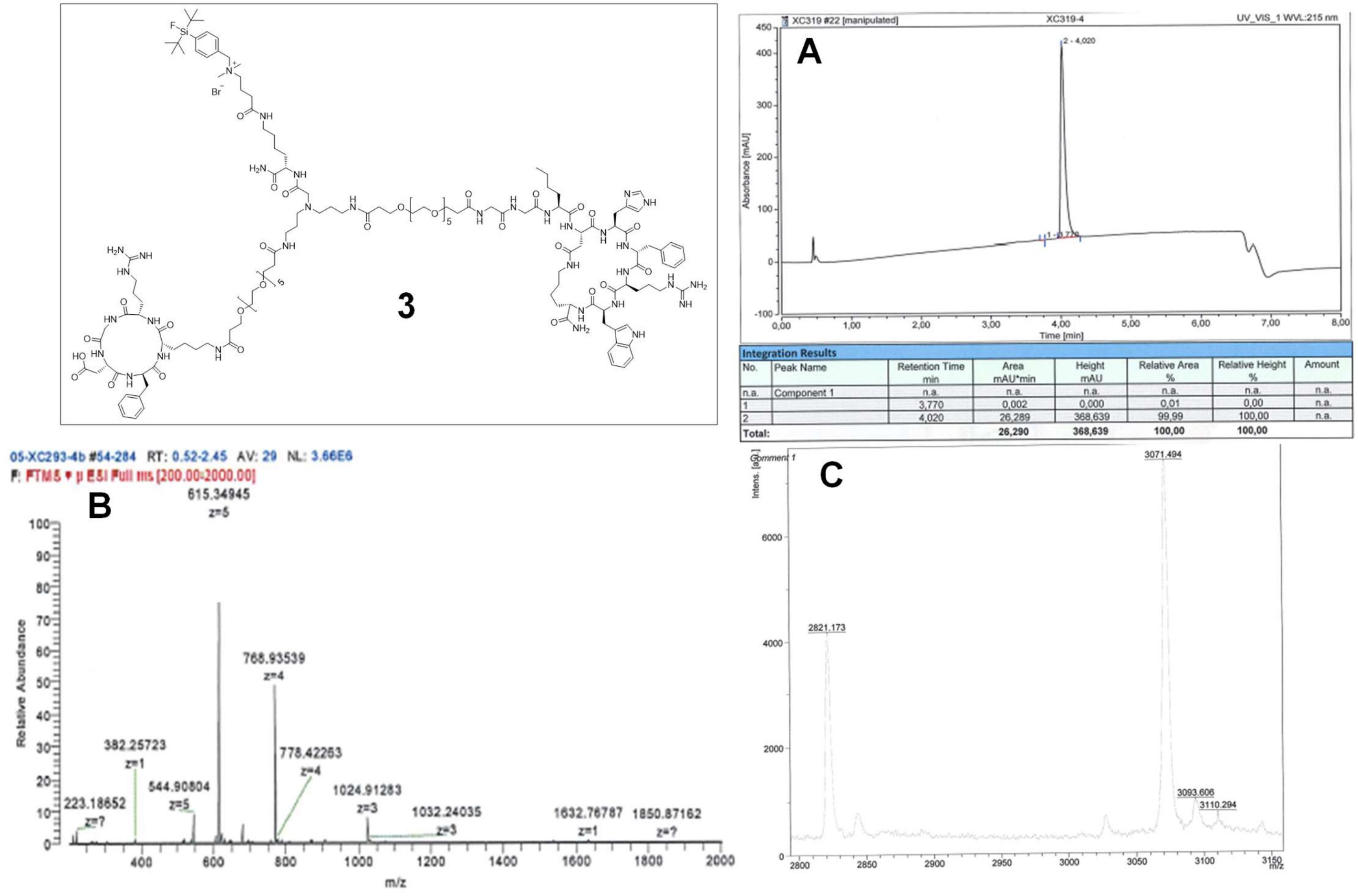

Integration Results							
No.	Peak Name	Retention Time min	Area mAU*min	Height mAU	Relative Area %	Relative Height %	Amount
n.a.	Component 1	n.a.	n.a.	n.a.	n.a.	n.a.	n.a.
1		3,770	0,002	0,000	0,01	0,00	n.a.
2		4,020	26,289	368,639	99,99	100,00	n.a.
Total:			26,290	368,639	100,00	100,00	

Figure S58. HPLC chromatogram (**A**) and mass spectra (**B**: ESI, **C**: MALDI) of SiFA*lin*-APG-PEG$_5$-c(RGDfK)/GG-Nle-c(DHfRWK) (**3**).

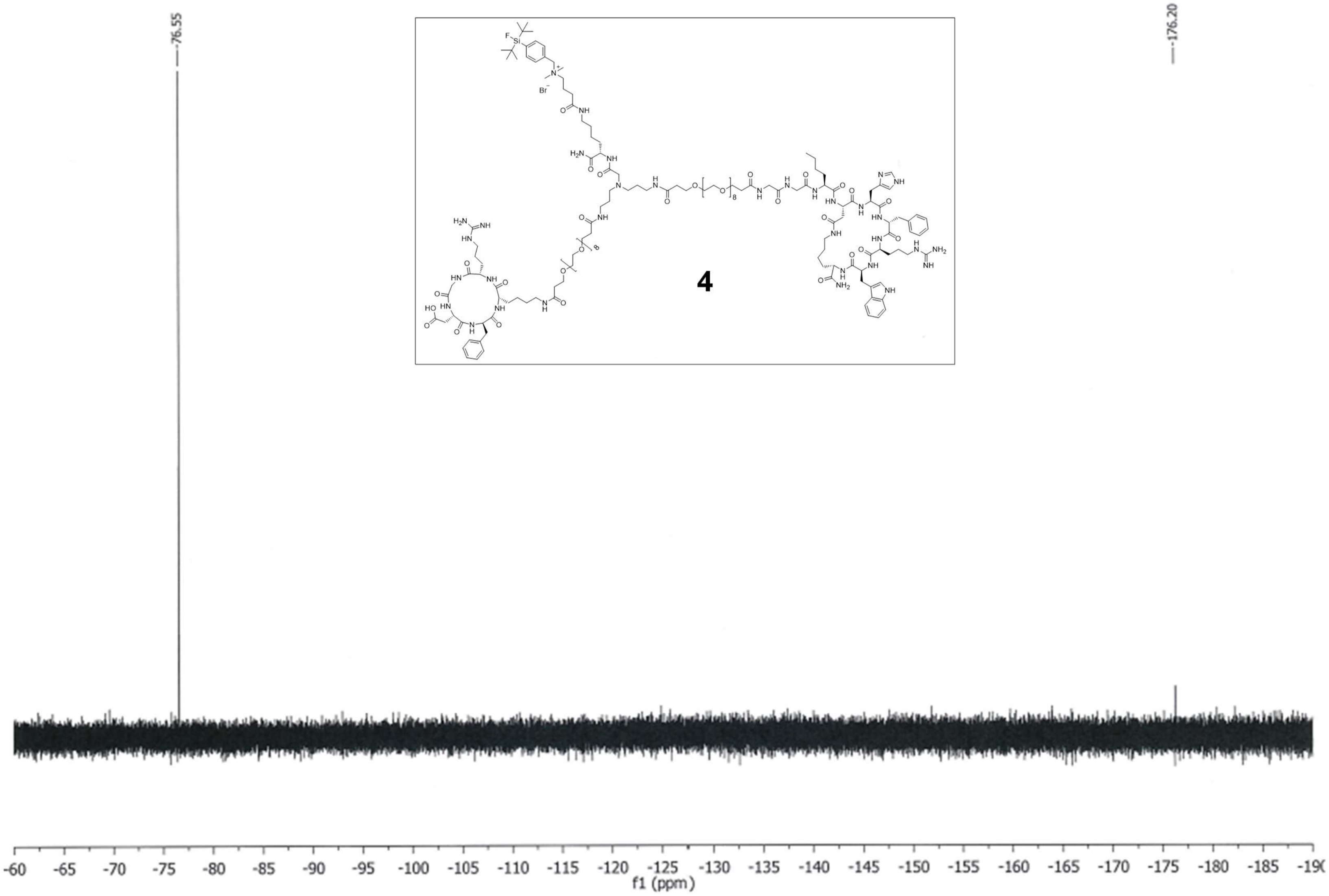

Figure S59. ^{19}F NMR spectrum of SiFA*lin*-APG-PEG$_8$-c(RGDfK)/GG-Nle-c(DHfRWK) (**4**).

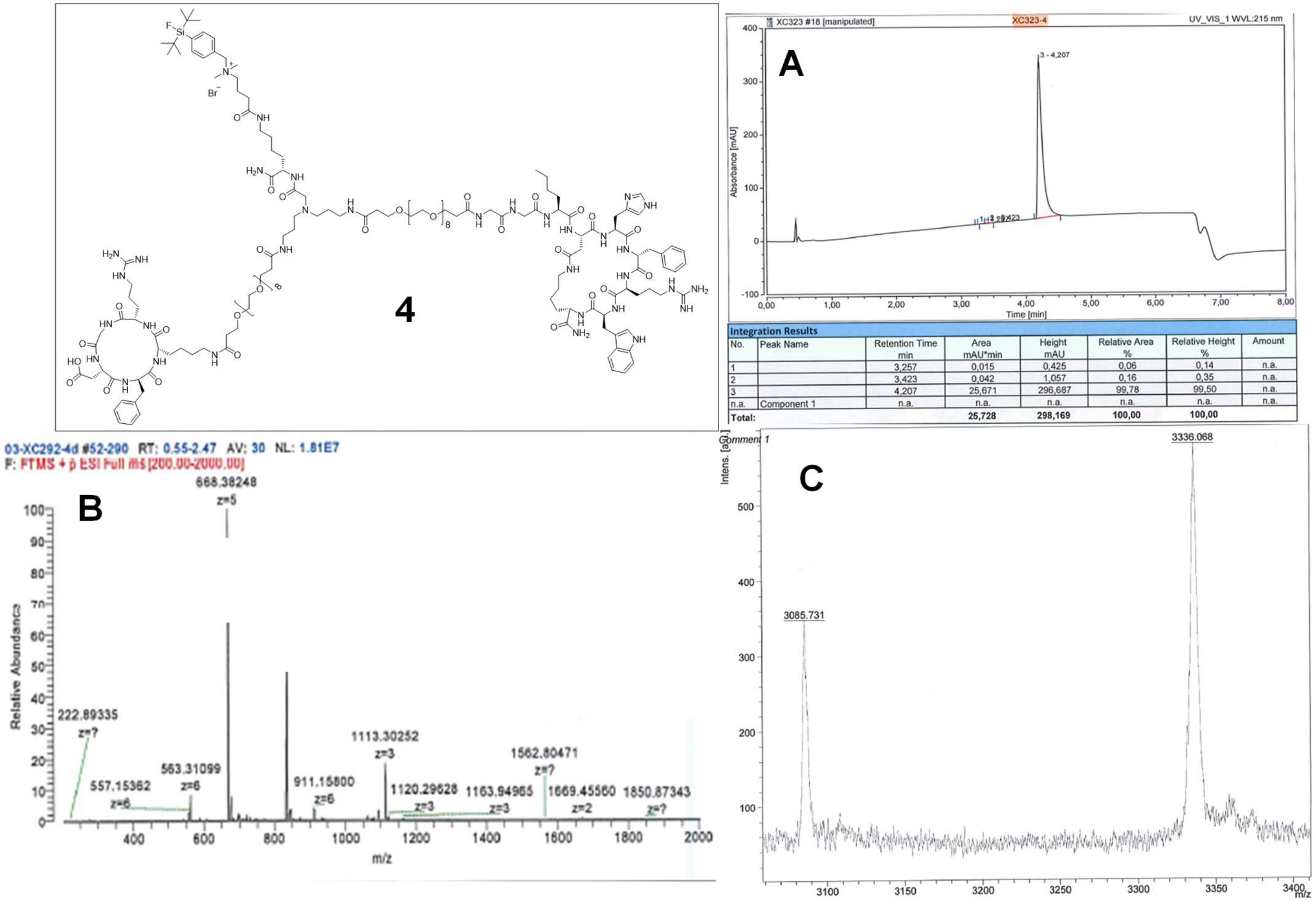

Integration Results							
No.	Peak Name	Retention Time min	Area mAU*min	Height mAU	Relative Area %	Relative Height %	Amount
1		3,257	0,015	0,425	0,06	0,14	n.a.
2		3,423	0,042	1,057	0,16	0,35	n.a.
3		4,207	25,671	296,687	99,78	99,50	n.a.
n.a.	Component 1	n.a.	n.a.	n.a.	n.a.	n.a.	n.a.
**Total:**			**25,728**	**298,169**	**100,00**	**100,00**	

Figure S60. HPLC chromatogram (**A**) and mass spectra (**B**: ESI, **C**: MALDI) of SiFA*lin*-APG-PEG$_8$-c(RGDfK)/GG-Nle-c(DHfRWK) (**4**).

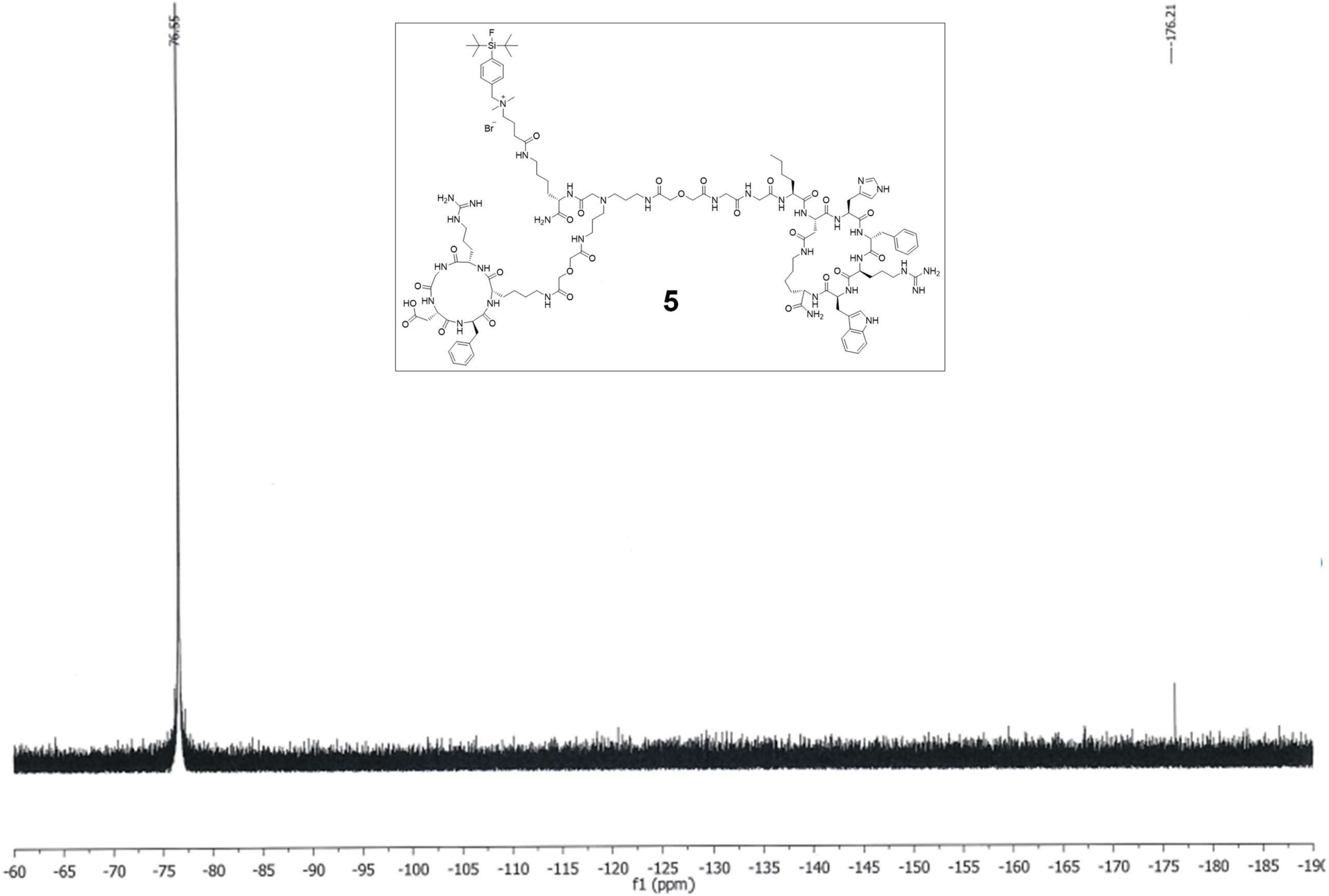

Figure S61. ^{19}F NMR spectrum of SiFA*lin*-APG-DIG-c(RGDfK)/GG-Nle-c(DHfRWK) (**5**).

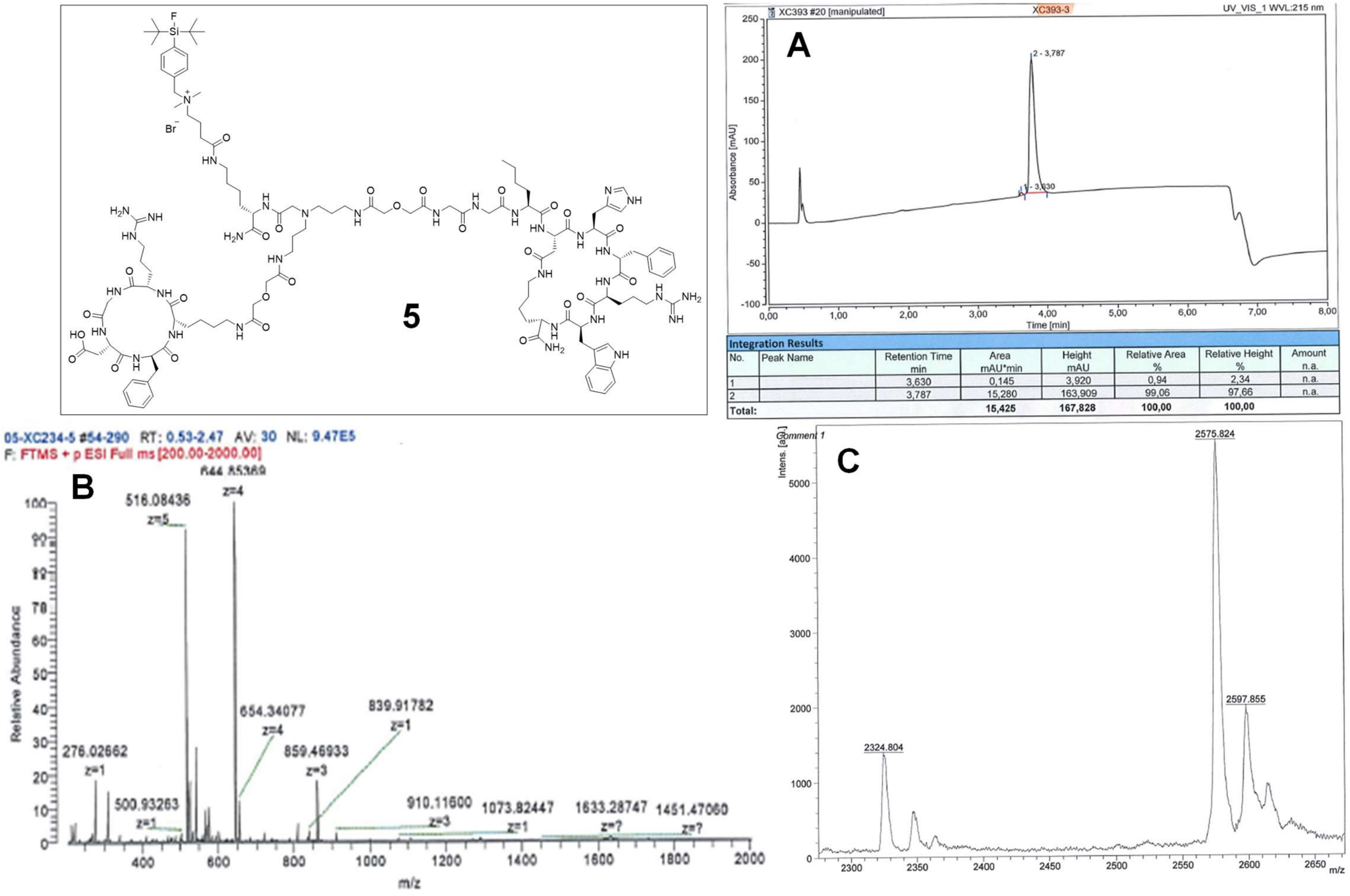

Integration Results

No.	Peak Name	Retention Time min	Area mAU*min	Height mAU	Relative Area %	Relative Height %	Amount n.a.
1		3,630	0,145	3,920	0,94	2,34	n.a.
2		3,787	15,280	163,909	99,06	97,66	n.a.
Total:			15,425	167,828	100,00	100,00	

Figure S62. HPLC chromatogram (**A**) and mass spectra (**B**: ESI, **C**: MALDI) of SiFA*lin*-APG-DIG-c(RGDfK)/GG-Nle-c(DHfRWK) (**5**).

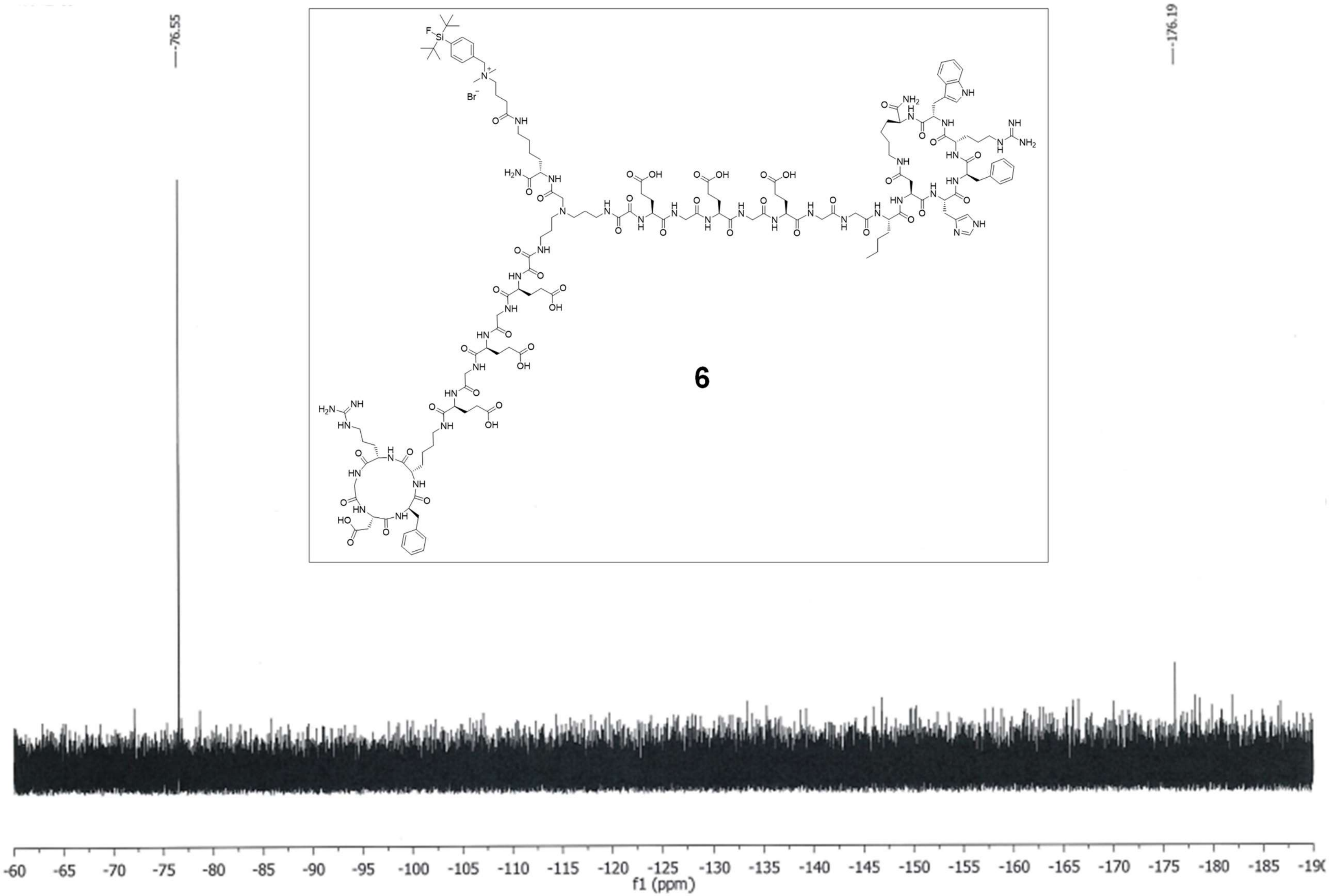

Figure S63. ^{19}F NMR spectrum of SiFA*lin*-APG-Ox-EGEGE-c(RGDfK)/GG-Nle-c(DHfRWK) (**6**).

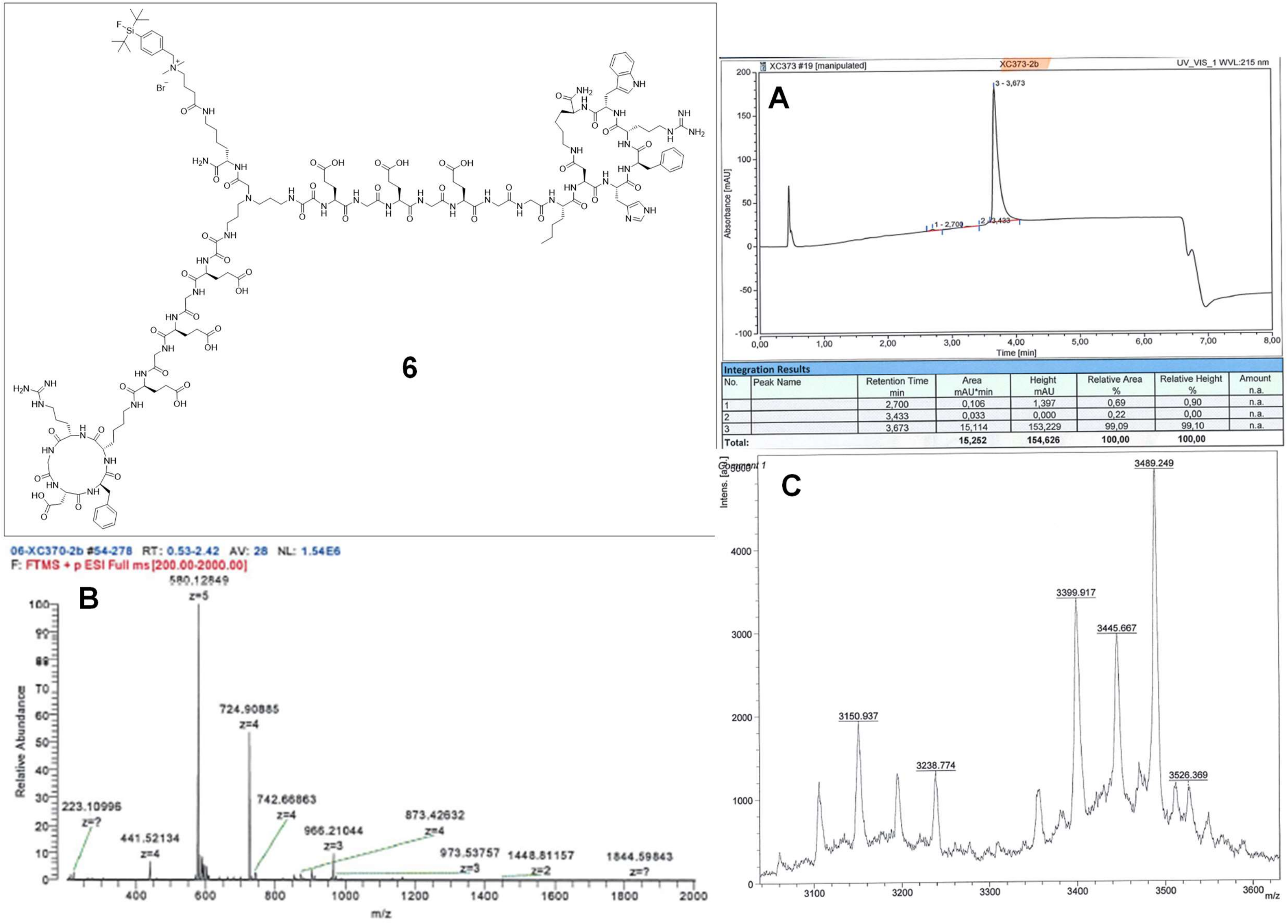

Integration Results							
No.	Peak Name	Retention Time min	Area mAU*min	Height mAU	Relative Area %	Relative Height %	Amount n.a.
1		2,700	0,106	1,397	0,69	0,90	n.a.
2		3,433	0,033	0,000	0,22	0,00	n.a.
3		3,673	15,114	153,229	99,09	99,10	n.a.
**Total:**			**15,252**	**154,626**	**100,00**	**100,00**	

Figure S64. HPLC chromatogram (**A**) and mass spectra (**B**: ESI, **C**: MALDI) of SiFA*lin*-APG-Ox-EGEGE-c(RGDfK)/GG-Nle-c(DHfRWK) (**6**).

4. Mass data, HPLC chromatograms and mass spectra of peptidic side products a–z

*HO-PEG$_1$-c(RGDfK) (**a**):* MS (MALDI) *m/z* calcd for $C_{35}H_{54}N_9O_{12}$ [*M*+H]$^+$ 792.39, found: 792.16; HRMS (ESI) *m/z* calcd for $C_{35}H_{54}N_9O_{12}$ [*M*+H]$^+$ 792.3886, found 792.3890.

*c(RGDfK)-PEG$_1$-c(RGDfK) (**b**):* MS (MALDI) *m/z* calcd for $C_{62}H_{92}N_{18}O_{18}$ [*M*]$^+$ 1376.68, found: 1376.51; HRMS (ESI) *m/z* calcd for $C_{62}H_{94}N_{18}O_{18}$ [*M*+2H]$^{2+}$ 689.3491, found 689.3506; calcd for $C_{62}H_{93}N_{18}O_{18}$ [*M*+H]$^+$ 1377.6910, found 1377.6972.

*HO-PEG$_3$-c(RGDfK) (**c**):* MS (MALDI) *m/z* calcd for $C_{39}H_{61}N_9O_{14}$ [*M*]$^+$ 879.43, found: 879.54; HRMS (ESI) *m/z* calcd for $C_{39}H_{62}N_9O_{14}$ [*M*+H]$^+$ 880.4411, found 880.4407.

*c(RGDfK)-PEG$_3$-c(RGDfK) (**d**):* MS (MALDI) *m/z* calcd for $C_{66}H_{100}N_{18}O_{20}$ [*M*]$^+$ 1464.74, found: 1464.78; HRMS (ESI) *m/z* calcd for $C_{66}H_{101}N_{18}O_{20}$ [*M*+H]$^+$ 1465.7434, found 1465.7475.

*HO-PEG$_5$-c(RGDfK) (**e**):* MS (MALDI) *m/z* calcd for $C_{43}H_{70}N_9O_{16}$ [*M*+H]$^+$ 968.49, found: 968.19; HRMS (ESI) *m/z* calcd for $C_{43}H_{70}N_9O_{16}$ [*M*+H]$^+$ 968.4935, found 968.4941.

*c(RGDfK)-PEG$_5$-c(RGDfK) (**f**):* MS (MALDI) *m/z* calcd for $C_{70}H_{109}N_{18}O_{22}$ [*M*]$^+$ 1553.80, found: 1553.12; HRMS (ESI) *m/z* calcd for $C_{70}H_{110}N_{18}O_{22}$ [*M*+2H]$^{2+}$ 777.4016, found 777.4054; calcd for $C_{70}H_{109}N_{18}O_{22}$ [*M*+H]$^+$ 1553.7958, found 1553.8101.

*HO-PEG$_8$-c(RGDfK) (**g**):* MS (MALDI) *m/z* calcd for $C_{49}H_{82}N_9O_{19}$ [*M*+H]$^+$ 1100.57, found: 1100.24; HRMS (ESI) *m/z* calcd for $C_{49}H_{82}N_9O_{19}$ [*M*+H]$^+$ 1100.5721, found 1100.5722.

*c(RGDfK)-PEG$_8$-c(RGDfK) (**h**):* MS (MALDI) *m/z* calcd for $C_{76}H_{121}N_{18}O_{25}$ [*M*+H]$^+$ 1685.87, found: 1685.15; HRMS (ESI) *m/z* calcd for $C_{76}H_{121}N_{18}NaO_{25}$ [*M*+H+Na]$^{2+}$ 854.4318, found 854.4318; calcd for $C_{76}H_{121}N_{18}O_{25}$ [*M*+H]$^+$ 1685.8745, found 1685.8771.

*HO-PEG$_1$-GG-Nle-c(DHfRWK) (**i**):* MS (MALDI) *m/z* calcd for $C_{60}H_{86}N_{17}O_{15}$ [*M*+H]$^+$ 1284.65, found: 1284.15; HRMS (ESI) *m/z* calcd for $C_{60}H_{87}N_{17}O_{15}$ [*M*+H]$^{2+}$ 642.8278, found 642.8286; calcd for $C_{60}H_{86}N_{17}O_{15}$ [*M*+H]$^+$: 1284.6517, found 1284.6534.

*c(DHfRWK)-Nle-GG-PEG$_1$-GG-Nle-c(DHfRWK) (**j**):* MS (MALDI) *m/z* calcd for $C_{112}H_{157}N_{34}O_{24}$ [*M*+H]$^+$ 2362.21, found: 2362.06; HRMS (ESI) *m/z* calcd for $C_{112}H_{159}N_{34}O_{24}$ [*M*+3H]$^{3+}$ 788.4095, found 788.4087; calcd for $C_{112}H_{158}N_{34}O_{24}$ [*M*+2H]$^{2+}$ 1182.1105, found 1182.1107.

*HO-PEG$_3$-GG-Nle-c(DHfRWK) (**k**):* MS (MALDI) *m/z* calcd for $C_{64}H_{94}N_{17}O_{17}$ [*M*+H]$^+$ 1372.70, found: 1372.49; HRMS (ESI) *m/z* calcd for $C_{64}H_{94}N_{17}O_{17}$ [*M*+H]$^{2+}$ 686.3501, found 686.3624; calcd for $C_{64}H_{94}N_{17}O_{17}$ [*M*+H]$^+$ 1372.7008, found 1372.7041.

*c(DHfRWK)-Nle-GG-PEG$_3$-GG-Nle-c(DHfRWK) (**l**):* MS (MALDI) *m/z* calcd for $C_{116}H_{165}N_{34}O_{26}$ [*M*+H]$^+$ 2450.26, found: 2450.78; HRMS (ESI) *m/z* calcd for $C_{116}H_{166}N_{34}O_{26}$ [*M*+2H]$^{2+}$ 1226.1368, found 1226.1374.

*HO-PEG$_5$-GG-Nle-c(DHfRWK) (**m**):* MS (MALDI) *m/z* calcd for $C_{68}H_{101}N_{17}O_{19}$ [*M*]$^+$ 1459.75, found: 1459.03; HRMS (ESI) *m/z* calcd for $C_{68}H_{102}N_{17}O_{19}$ [*M*+H]$^+$ 1460.7532, found 1460.7528.

*c(DHfRWK)-Nle-GG-PEG$_5$-GG-Nle-c(DHfRWK) (**n**):* MS (MALDI) *m/z* calcd for $C_{120}H_{173}N_{34}O_{28}$ [*M*+H]$^+$ 2538.32, found: 2538.09; HRMS (ESI) *m/z* calcd for $C_{120}H_{175}N_{34}O_{28}$ [*M*+3H]$^{3+}$ 847.1111, found 847.1110; calcd for $C_{120}H_{174}N_{34}O_{28}$ [*M*+2H]$^{2+}$ 1270.1630, found 1270.1641.

*HO-PEG$_8$-GG-Nle-c(DHfRWK) (**o**):* MS (MALDI) *m/z* calcd for $C_{74}H_{114}N_{17}O_{22}$ [*M*+H]$^+$ 1592.83, found: 1592.11; HRMS (ESI) *m/z* calcd for $C_{74}H_{114}N_{17}O_{22}$ [*M*+H]$^+$ 1592.8319, found 1592.8321.

*c(DHfRWK)-Nle-GG-PEG$_8$-GG-Nle-c(DHfRWK) (**p**):* MS (MALDI) *m/z* calcd for $C_{126}H_{185}N_{34}O_{31}$ [*M*+H]$^+$ 2670.39, found: 2670.33; HRMS (ESI) *m/z* calcd for $C_{126}H_{188}N_{34}O_{31}$ [*M*+4H]$^{4+}$ 668.6048, found 668. 6048; calcd for $C_{126}H_{187}N_{34}O_{31}$ [*M*+3H]$^{3+}$ 891.1373, found 891.1372; calcd for $C_{126}H_{186}N_{34}O_{31}$ [*M*+2H]$^{2+}$ 1336.2023, found 1336.2039.

*c(RGDfK)-DIG-c(RGDfK) (**q**):* MS (MALDI) *m/z* calcd for $C_{58}H_{85}N_{18}O_{17}$ [*M*+H]$^+$ 1305.63, found: 1305.49; HRMS (ESI) *m/z* calcd for $C_{58}H_{86}N_{18}O_{17}$ [*M*+2H]$^{2+}$ 653.3204, found 653.3207; calcd for $C_{58}H_{85}N_{18}O_{17}$ [*M*+H]$^+$ 1305.6335, found 1305.6356.

*c(RGDfK)-EGEGE-Ox-EGEGE-c(RGDfK) (**r**):* MS (MALDI) *m/z* calcd for $C_{58}H_{85}N_{18}O_{17}$ [*M*+H]$^+$ 2263.95, found: 2263.82; HRMS (ESI) *m/z* calcd for $C_{94}H_{136}N_{28}O_{38}$ [*M*+2H]$^{2+}$ 1132.9796, found 1132.9793.

*c(DHfRWK)-Nle-GG-DIG-GG-Nle-c(DHfRWK) (**s**):* MS (MALDI) *m/z* calcd for $C_{108}H_{148}N_{34}O_{23}$ [*M*]$^+$ 2289.15, found: 2289.51; HRMS (ESI) *m/z* calcd for $C_{108}H_{151}N_{34}O_{23}$ [*M*+3H]$^{3+}$ 764.3903, found 764.3896; calcd for $C_{108}H_{150}N_{34}O_{23}$ [*M*+2H]$^{2+}$ 1146.0818, found 1146.0820.

*c(DHfRWK)-Nle-GG-EGEGE-Ox-EGEGE-GG-Nle-c(DHfRWK) (**t**):* MS (MALDI) *m/z* calcd for $C_{144}H_{199}N_{44}O_{44}$ [*M*+H]$^+$ 3248.47, found: 3248.29; HRMS (ESI) *m/z* calcd for $C_{144}H_{201}N_{44}O_{44}$ [*M*+3H]$^{3+}$ 1083.8287, found 1083.8283; calcd for $C_{144}H_{200}N_{44}O_{44}$ [*M*+2H]$^{2+}$ 1625.2394, found 1625.2388.

*SiFAlin-APG-[PEG$_1$-c(RGDfK)]$_2$ (**u**):* MS (MALDI) *m/z* calcd for $C_{105}H_{169}FN_{25}O_{25}Si$ [*M*]$^+$ 2227.25, found: 2227.78; HRMS (ESI) *m/z* calcd for $C_{105}H_{171}FN_{25}O_{25}Si$ [*M*+2H]$^{3+}$ 743.4216, found 743.4216; calcd for $C_{105}H_{170}FN_{25}O_{25}Si$ [*M*+H]$^{2+}$ 1114.6288, found 1114.6282.

*SiFAlin-APG-[PEG$_3$-c(RGDfK)]$_2$ (**v**):* MS (MALDI) *m/z* calcd for $C_{113}H_{186}FN_{25}O_{29}Si$ [*M*+H]$^+$ 2404.36, found: 2404.59; HRMS (ESI) *m/z* calcd for $C_{113}H_{187}FN_{25}O_{29}Si$ [*M*+2H]$^{3+}$ 802.1232, found 802.1231; calcd for $C_{113}H_{186}FN_{25}O_{29}Si$ [*M*+H]$^{2+}$ 1202.6812, found 1202.6806.

*SiFAlin-APG-[PEG$_5$-c(RGDfK)]$_2$ (**w**):* MS (MALDI) *m/z* calcd for $C_{121}H_{202}FN_{25}O_{33}Si$ [*M*+H]$^+$ 2580.46, found: 2580.38; HRMS (ESI) *m/z* calcd for $C_{121}H_{204}FN_{25}O_{33}Si$ [*M*+3H]$^{4+}$ 645.8705, found 645.8709; calcd for $C_{121}H_{203}FN_{25}O_{33}Si$ [*M*+2H]$^{3+}$ 860.8249, found 860.8249; calcd for $C_{121}H_{202}FN_{25}O_{33}Si$ [*M*+H]$^{2+}$ 1290.7336, found 1290.7334.

*SiFAlin-APG-[PEG$_8$-c(RGDfK)]$_2$ (**x**):* MS (MALDI) *m/z* calcd for $C_{133}H_{225}FN_{25}O_{39}Si$ [*M*+H]$^+$ 2843.61, found: 2843.59; HRMS (ESI) *m/z* calcd for $C_{133}H_{228}FN_{25}O_{39}Si$ [*M*+3H]$^{4+}$ 711.9098, found 711.9095; calcd for $C_{133}H_{227}FN_{25}O_{39}Si$ [*M*+2H]$^{3+}$ 948.8773, found 948.8771; calcd for $C_{133}H_{226}FN_{25}O_{39}Si$ [*M*+H]$^{2+}$ 1422.8123, found 1422.8132.

*SiFAlin-APG-[DIG-c(RGDfK)]$_2$ (**y**):* MS (MALDI) *m/z* calcd for $C_{97}H_{153}FN_{25}O_{23}Si$ [*M*]$^+$ 2083.13, found: 2083.43; HRMS (ESI) *m/z* calcd for $C_{97}H_{150}FN_{25}O_{23}Si$ [*M*-3H]$^+$ 2081.1118, found 2081.4647.

*SiFAlin-APG-[Ox-EGEGE-c(RGDfK)]$_2$ (**z**):* MS (MALDI) *m/z* calcd for $C_{131}H_{200}FN_{35}O_{43}Si$ [*M*+H]$^+$ 2998.43, found: 2998.28; HRMS (ESI) *m/z* calcd for $C_{123}H_{179}N_{35}O_{43}$ [*M*-$C_7H_{17}FSi$]$^+$ 1417.6459, found 1417.6307.

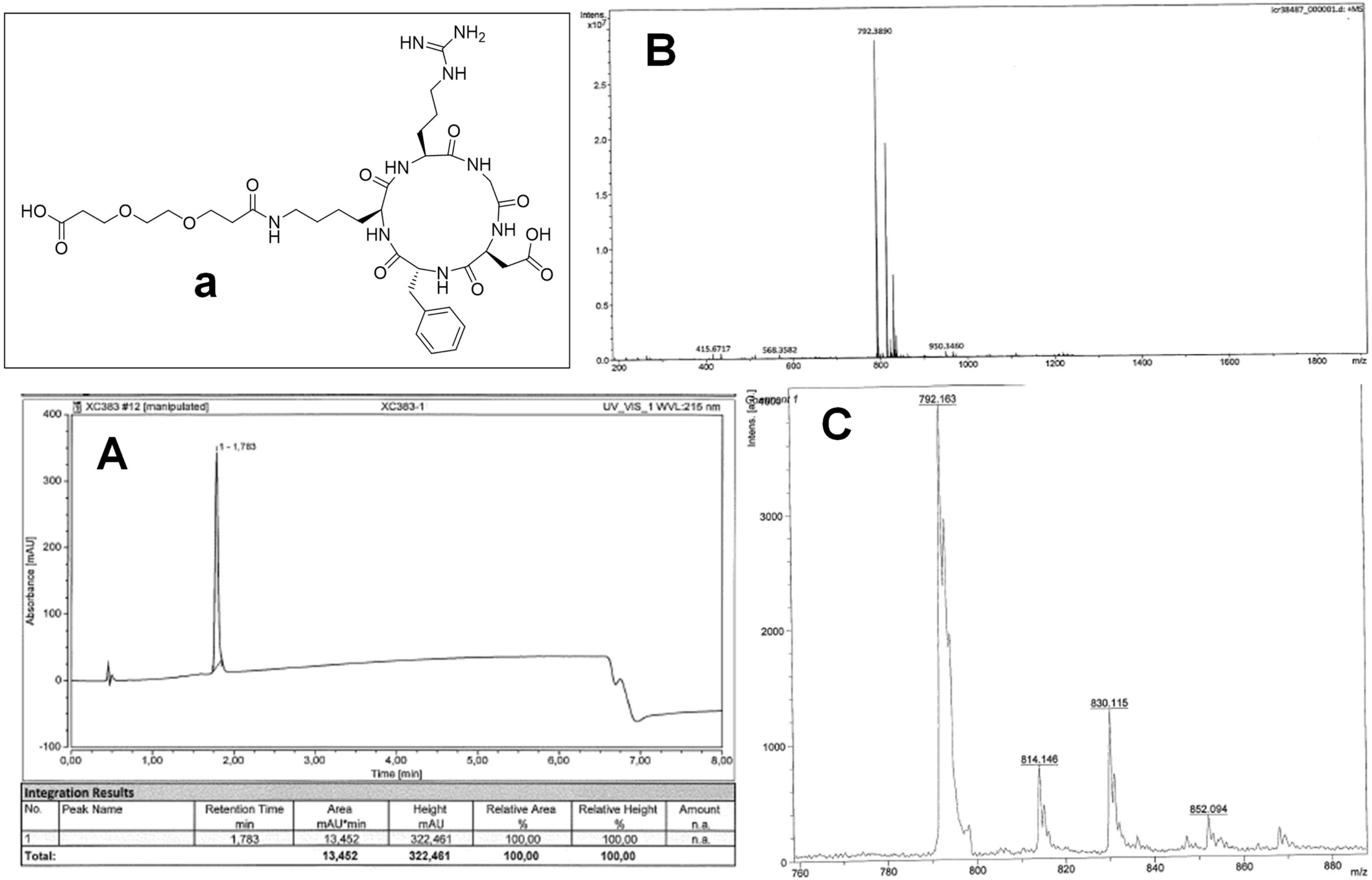

No.	Peak Name	Retention Time min	Area mAU*min	Height mAU	Relative Area %	Relative Height %	Amount n.a.
1		1,783	13,452	322,461	100,00	100,00	n.a.
Total:			13,452	322,461	100,00	100,00	

Figure S65. HPLC chromatogram (**A**) and mass spectra (**B**: ESI, **C**: MALDI) of HO-PEG$_1$-c(RGDfK) (**a**).

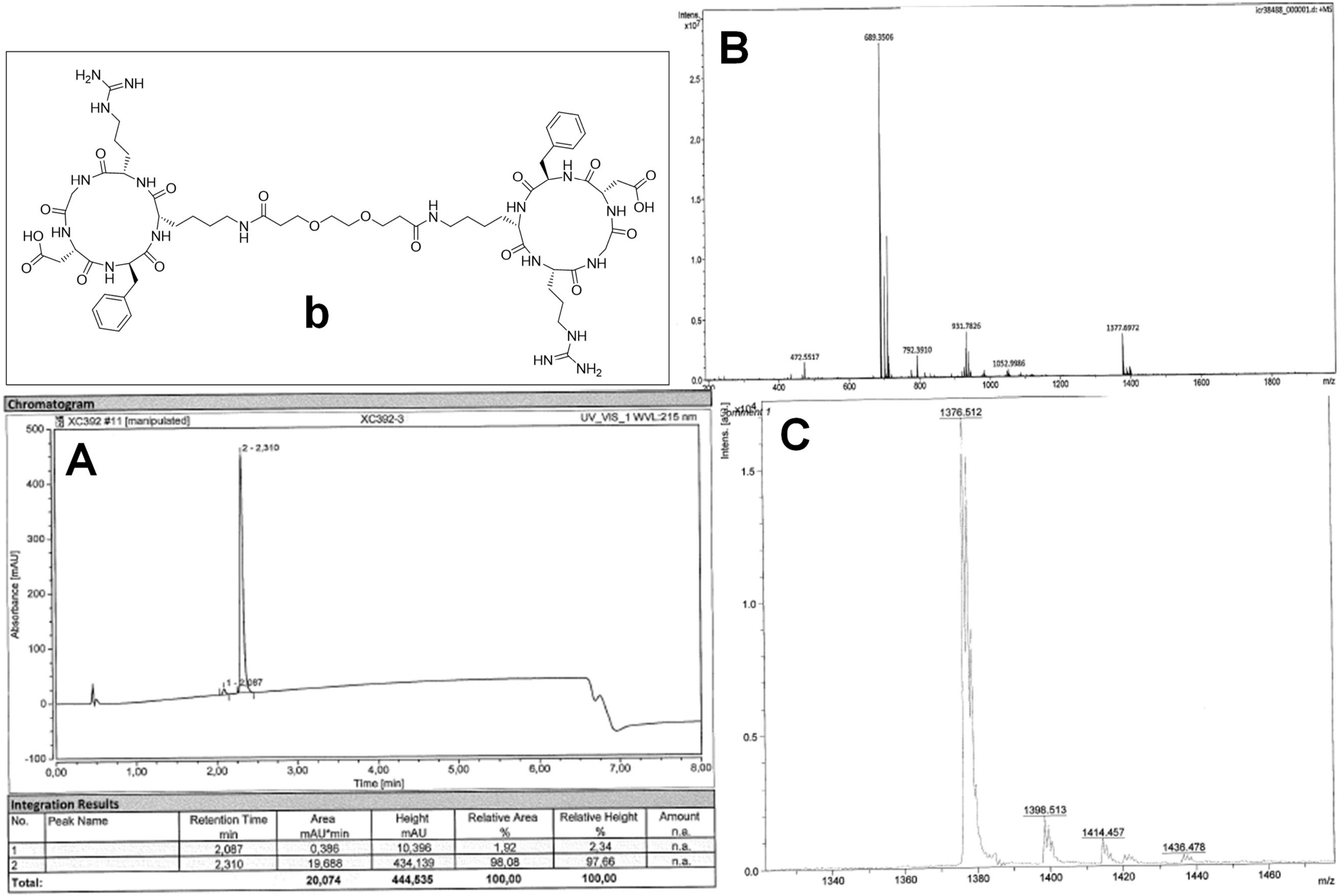

No.	Peak Name	Retention Time min	Area mAU*min	Height mAU	Relative Area %	Relative Height %	Amount n.a.
1		2,087	0,386	10,396	1,92	2,34	n.a.
2		2,310	19,688	434,139	98,08	97,66	n.a.
Total:			20,074	444,535	100,00	100,00	

Figure S66. HPLC chromatogram (**A**) and mass spectra (**B**: ESI, **C**: MALDI) of c(RGDfK)-PEG$_1$-c(RGDfK) (**b**).

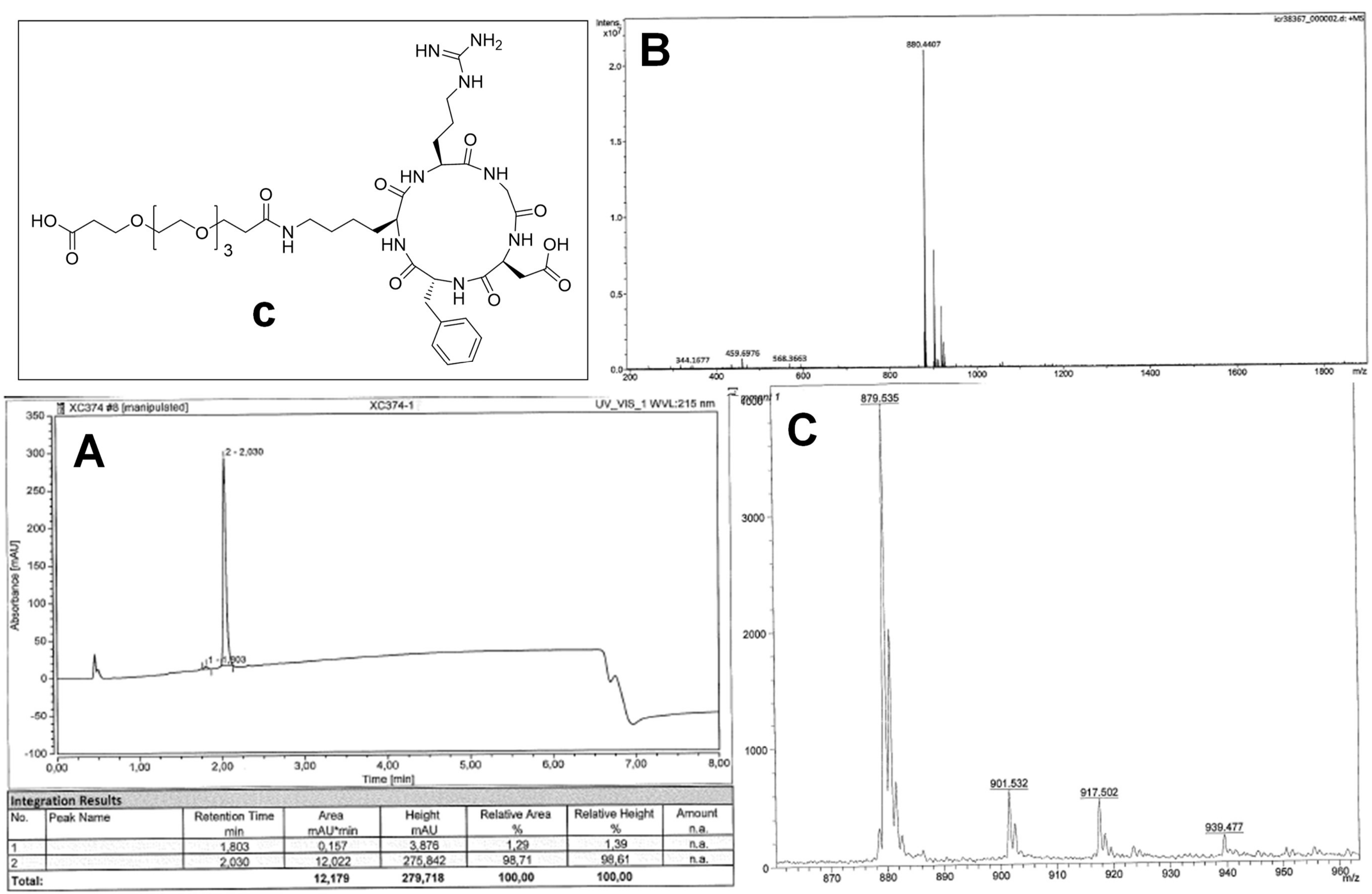

Integration Results							
No.	Peak Name	Retention Time min	Area mAU*min	Height mAU	Relative Area %	Relative Height %	Amount n.a.
1		1,803	0,157	3,876	1,29	1,39	n.a.
2		2,030	12,022	275,842	98,71	98,61	n.a.
Total:			12,179	279,718	100,00	100,00	

Figure S67. HPLC chromatogram (**A**) and mass spectra (**B**: ESI, **C**: MALDI) of HO-PEG$_3$-c(RGDfK) (**c**).

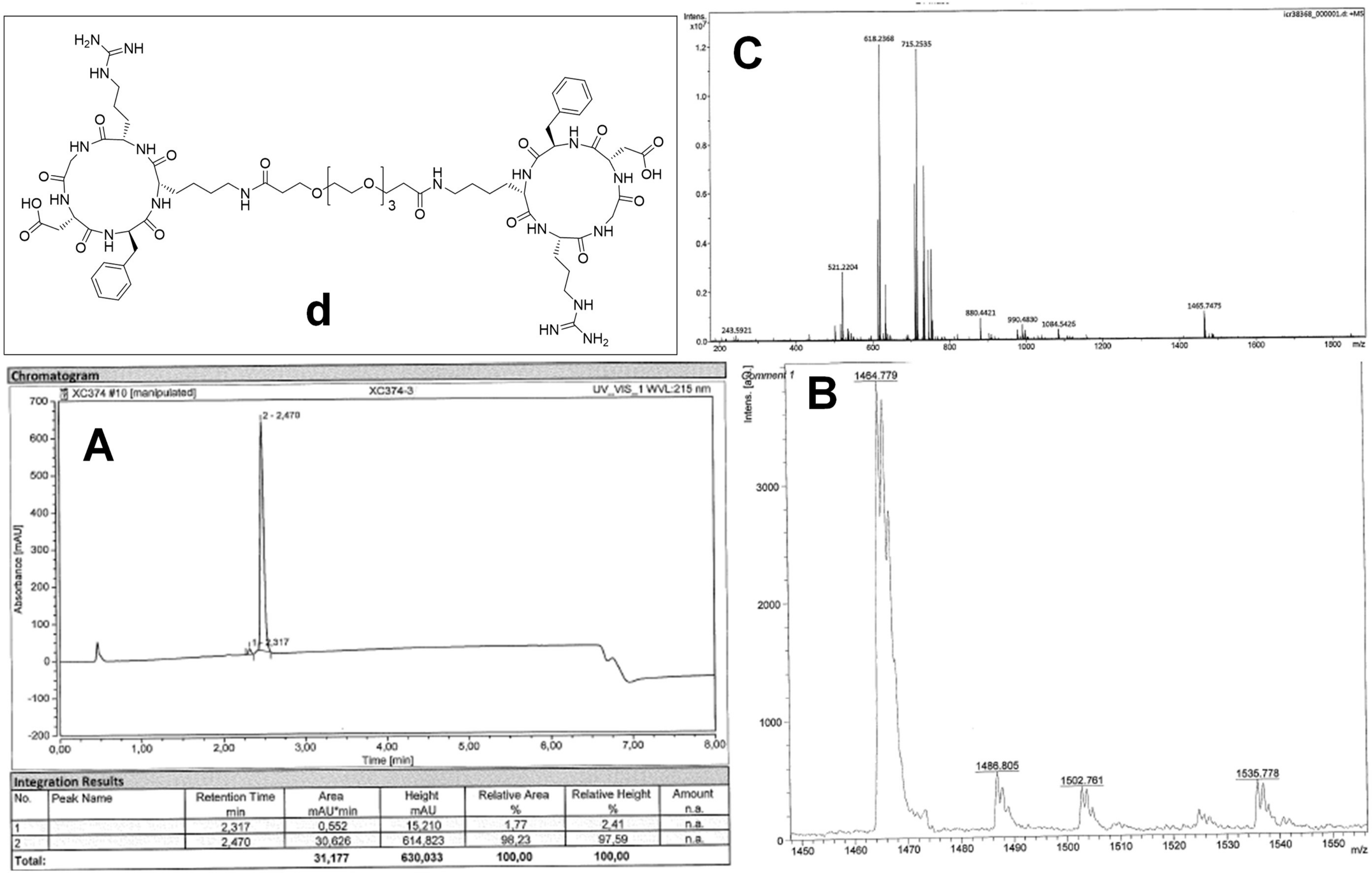

No.	Peak Name	Retention Time min	Area mAU*min	Height mAU	Relative Area %	Relative Height %	Amount n.a.
1		2,317	0,552	15,210	1,77	2,41	n.a.
2		2,470	30,626	614,823	98,23	97,59	n.a.
Total:			31,177	630,033	100,00	100,00	

Figure S68. HPLC chromatogram (**A**) and mass spectra (**B**: ESI, **C**: MALDI) of c(RGDfK)-PEG$_3$-c(RGDfK) (**d**).

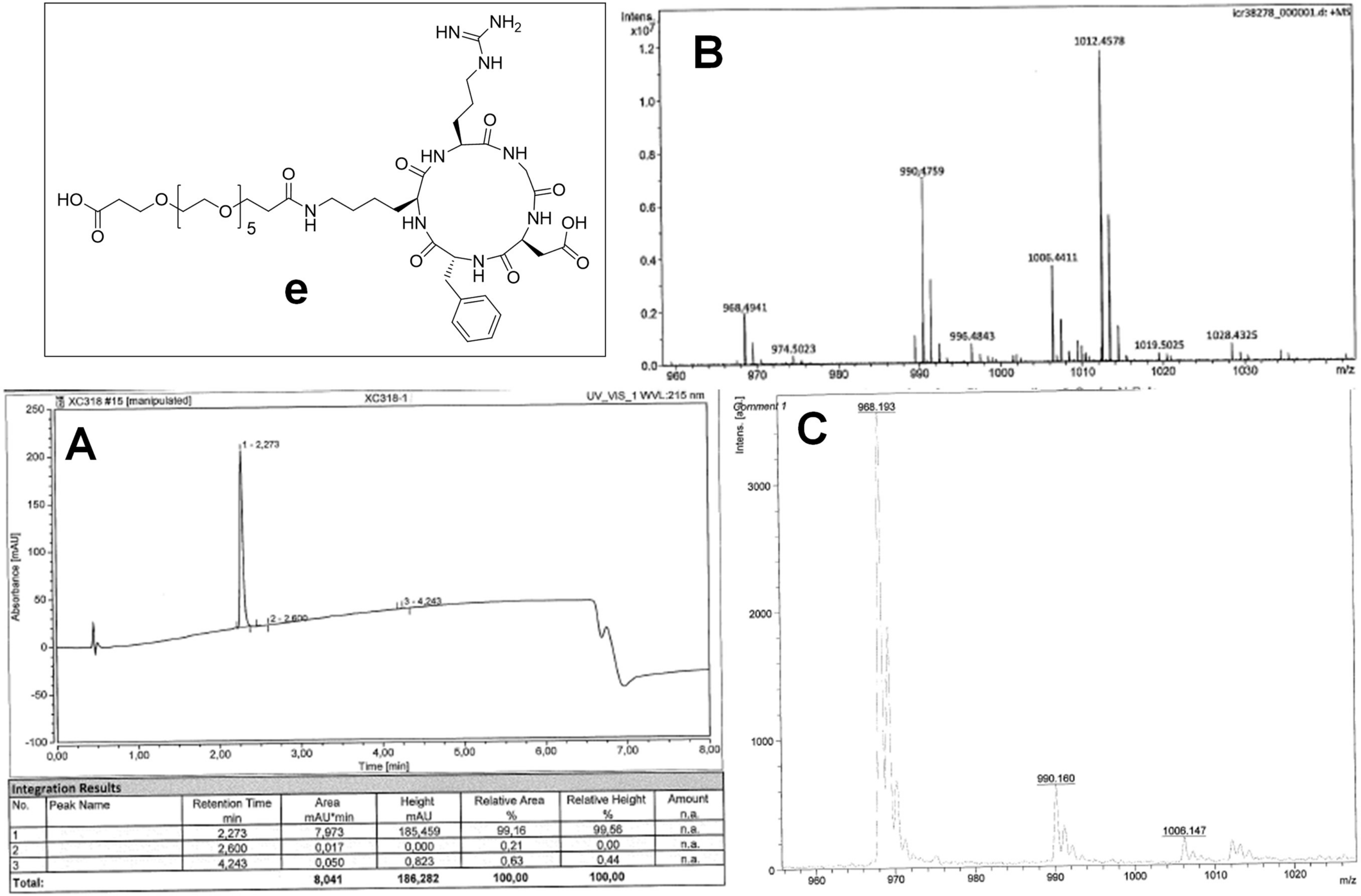

Integration Results

No.	Peak Name	Retention Time min	Area mAU*min	Height mAU	Relative Area %	Relative Height %	Amount n.a.
1		2,273	7,973	185,459	99,16	99,56	n.a.
2		2,600	0,017	0,000	0,21	0,00	n.a.
3		4,243	0,050	0,823	0,63	0,44	n.a.
Total:			8,041	186,282	100,00	100,00	

Figure S69. HPLC chromatogram (**A**) and mass spectra (**B**: ESI, **C**: MALDI) of HO-PEG$_5$-c(RGDfK) (**e**).

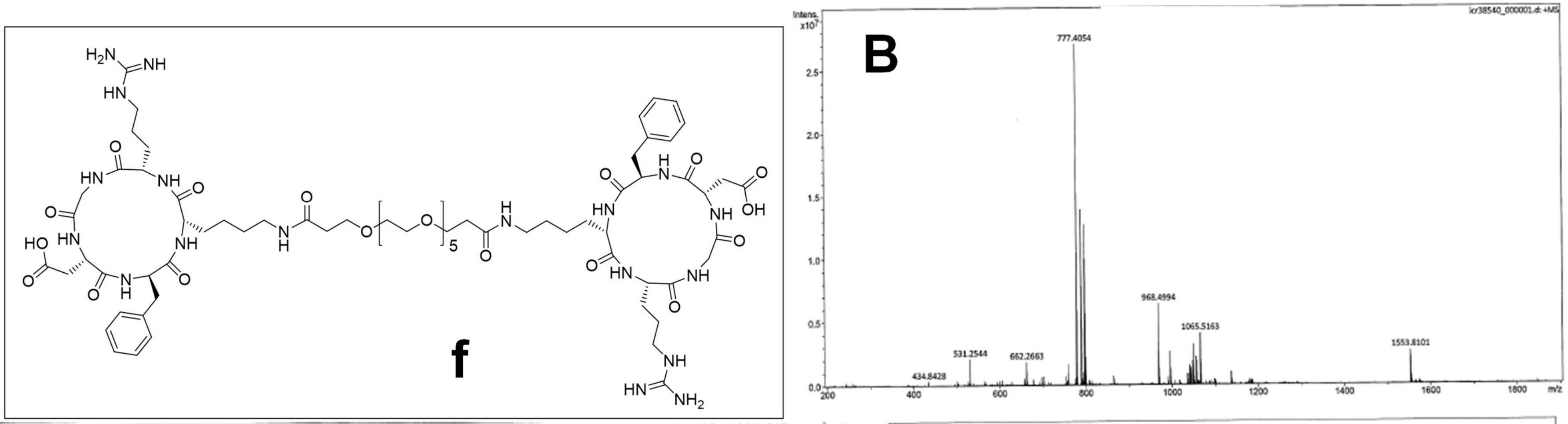

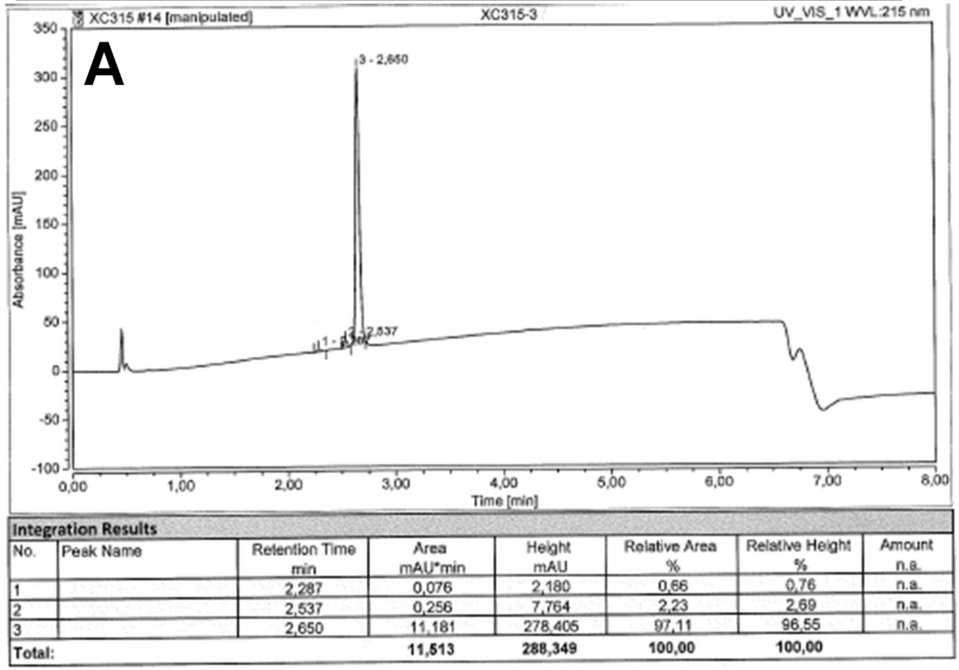

Integration Results							
No.	Peak Name	Retention Time min	Area mAU*min	Height mAU	Relative Area %	Relative Height %	Amount n.a.
1		2,287	0,076	2,180	0,66	0,76	n.a.
2		2,537	0,256	7,764	2,23	2,69	n.a.
3		2,650	11,181	278,405	97,11	96,55	n.a.
Total:			11,513	288,349	100,00	100,00	

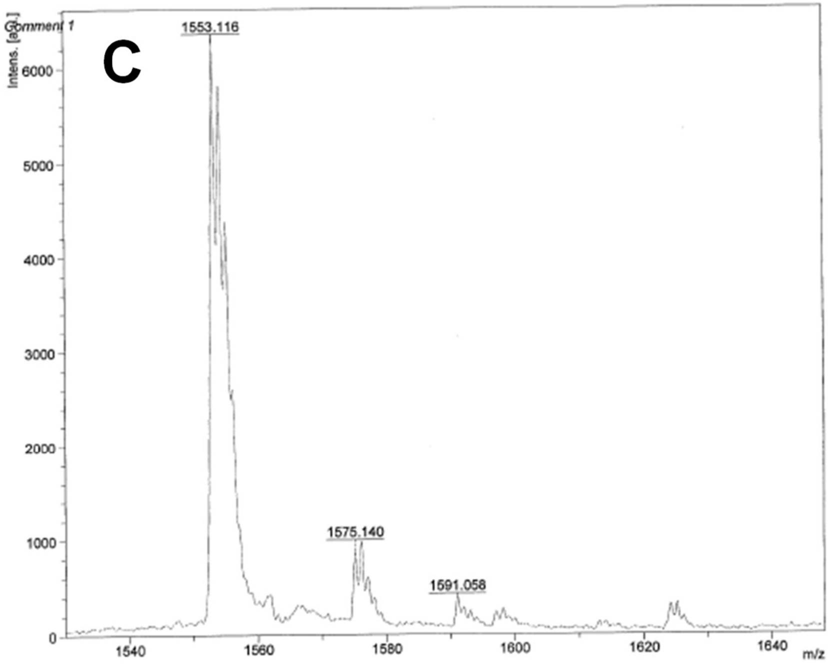

Figure S70. HPLC chromatogram (**A**) and mass spectra (**B**: ESI, **C**: MALDI) of c(RGDfK)-PEG$_5$-c(RGDfK) (**f**).

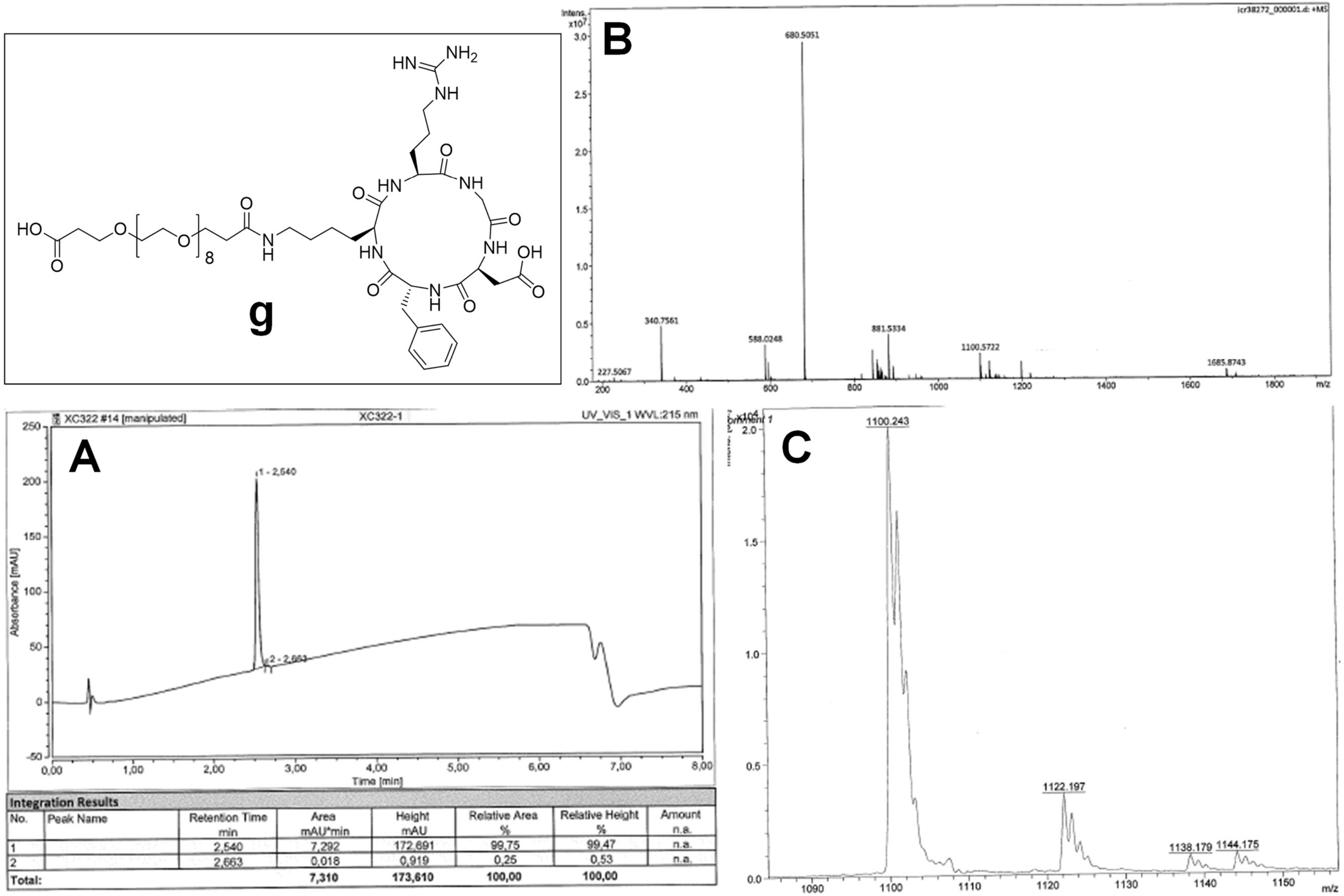

Integration Results

No.	Peak Name	Retention Time min	Area mAU*min	Height mAU	Relative Area %	Relative Height %	Amount n.a.
1		2,540	7,292	172,691	99,75	99,47	n.a.
2		2,663	0,018	0,919	0,25	0,53	n.a.
**Total:**			**7,310**	**173,610**	**100,00**	**100,00**	

Figure S71. HPLC chromatogram (**A**) and mass spectra (**B**: ESI, **C**: MALDI) of HO-PEG$_8$-c(RGDfK) (**g**).

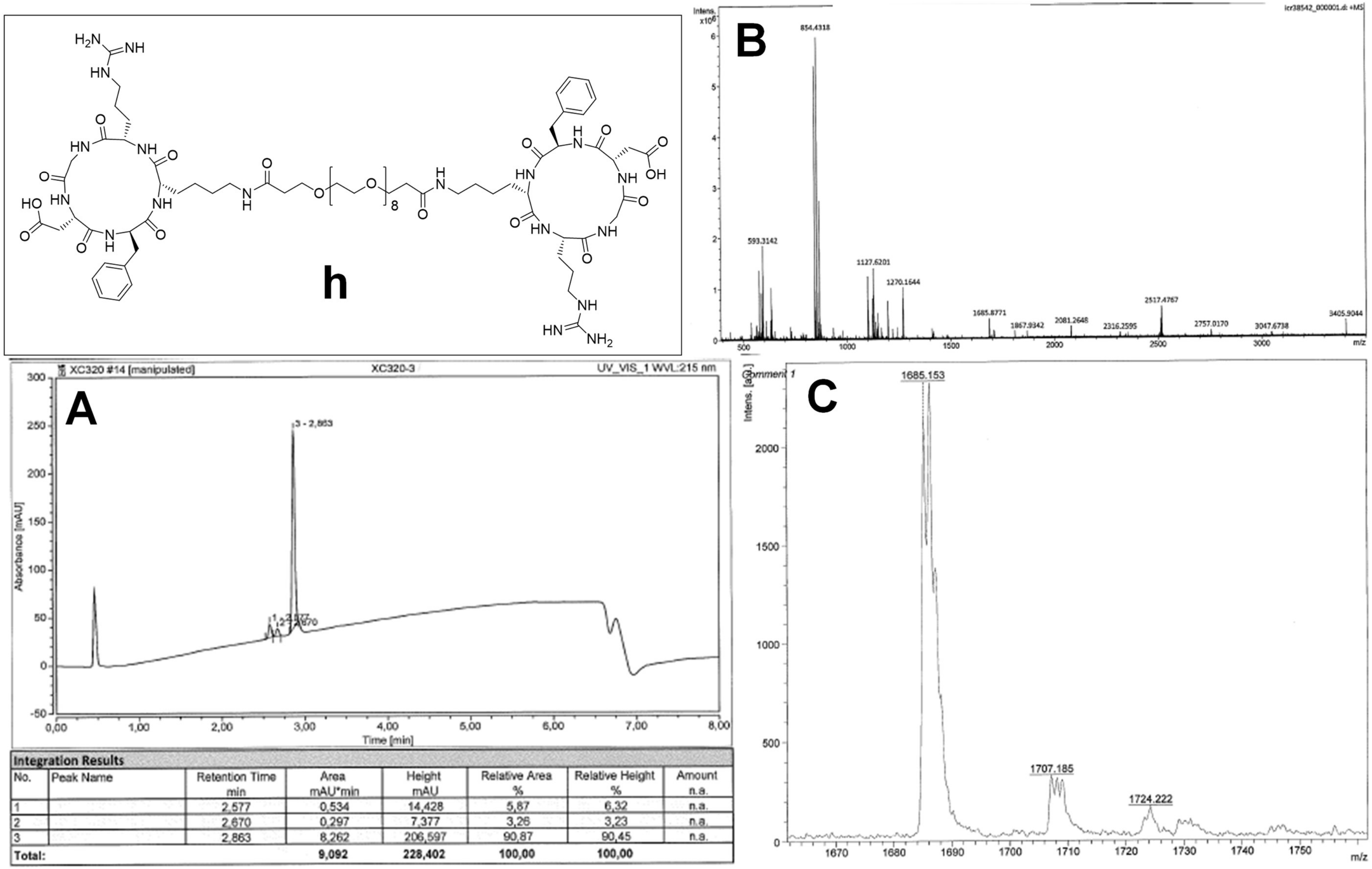

Integration Results

No.	Peak Name	Retention Time min	Area mAU*min	Height mAU	Relative Area %	Relative Height %	Amount n.a.
1		2,577	0,534	14,428	5,87	6,32	n.a.
2		2,670	0,297	7,377	3,26	3,23	n.a.
3		2,863	8,262	206,597	90,87	90,45	n.a.
**Total:**			**9,092**	**228,402**	**100,00**	**100,00**	

Figure S72. HPLC chromatogram (**A**) and mass spectra (**B**: ESI, **C**: MALDI) of c(RGDfK)-PEG$_8$-c(RGDfK) (**h**).

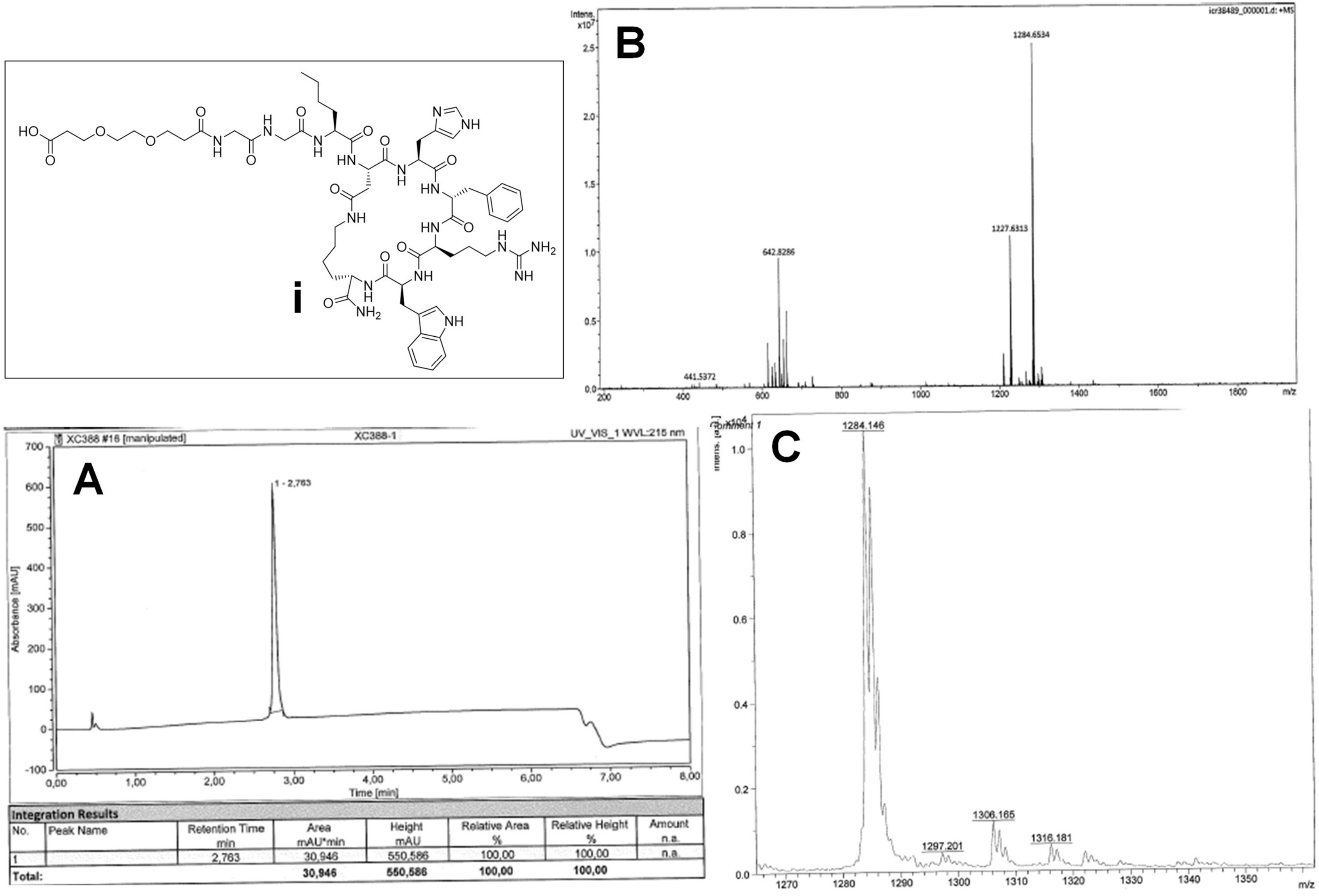

Integration Results

No.	Peak Name	Retention Time min	Area mAU*min	Height mAU	Relative Area %	Relative Height %	Amount n.a.
1		2,763	30,946	550,586	100,00	100,00	n.a.
**Total:**			**30,946**	**550,586**	**100,00**	**100,00**	

Figure S73. HPLC chromatogram (**A**) and mass spectra (**B**: ESI, **C**: MALDI) of HO-PEG$_1$-GG-Nle-c(DHfRWK) (**i**).

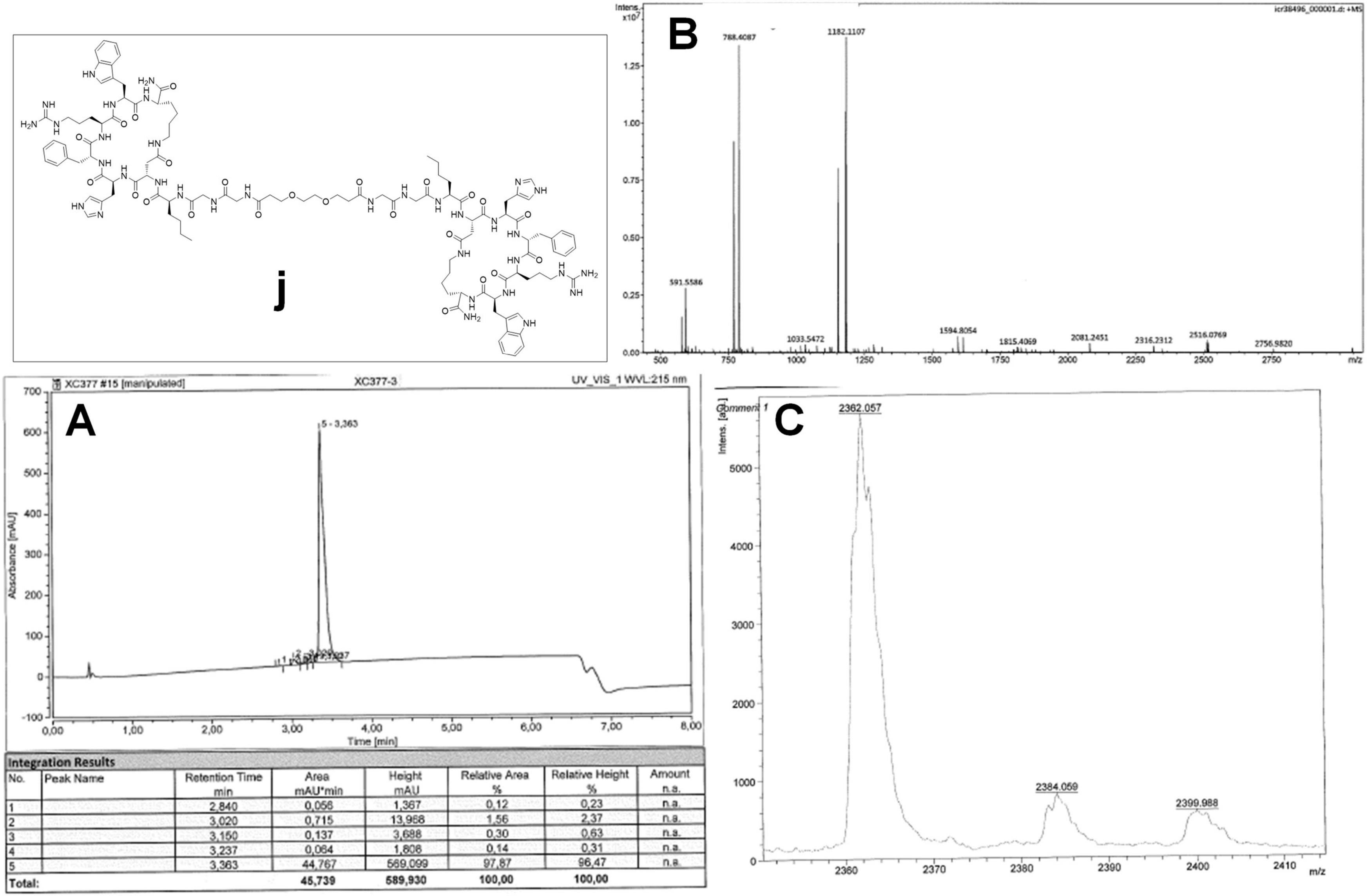

Integration Results

No.	Peak Name	Retention Time min	Area mAU*min	Height mAU	Relative Area %	Relative Height %	Amount n.a.
1		2,840	0,056	1,367	0,12	0,23	n.a.
2		3,020	0,715	13,968	1,56	2,37	n.a.
3		3,150	0,137	3,688	0,30	0,63	n.a.
4		3,237	0,064	1,806	0,14	0,31	n.a.
5		3,363	44,767	569,099	97,87	96,47	n.a.
Total:			45,739	589,930	100,00	100,00	

Figure S74. HPLC chromatogram (**A**) and mass spectra (**B**: ESI, **C**: MALDI) of c(DHfRWK)-Nle-GG-PEG$_1$-GG-Nle-c(DHfRWK) (**j**).

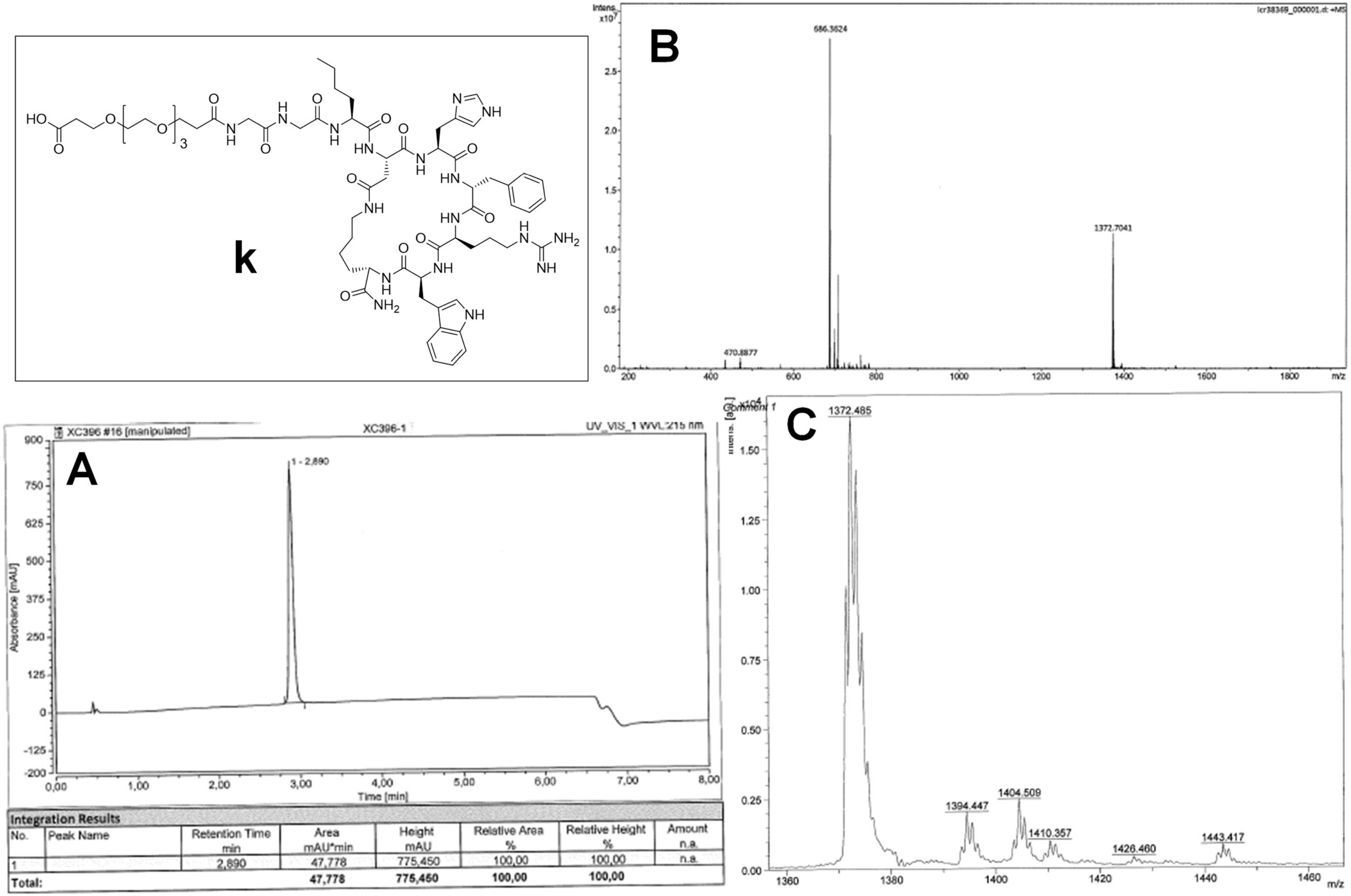

Integration Results

No.	Peak Name	Retention Time min	Area mAU*min	Height mAU	Relative Area %	Relative Height %	Amount n.a.
1		2,890	47,778	775,450	100,00	100,00	n.a.
**Total:**			**47,778**	**775,450**	**100,00**	**100,00**	

Figure S75. HPLC chromatogram (**A**) and mass spectra (**B**: ESI, **C**: MALDI) of HO-PEG$_3$-GG-Nle-c(DHfRWK) (**k**).

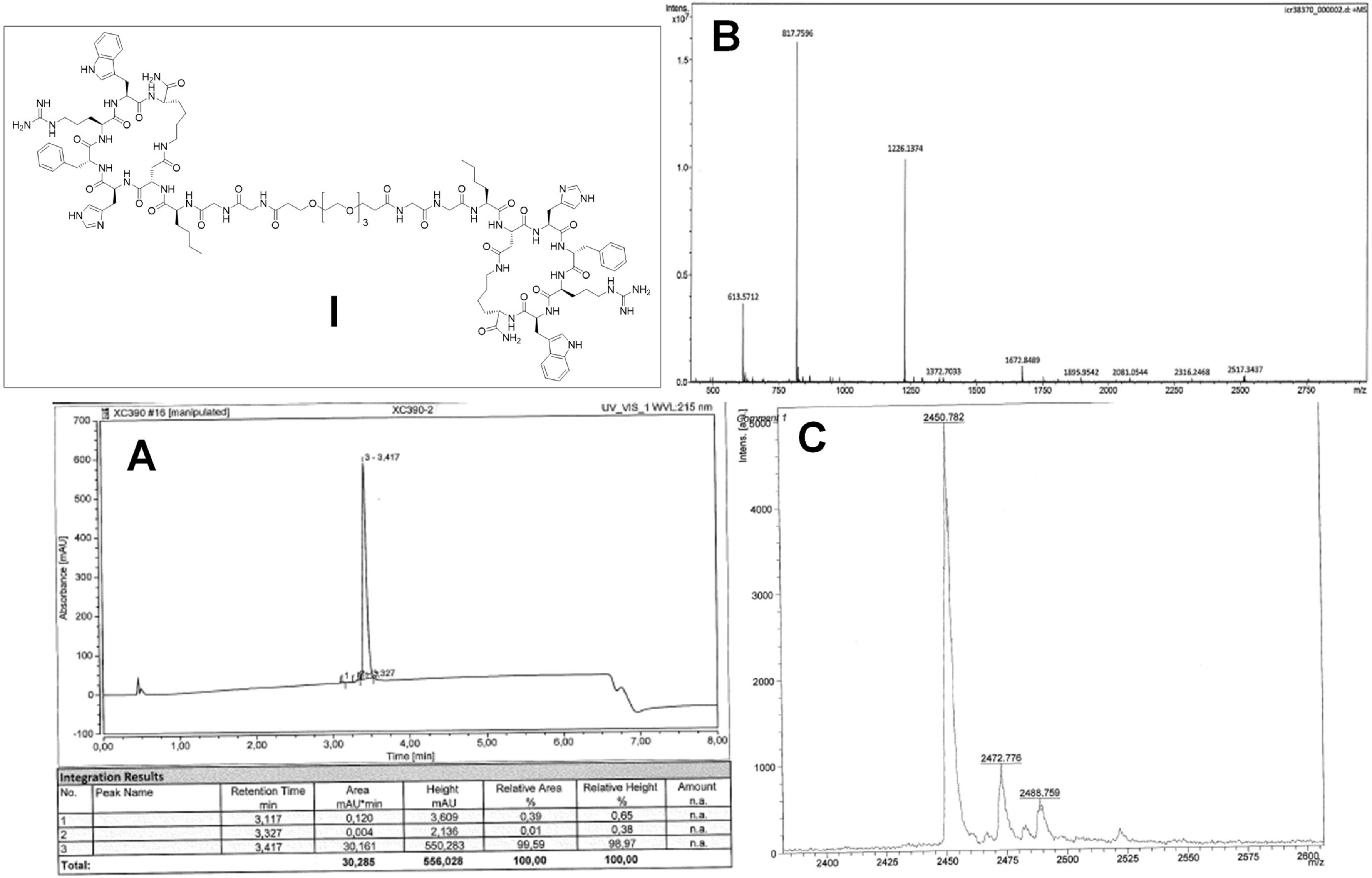

Integration Results

No.	Peak Name	Retention Time min	Area mAU*min	Height mAU	Relative Area %	Relative Height %	Amount n.a.
1		3,117	0,120	3,609	0,39	0,65	n.a.
2		3,327	0,004	2,136	0,01	0,38	n.a.
3		3,417	30,161	550,283	99,59	98,97	n.a.
**Total:**			**30,285**	**556,028**	**100,00**	**100,00**	

Figure S76. HPLC chromatogram (**A**) and mass spectra (**B**: ESI, **C**: MALDI) of c(DHfRWK)-Nle-GG-PEG_3-GG-Nle-c(DHfRWK) (**I**).

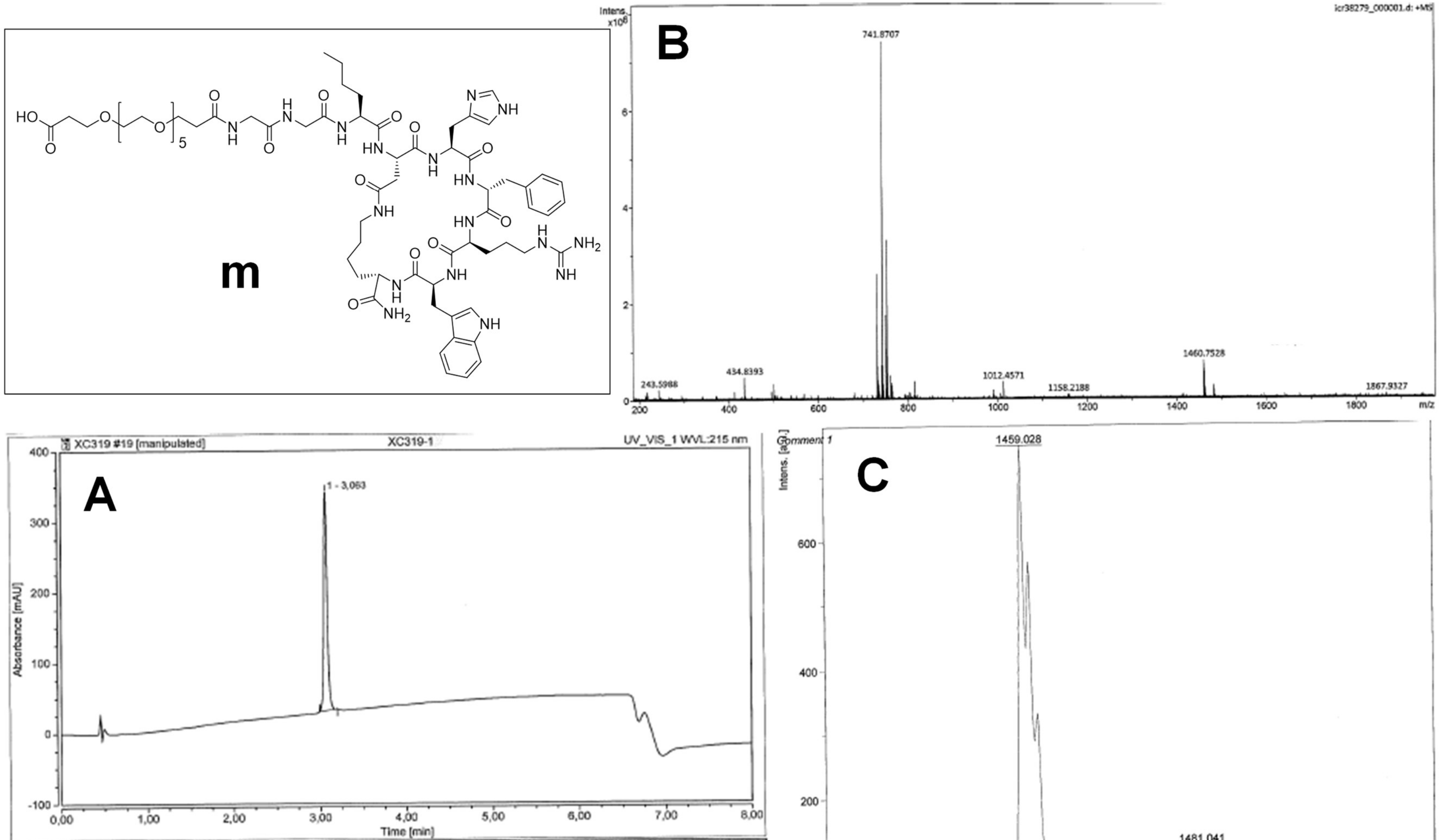

Integration Results

No.	Peak Name	Retention Time min	Area mAU*min	Height mAU	Relative Area %	Relative Height %	Amount
n.a.	Component 1	n.a.	n.a.	n.a.	n.a.	n.a.	n.a.
1		3,063	13,904	309,479	100,00	100,00	n.a.
**Total:**			**13,904**	**309,479**	**100,00**	**100,00**	

Figure S77. HPLC chromatogram (**A**) and mass spectra (**B**: ESI, **C**: MALDI) of HO-PEG$_5$-GG-Nle-c(DHfRWK) (**m**).

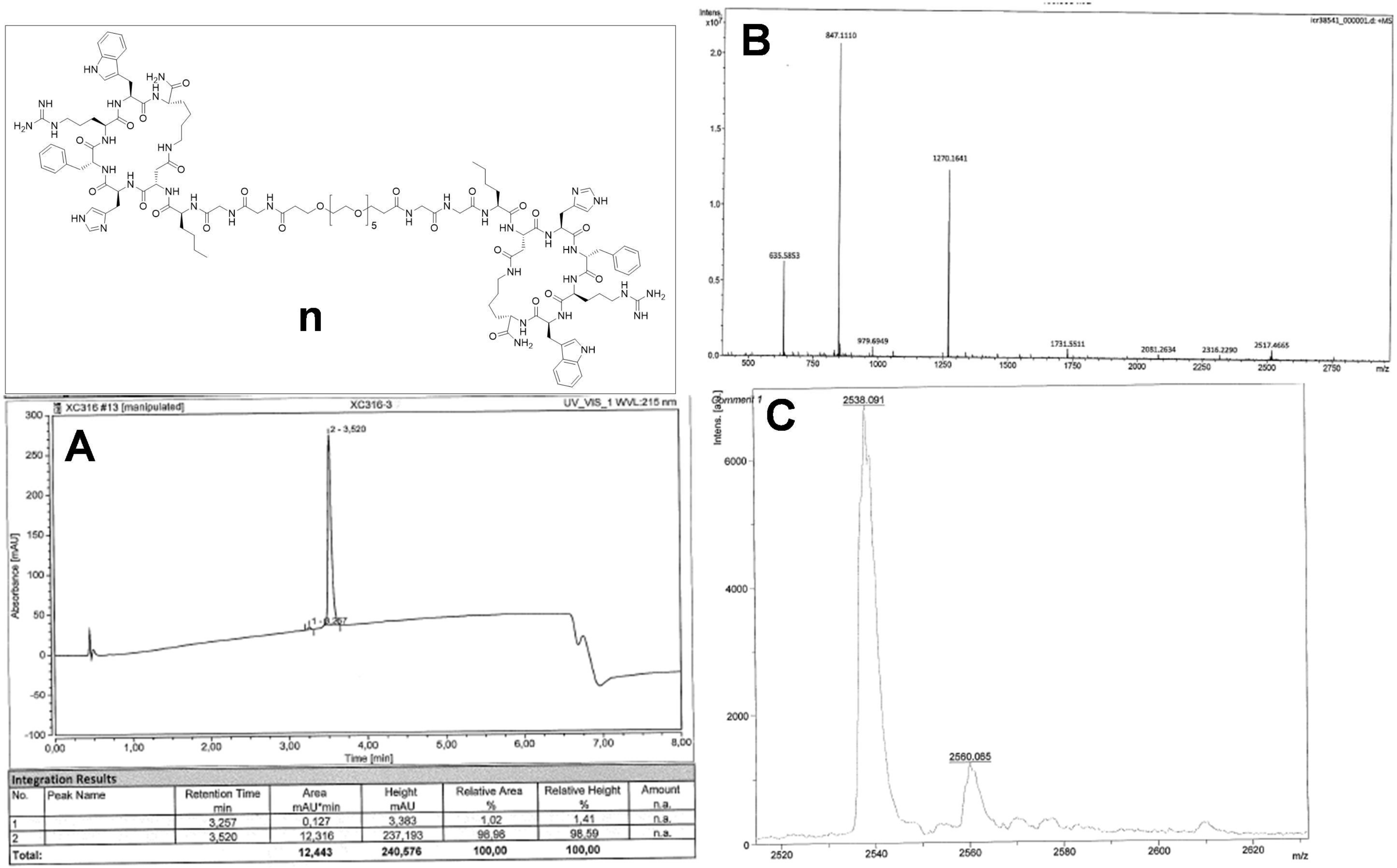

Integration Results

No.	Peak Name	Retention Time min	Area mAU*min	Height mAU	Relative Area %	Relative Height %	Amount n.a.
1		3,257	0,127	3,383	1,02	1,41	n.a.
2		3,520	12,316	237,193	98,98	98,59	n.a.
**Total:**			**12,443**	**240,576**	**100,00**	**100,00**	

Figure S78. HPLC chromatogram (**A**) and mass spectra (**B**: ESI, **C**: MALDI) of c(DHfRWK)-Nle-GG-PEG$_5$-GG-Nle-c(DHfRWK) (**n**).

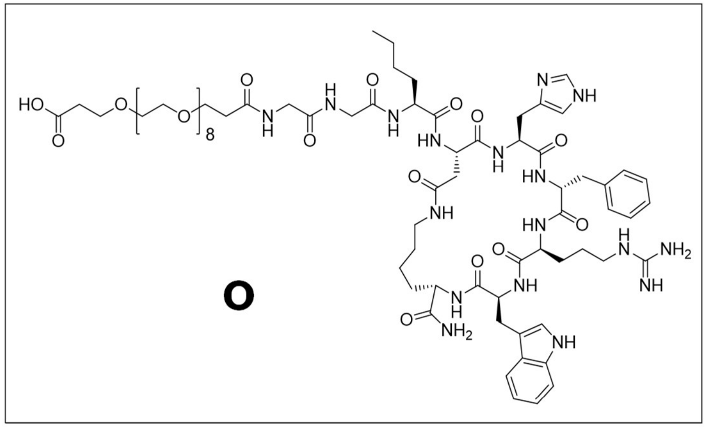

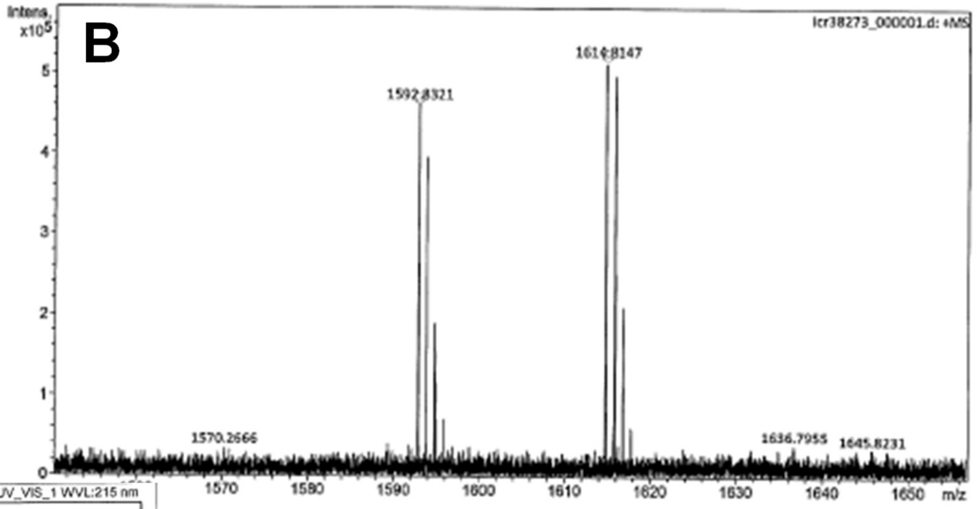

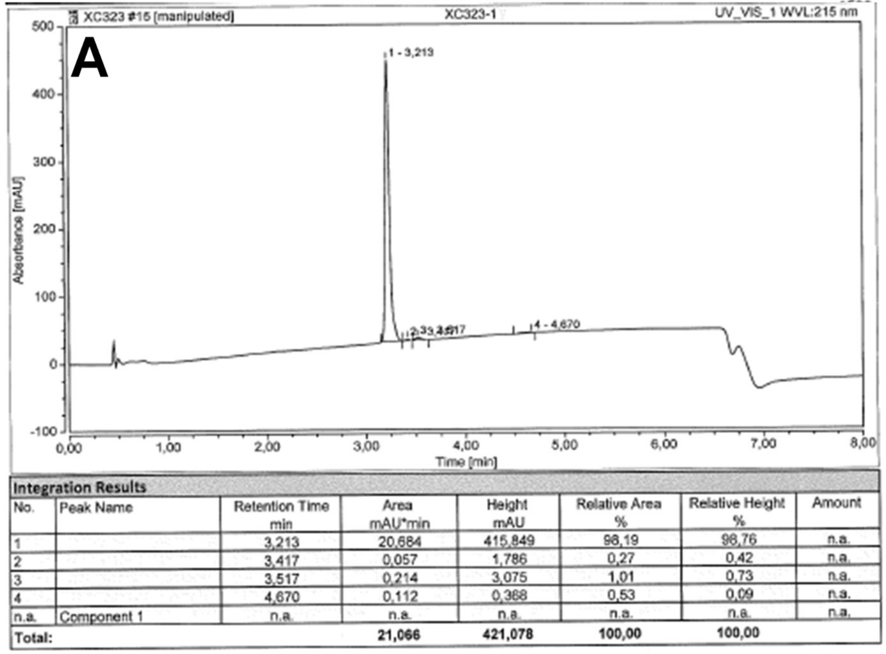

Integration Results							
No.	Peak Name	Retention Time min	Area mAU*min	Height mAU	Relative Area %	Relative Height %	Amount
1		3,213	20,684	415,849	98,19	98,76	n.a.
2		3,417	0,057	1,786	0,27	0,42	n.a.
3		3,517	0,214	3,075	1,01	0,73	n.a.
4		4,670	0,112	0,368	0,53	0,09	n.a.
n.a.	Component 1	n.a.	n.a.	n.a.	n.a.	n.a.	n.a.
Total:			21,066	421,078	100,00	100,00	

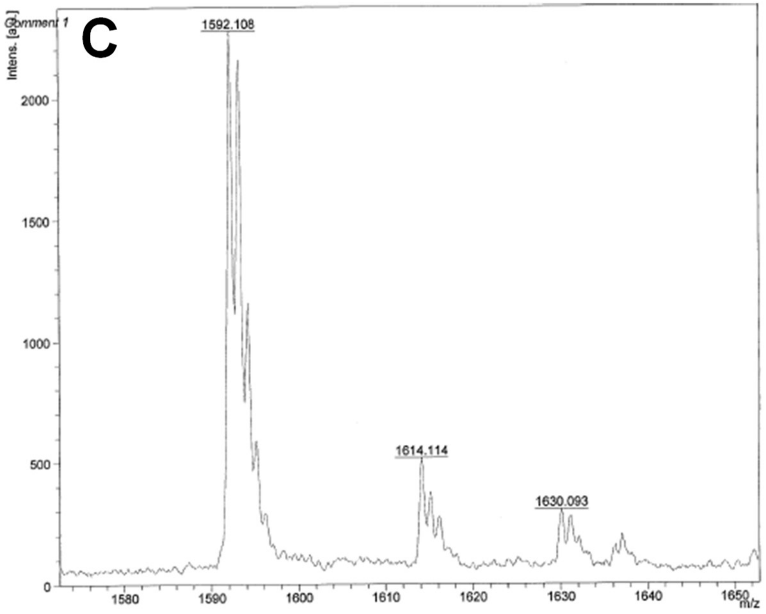

Figure S79. HPLC chromatogram (**A**) and mass spectra (**B**: ESI, **C**: MALDI) of HO-PEG$_8$-GG-Nle-c(DHfRWK) (**o**).

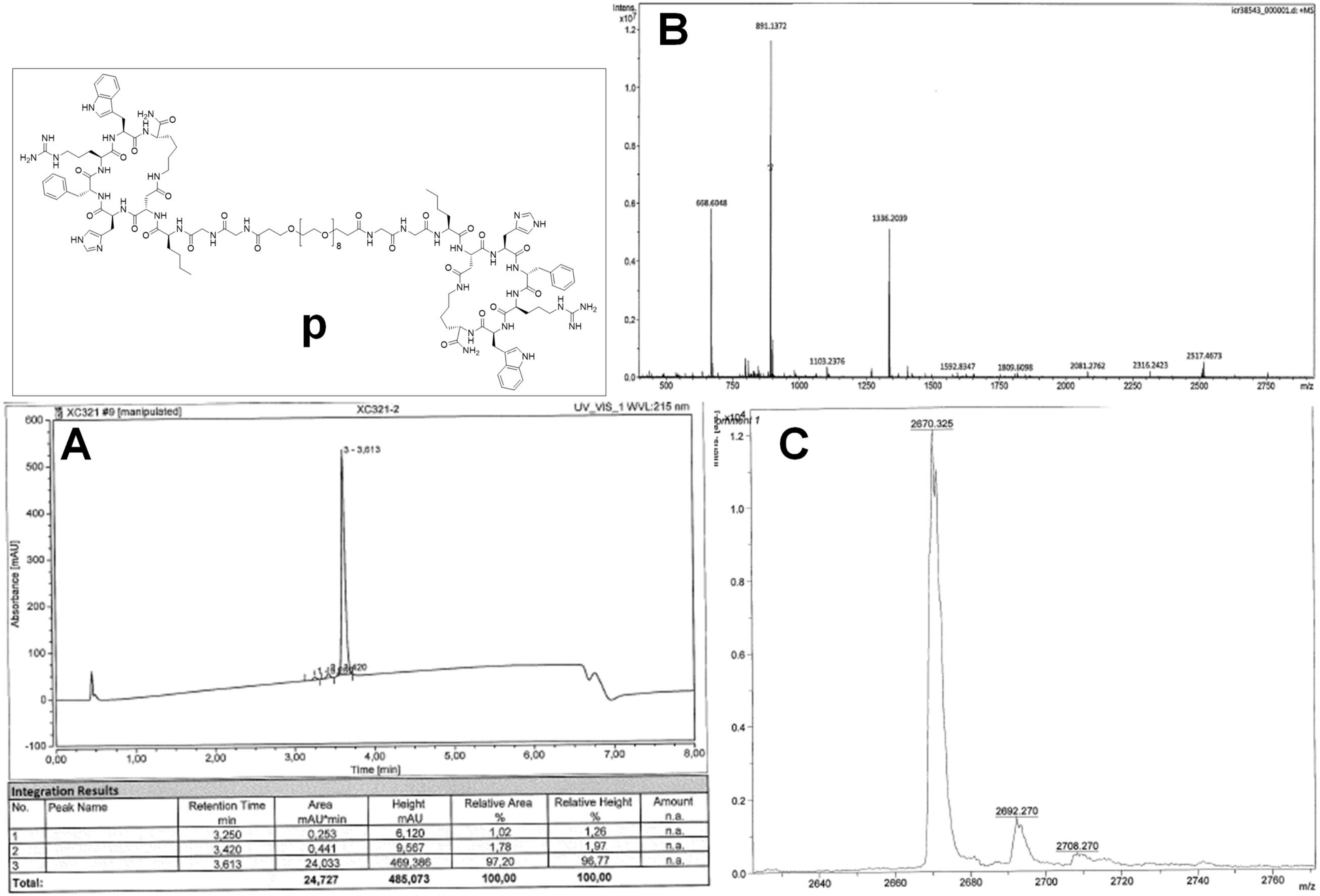

Integration Results

No.	Peak Name	Retention Time min	Area mAU*min	Height mAU	Relative Area %	Relative Height %	Amount n.a.
1		3,250	0,253	6,120	1,02	1,26	n.a.
2		3,420	0,441	9,567	1,78	1,97	n.a.
3		3,613	24,033	469,386	97,20	96,77	n.a.
**Total:**			**24,727**	**485,073**	**100,00**	**100,00**	

Figure S80. HPLC chromatogram (**A**) and mass spectra (**B**: ESI, **C**: MALDI) of c(DHfRWK)-Nle-GG-PEG$_8$-GG-Nle-c(DHfRWK) (**p**).

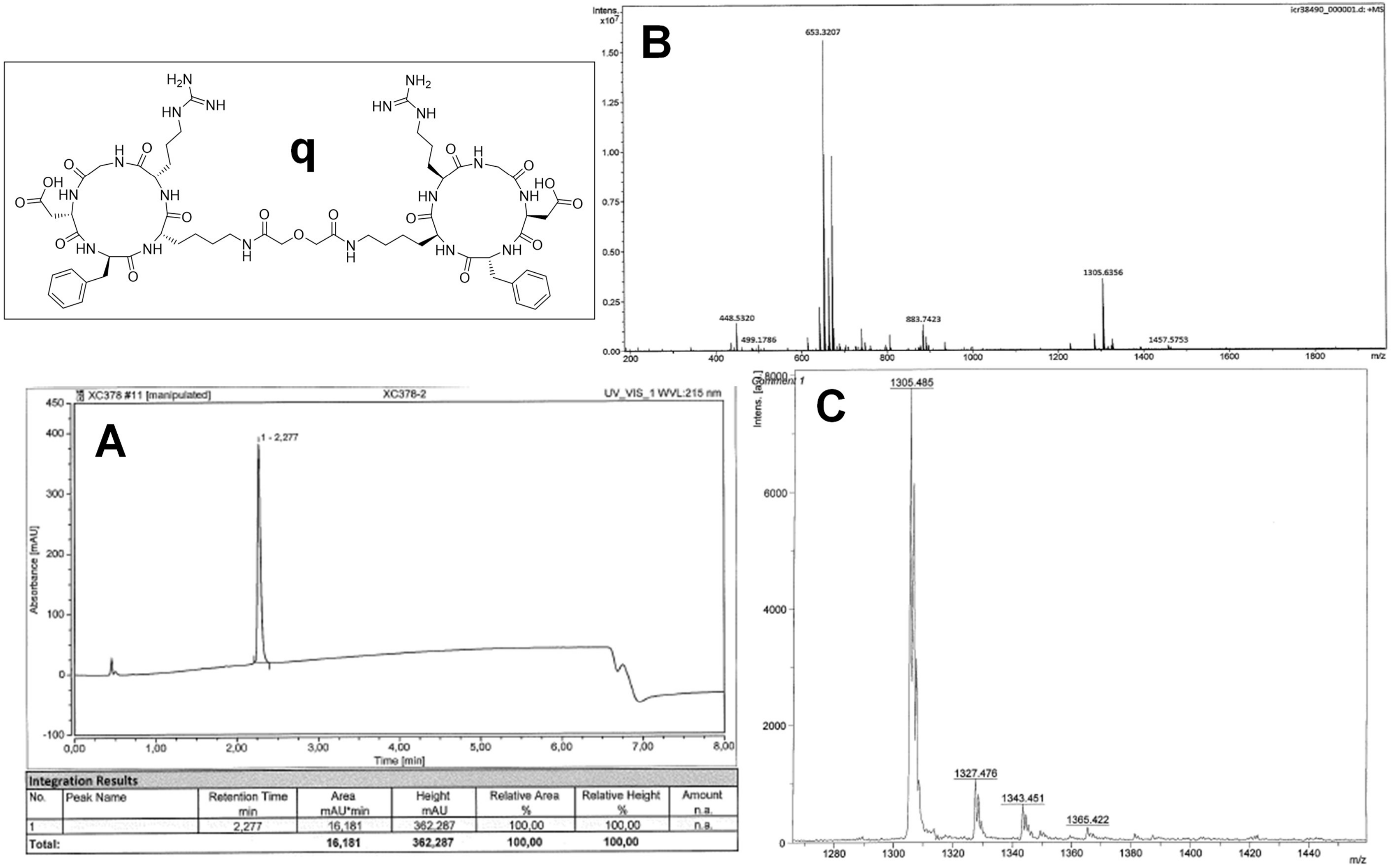

Integration Results							
No.	Peak Name	Retention Time min	Area mAU*min	Height mAU	Relative Area %	Relative Height %	Amount n.a.
1		2,277	16,181	362,287	100,00	100,00	n.a.
**Total:**			**16,181**	**362,287**	**100,00**	**100,00**	

Figure S81. HPLC chromatogram (**A**) and mass spectra (**B**: ESI, **C**: MALDI) of c(RGDfK)-DIG-c(RGDfK) (**q**).

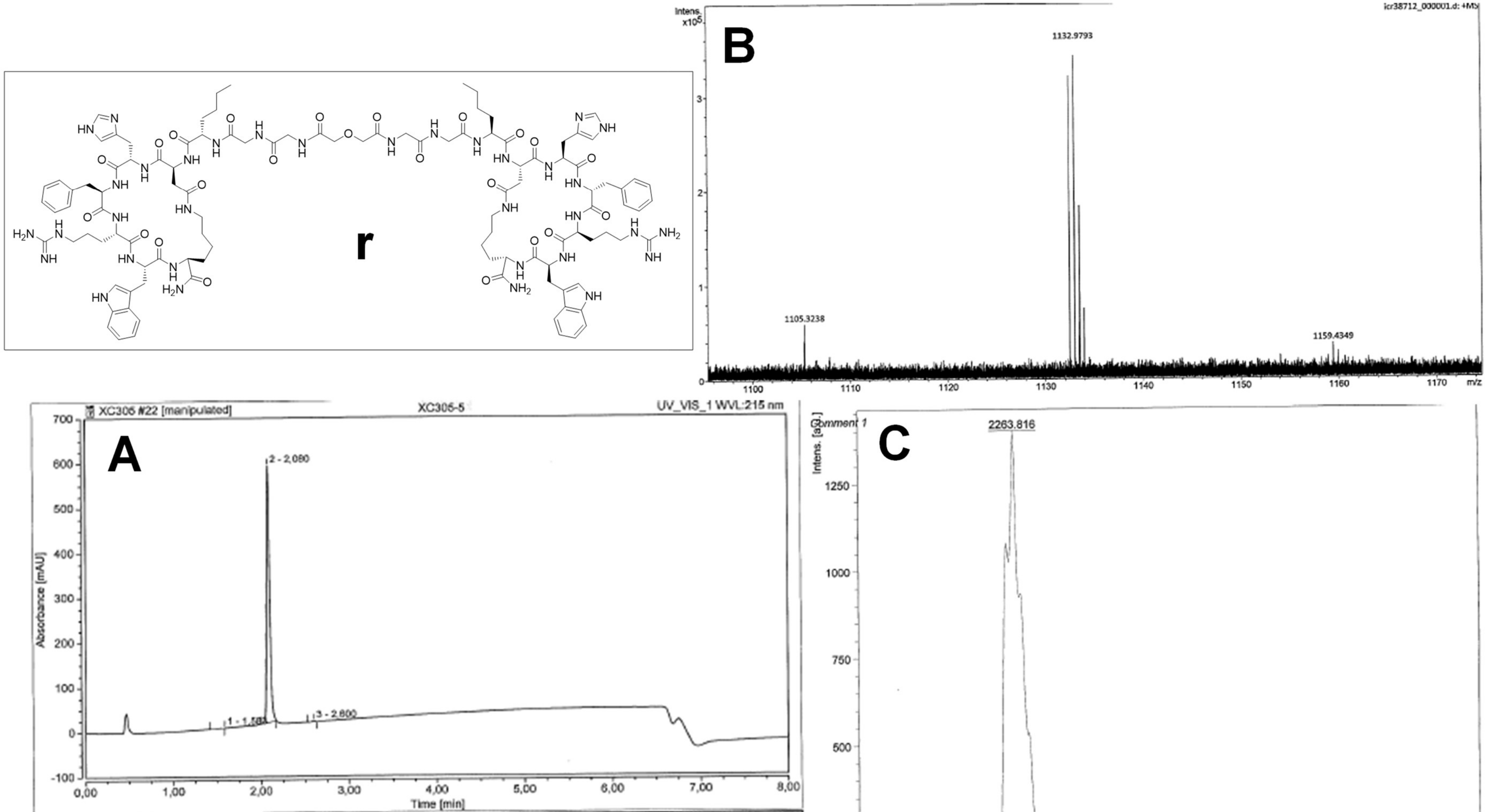

Integration Results

No.	Peak Name	Retention Time min	Area mAU*min	Height mAU	Relative Area %	Relative Height %	Amount n.a.
1		1,583	0,013	0,000	0,06	0,00	n.a.
2		2,080	22,956	571,859	99,79	99,83	n.a.
3		2,600	0,034	0,956	0,15	0,17	n.a.
**Total:**			**23,003**	**572,814**	**100,00**	**100,00**	

Figure S82. HPLC chromatogram (**A**) and mass spectra (**B**: ESI, **C**: MALDI) of c(RGDfK)-EGEGE-Ox-EGEGE-c(RGDfK) (**r**).

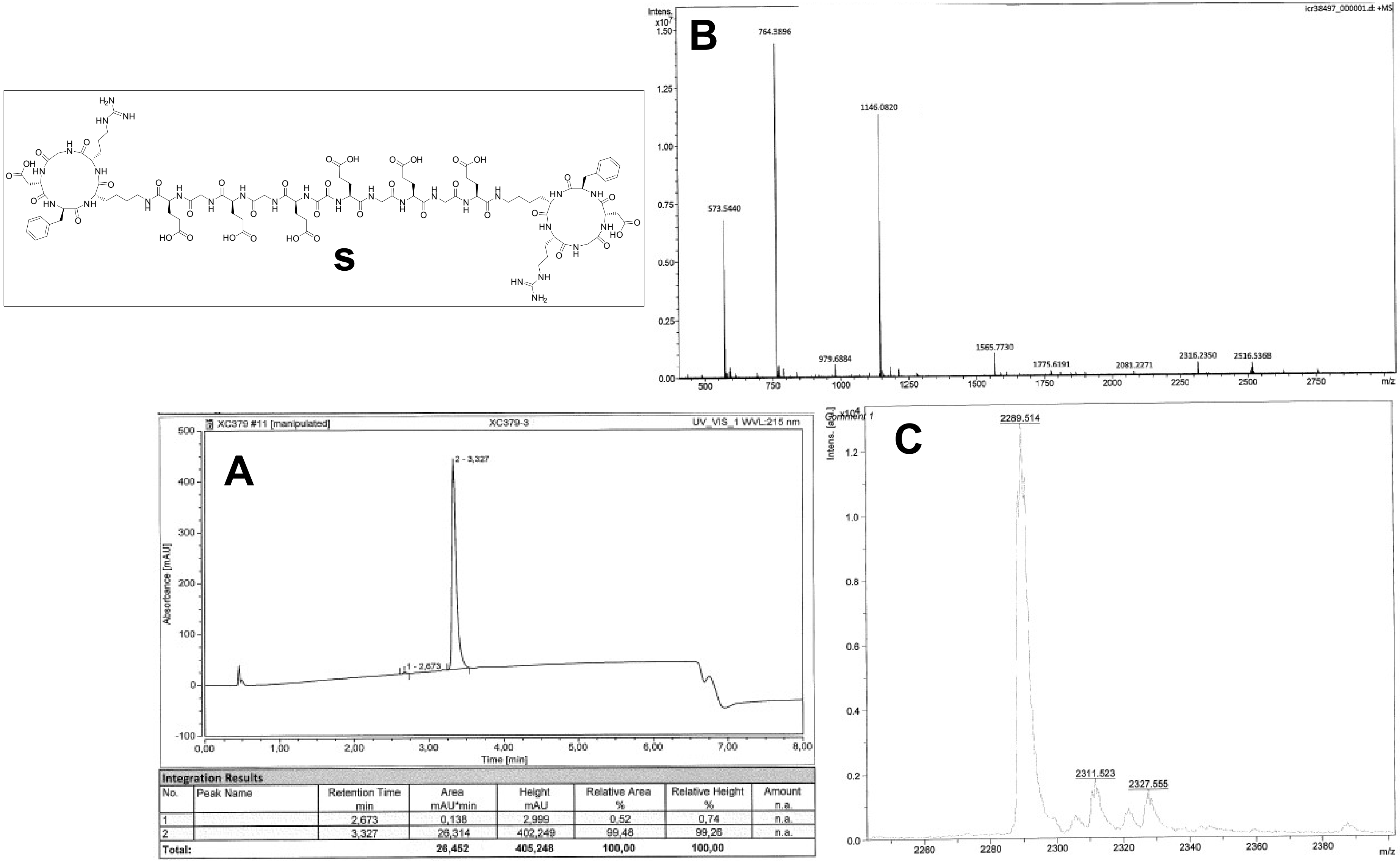

No.	Peak Name	Retention Time min	Area mAU*min	Height mAU	Relative Area %	Relative Height %	Amount n.a.
1		2,673	0,138	2,999	0,52	0,74	n.a.
2		3,327	26,314	402,249	99,48	99,26	n.a.
**Total:**			**26,452**	**405,248**	**100,00**	**100,00**	

Figure S83. HPLC chromatogram (**A**) and mass spectra (**B**: ESI, **C**: MALDI) of c(DHfRWK)-Nle-GG-DIG-GG-Nle-c(DHfRWK) (**s**).

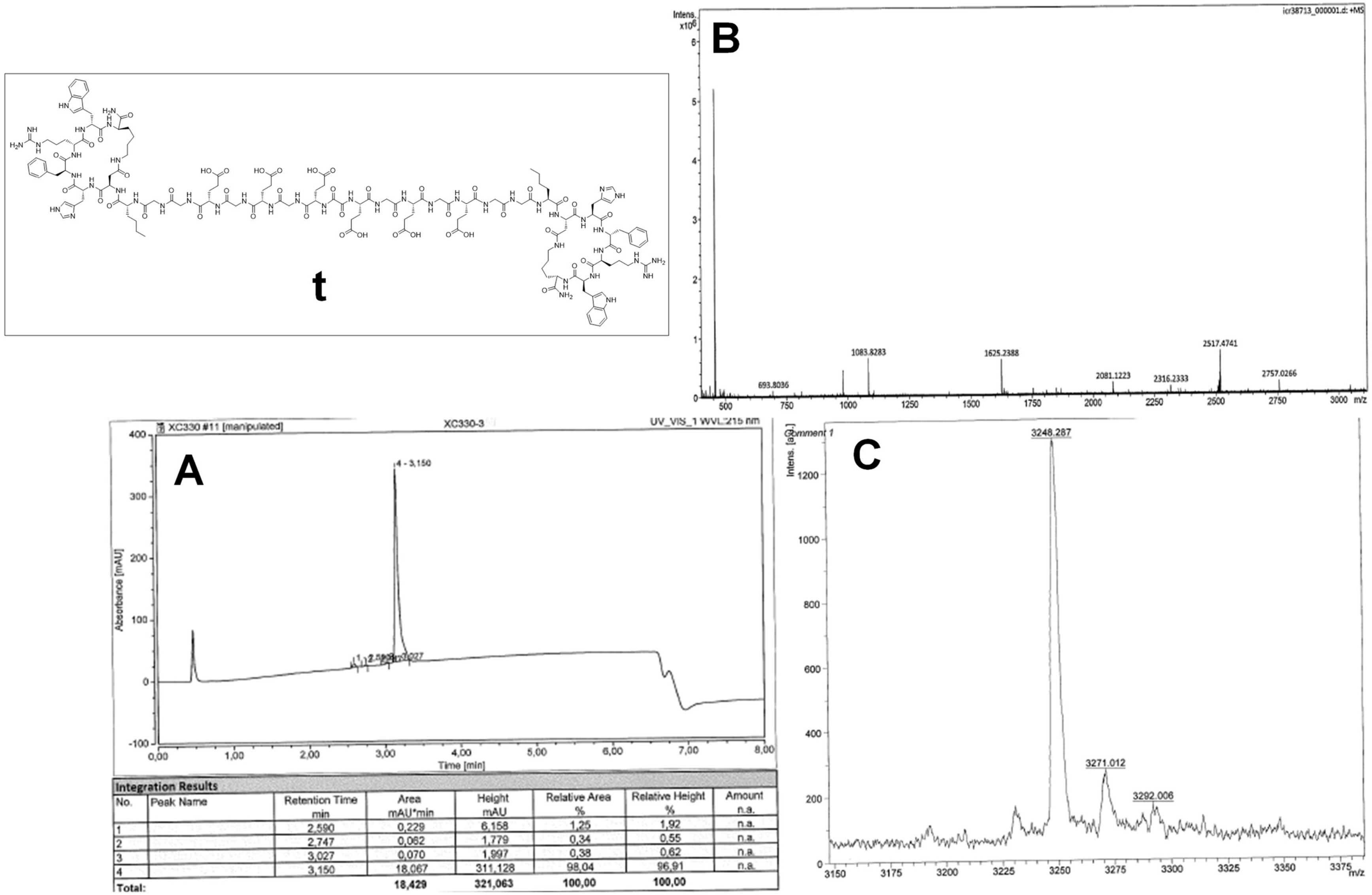

Integration Results							
No.	Peak Name	Retention Time min	Area mAU*min	Height mAU	Relative Area %	Relative Height %	Amount n.a.
1		2,590	0,229	6,158	1,25	1,92	n.a.
2		2,747	0,062	1,779	0,34	0,55	n.a.
3		3,027	0,070	1,997	0,38	0,62	n.a.
4		3,150	18,067	311,128	98,04	96,91	n.a.
Total:			18,429	321,063	100,00	100,00	

Figure S84. HPLC chromatogram (**A**) and mass spectra (**B**: ESI, **C**: MALDI) of c(DHfRWK)-Nle-GG-EGEGE-Ox-EGEGE-GG-Nle-c(DHfRWK) (**t**).

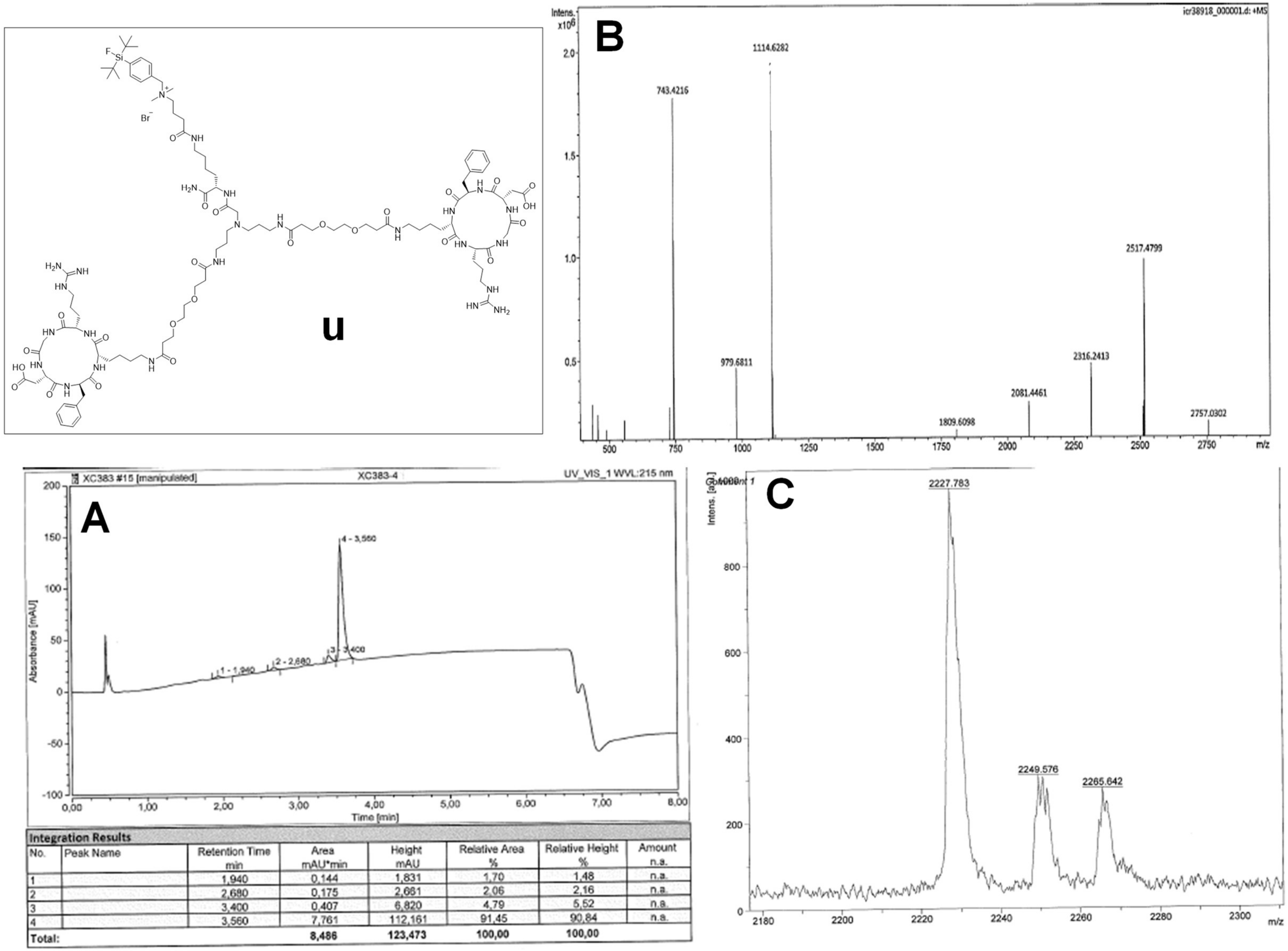

Figure S85. HPLC chromatogram (**A**) and mass spectra (**B**: ESI, **C**: MALDI) of SiFA*lin*-APG-[PEG$_1$-c(RGDfK)]$_2$ (**u**).

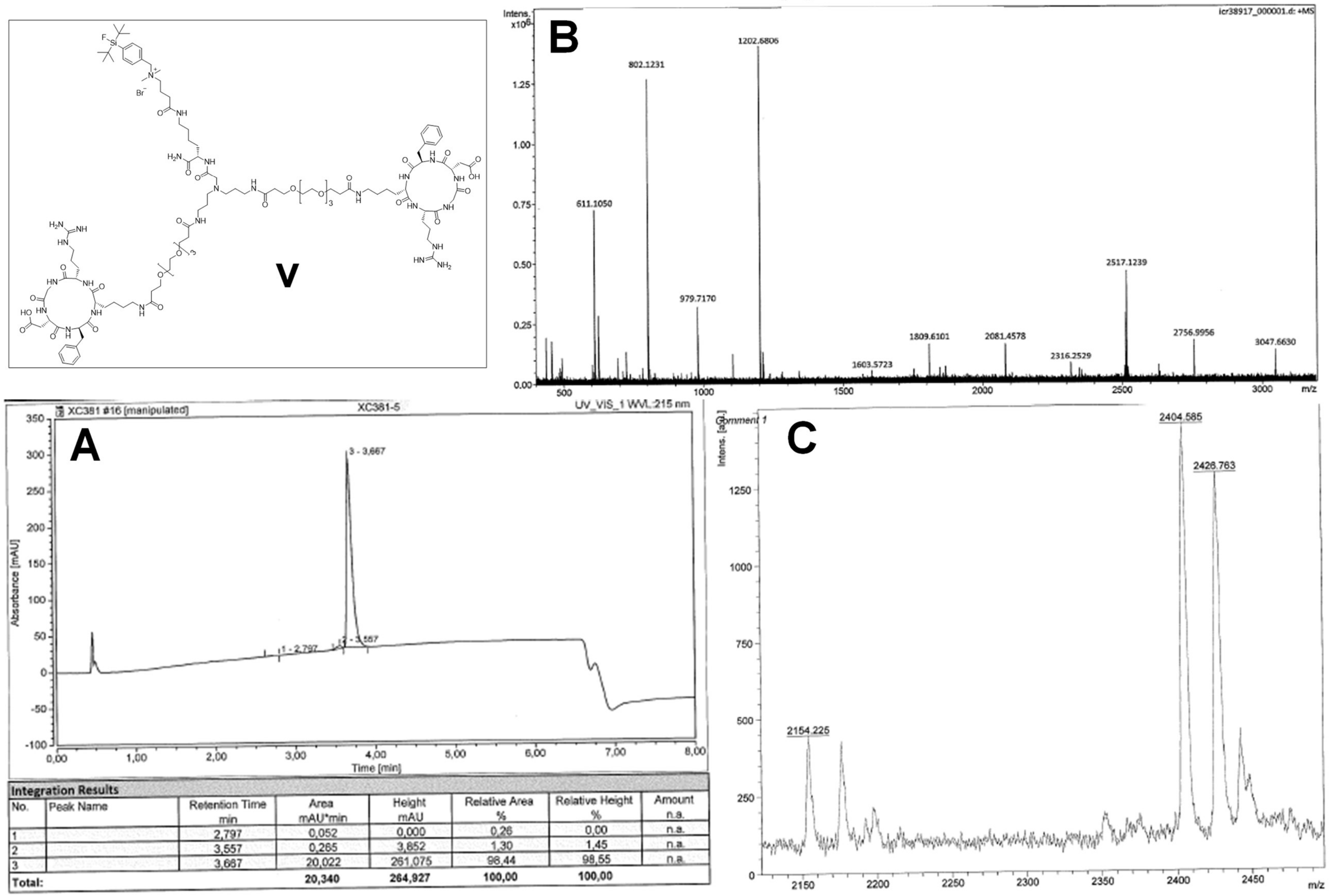

Integration Results

No.	Peak Name	Retention Time min	Area mAU*min	Height mAU	Relative Area %	Relative Height %	Amount n.a.
1		2,797	0,052	0,000	0,26	0,00	n.a.
2		3,557	0,265	3,852	1,30	1,45	n.a.
3		3,667	20,022	261,075	98,44	98,55	n.a.
Total:			20,340	264,927	100,00	100,00	

Figure S86. HPLC chromatogram (**A**) and mass spectra (**B**: ESI, **C**: MALDI) of SiFA*lin*-APG-[PEG$_3$-c(RGDfK)]$_2$ (**v**).

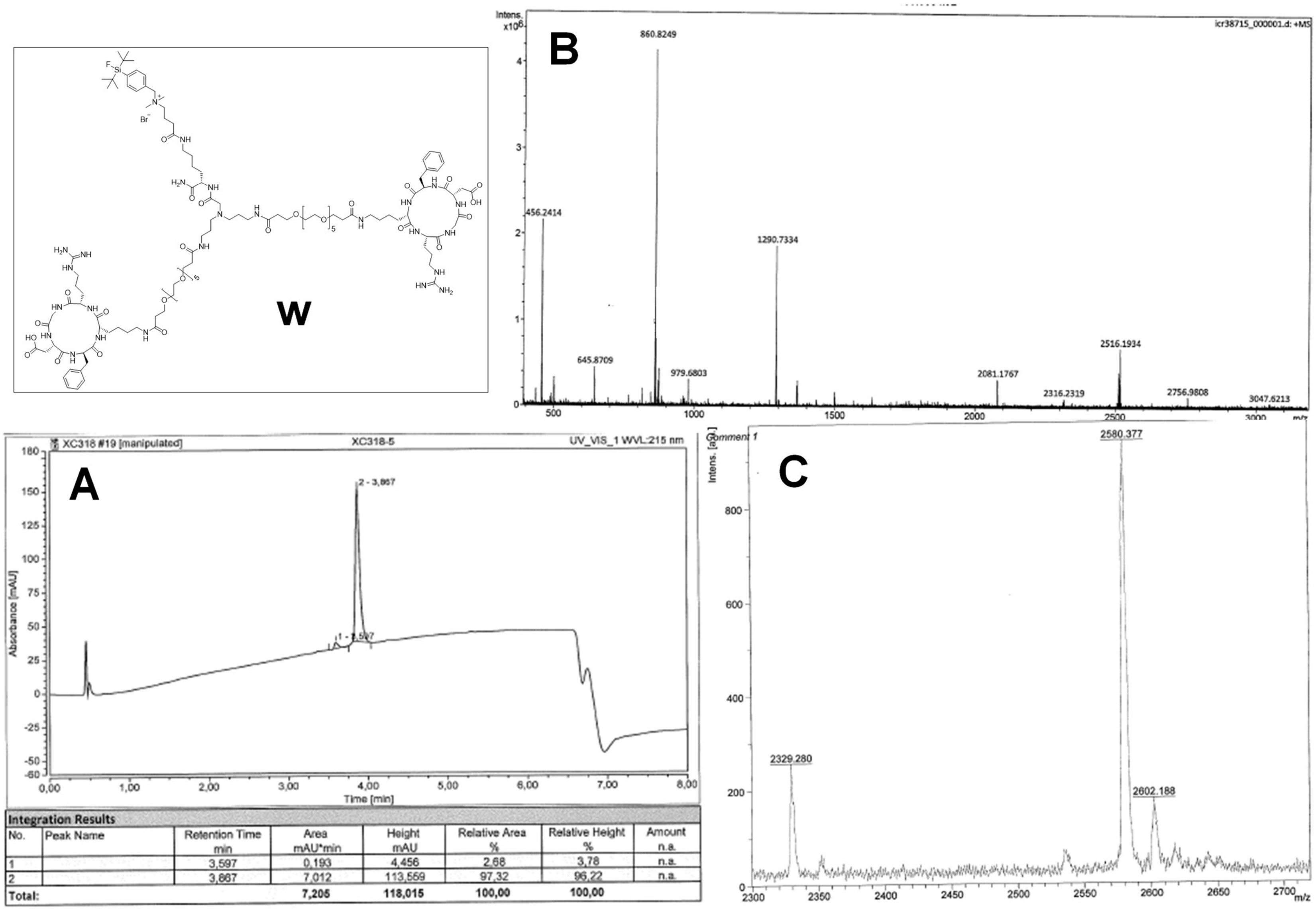

Integration Results							
No.	Peak Name	Retention Time min	Area mAU*min	Height mAU	Relative Area %	Relative Height %	Amount n.a.
1		3,597	0,193	4,456	2,68	3,78	n.a.
2		3,867	7,012	113,559	97,32	96,22	n.a.
Total:			7,205	118,015	100,00	100,00	

Figure S87. HPLC chromatogram (**A**) and mass spectra (**B**: ESI, **C**: MALDI) of SiFA*lin*-APG-[PEG$_5$-c(RGDfK)]$_2$ (**w**).

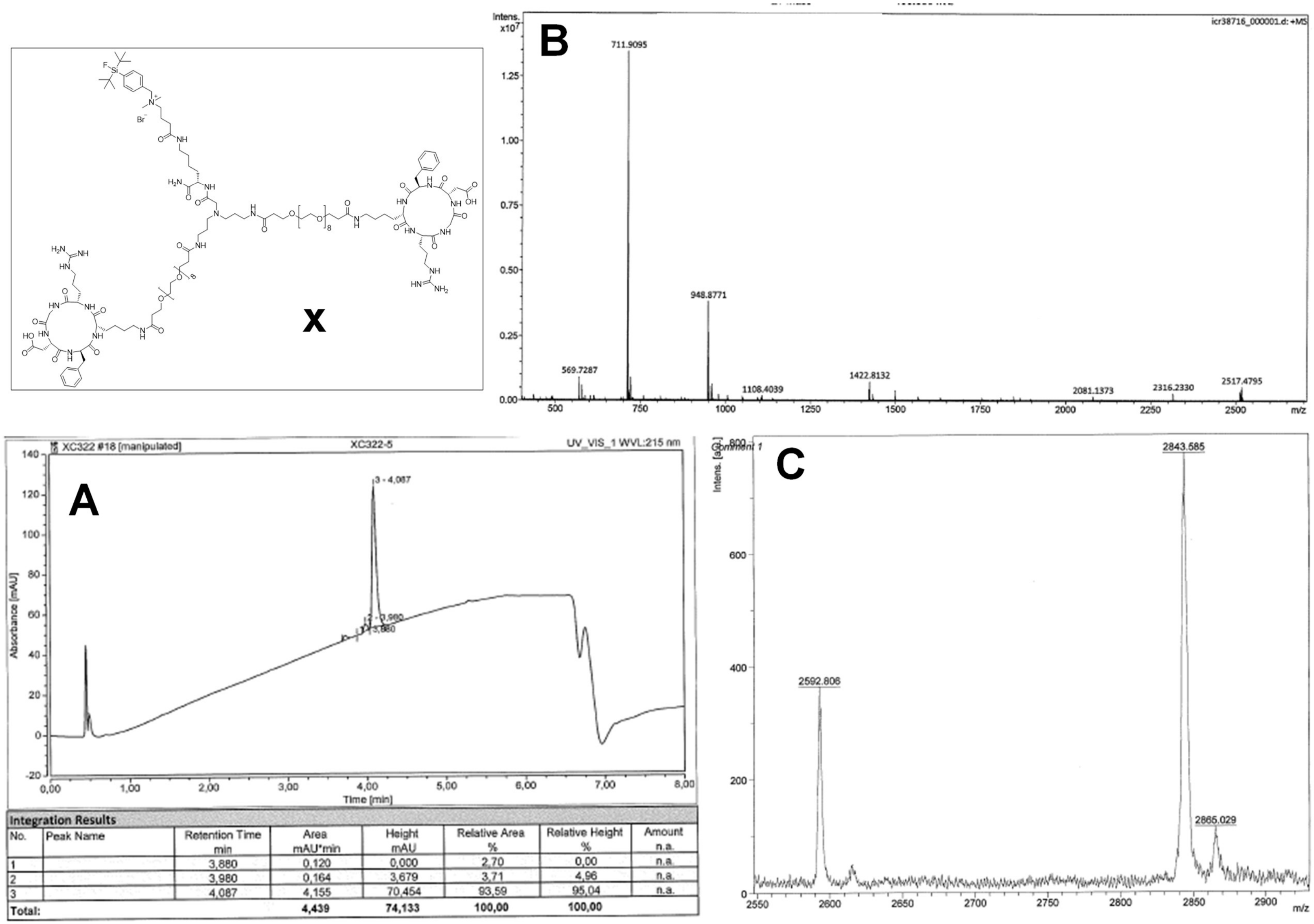

Integration Results

No.	Peak Name	Retention Time min	Area mAU*min	Height mAU	Relative Area %	Relative Height %	Amount n.a.
1		3,880	0,120	0,000	2,70	0,00	n.a.
2		3,980	0,164	3,679	3,71	4,96	n.a.
3		4,087	4,155	70,454	93,59	95,04	n.a.
**Total:**			**4,439**	**74,133**	**100,00**	**100,00**	

Figure S88. HPLC chromatogram (**A**) and mass spectra (**B**: ESI, **C**: MALDI) of SiFA*lin*-APG-[PEG$_8$-c(RGDfK)]$_2$ (**x**).

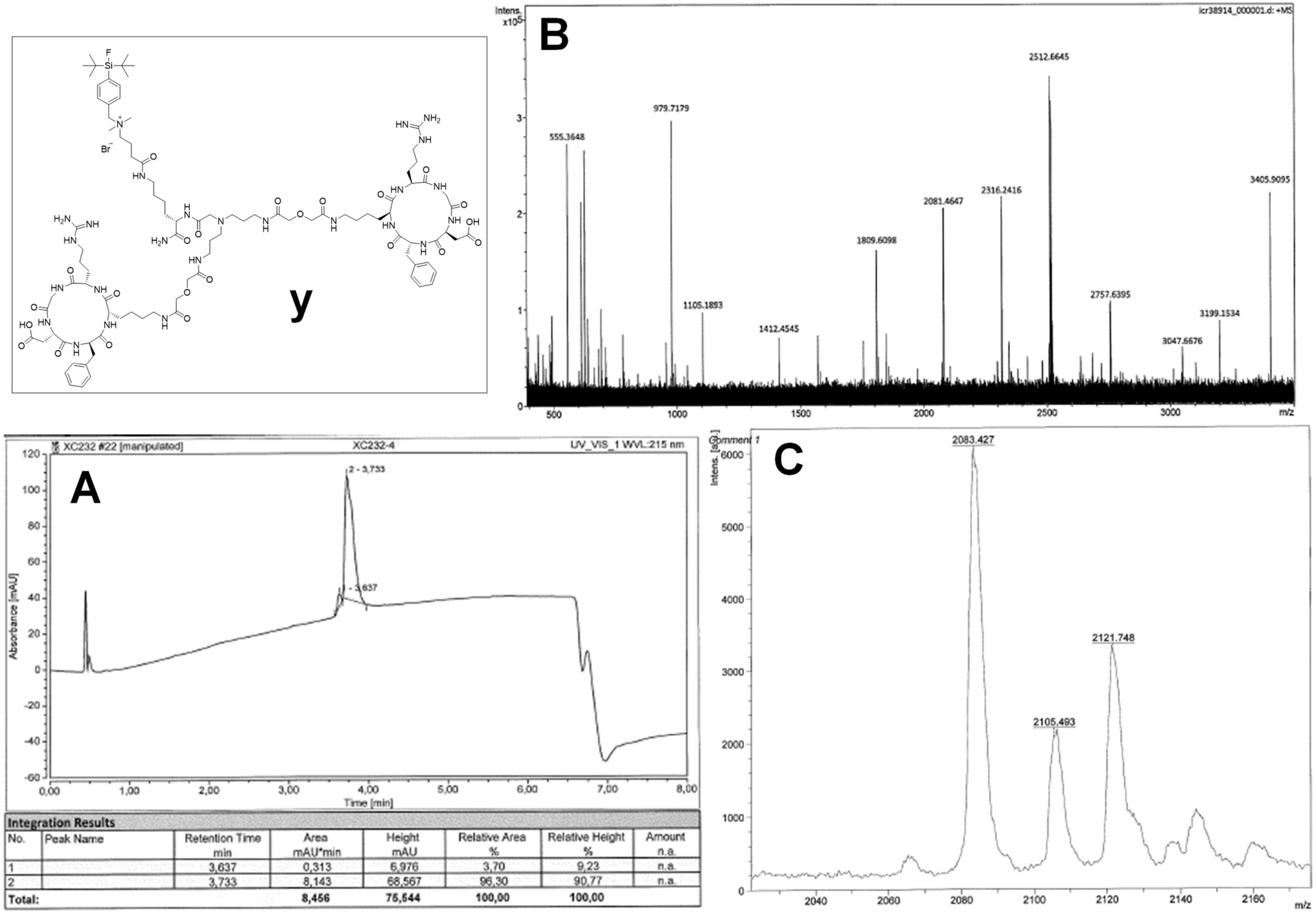

Integration Results							
No.	Peak Name	Retention Time min	Area mAU*min	Height mAU	Relative Area %	Relative Height %	Amount n.a.
1		3,637	0,313	6,976	3,70	9,23	n.a.
2		3,733	8,143	68,567	96,30	90,77	n.a.
Total:			8,456	75,544	100,00	100,00	

Figure S89. HPLC chromatogram (**A**) and mass spectra (**B**: ESI, **C**: MALDI) of SiFA*lin*-APG-[DIG-c(RGDfK)]$_2$ (**y**).

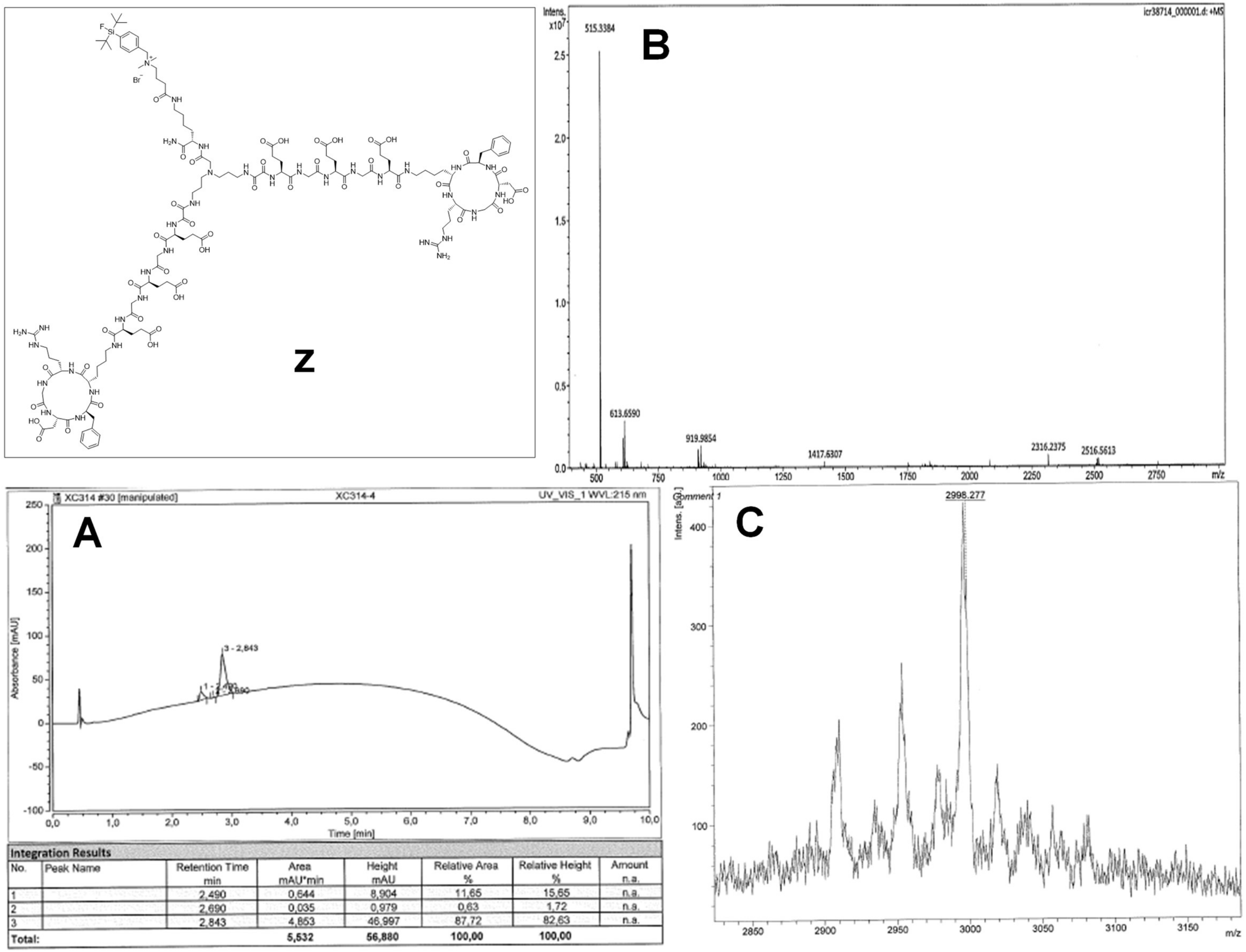

Integration Results

No.	Peak Name	Retention Time min	Area mAU*min	Height mAU	Relative Area %	Relative Height %	Amount n.a.
1		2,490	0,644	8,904	11,65	15,65	n.a.
2		2,690	0,035	0,979	0,63	1,72	n.a.
3		2,843	4,853	46,997	87,72	82,63	n.a.
**Total:**			**5,532**	**56,880**	**100,00**	**100,00**	

Figure S90. HPLC chromatogram (**A**) and mass spectra (**B**: ESI, **C**: MALDI) of SiFA*lin*-APG-[Ox-EGEGE-c(RGDfK)]$_2$ (**z**).

5. Radio-HPLC chromatograms of [^{18}F]1-[^{18}F]6 from the investigation regarding serum stability

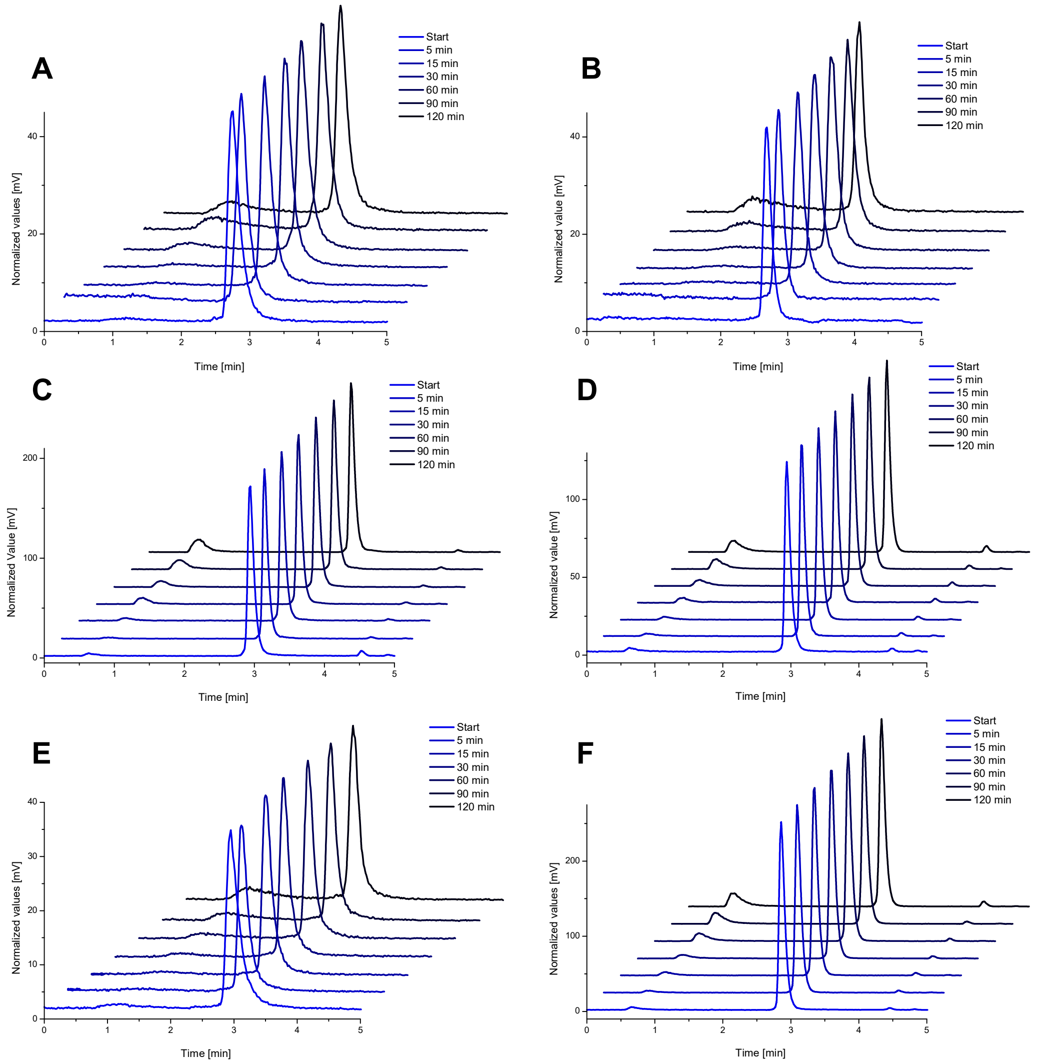

Figure S91. Depiction of the radio-HPLC chromatograms for [^{18}F]**1** – [^{18}F]**6** at certain time points. **A** [^{18}F]**1**; **B** [^{18}F]**2**; **C** [^{18}F]**3**; **D** [^{18}F]**4**; **E** [^{18}F]**5**; **F** [^{18}F]**6**.

6. Binding curves of the monomeric peptides c(RGDfK) and GG-Nle-c(DHfRWK)

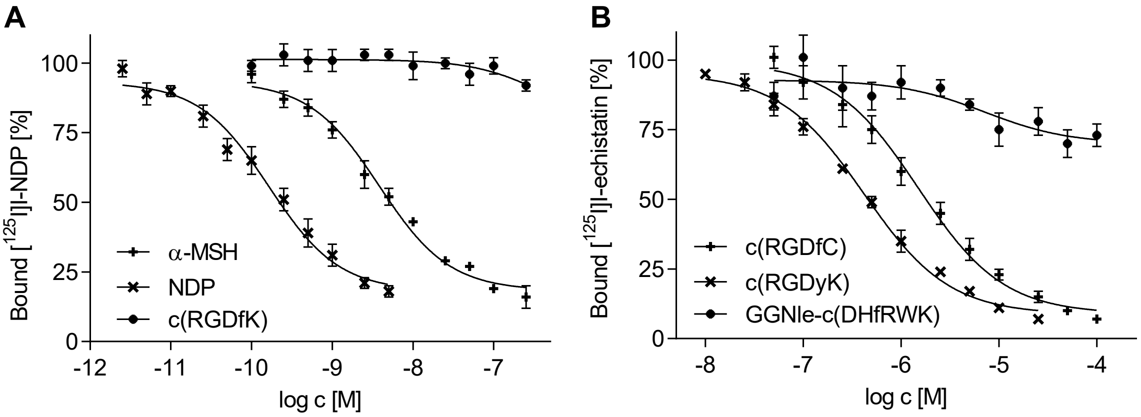

Figure S92. Depiction of the determined binding curves of c(RGDfK) on MC1R-positive B16F10-cells (**A**) and GG-Nle-c(DHfRWK) on $\alpha_v\beta_3$-positive U87MG-cells (**B**). Values are depicted as mean (n = 9), error bars represent SD.

7. *Ex vivo* biodistribution data of [^{18}F]2 and [^{18}F]4

Table S1. Determined ID/g-values of *ex vivo* biodistribution data of [^{18}F]**2** and [^{18}F]**4**. Values are given as mean (n = 3) ± SD.

Tissue	ID/g ([^{18}F]**2**) [%]				ID/g ([^{18}F]**4**) [%]			
	[^{18}F]**2**	NDP blocking	c(RGDyK) blocking	double blocking	[^{18}F]**4**	NDP blocking	c(RGDyK) blocking	double blocking
blood	0.99 ± 0.23	0.84 ± 0.12	1.14 ± 0.15	1.13 ± 0.10	0.59 ± 0.08	2.07 ± 0.94	0.83 ± 0.06	1.00 ± 0.15
spleen	2.73 ± 0.85	2.75 ± 0.55	0.94 ± 0.19	1.07 ± 0.32	1.79 ± 0.31	1.51 ± 0.15	1.71 ± 0.62	0.69 ± 0.10
liver	17.20 ± 6.58	14.88 ± 5.07	4.94 ± 1.39	8.50 ± 2.71	8.30 ± 2.77	5.16 ± 0.56	10.37 ± 3.65	4.04 ± 1.06
kidneys	13.24 ± 3.57	17.27 ± 2.08	12.09 ± 2.30	24.02 ± 6.34	7.72 ± 2.57	23.11 ± 4.97	21.35 ± 4.04	26.54 ± 6.59
pancreas	0.91 ± 0.29	0.80 ± 0.15	0.36 ± 0.04	0.55 ± 0.15	0.25 ± 0.08	0.52 ± 0.12	0.66 ± 0.19	0.32 ± 0.04
lung	2.89 ± 1.04	2.98 ± 0.87	1.07 ± 0.15	1.30 ± 0.36	3.45 ± 1.15	1.25 ± 0.06	2.75 ± 1.04	0.88 ± 0.14
heart	1.24 ± 0.36	1.06 ± 0.31	0.62 ± 0.09	0.93 ± 0.18	0.56 ± 0.19	0.73 ± 0.06	1.51 ± 0.57	0.52 ± 0.06
brain	0.18 ± 0.02	0.13 ± 0.03	0.13 ± 0.05	0.07 ± 0.01	0.10 ± 0.03	0.19 ± 0.07	0.07 ± 0.02	0.07 ± 0.01
bone	8.01 ± 3.48	9.74 ± 2.85	4.12 ± 0.73	4.47 ± 1.57	18.07 ± 6.02	4.61 ± 0.71	4.99 ± 0.71	2.99 ± 0.78
muscle	4.37 ± 2.58	1.37 ± 0.26	0.38 ± 0.04	0.34 ± 0.07	0.07 ± 0.02	0.63 ± 0.07	1.62 ± 0.42	2.58 ± 1.28
tail	37.99 ± 14.37	37.64 ± 8.67	68.98 ± 3.84	53.33 ± 12.82	25.29 ± 8.43	52.78 ± 5.59	44.27 ± 12.11	55.60 ± 7.12
B16F10 tumor	2.32 ± 0.49	1.83 ± 0.24	1.98 ± 0.18	1.14 ± 0.42	2.58 ± 0.86	1.33 ± 0.27	2.29 ± 0.24	2.49 ± 0.09
U87MG tumor	2.33 ± 0.46	3.19 ± 0.33	1.48 ± 0.12	1.00 ± 0.11	3.92 ± 1.31	2.53 ± 0.15	2.67 ± 0.66	0.90 ± 0.05
stomach	1.56 ± 0.29	1.52 ± 0.31	0.54 ± 0.04	0.54 ± 0.12	0.38 ± 0.13	1.03 ± 0.11	0.99 ± 0.16	0.42 ± 0.03
small intestine	2.46 ± 1.77	2.75 ± 0.36	0.75 ± 0.11	0.90 ± 0.23	0.07 ± 0.02	1.60 ± 0.26	1.46 ± 0.53	0.54 ± 0.08
colon	1.58 ± 0.44	1.44 ± 0.34	0.50 ± 0.07	0.63 ± 0.20	0.77 ± 0.26	0.96 ± 0.13	0.80 ± 0.32	0.42 ± 0.05

Table S2. Determined tumor-to-blood, tumor-to-muscle, tumor-to-liver, and tumor-to-kidney ratios of [^{18}F]**2** and [^{18}F]**4**. Values are given as mean (n = 3) ± SD. Asterisks indicate statistically significant values (*: $p < 0.05$, **: $p < 0.01$ and ***: $p < 0.001$).

Ratio	[^{18}F]**2**		[^{18}F]**4**	
	B16F10	U87MG	B16F10	U87MG
tumor/blood	2.42 ± 0.73	2.40 ± 0.67	4.36 ± 1.37	5.08 ± 0.75**
tumor/muscle	0.68 ± 0.41	0.79 ± 0.66	38.56 ± 9.24**	45.72 ± 8.62***
tumor/liver	0.16 ± 0.11	0.14 ± 0.03	0.36 ± 0.23	0.39 ± 0.14
tumor/kidneys	0.19 ± 0.10	0.18 ± 0.02	0.38 ± 0.24	0.40 ± 0.07**

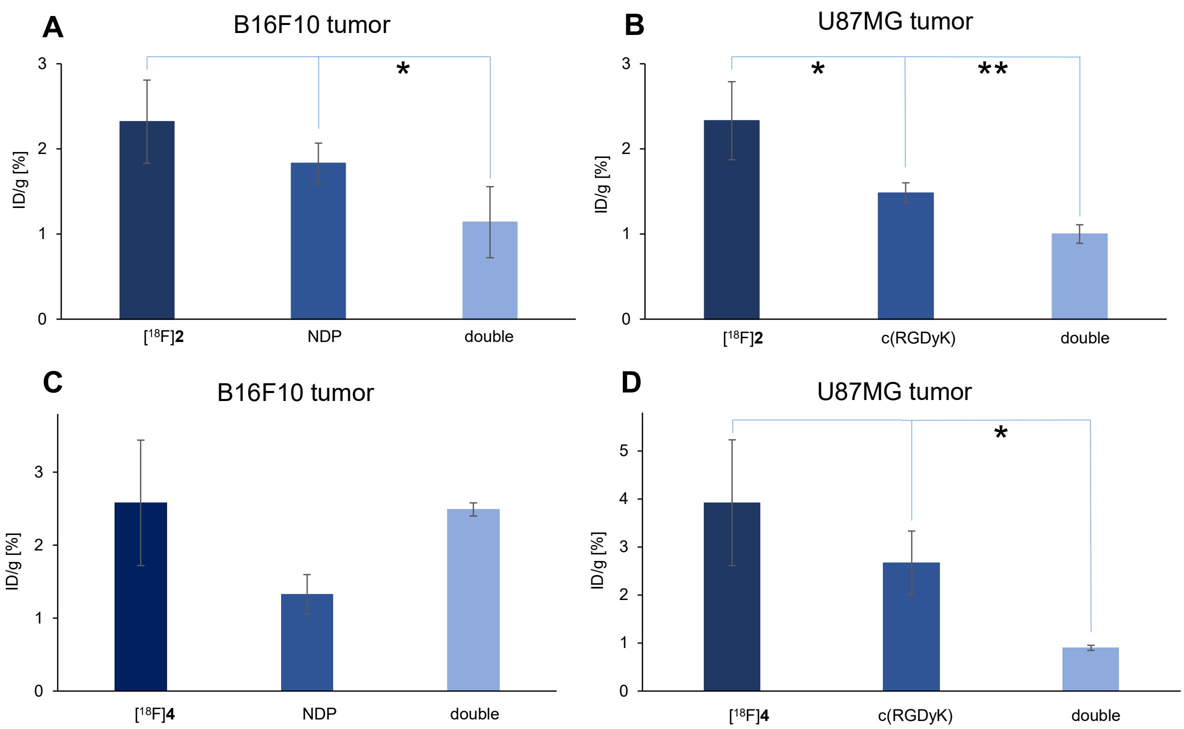

Figure S93. Depiction of tumor uptake of [^{18}F]**2** and [^{18}F]**4** with the respective blocking substances. **A** [^{18}F]**2** in B16F10 tumor; **B** [^{18}F]**2** in U87MG tumor; **C** [^{18}F]**4** in B16F10 tumor; **D** [^{18}F]**4** in U87MG tumor. Values are depicted as mean (n = 3), error bars represent SD.
